# Analysis of DNMT1 gene variants in progression of neural tube defects—an *in silico* to *in vitro* approach

**DOI:** 10.1042/BSR20220998

**Published:** 2022-12-06

**Authors:** Susanta Sadhukhan, Nirvika Paul, Sudakshina Ghosh, Dinesh Munian, Kausik Ganguly, Krishnendu Ghosh, Mainak Sengupta, Madhusudan Das

**Affiliations:** 1 Department of Zoology, University of Calcutta, 35 Ballygunge Circular Road, Kolkata 700019, India; 2 Department of Zoology, Vidyasagar College for Women, 39 Sankar Ghosh Lane, Kolkata 700006, India; 3 Department of Neonatology, Institute of Postgraduate Medical Education and Research, 244 Acharya Jagadish Chandra Bose Road, Kolkata 700020, India; 4 Department of Genetics, University of Calcutta, 35 Ballygunge Circular Road, Kolkata 700019, India

**Keywords:** in silico, DNMT1, Epigenetics, Methylation, Neural tube defects (NTDs), Spina bifida

## Abstract

Neural tube defects (NTDs) are significant congenital deformities of the central nervous system among which spina bifida is the most common form that occurs due to defect in the neurulation process of embryogenesis. NTDs are among the most common type of birth defects occurring at a range of 0.5–10 in every 1000 live births worldwide and are thought to have multifactorial etiology, including multigenetic and epigenetic notions. Epigenetic regulations control differential gene expression in normal and disease phenotypes. DNA methylation is a significant epigenetic process, guided by DNMT1, one of the most important maintenance methylating agents. However, the relationship between DNMT1 and NTDs had always been inconclusive and poorly understood. In the present study, by utilizing *in silico* methodologies we tried to figure out potent single nucleotide variants (SNVs) that could play roles in generating functional differences in DNMT1 expression and we also tried to check (by *in vitro* method) if there is any connection between DNMT1 expression and spina bifida condition. A number of coding and non-coding (both intragenic and intergenic) SNVs of *DNMT1* were found (using the *in silico* methods) that have potentials to alter its expression. From the *in vitro* experimentations, differential *DNMT1* RNA expressions were found between spina bifida affected newborns and their respective mothers when compared with controls. It is the first report of NTD from Eastern India precisely showing inverse correlation between *DNMT1* expression and occurrence of NTD. The findings of the present study could be further considered for early prognosis and future experimental designs.

## Introduction

Chemical modification of DNA or histone protein is mediated by epigenetic processes such as methylation, acetylation, microRNAs and ubiquitination. DNA methylation is a covalent modification occurring at the cytosine residues in CpG sites near the regulatory regions of genes with lasting inheritable effects without changing the sequence. The DNA methylation is mediated by a family of enzymes, are known as DNA methyltransferases (DNMTs) [1].

These enzymes (DNMTs) are of two classes: one is maintenance methyltransferases (*DNMT1*, cytogenetic location: 19p13.2, OMIM *126375), most abundant form and prefer for methylation of hemi methylated DNA and other one is *de novo* methyltransferases (DNMT3A and DNMT3B) to establish tissue specific DNA methylation pattern during development [2]. DNMTs catalyze the transfer of methyl group from S-Adenosyl methionine (SAM) to the cytosine residue in CpG dinucleotides of DNA. This methylation of cytosine helps in the formation of 5-methylcytosine and consequently abundance of 5-methylcytosine in their promoter to promote the region-specific silencing process. This in course can affect gene transcription by altering the accessibility of RNA polymerase and transcription factors [3].

This epigenetic regulation is vital during growth and developmental processes. Inconsistency often results into numbers of developmental disorders. DNMT1 shows activity in adult nervous system although its specific function is poorly understood. The enzyme is thought to have regulative roles in neuronal survival, maturation, differentiation, migration and intra-inter neuronal connections. Variations in this gene has been witnessed to be associated with cerebellar ataxia, deafness, and narcolepsy, and neuropathy, hereditary sensory. It has also been shown that a lack of both maternal and zygotic DNMT1 gene expression results into complete demethylation of imprinted genes in blastocysts causing developmental anomalies [4]. In human, *DNMT1 gene* controls the expression of the maternally imprinted genes like—insulin-like growth factor 2 (*IGF2*), paternally expressed 3 (*PEG3*) and long interspersed nuclear elements1 (*LINE1*) methylation. However, during embryogenesis *DNMT1* regulates the process of neural tube formation and its closing, any changes in the imprinted gene can play a crucial role leading to neural tube defects (NTDs) [5]. NTDs are one of the most common and severe congenital malformations. Each year, nearly 300,000 babies worldwide [6] and 2.41–4.1 babies per 1000 live births in India are affected by NTDs [7,8]. Most NTDs are sporadic in nature, with multifactorial polygenic or oligogenic patterns combined with epidemiological and biochemical risk factors [9,10]. Thus, it seems possible that genetic variations in *DNMT1* could influence some of the previously described proteins that need to be considered to understand the progression of NTDs.

On the other hand, genetic association studies of nucleotide variants of *DNMT1* gene are scattered and sporadic and fail to describe the functional effects of concerned single nucleotide variants (SNVs). Thus, in the present study we had tried to analyze and assess role(s) of *DNMT1* (if any) through both *in silico* and *in vitro* approaches among two case mothers (both aged 28 years) of two different societal background from Suburban areas of West Bengal and their respective affected newborns (one from each). Both the babies were having spina bifida in terms of NTD and subsequently died at the age of around 5 weeks. Samples procured from two normal mothers were considered as controls. However, no sample had been procured from normal newborns due to ethical and emotional constrains.

## Methods

### Selection of genetic variants of DNMT1 for *in silico* analyses

Intragenic (non-synonymous, synonymous and intronic) SNVs were procured from dbSNP and intergenic variants were searched with Table Browser tool of UCSC genome browser [11] using GRCh37/hg19 assembly and genomic co-ordinates of the DNase hypersensitive sites (DHS). Regulatory Element Database [12] was searched to find out coordinates of correlated DHS of *DNMT1*.

### Analyses of non-synonymous variants (non-syn SNVs)

Any non-synonymous single nucleotide variant (non-syn-SNV) substitutes the canonical amino acid sequence of a protein, thus, we tried to predict the functional effect(s) (if any) of every such nucleotide substitution (tabulated in dbSNP for *DNMT1*) in terms of sequence conservation, disease related functional annotations and overall stability of protein structure. SIFT [13], PROVEAN [14] and PolyPhen-2 [15] consider multiple sequence alignments and conservation of a particular amino acid residue within a particular position of the concerned protein. We used the ‘PROVEAN HUMAN PROTEIN BATCH’ submission option and submitted the substitutions to be analyzed in the prescribed format. PROVEAN calculates and considers a cut off score of -2.5 for each of the amino acid substitution submitted for analysis. If the individual score of each substitution is less than -2.5, then that substitution is considered ‘Deleterious’, on the other hand, a cut-off of 0.05 is considered with SIFT analyses, where any amino acid substitution procuring a score less than 0.05 is considered as ‘Damaging’ substitution. In PolyPhen-2, we used the ‘Batch Query’ option; it calculates conservation score for each submitted query and gives prediction results as ‘Probably Damaging’, ‘Possibly Damaging’ or ‘Benign’. SNPs&GO [16] considers coherent descriptions of gene products in terms of their associated biological processes, cellular components and molecular functions. Thus, a prediction made by SNPs&GO annotates an amino acid substitution as ‘Disease’ related or ‘Neutral’ with a Reliability Index (R.I.) score within the numerical range of ‘1 to 10’ (more the R.I. score, stronger the prediction is). To get the results using SNPs&GO, we submitted each amino acid substitution separately in the required format. In contrast to the four tools already mentioned, fathmm [17] and I-Mutant Suite [18] consider changes in structural stability of concerned protein based on pre-defined structural attributes like hidden markov model (HMM) and Support Vector Machine (SVM) based calculations, respectively. Whereas, fathmm confers an amino acid substitution as ‘Damaging’ or ‘Tolerated’, I-Mutant Suite calculates ‘Increase’ or ‘Decrease’ in protein stability based on SVM calculations and provides change in free energy values (in Kcal/mol unit). Here, we used the ‘Inherited Disease’ option of fathmm and submitted our queries in a batch. For I-Mutant Suite, we used ‘Prediction of protein stability changes upon single point variation from *Protein Sequence*’ option. For all the web-tools discussed so far, only the default options were used for our analyses. We used all the above tools, except I-Mutant Suite, for prioritization of non-syn-SNVs. Predictions of I-Mutant Suite were tabulated to check if DNMT1 protein stability increases or decreases due to any single nucleotide variation, as both increase and decrease in protein stability may contribute role toward disease pathogenesis. Finally, the pdb file 4WXX was used to assess the structural alterations like changes in hydrogen bonds, electrostatic potential and introduction of steric clash for the most deteriorating non-synonymous variants as assessed by above mentioned tools, using DeepView - Swiss-PdbViewer [19].

### Analyses of synonymous variants (syn SNVs)

We know that any synonymous single nucleotide variant (Syn-SNV) does not bring in any amino acid substitution in the polypeptide chain of concerned protein but it creates functional changes in protein turn-over rate. We analyzed all the Syn-SNVs of *DNMT1* tabulated in dbSNP for predicting their possible impacts (if any) in changing codon usage from codon table provided in Codon Usage Database (https://www.kazusa.or.jp/codon/). In doing so, we considered change in ‘fraction’ values before and after a particular nucleotide substitution. A Syn-SNV may contribute in alteration of secondary mRNA structure, which can further change the tertiary and biologically active mRNA structure. To predict the effects of syn-SNVs of *DNMT1*, we utilized RNA Folding form of mfold Web Server (http://www.unafold.org/mfold/applications/rna-folding-form-v2.php) [20]. All the options of RNA folding form were kept as default and no modification was made. RNA folding form considers all the probable stem-looped structures of the submitted single strand sequence and gives out results on a hierarchical basis of initial Δ*G* values for all the probable stem-looped secondary structures possible, under the ‘View Individual Structures’ section. We checked and compared the circular structure plots (available with ‘Structure 1’ of each result, representing the most stable secondary folding) for all the alleles concerned with a syn-SNV, noted the change in delG values within result table and checked the comparative pictures of the circular plots of our results. Finally, to assess the impact of a syn-SNV upon exon skipping, we took help of Ex-skip [21]. Ex-skip incorporates Exon Splicing Enhancer/Exon Splicing Suppressor (ESE/ESS) profile between ‘wild type’ and ‘mutant’ nucleotide residues for each of the syn-SNV tested. We tried to check if any syn-SNV incorporated in our study has more or less chance of exon skipping than its other alleles.

### Analyses of intronic and intergenic variants for their potential regulatory roles

Intronic SNVs of *DNMT1* recruited from dbSNP and intergenic SNVs recruited using Regulatory Element Database and UCSC genome browser were all tested for checking their probable regulatory roles following the study of rSNPBase (http://rsnp.psych.ac.cn/listSearch.do) [22] utilizing rSNPBase [23]. RegulomeDB [24] and Haploreg v4.1 [25], all of which entails functional experiment-based predictions available with ENCODE database (The ENCODE Project Consortium, 2012). rSNPBase helped in identifying potential target loci based on spatio-temporal and experimental eQTL (expression Quantitative Trait Loci) labels. Linkage disequilibrium (LD) correlations between SNVs are also analyzed to get the results of probable functional regulatory effects of query SNVs in the form of a SNV-set basis, rather than individual single result for each query SNV. In this study, we used ‘List search’ option of rSNPBase (http://rsnp.psych.ac.cn/listSearch.do). SNV IDs were submitted in the search box in enter delimited format, followed by the searching step. It is to be noted that for each query, the ‘rSNP’ column of the result page showed ‘yes’ if any experimental data are available; otherwise ‘no’. We did not include results with ‘no’ output in our filtration. SNVs that were found to have regulatory effects during rSNPBase analyses were searched further in RegulomeDB. RegulomeDB provides a heuristic scoring system, with increasing confidence, for each query variant based on its functional location and consequence, if any. The scoring system ranges from 1 to 6. Score category 1 and all its subcategories indicate that the variant is ‘likely to affect binding and linked to expression of a gene target’. Score category 2 along with its sub-categories; indicate that the query variant is ‘likely to affect binding’. Categories 3 to 6 represent those variants for whom lesser evidences are there with respect to their regulatory potentials. The ‘dbSNP IDs’ option of RegulomeDB was used in this study and all the SNV IDs were submitted in enter delimited format. RegulomeDB (http://www.regulomedb.org) involved manually curated regions that have been experimentally characterized to be involved in regulation, ChIP-seq information for a variety of important regulatory factors across diverse set of cell types, chromatin state information across different cell types and eQTL information to allow the association of distal sites with gene promoters. HaploReg v4.1 (http://archive.broadinstitute.org/mammals/haploreg/haploreg.php) helps to search SNVs within LD blocks based on chromatin state data along with conservation and regulatory motif alteration data for all query SNVs. SNV Ids that obtained score 1 and score 2 during RegulomeDB analysis, were pooled together and further searched in HaploReg v4.1. For searching purpose, we created and uploaded a text file with SNV IDs in enter delimited format having only one SNV ID per line. Before searching, the LD threshold value of HaploReg was set to 0.6 and other setting options were left unchanged. HaploReg v4.1 was used only to check if any of our prioritized SNV was in LD with another query SNP, or not. Therefore, it is worth mentioning that HaploReg v4.1 was not used to prioritize SNVs.

### Searching targets of regulations and modes of regulations

After prioritization of intronic and intergenic variants using the afore-mentioned tools, we tried to find the target loci for each of the prioritized potential regulatory single nucleotide variants (rSNVs) of *DNMT1*. We followed the hyperlinks provided by rSNPBase (on the SNV identifiers) and checked the ‘SNP Report’ page. Types of regulations and targets of such regulations were checked for each query rSNVfrom ‘SNP Report’‘ page. It is important to mention here that the target locus (or loci) of each rSNV provide a hint for plausible functional implication of the variant in question.

### STRING v11 analyses

‘The STRING database aims to collect, score and integrate all publicly available sources of protein-protein interaction information, and to complement these with computational predictions. Its goal is to achieve a comprehensive and objective global network, including direct (physical) as well as indirect (functional) interactions’. [26]. After prioritization of intronic and intergenic SNVs for checking their potential regulatory role(s) (if any), we tried to check if the target loci fetched from ‘SNP Report’ page of rSNPBase, showed any interaction among themselves or with *DNMT1*.

### Collection of samples and meta-data

**Figure 1:**
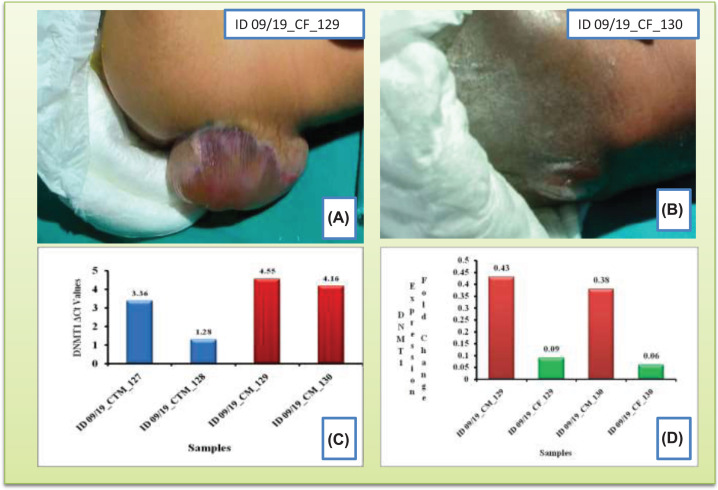
NTD and DNMT 1 gene expression (**A,B**) Pre-mortal photographs of subject newborns with NTD/ Spina bifida. (**C**) The column histogram reflecting the comparison of DNMT1 expression in terms of ΔCt values between control mother (CTM, in blue) and case mother (CM, in red) who gave birth to NTD babies (assuming that higher the Δ*C*t, lower will be the expression. In the current study higher DNMT1 expression was seen in control mothers than case mothers). (**D**) The column histogram reflecting the comparison of DNMT1 expression fold change (2^−ΔΔCt^) between case mother (CM, in red) and their respective fetus (CF, in green) having NTD reflecting higher expression in case mothers that their respective fetus.

The peripheral blood samples of both mothers and their NTD babies (Figure 1A) were collected from the Department of Neonatology, Institute of Post Graduate Medical Education & Research (IPGME&R), Kolkata, India. Age and ethnicity matched control samples with no familial history of NTDs were recruited from the same clinic. Socio-economic condition, occupation of parents, smoking and drinking habits, regular food habit, folate intake during pregnancy, pregnancy term, status of diabetes and any previous family history of congenital defect, were also considered. The clinical and demographic characteristics of case and control mothers showed that the socio-economic status of case and control groups were similar. None of the mother had any smoking or drinking habits. Both case and control patient groups were on routine folate supplementation. In term of pregnancy all mothers are primigravida. Diabetes condition was not found in any case or control subject.

The entire study was kept under the loop depending on the questionnaire approved by ethical committee of IPGME&R, Kolkata [Memo No. Inst/IEC/2015/43] abiding by the Declaration of Helsinki of World medical Council. All the participants were being told meticulously about the research utilities, outcomes and also their orientations of association in their own dialect(s) prior taking their confirmation (in writing, from the patients themselves or their related keen, as applicable in time) willing to participate in this said study abiding by all the medico-legal norms (without any pay-off in cash or kind either) under close monitoring of the concerned medical team.

### qRT PCR

Quantitative mRNA expression was analyzed using real-time quantitative PCR (qRT-PCR). Respective primers were CAACGGATTTGGTCGTATTGG (FP) and GCAACAATATCCACTTTACCAGAGTTAA (RP) for GAPDH as housekeeping & CCCCTGAGCCCTACCGAAT (FP) and CTCGCTGGAGTGGACTTGTG (RP) for DNMT1 as methylation marker. Product sizes of the primers were calculated from ‘NCBI PCR PRIMER BLAST’ and their optimum annealing temperature were determined by array of gradient PCR runs. Both the primers were having Tm of 59.1°C. The product length of DNMT1 was 142 bp while product length of GAPDH was 72 bp. ‘SYBR Green I master mix’ (Agilent Technologies, U.S.A.) was used for fluorescent master mix along with ‘ROX’ (Agilent Technologies, U.S.A.) as reference dye diluted in DEPC water (1:100). Entire reaction mixture along with respective primers, DEPC water and cDNA was taken into ‘AriaMX Real Time PCR cycler’ (Agilent Technologies, U.S.A.). The amplification and melt curve were obtained along with *C*q values by analyzing with ‘AriaMX Software v1.0’ (Agilent Technologies, U.S.A.). This *C*q and successive Δ*C*t values (by substracting Housekeeping GAPDH *C*q value from DNMT1 *C*q) had been plotted graphically. The lesser the Δ*C*t value, greater will be the expression level. Moreover, fold changes in expression were calculated in terms of ΔΔ*C*t value and plotted furthermore, where needed.

## Results

### Result of selection of genetic variants of DNMT1 for *in silico* analyses

We found 775 non-syn SNVs, 576 syn-SNVs, 14336 intronic SNVs from dbSNP during SNV curation. When we searched Regulatory Element Database for number of DHS correlated with expression of *DNMT1*, we found 1781 DHS to be positively correlated and 133 DHS to be negatively correlated with *DNMT1* expression. We found 129 SNVs from all the positively correlated DHS and 9 SNVs from the negatively correlated DHS which we termed as intergenic SNVs.

### Result of prioritization of non-synonymous SNVs (non-syn SNVs)

As mentioned, we found 775 non-syn SNVs for *DNMT1* from dbSNP and after analyzing them, we found 227, 277, 171, 284, 190 (Supplementary Table S1) non-syn SNVs to get annotated as - ‘deleterious’, ‘damaging’, ‘disease’ causing, ‘damaging’ and ‘damaging’ substitutions by PROVEAN, SIFT, SNPs&GO, PolyPhen 2 and fathmm, respectively. We found 17 non-syn SNVs (Table 1) being commonly predicted by all the aforementioned tools to have ‘damaging’, ‘deleterious’ or ‘disease’ causing effects upon respective substitutions. Of those 17 non-syn SNVs, 6 SNVs [S1556F (rs1388362405), R1555C (rs1461695373), G1449R (rs770571074), R1261W (rs1052868434), G806R (rs183555527), D785H (rs1244845928)] were found to have changes in number of hydrogen bonds, changes in electrostatic potentials and also introduction of steric clashes upon allelic alterations when assessed through DeepView - Swiss-PdbViewer (see Table 2 and Supplementary Figure S4) thus, these six non-synonymous SNVs can be termed as most potent and their roles can further be assessed using functional experimentations.

**Table 1:** Summary of non-synonymous single nucleotide variant (Non-syn-SNV) analyses of DNMT1

Non-synonymous SNV	rs930531589	rs1388362405	rs1461695373	rs770571074	rs1182970957	rs1052868434	rs759915904	rs1167927296	rs776149246	rs1303994790	rs1314966532	rs868584117	rs183555527	rs1244845928	rs1181332562	rs199473690	rs199473692
Amino acid substitution	H1573Q	S1556F	R1555C	G1449R	C1294G	R1261W	G1231R	I996T	Y991C	A874V	F864L	S840C	G806R	D785H	Y775C	Y495C	Y495H
PROVEAN predictions	DELETERIOUS	DELETERIOUS	DELETERIOUS	DELETERIOUS	DELETERIOUS	DELETERIOUS	DELETERIOUS	DELETERIOUS	DELETERIOUS	DELETERIOUS	DELETERIOUS	DELETERIOUS	DELETERIOUS	DELETERIOUS	DELETERIOUS	DELETERIOUS	DELETERIOUS
PROVEAN score (cut off > -2.5)	-6.57	-5.02	-6.82	-6.64	-10.83	-5.85	-7.37	-4.58	-5.77	-3.65	-5.36	-2.98	-7.13	-3.46	-5.11	-8.11	-4.51
SIFT prediction	DAMAGING	DAMAGING	DAMAGING	DAMAGING	DAMAGING	DAMAGING	DAMAGING	DAMAGING	DAMAGING	DAMAGING	DAMAGING	DAMAGING	DAMAGING	DAMAGING	DAMAGING	DAMAGING	DAMAGING
SIFT score (cutoff = 0.05)	0.001	0	0	0	0.004	0.001	0.001	0.001	0.001	0.002	0.045	0.021	0.005	0.004	0.05	0	0
SNPs&GO predictions for effect of substitution	DISEASE	DISEASE	DISEASE	DISEASE	DISEASE	DISEASE	DISEASE	DISEASE	DISEASE	DISEASE	DISEASE	DISEASE	DISEASE	DISEASE	DISEASE	DISEASE	DISEASE
Reliability Index (R.I.) of SNPs&GO prediction	1	0	4	1	2	2	4	4	4	2	4	2	7	1	2	6	6
POLYPHEN2 (HumDiv) predictions	probably damaging	probably damaging	probably damaging	probably damaging	probably damaging	probably damaging	probably damaging	probably damaging	probably damaging	probably damaging	probably damaging	probably damaging	probably damaging	probably damaging	probably damaging	probably damaging	probably damaging
POLYPHEN2 scores	1	1	1	1	0.998	1	1	1	0.999	0.999	0.997	0.999	0.987	0.993	0.999	1	1
fathmm predictions	DAMAGING	DAMAGING	DAMAGING	DAMAGING	DAMAGING	DAMAGING	DAMAGING	DAMAGING	DAMAGING	DAMAGING	DAMAGING	DAMAGING	DAMAGING	DAMAGING	DAMAGING	DAMAGING	DAMAGING
fathmm scores	-1.79	-1.74	-2.62	-1.86	-1.81	-1.87	-1.79	-2.77	-2.37	-2.08	-2.02	-2.04	-2.1	-2.1	-2.08	-3.34	-3.32
Cumulative prediction scores	6	3	5	6	8	6	3	8	4	5	7	1	2	2	2	2	6
Protein stability precition of iMutant	Decrease	Decrease	Decrease	Decrease	Decrease	Decrease	Decrease	Decrease	Decrease	Decrease	Decrease	Decrease	Decrease	Decrease	Decrease	Decrease	Decrease
DDG values (Kcal/mol) of predictions from iMutant 3.0 (DDG<0: Decrease Stability; DDG>0: Increase Stability)	-0.57	0.14	-1.2	-0.63	-1.22	-0.74	-0.34	-1.87	-1.2	-0.11	-1	-0.4	-0.39	-0.38	-1.06	-0.93	-1.22

**Table 2:** Summary of assessment of non-synonymous single nucleotide variants using DeepView - Swiss-PdbViewer

Non-synonymous SNV	Amino acid substitution	Change in hydrogen bonds	Introduction of steric clash	Change in electrostatic potential
**rs930531589**	**H1573Q**	Lost 2 bonds	Gain of 3 hydrogen bonds	None
**rs1388362405**	**S1556F**	Lost 3 bonds	Gain of 1 hydrogen bond and 3 steric clashes	Change found
**rs1461695373**	**R1555C**	Lost 5 bonds	Gain 2 hydrogen bonds	Change found
**rs770571074**	**G1449R**	Lost 1 bond	Gain 3 hydrogen bonds	Change found
**rs1182970957**	**C1294G**	Lost 1 bond	No change	None
**rs1052868434**	**R1261W**	Lost 4 bonds	Gain 2 hydrogen bonds	Change found
**rs759915904**	**G1231R**	Lost 1 bond	Gain 2 hydrogen bonds	None
**rs1167927296**	**I996T**	Lost 1 and Gain 1	Gain 5 hydrogen bonds	None
**rs776149246**	**Y991C**	Lost 1 bond	Gain of 5 hydrogen bonds and 1 steric clash	None
**rs1303994790**	**A874V**	No change	Gain 2 hydrogen bonds	None
**rs1314966532**	**F864L**	Lost 1 bond	Gain 2 hydrogen bonds	None
**rs868584117**	**S840C**	No change	Gain 2 hydrogen bonds	None
**rs183555527**	**G806R**	Lost 1 bond	Gain 3 hydrogen bonds	Change found
**rs1244845928**	**D785H**	Lost 1 bond	Gain of 1 hydrogen bond and 1 steric clashe	Change found
**rs1181332562**	**Y775C**	Lost 1 bond	Lost 1 bond	None
**rs199473690**	**Y495C**	Lost 2 bonds	Gain 4 hydrogen bonds	None
**rs199473692**	**Y495H**	Lost 2 bonds	Gain 4 hydrogen bonds	None

### Result of prioritization of synonymous SNVs (syn SNVs)

We found 576 syn SNVs from dbSNP and checked them all for potential changes in Codon Usage Biasness, alteration in secondary mRNA structures and potential alterations in exon skipping. We found 55 syn SNVs to have 2-fold or more increase and 105 syn SNVs to have 2-fold or more decrease in codon usage upon concerned allelic changes, respectively. After checking all the 576 syn SNVs for changes in secondary mRNA structure, 82syn SNVs (see Supplementary Figure S1) were found to have significant alterations in mRNA secondary structures as evident from the respective ‘circular plots’ and 146 syn SNVs were found to have significant changes in exon skipping after EX-SKIP analyses. Altogether, 72 syn SNVs (see Supplementary Table S2) were found to get commonly predicted as having codon usage changes of 2-fold or more, significant alteration in secondary mRNA structures and chances of altered exon skipping upon concerned allelic changes, which may alter the equilibrium of DNMT1 mRNA at molecular level.

### Result of prioritization of intronic SNVs

We checked 14336 intronic SNVs for assessing their probable regulatory roles (if any). 815 intronic SNVs out of 14336 were assigned as ‘rSNP’ after rSNPBase analyses. After RegulomeDB analyses, 65 intronic SNVs obtained scores 1 or 2 which further support their regulatory activities with maximum evidences in form of functional data available with RegulomeDB. Out of the 65 intronic SNVs 9 were found to be in LD with other query intronic SNVs (see Supplementary Table S3).

### Result of prioritization of intergenic SNVs

We found 1781 intergenic SNVs to reside within positively correlated DHS and 133 intergenic SNVs to reside within negatively correlated DHS of *DNMT1*. After rSNPBase analyses, we found 129 SNV of positively correlated DHS and 9 SNV of negatively correlated DHS to be assigned as ‘rSNP’. All the ‘rSNP’s were then subjected to RegulomeDB analyses and 29 ‘rSNP’ from positively correlated DHS and 2 ‘rSNP’ from negatively correlated DHS were found to obtained either score 1 or 2 with maximal evidences in support of their regulatory activities. After HaploReg v4.1 analysis we found no SNV from positively correlated DHS to be in LD with other query SNVs (see Supplementary Table S4).

### Result of searching targets of regulatory SNVs of DNMT1

All the prioritized 65 intronic SNVs were found to target 40 different loci other than *DNMT1* through ‘distal transcriptional regulation’. On the other hand, 29 prioritized intergenic SNVs from positively correlated DHS of *DNMT1* were found to target 10 loci other than *DNMT1* through ‘proximal transcriptional regulation’, 74 loci other than *DNMT1* through ‘distal transcriptional regulation’ and 6 different loci through ‘RNA binding protein mediated regulations’. Two prioritized intergenic SNV from negatively correlated DHS were found to target only *EIF3G* through ‘proximal transcriptional regulation’ and 7 different loci other than *DNMT1* through ‘distal transcriptional regulation’. Altogether, prioritized rSNVs of *DNMT1* were found to target 76 different loci other than *DNMT1* through different modes of regulations.

### Result of STRING v11.0 analyses

After intronic and intergenic SNV specific analyses we found 76 different loci to be targeted by *DNMT1* harboring rSNVs. But after STRING analyses we found only 4 loci (HIST1H3H, Eukaryotic translation initiation factor 3 subunit G or EIF3G, Mitochondrial Ribosomal Protein L4 or MRPL4 and Eukaryotic Translation Elongation Factor 2 or EEF2) to reside within the same interactome with *DNMT1* (see Supplementary Figure S2) when ‘experiment’, ‘co-expression’, ‘gene fusion’ specific filters of STRING were applied i.e. filters associated with functional experimental data.

### qRT-PCR Result

The DNMT1 expression in term of SYBR expression had been considered while keeping GAPDH as the housekeeping expression. The Figure 1C suggested lower Δ*C*t (higher expression level of DNMT1) in control mother (blue histogram columns) than the NTD case mothers (red histogram columns). On the other hand, DNMT1 related maintenance methylation had mostly been observed in experimental case mothers (red histogram columns) yielding higher level of 2^−ΔΔCt^ expression fold change of 0.43 and 0.38, respectively. However, in their respective NTD fetus (green histogram columns of Figure 1D), the degree of subsequent DNMT1 expression also being reduced giving lower level of 2^−ΔΔCt^ expression fold change of 0.09 and 0.06, respectively. Thus, from this experimental case analysis it was evident that decrease of DNMT1 expression is coherent with the disease both in case mothers and their respective NTD fetus when compared with control mothers.

## Discussion

DNA methylation is a major epigenetic modification predominantly involved in eukaryotic gene silencing. During embryonic development, future lineage specific differentiations without any unwanted cellular regression are carried out by the DNMT genes [27]. DNMT1, the predominant maintenance methylator, contains numerous regulatory domains finely tuned with normal embryological development in mammals. Any DNMT1 related change in the imprinted genes and transposable elements responsible for normal neurulation process, could result NTD in offspring [5].

All the curated SNVs of *DNMT1* were checked for their plausible deleterious effects which may translate into altered *DNMT1* expression and function. Upon completion of prioritization of all the possible SNVs of *DNMT1*, we found 17 non-syn-SNVs, 82 syn-SNVs, 65 intronic SNVs and 31 intergenic SNVs to be most potent genetic variants for checking their association(s) and functional role(s), (if any), using follow-up association and functional experiments, respectively. As already mentioned that after STRING v11.0 analyses, we found HIST1H3H, EIF3G, MRPL4 and EEF2 to reside within same interactome with DNMT1. It became necessary for us to check if we could find any probable role of any of the 4 interacting proteins of DNMT1 in terms of their involvement in any neurological altered manifestation. Interestingly, after extensive literature search, we found that variations in *HIST1H3H* are well known for their roles in pediatric non-brain stem glioblastoma [28]. On one hand, EIF3G is well known for its ubiquitous roles in eukaryotic translation initiation and on the other hand *MRPL4* codes an integral component of mitochondrial large ribosomal subunit. Recently, it has been seen that Mrpl4 plays important role for development of hypertension and stroke in rats [29]. Mice homolog of EEF2 is already known for its role in generation of long-term depression [30]. It is important to mention here that several studies opined association of defective function of DNMT gene with various complex disorders like hereditary sensory and autonomic neuropathy type 1 (HSAN1), dementia [31], schizophrenia [32] and cancer [1]. All the 17 prioritized non-syn SNVs of *DNMT1*, were checked in DeepView - Swiss-PdbViewer for alteration in protein structure and interestingly, 6 of them were found to modulate different structural conformational changes in protein (electrostatic potential and steric clashes), which could result into altered function of DNMT1 enzyme.

After successfully prioritizing potential functional SNVs of *DNMT1* using *in silico* tools, it became necessary for us to check the expression pattern of DNMT1 in NTD model and in respective control. From our qRT-PCR based result, it is evident that lower DNMT1 gene expression is prevalent in NTD affected babies when compared to their respective mothers, which is in contrast with the fact that levels of DNMT1 stay at comparative level in both normal embryo and adult blood (data procured from SCREEN interface of ENCODE, Supplementary Figure S3) [33]. Thus, our study portrayed a plausible relationship between occurrence of NTD and lowered DNMT1 level in the newborns which in turn could be result of anomalous expression of *DNMT1*-regulated (imprinted) genes like *IGF2*, *PEG3* and *LINE1* contributing to neuro-pathogenesis.

## Conclusion

We believe our case report would pave ways for future association and functional studies in quest of searching functional relationship between variations in DNMT1 expression and NTD manifestation. With the best of our knowledge, this is the first report from this part of the globe which could be helpful in better diagnosis of NTD and might also be helpful to evaluate the genetic consequences in ethnically distinct East Indian population sublimely.

## Supplementary Material

Supplementary Figures S1-S4 and Tables S1-S4

## Data Availability

The data that support the findings of this study are available from the corresponding author upon reasonable request. The data may be available upon request.

## Abbreviations

DNMT: *de novo* methyltransferase
LD: linkage disequilibrium
NTD: neural tube defect
SAM: S-adenosyl methionine
SNV: single nucleotide variant
