# Supplementary material for: Analysis of DNMT1 gene variants in progression of neural tube defects—an in silico to in vitro approach
Source: Biosci Rep. 2022 Dec 6;42(12):BSR20220998. doi: 10.1042/BSR20220998 (PMC9727204; doi:10.1042/BSR20220998)
Supplement: Supplementary Figures S1-S4 and Tables S1-S4 [file BSR-2022-0998_supp.pdf]

Analysis of DNMT1 Gene Variants in Progression of Neural Tube Defects-an *insilico* to *invitro* approach

Susanta Sadhukhan[1†], Nirvika Paul[1†], Sudakshina Ghosh[2], Dinesh Munian[3], Kausik Ganguly[4], Krishnendu Ghosh[1], Mainak Sengupta[4], Madhusudan Das[1,*]

[1]Department of Zoology, University of Calcutta, 35 Ballygunge Circular Road,Kolkata-700019,India[2]
Department of Zoology, Vidyasagar College for Women, 39 Sankar Ghosh Lane, Kolkata-700006, India[3]
Department of Neonatology, Institute of Postgraduate Medical Education & Research, 244 AcharyaJagadishChandraBose Road,Kolkata,700020,India
[4]Department of Genetics, University of Calcutta, 35 Ballygunge Circular Road, Kolkata-700019,India
[*]Corresponding author at the Dept. of Zoology, University of Calcutta: madhuzoo@yahoo.com /mdzoo@caluniv.ac.in (mail id)
†Authors contributed equally

Supplementary Figure 1: Results of *DNMT1* containing 82 synonymous variants (syn SNVs) for which significant alterations were found in circular plots.

rs147713850

T

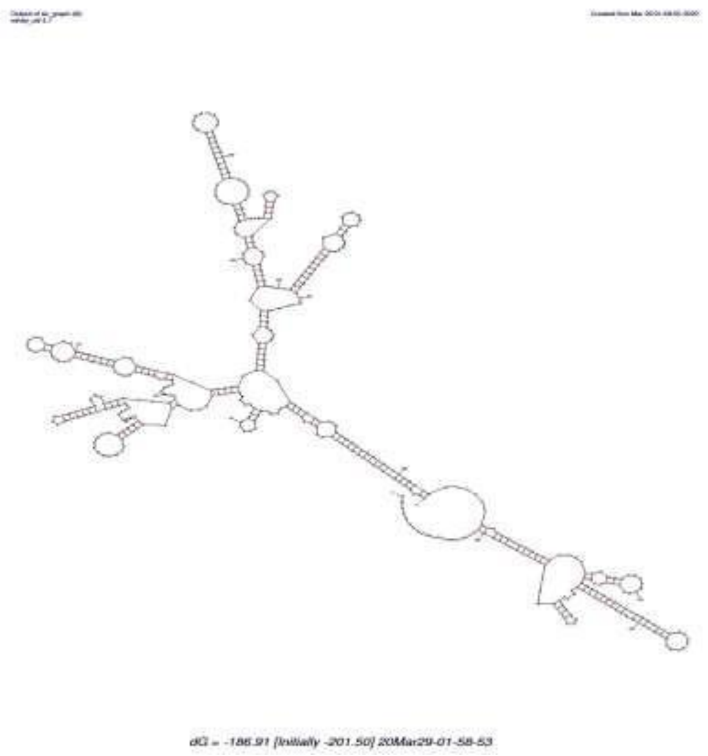

C

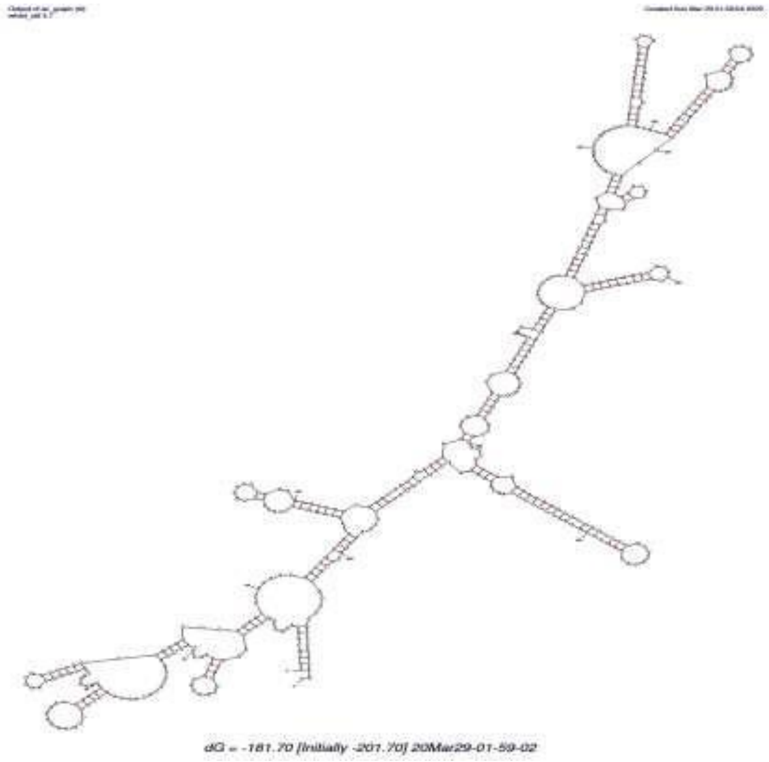

rs368325985

G

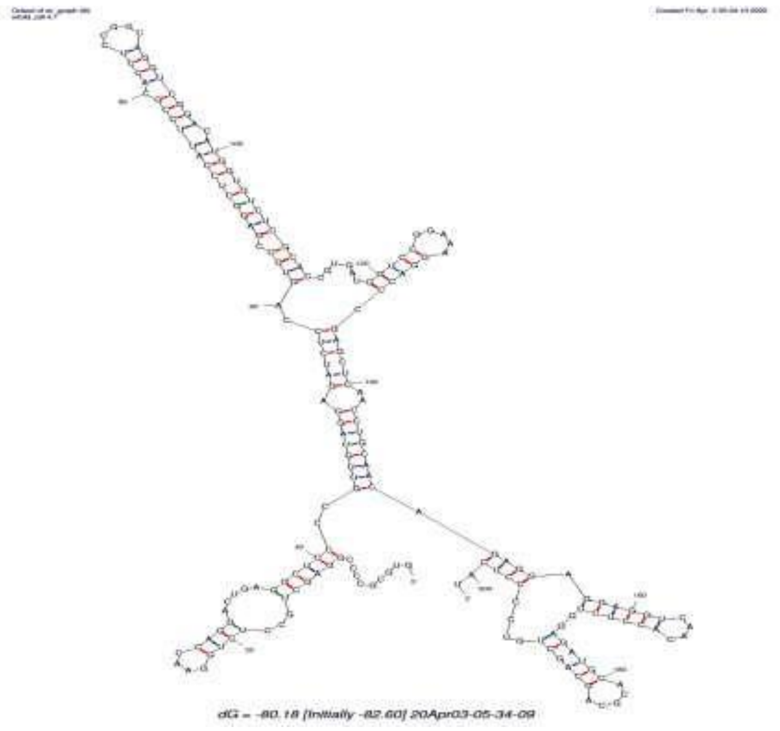

A

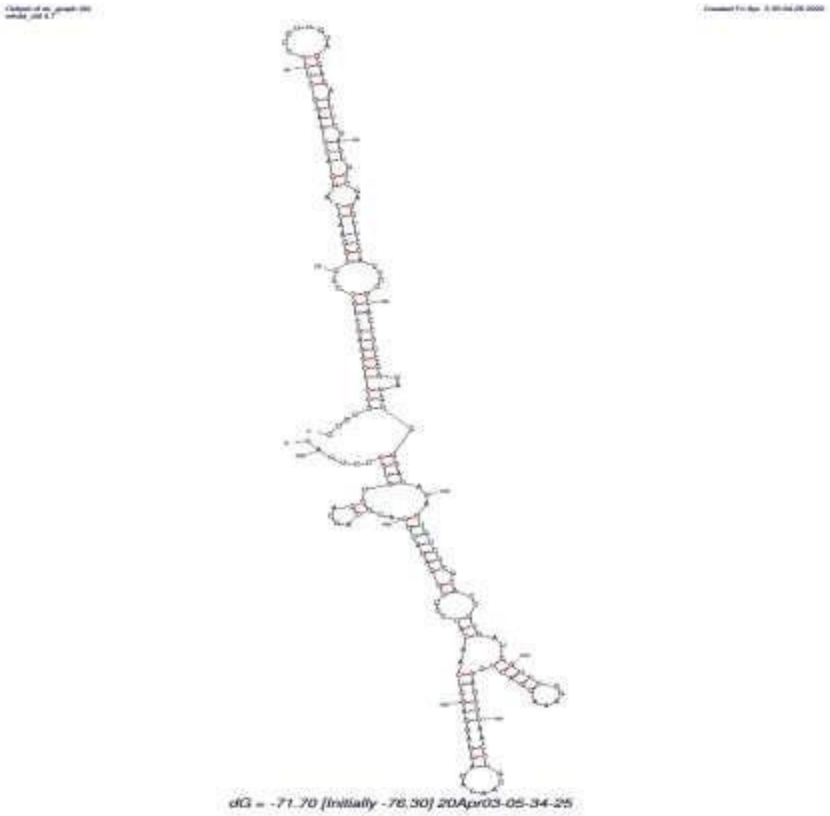

rs184125970

G

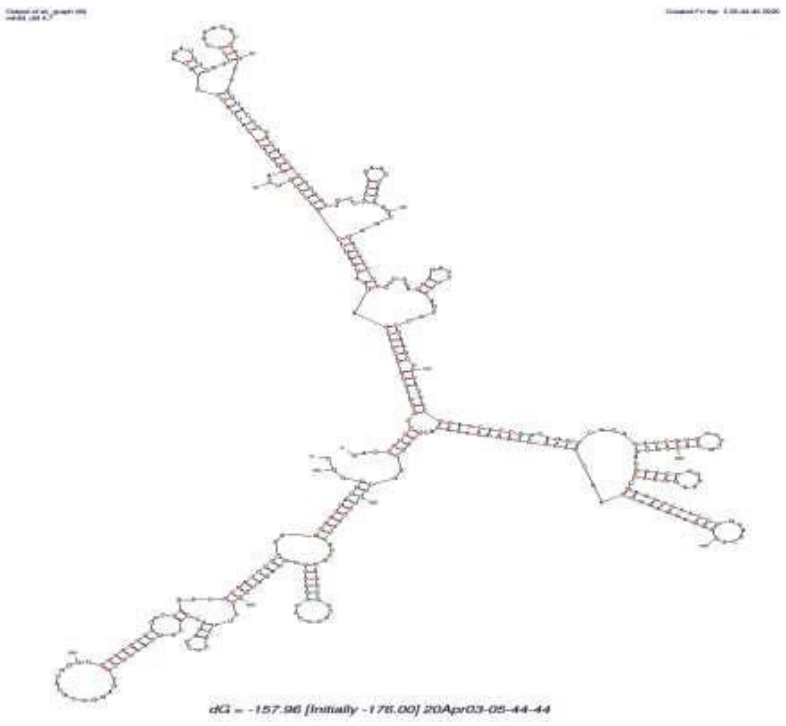

A

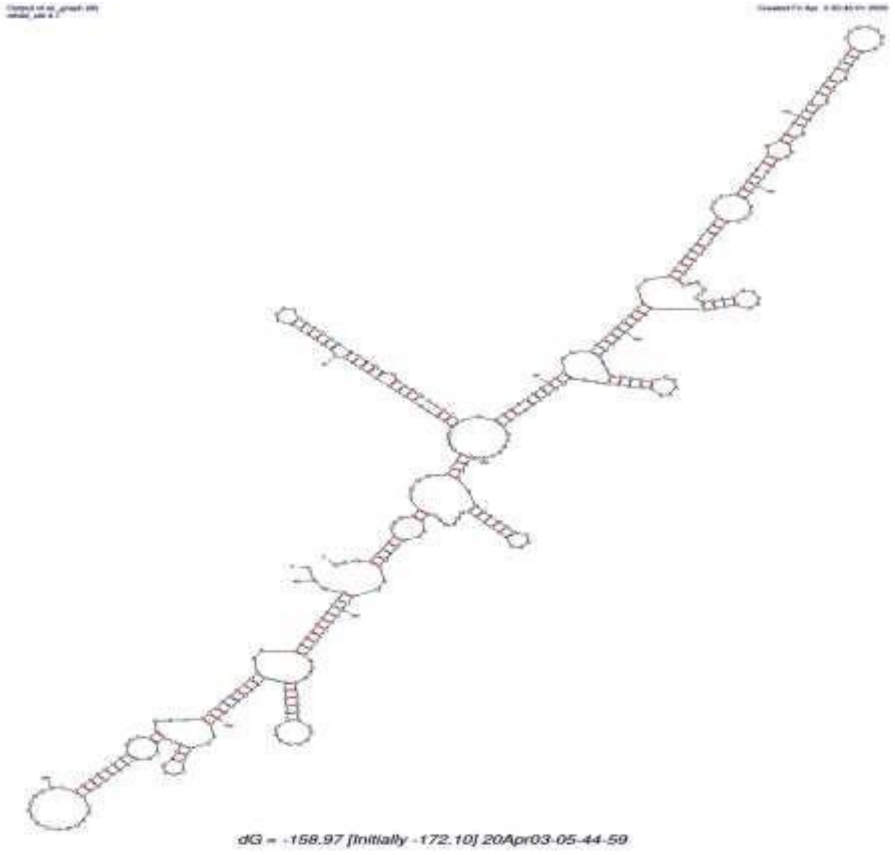

rs372988540

G

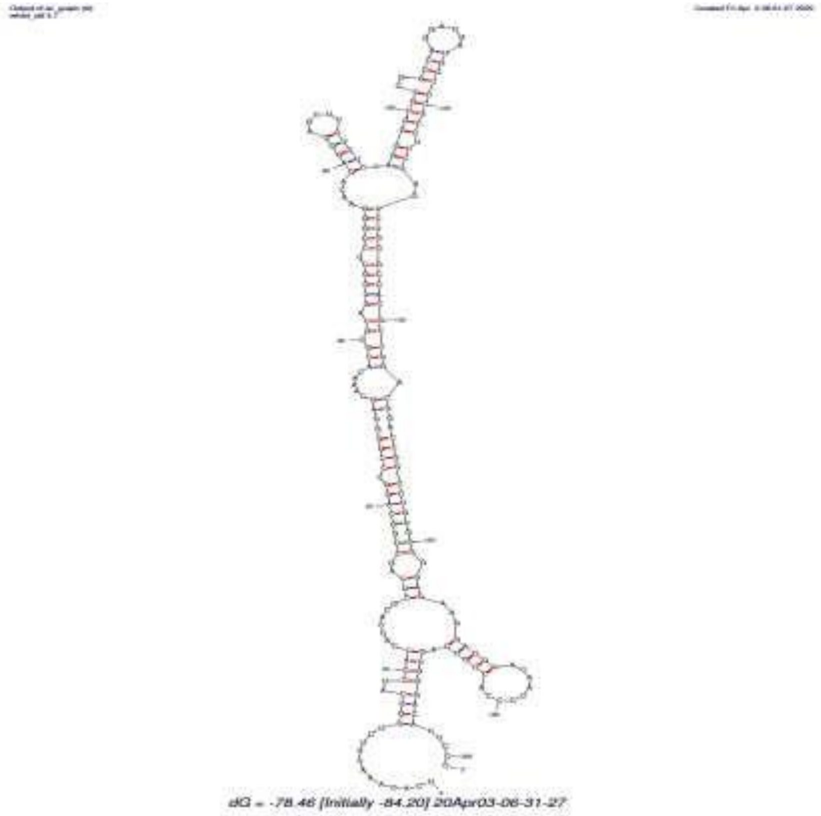

A

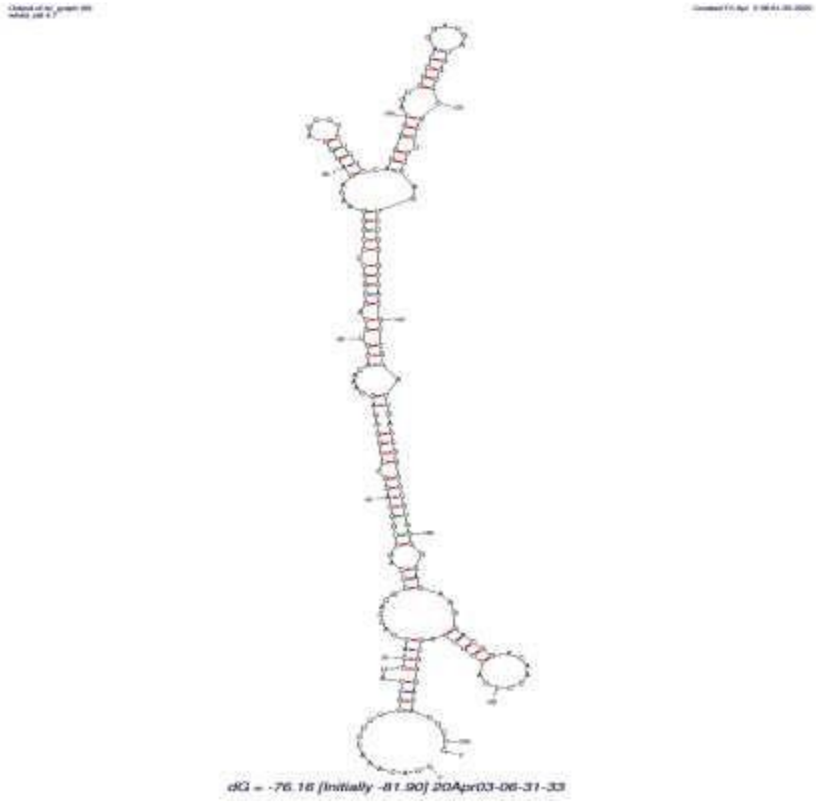

rs201007703

G

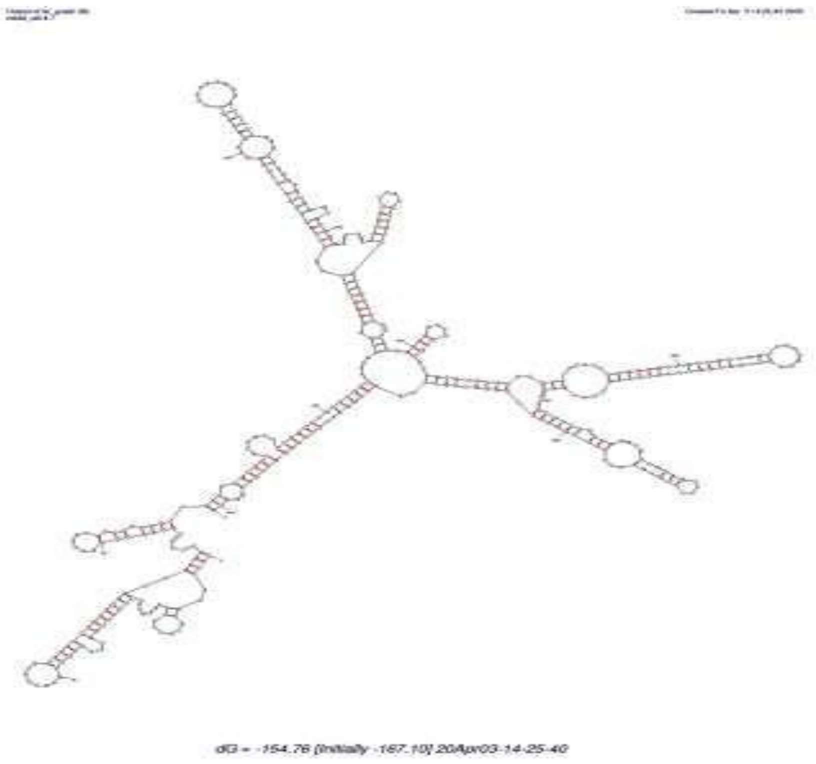

A

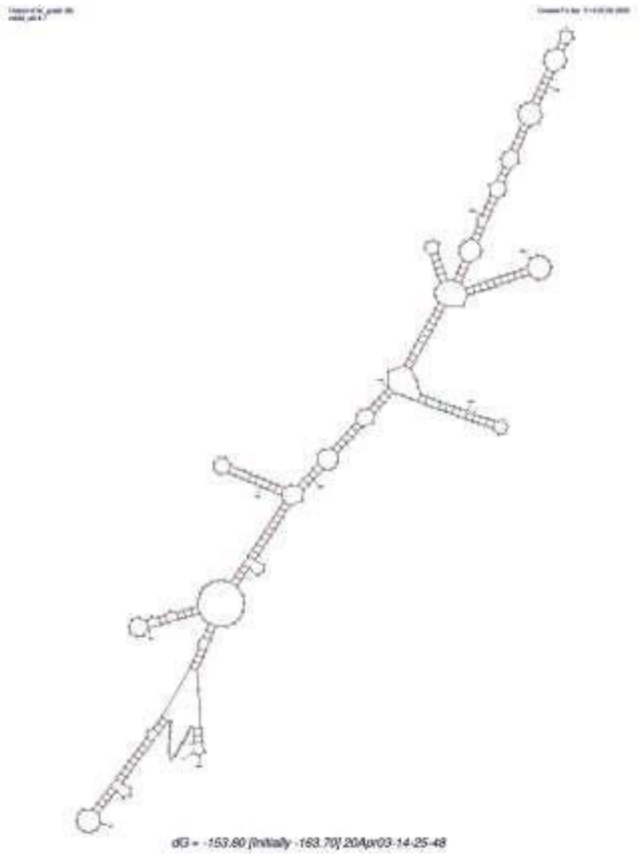

rs772752543

G

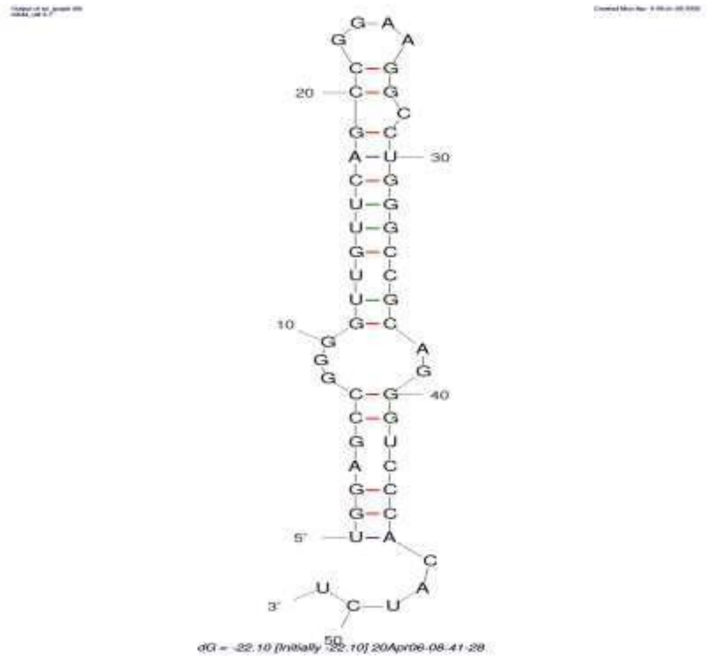

A

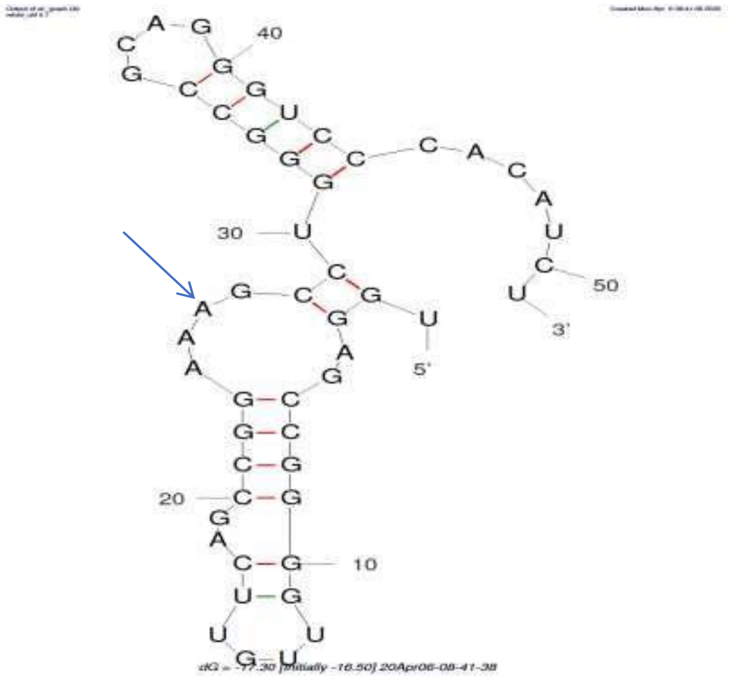

rs761140414

G

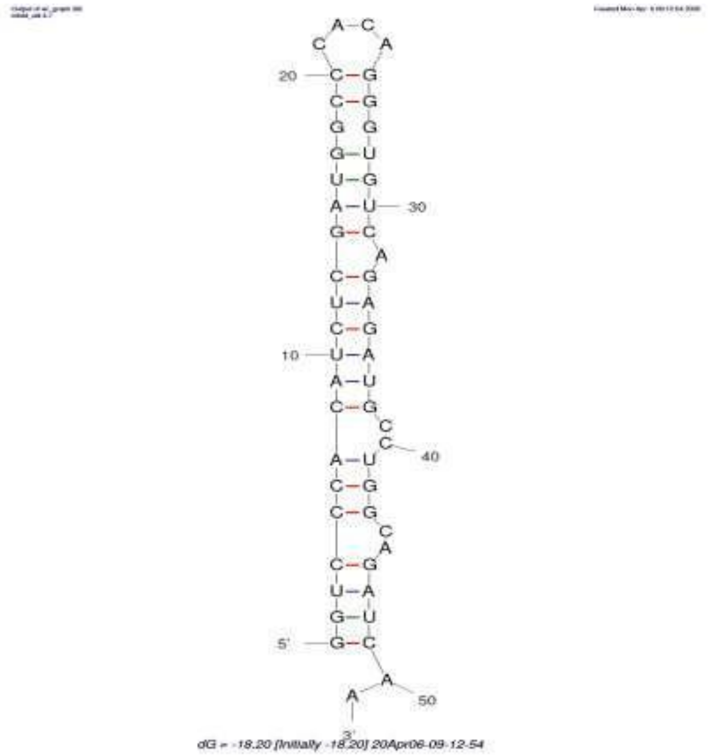

A

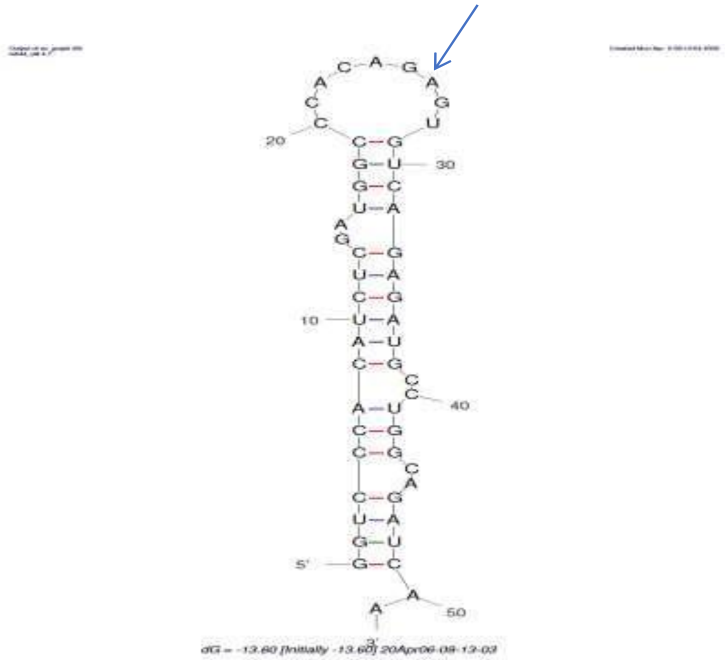

rs199739823

G

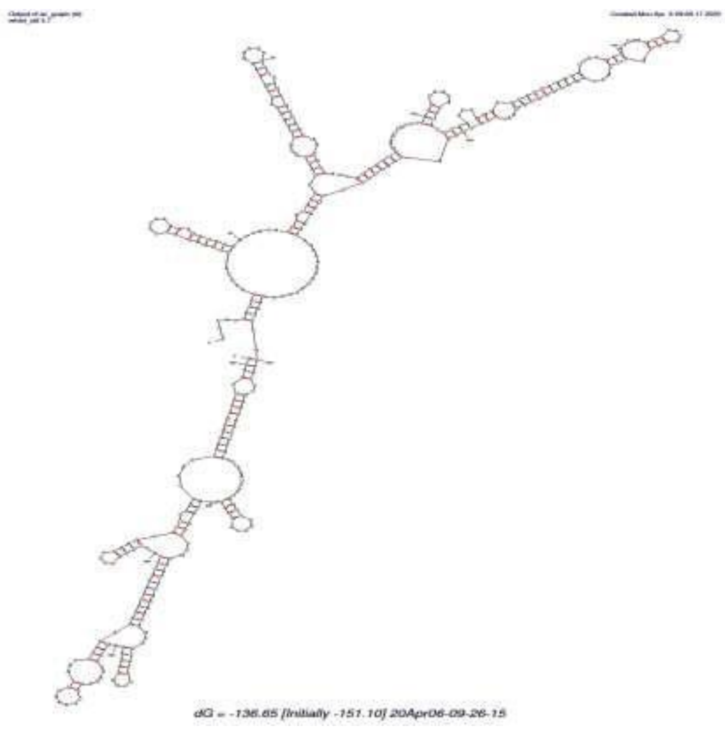

A

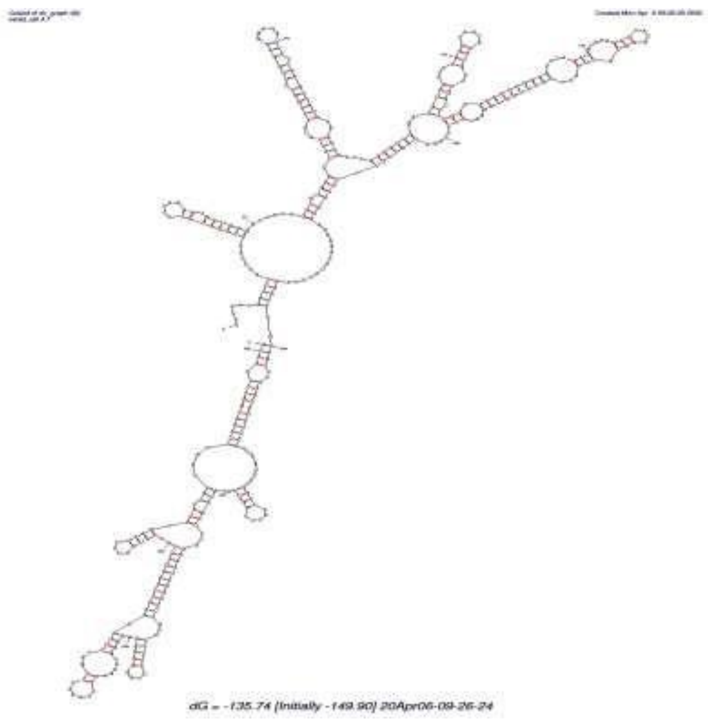

rs750931983

G

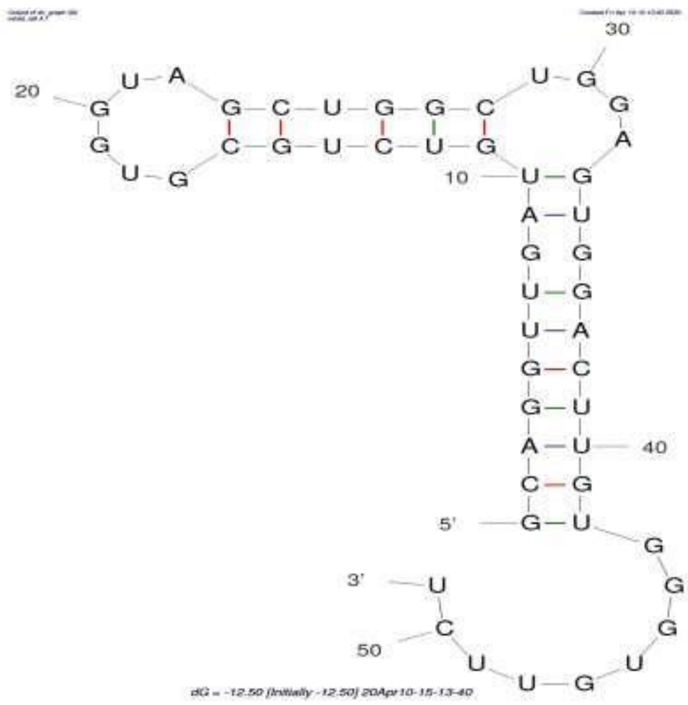

A

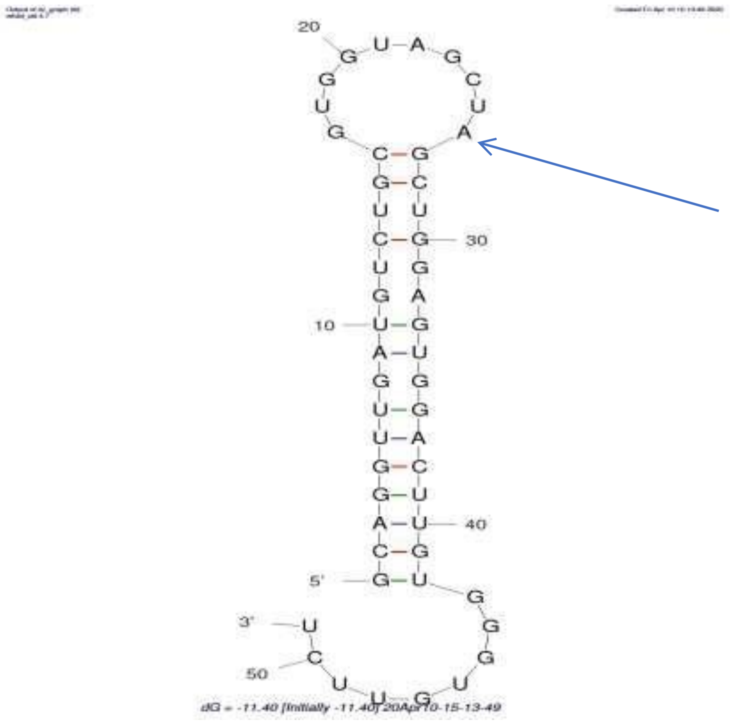

rs372694243

G

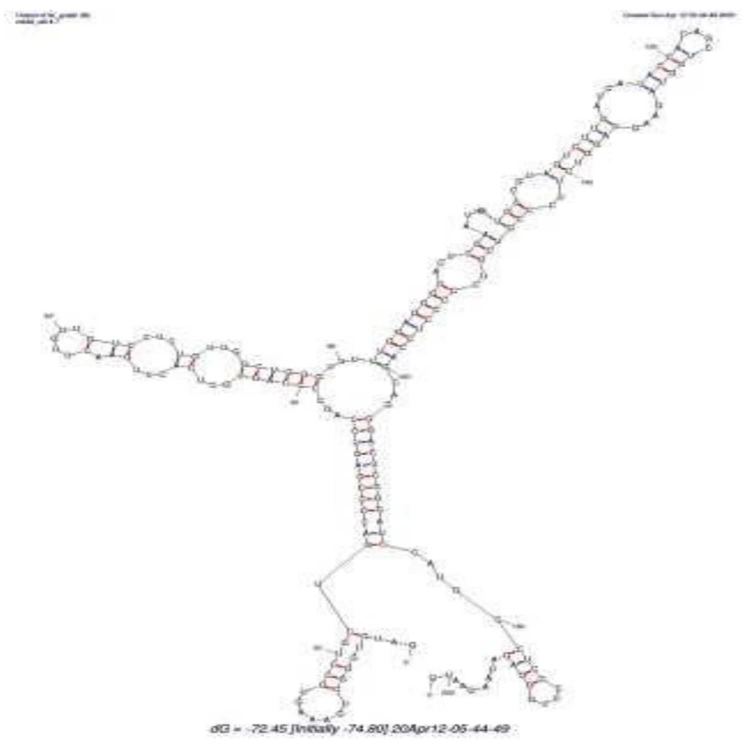

A

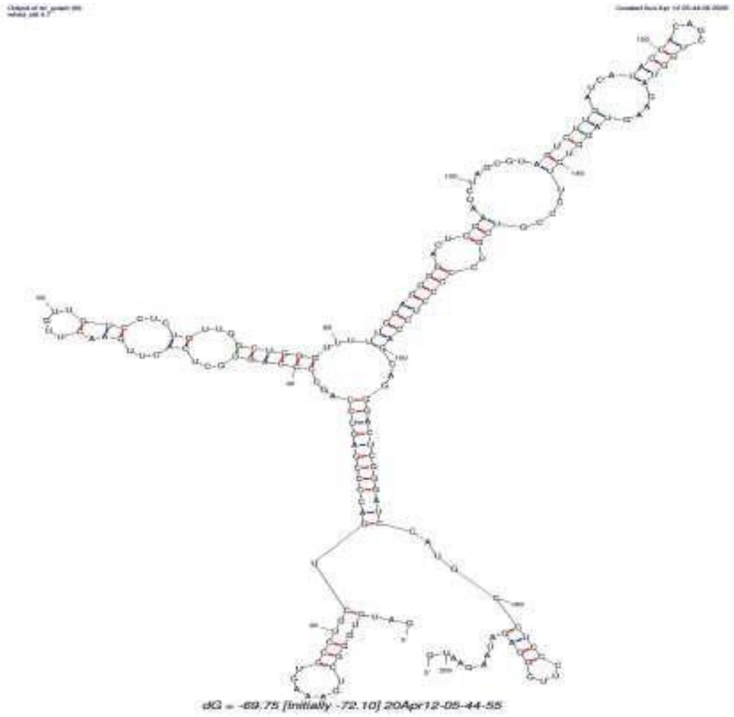

rs749216491

G

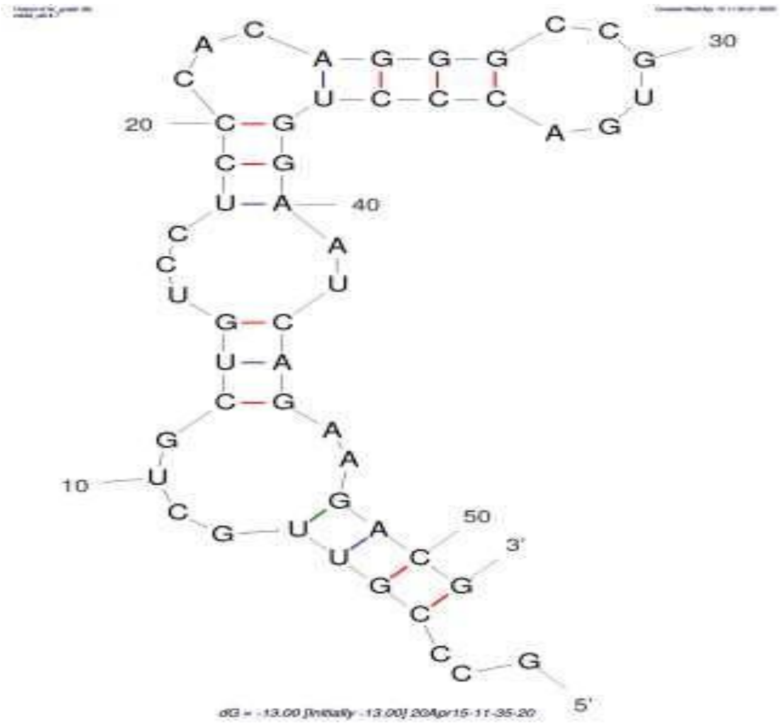

A

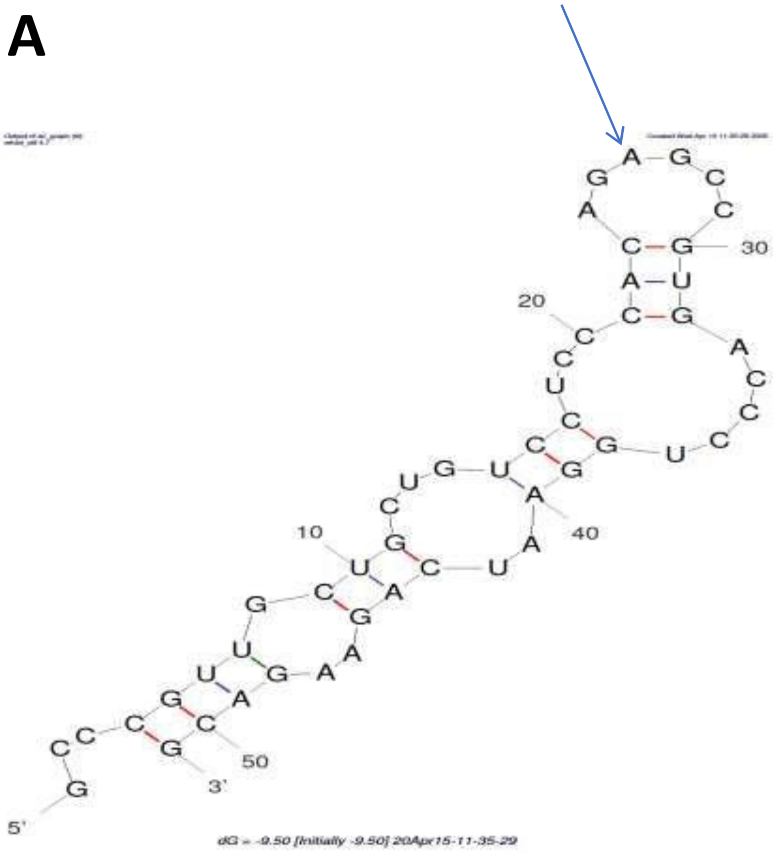

rs778997572

G

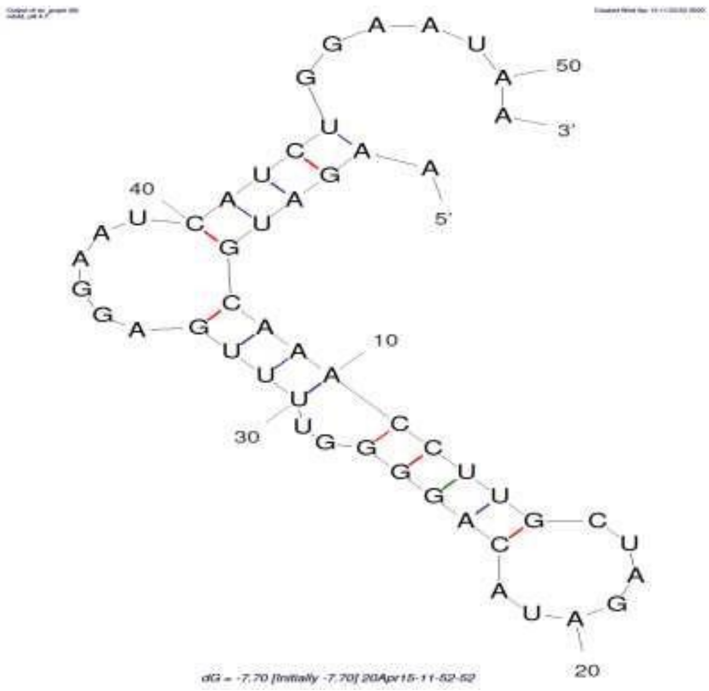

A

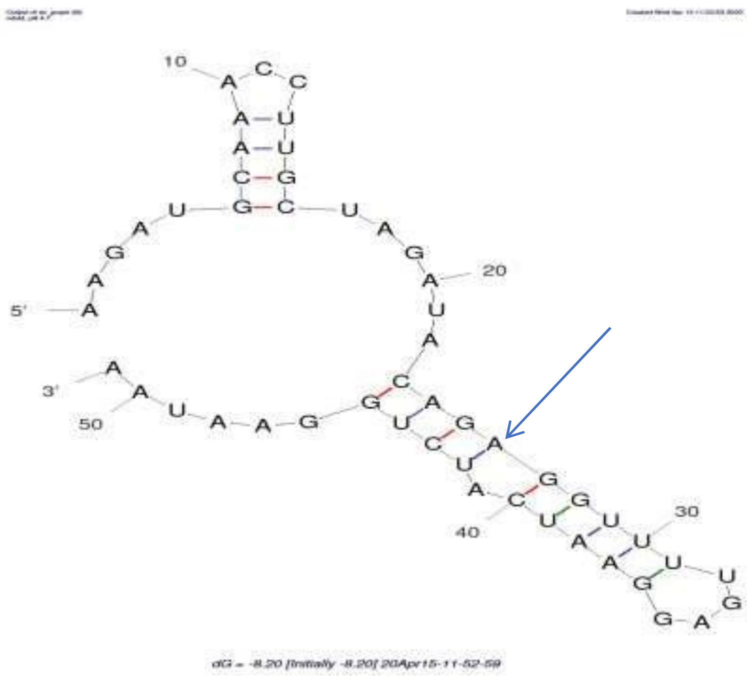

rs771365059

G

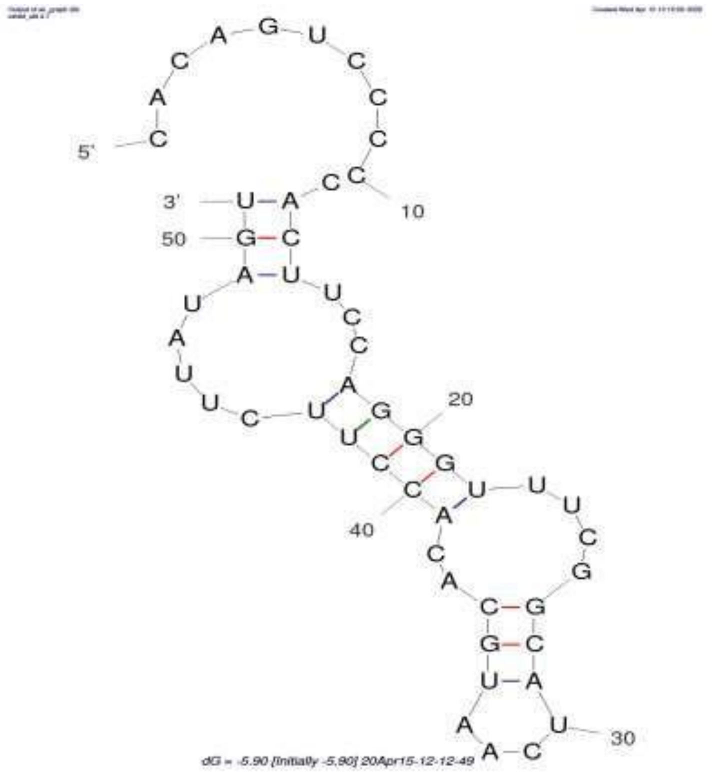

A

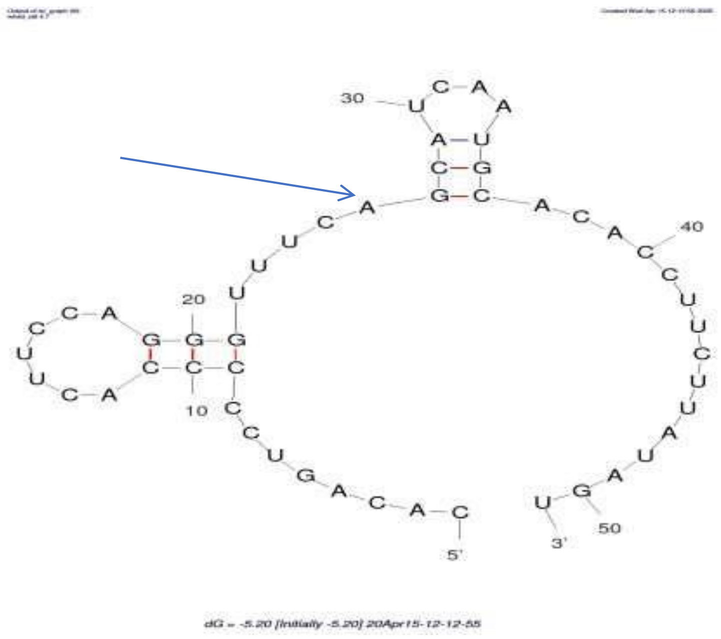

rs761852984

G

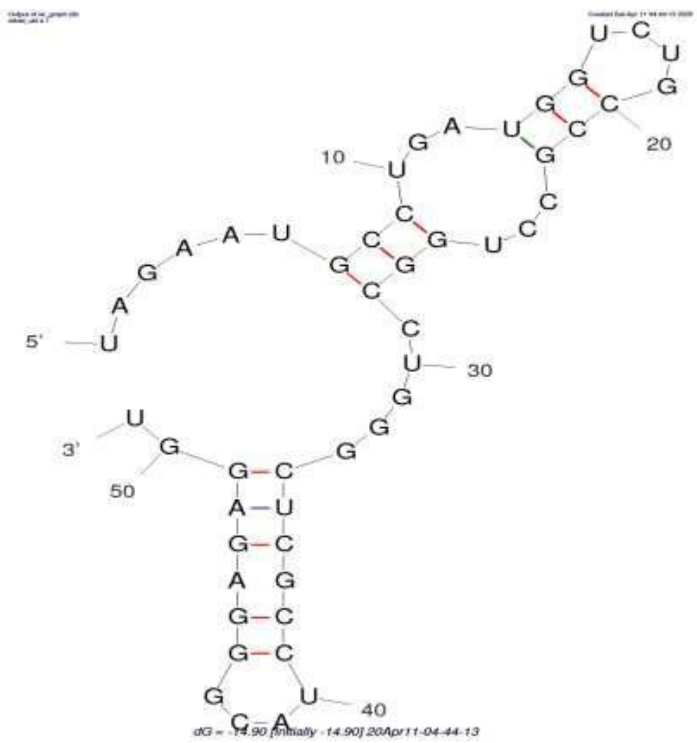

A

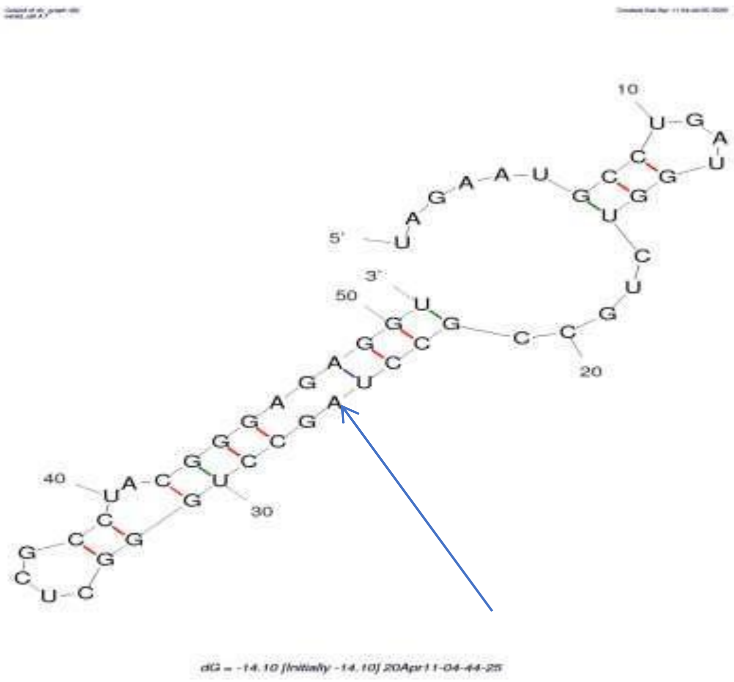

rs748007341

G

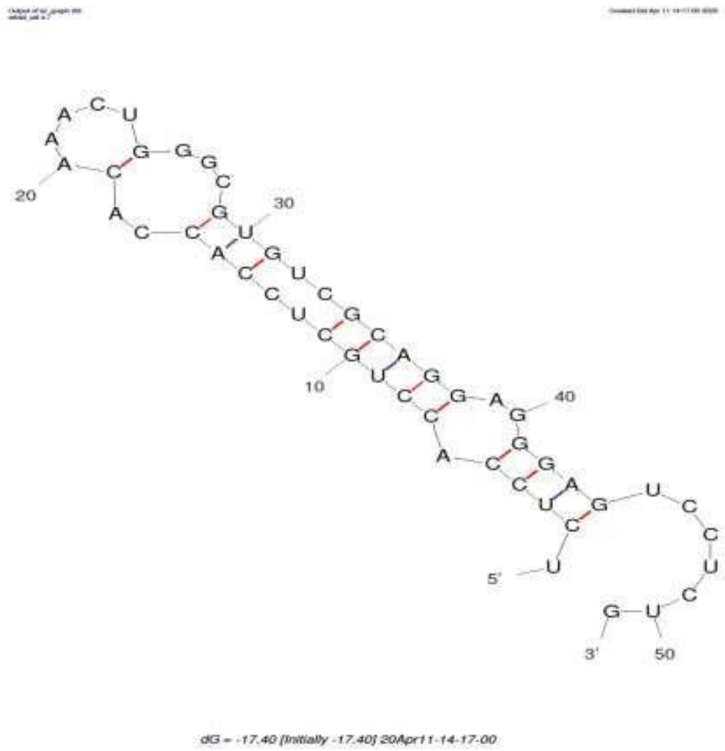

A

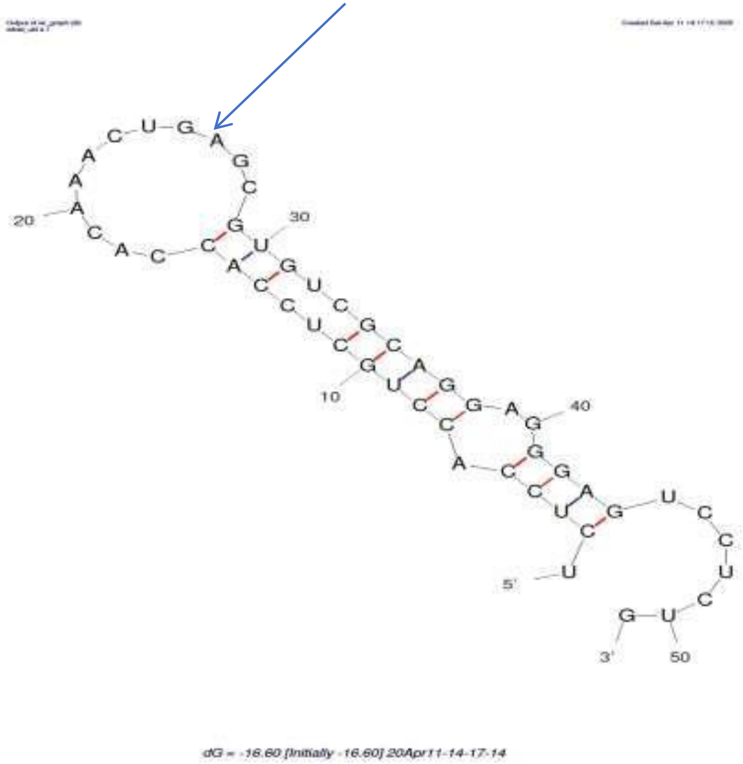

rs1459010920

G

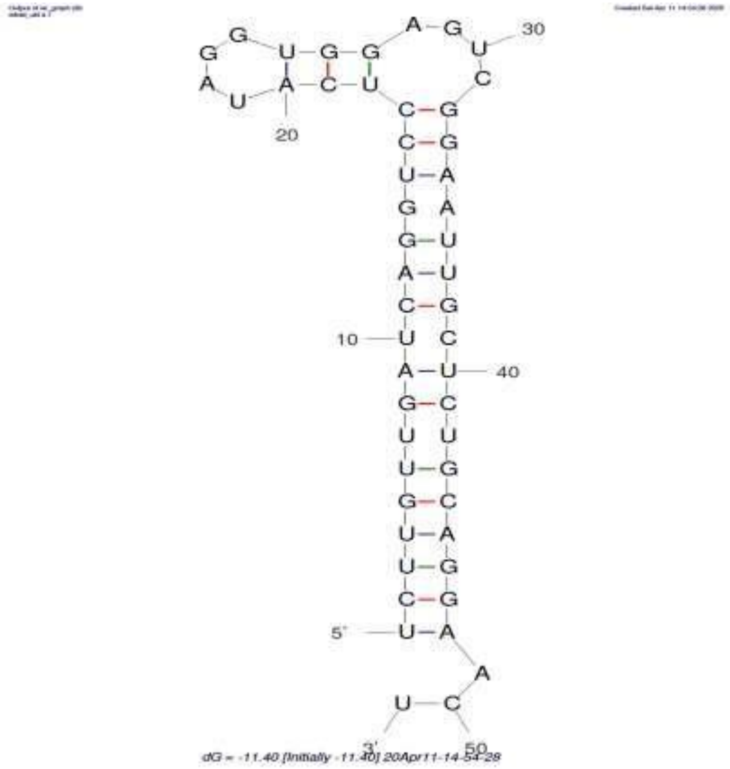

A

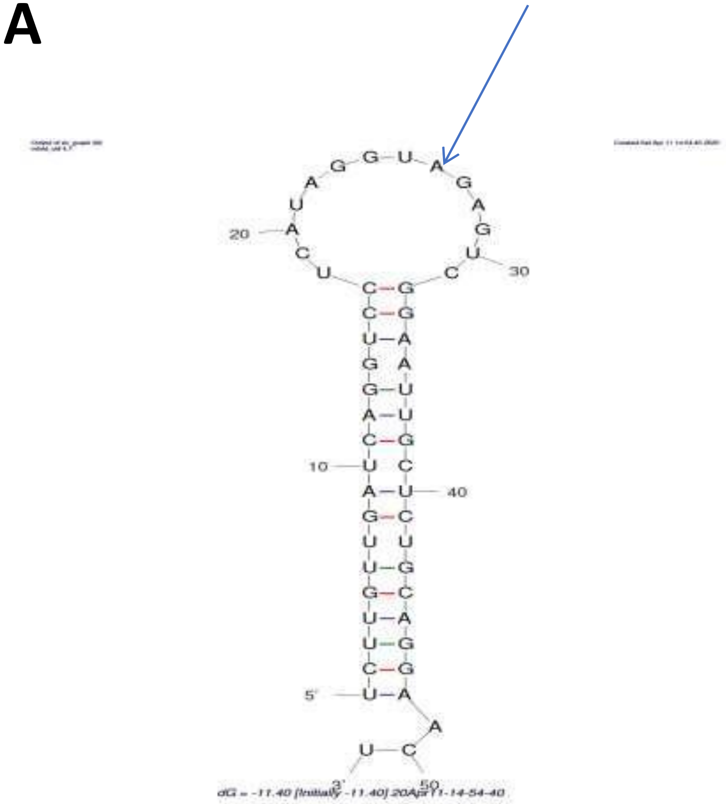

rs764039807

G

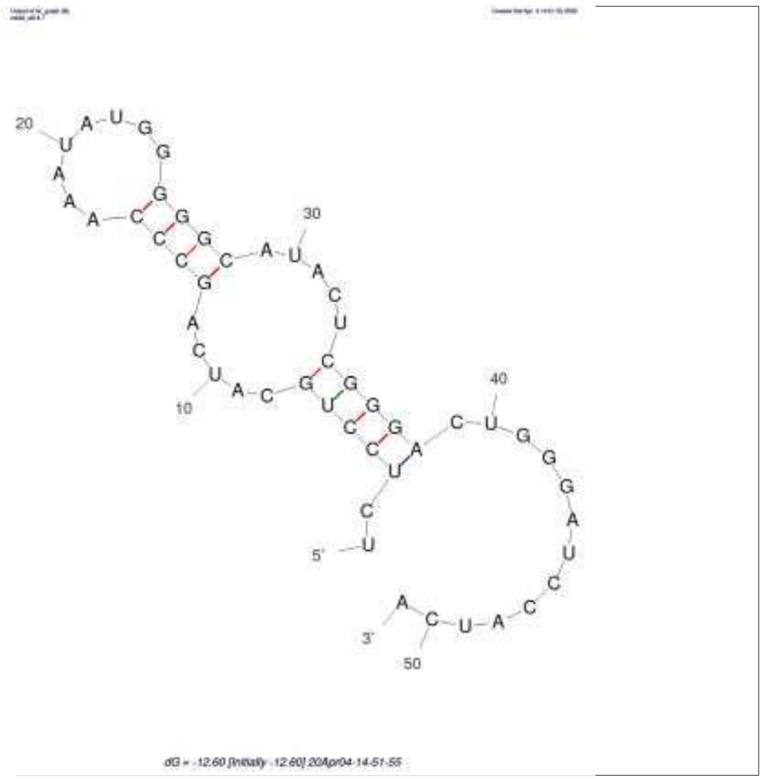

A

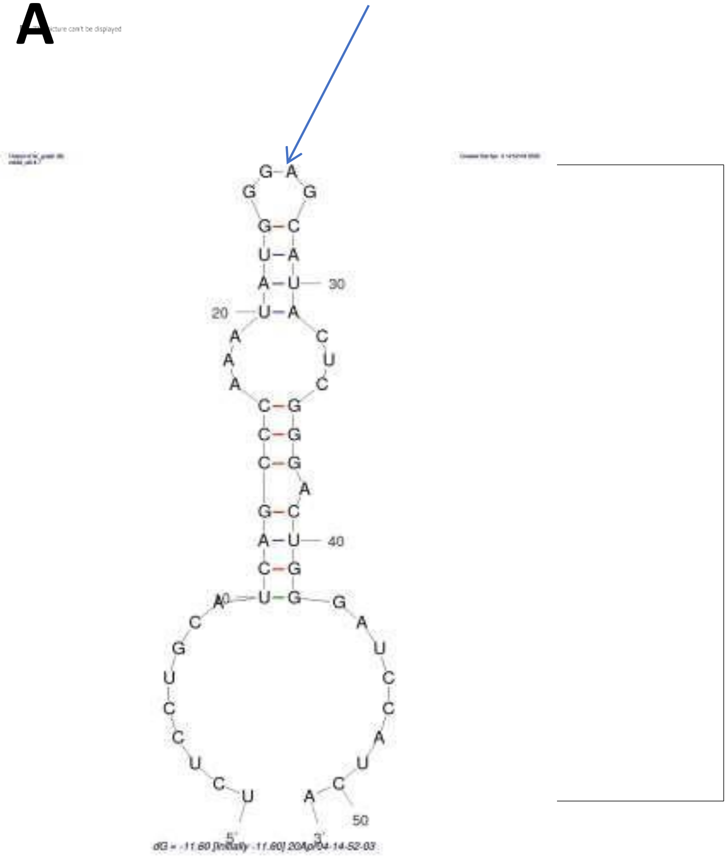

rs749045750

G

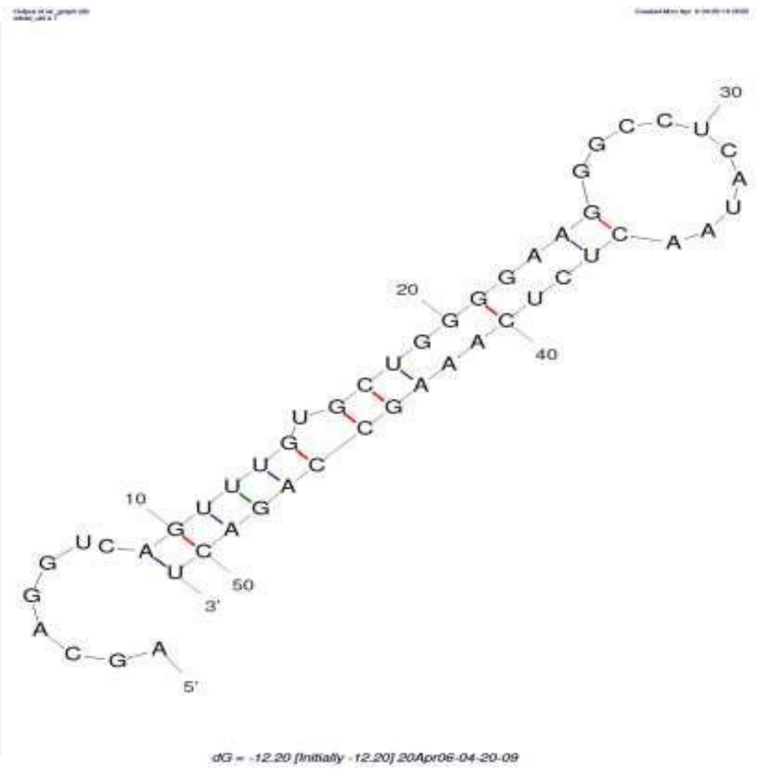

A

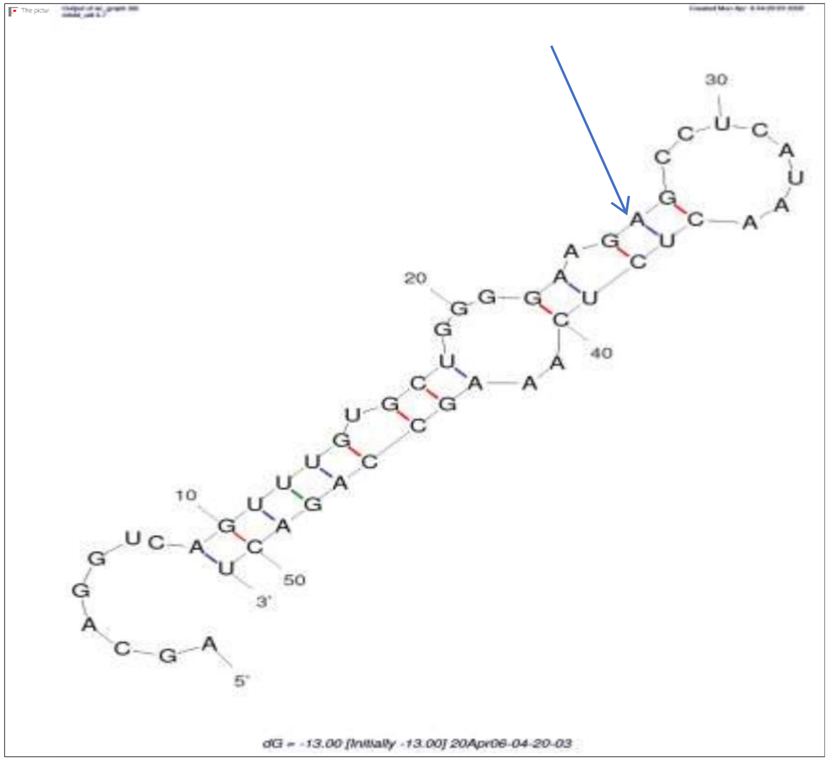

rs991716655

G

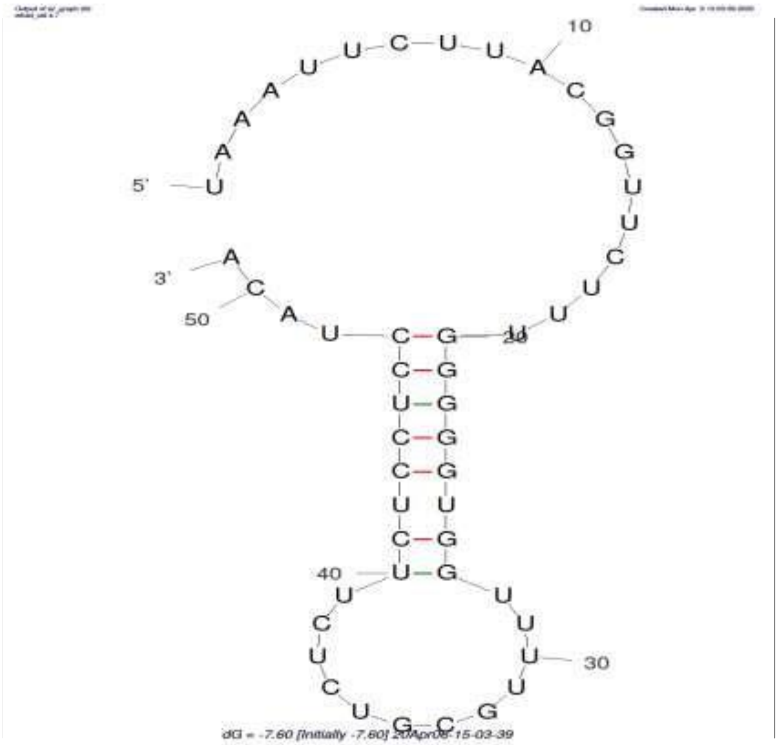

A

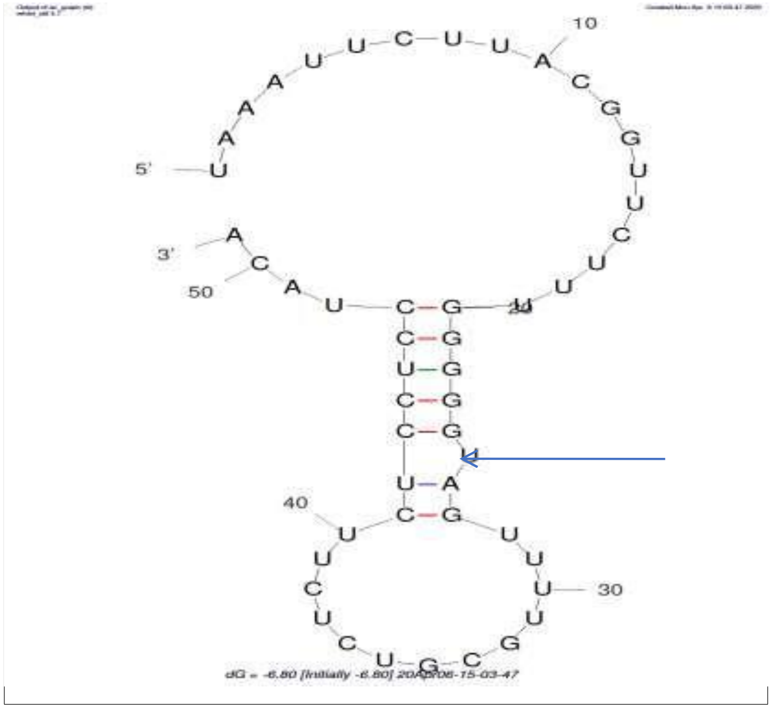

rs748449856

G

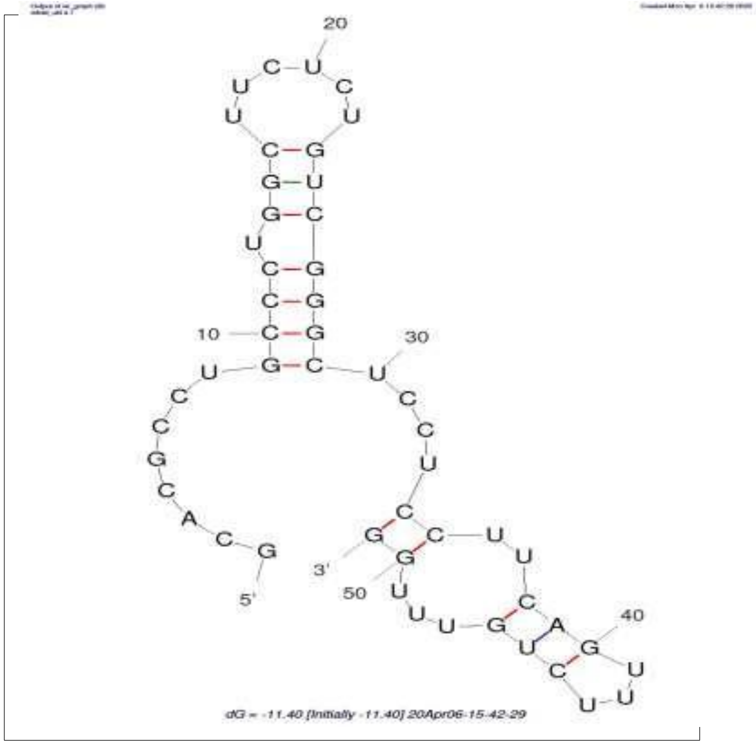

A

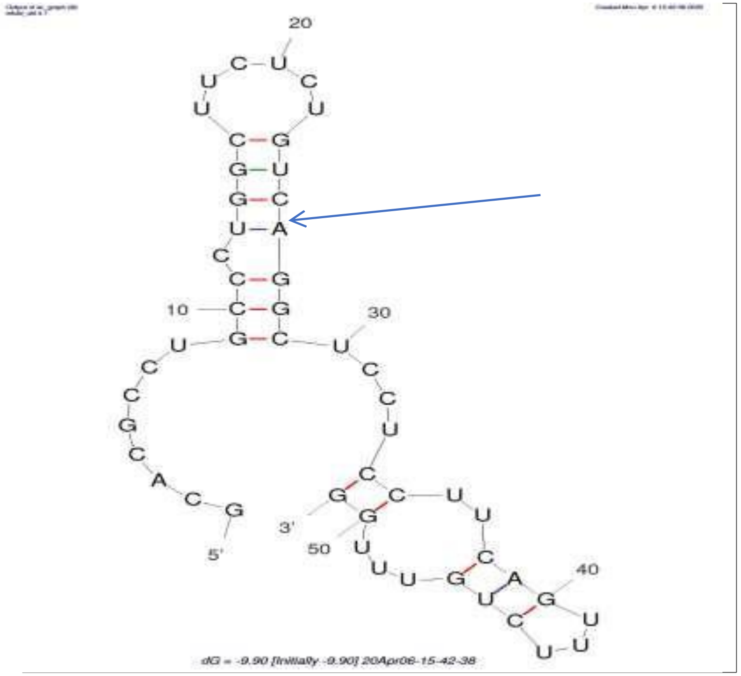

rs750234435

G

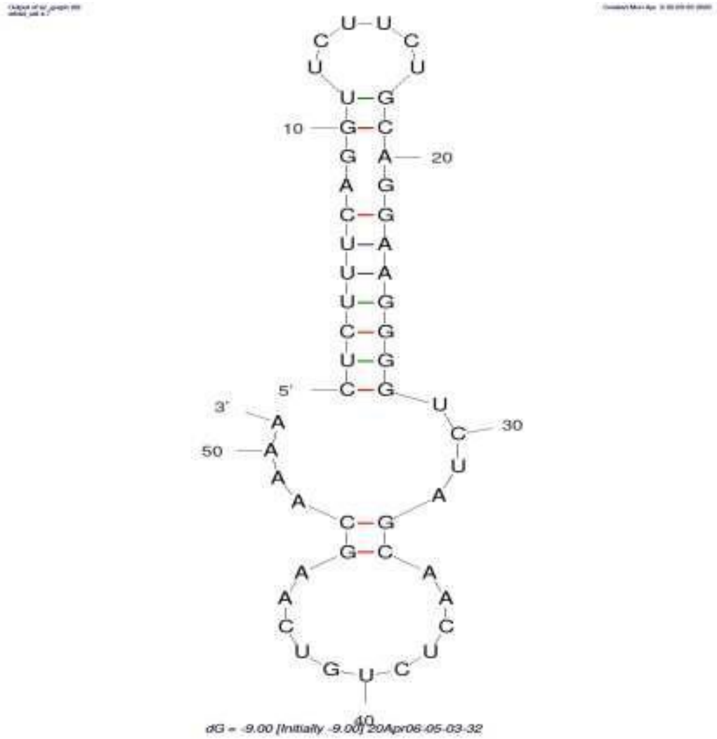

A

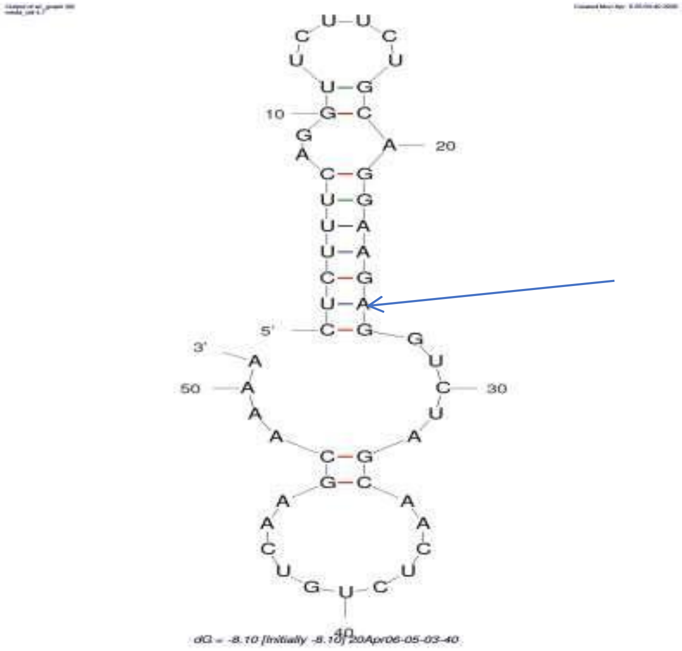

rs369311643

G

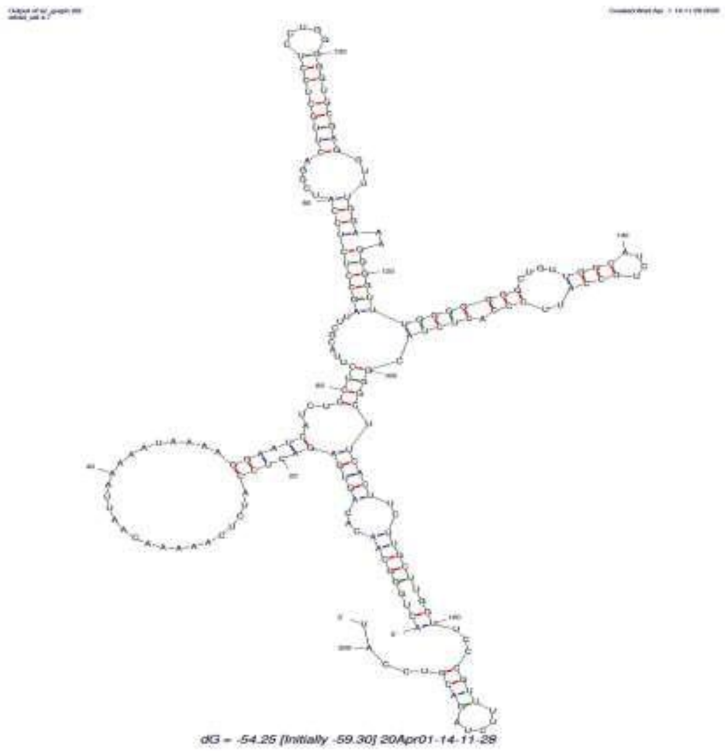

A

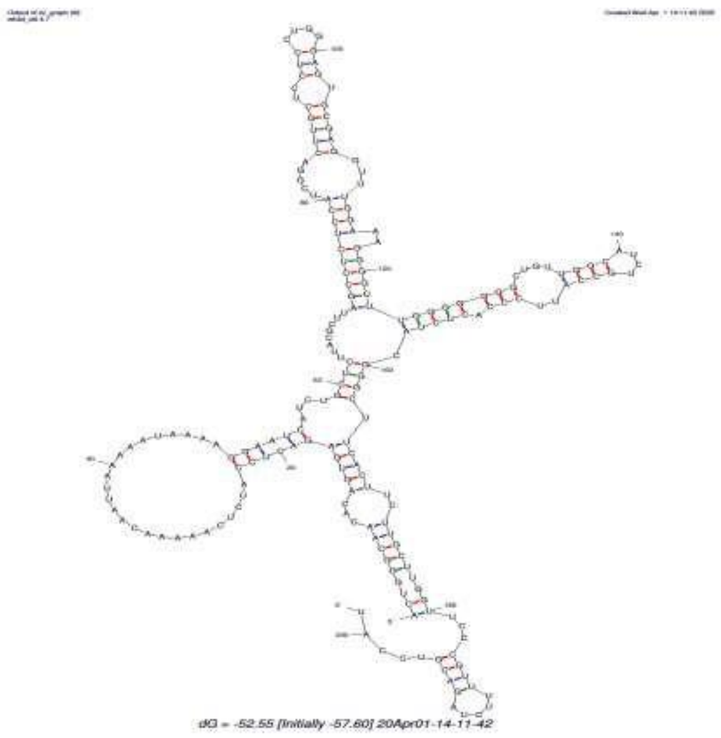

rs893895641

A

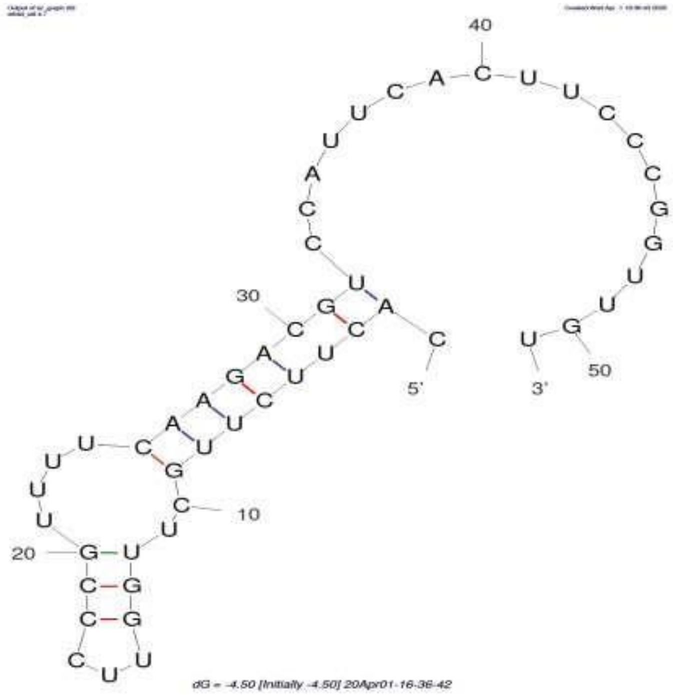

G

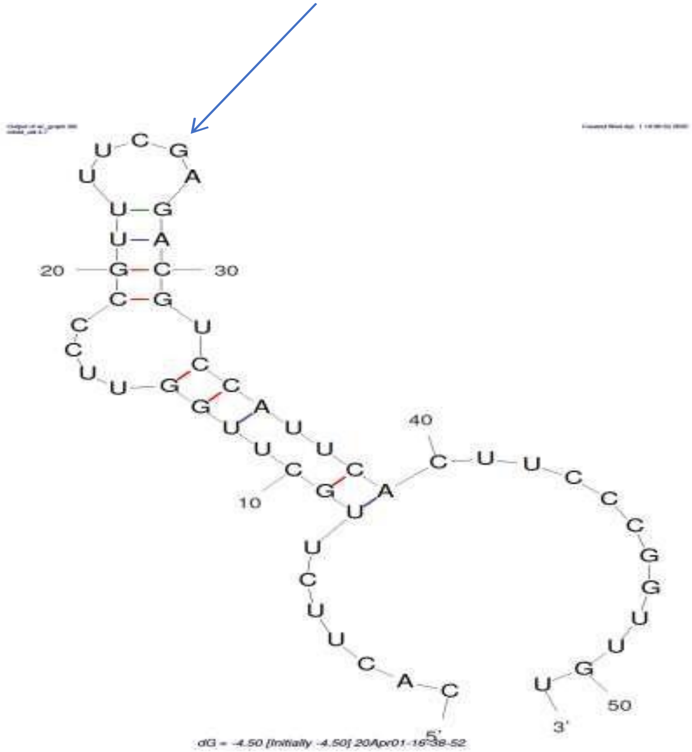

rs754986621

T

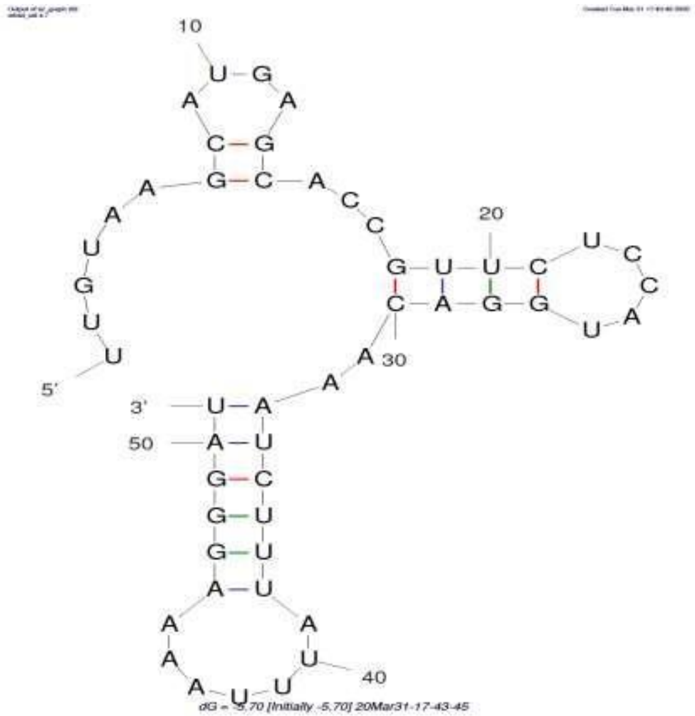

C

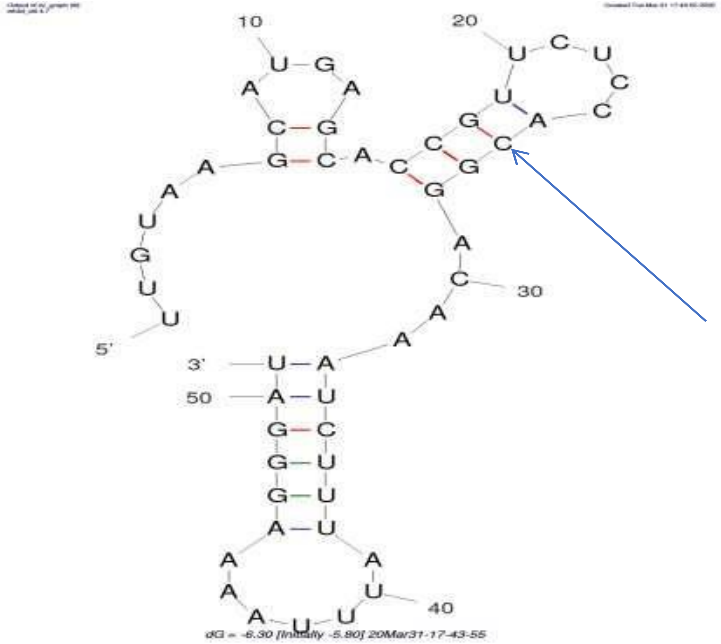

rs1456849796

A

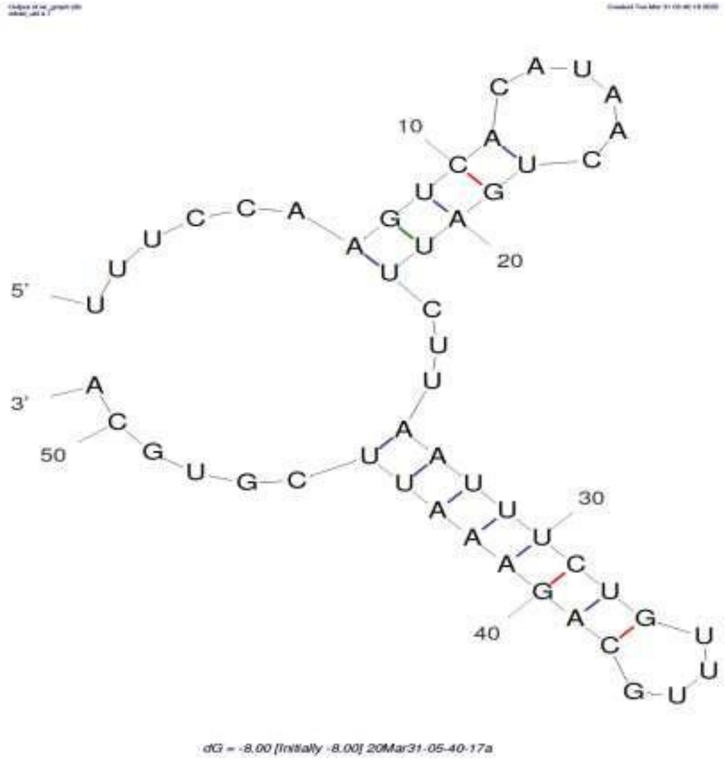

C

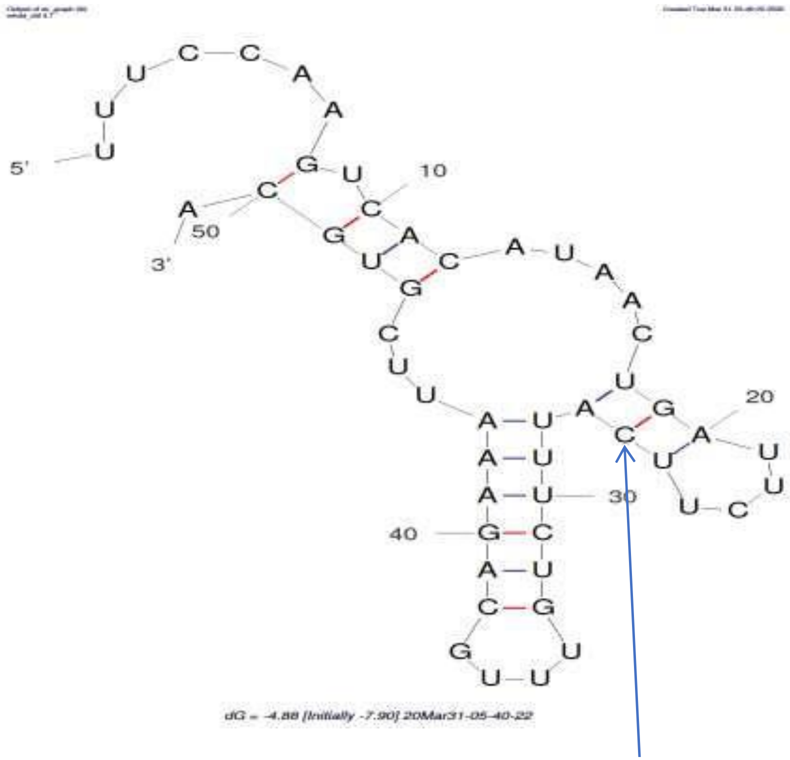

rs780874970

C

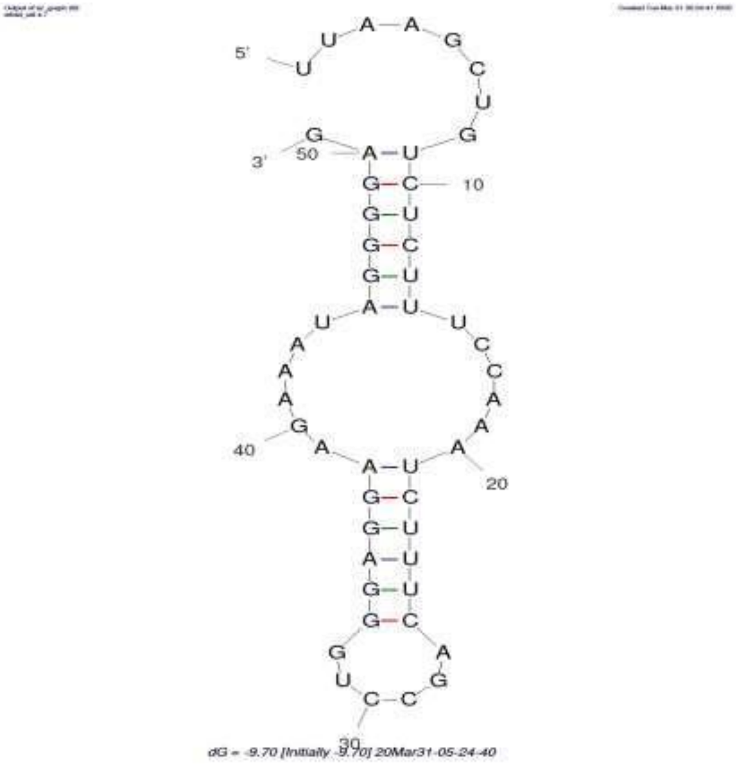

G

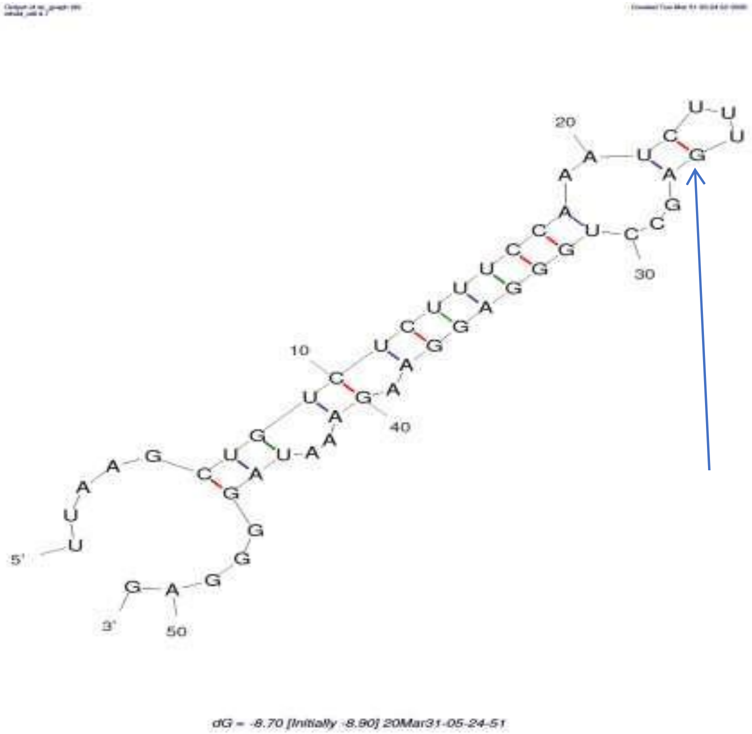

rs564679424

G

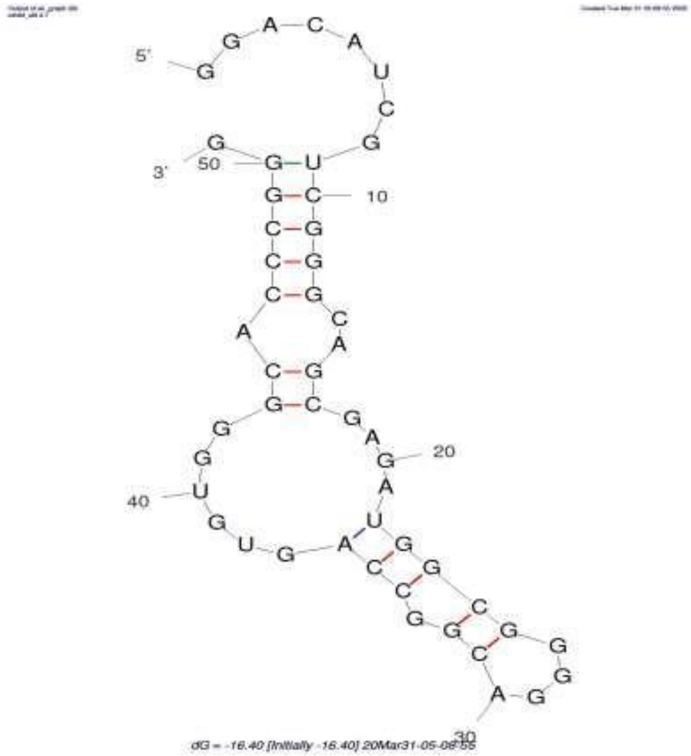

A

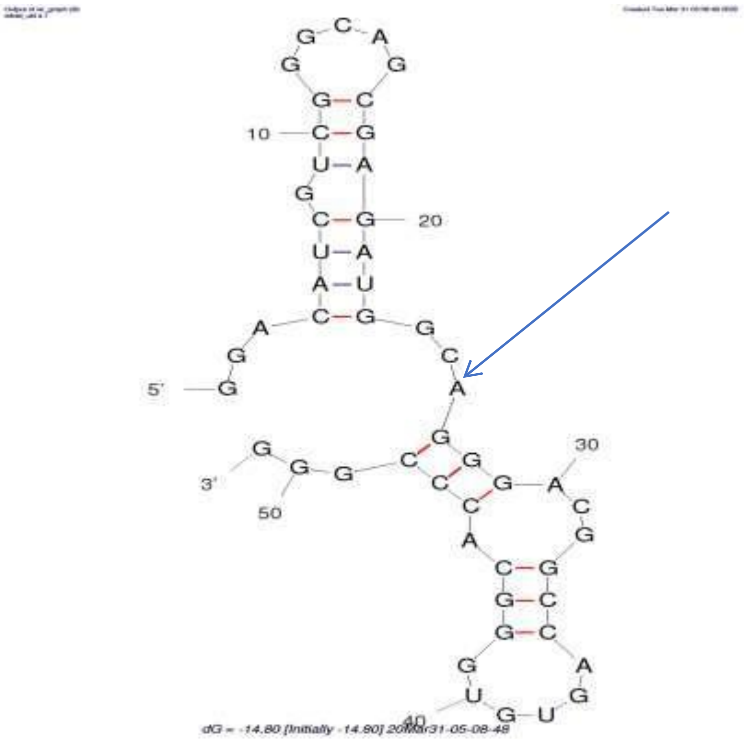

rs773637284

C

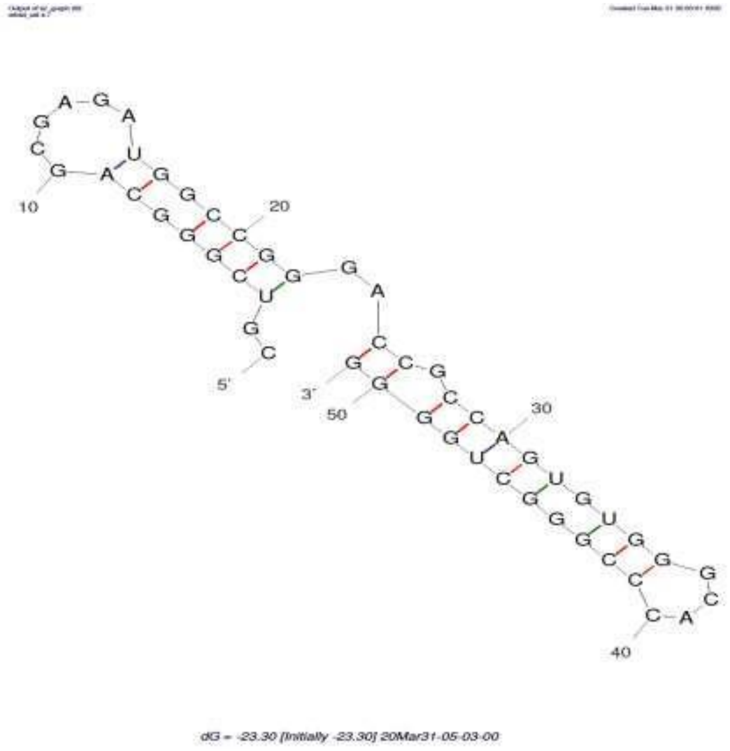

T

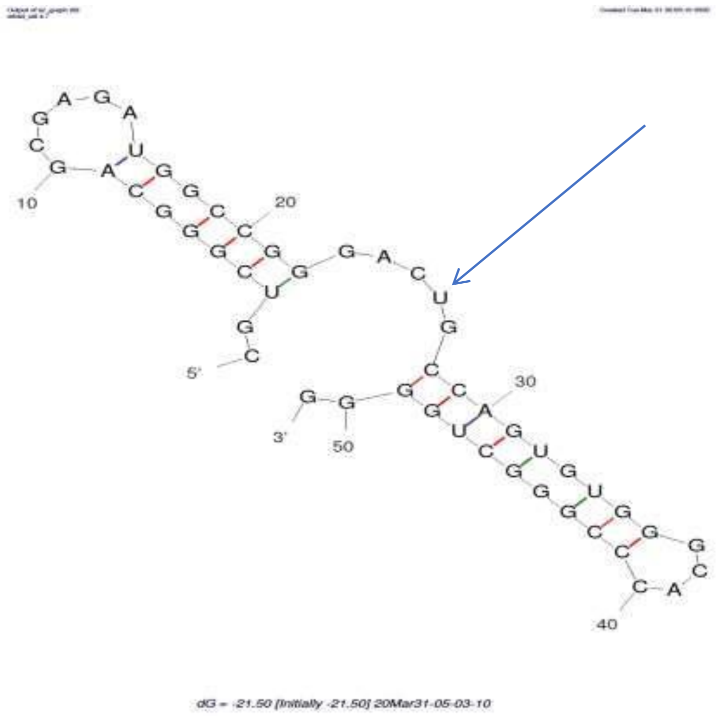

rs529120158

C

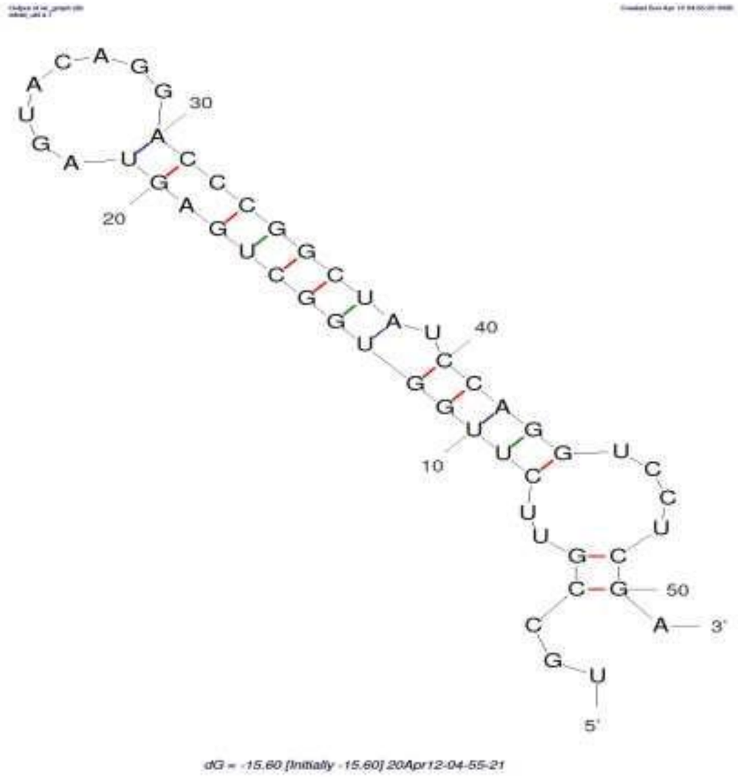

G

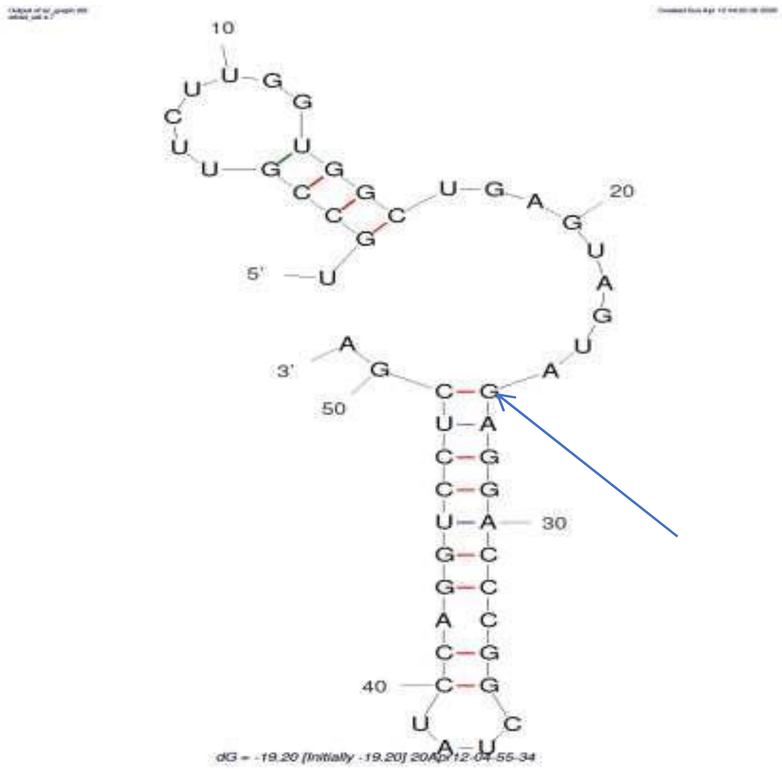

rs1452414039

G

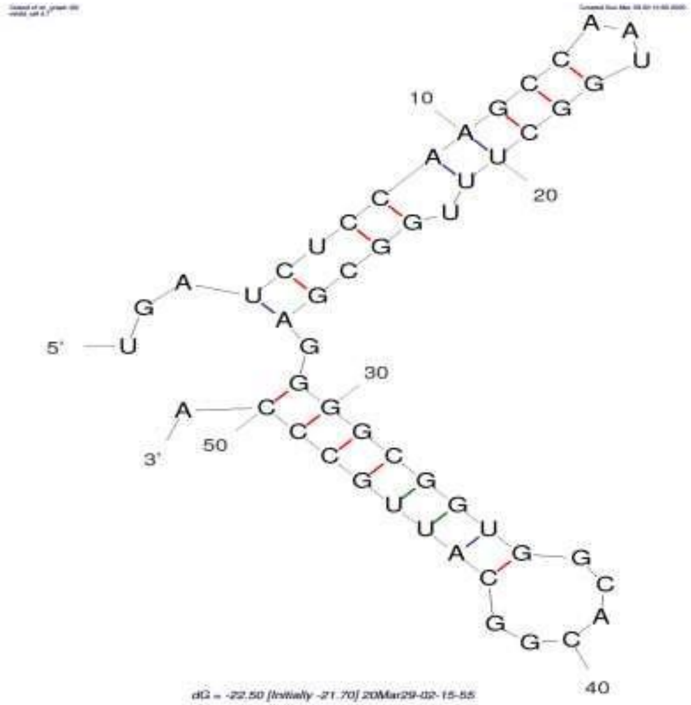

A

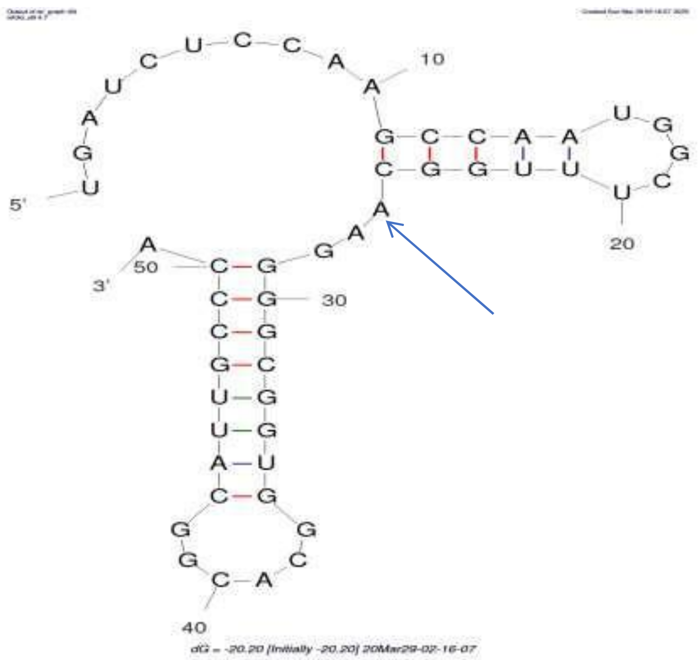

rs769962566

C

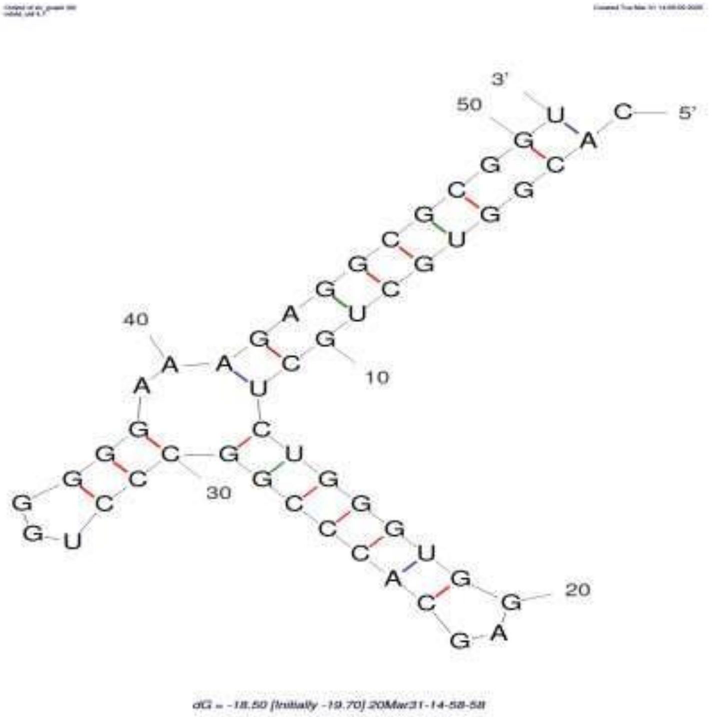

T

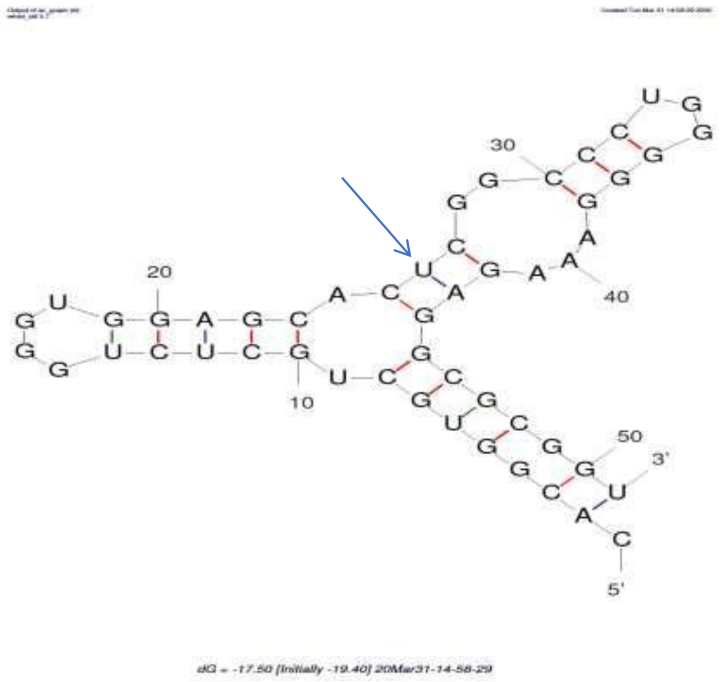

rs760037814

C

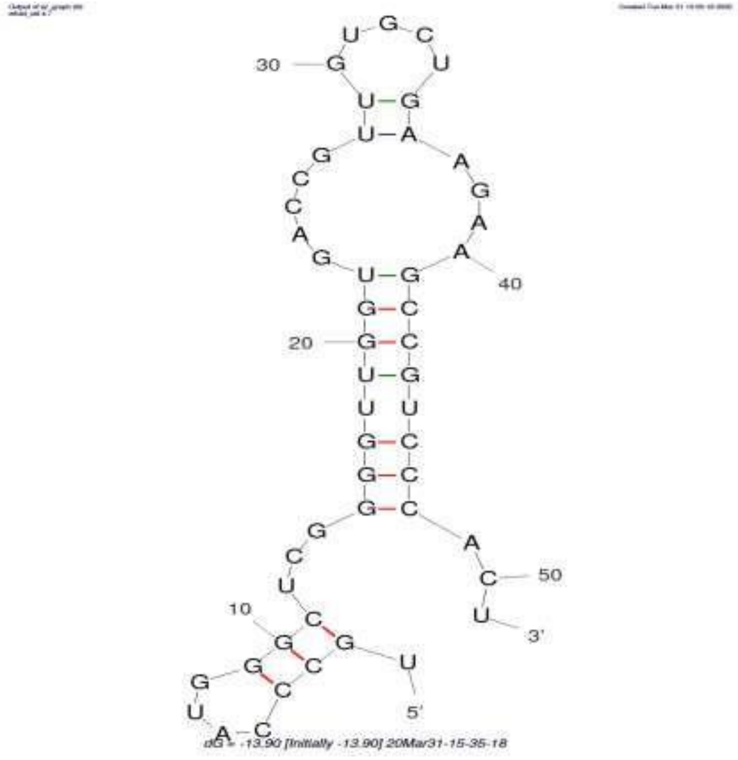

G

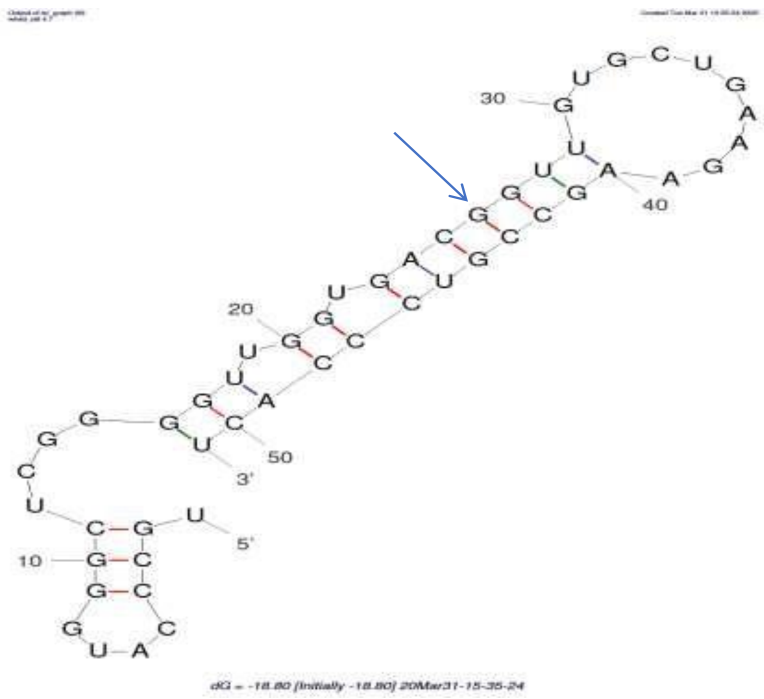

rs902335532

G

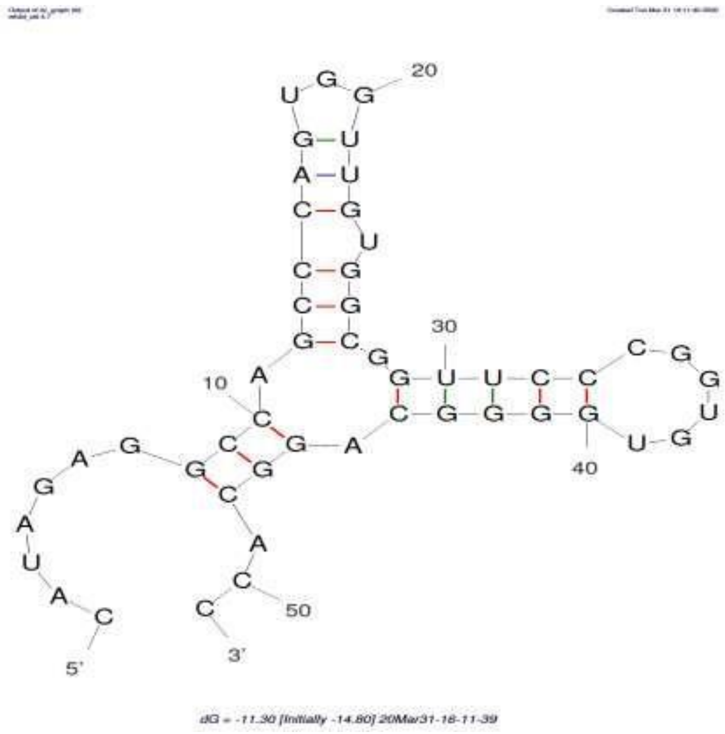

T

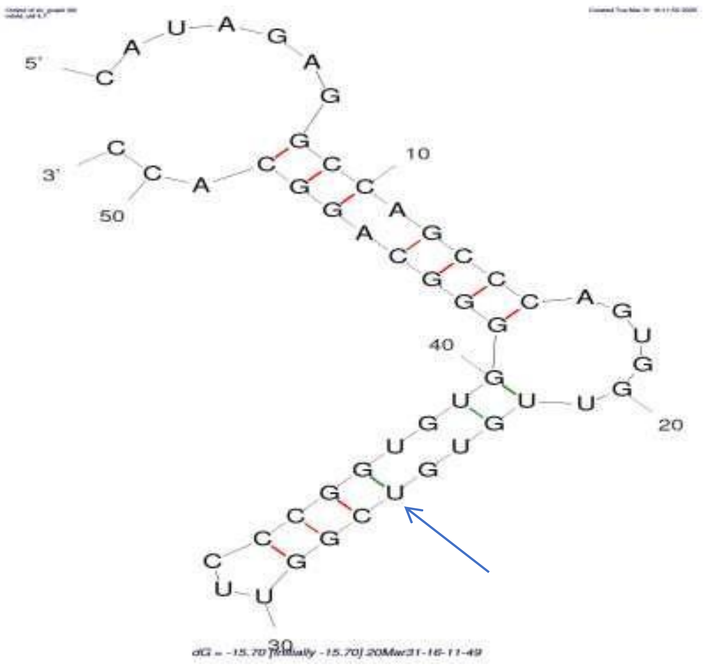

rs372868127

C

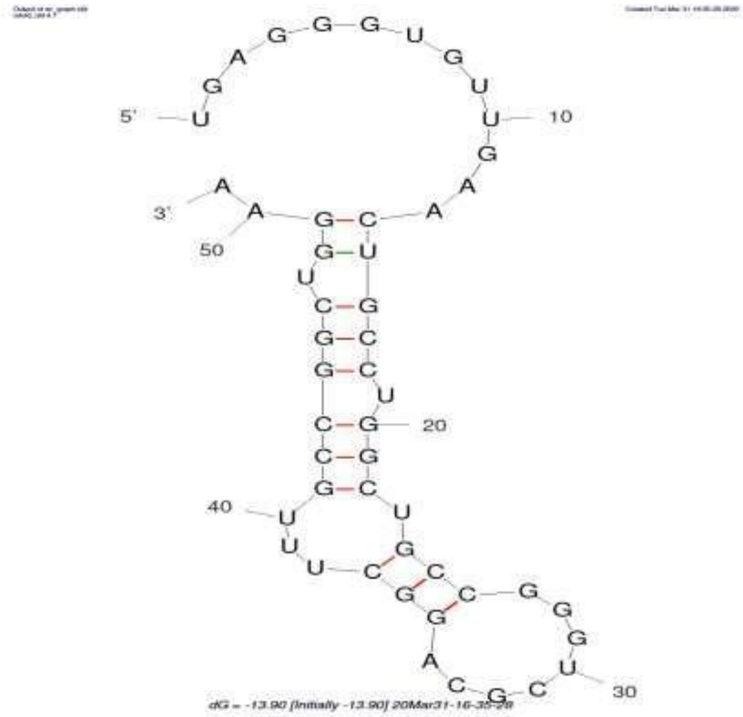

G

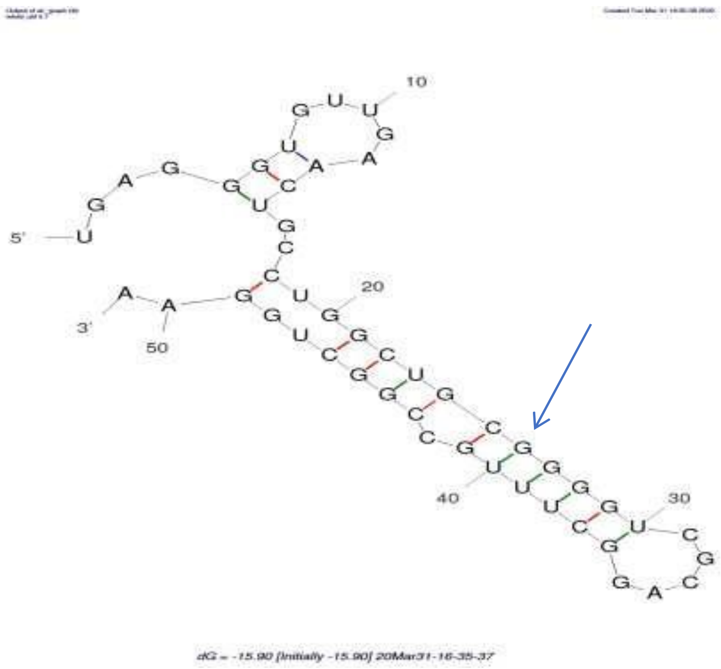

rs1472939030

C

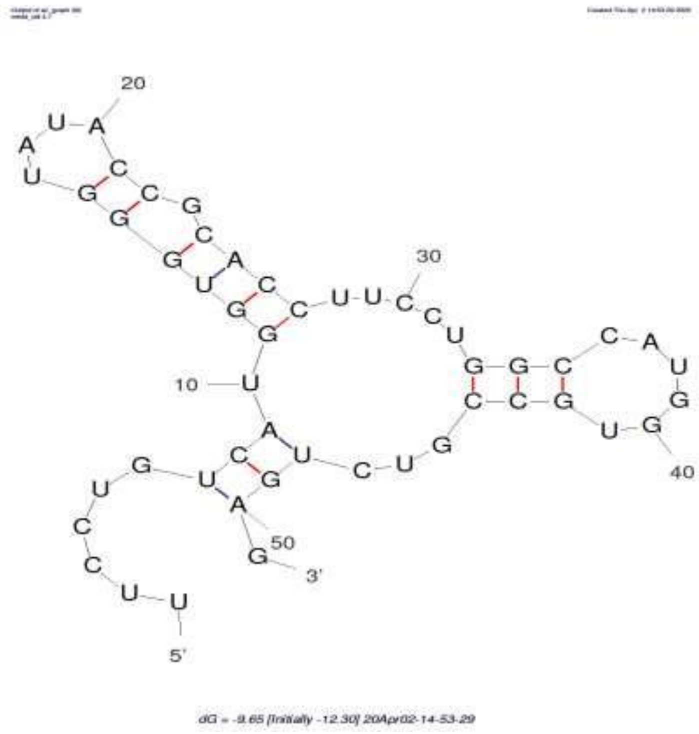

T

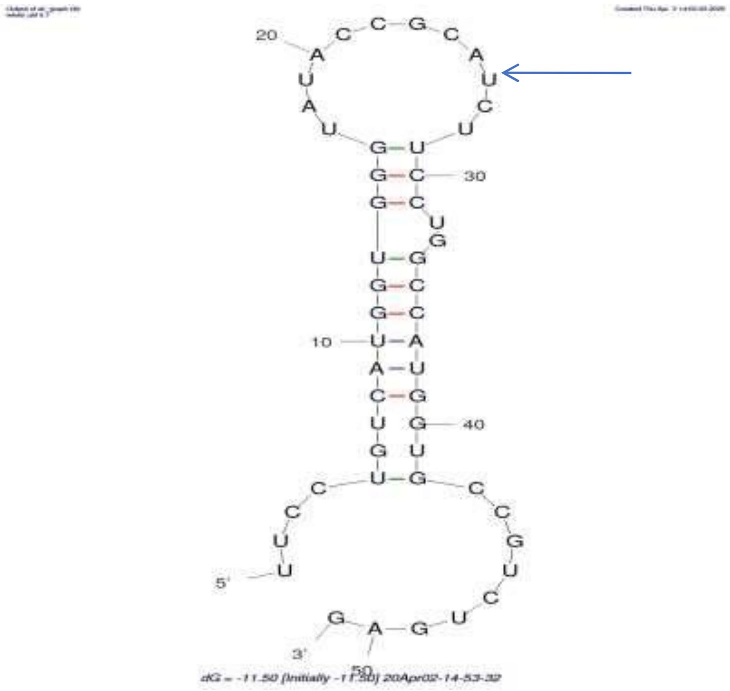

rs1452755314

A **T**

G

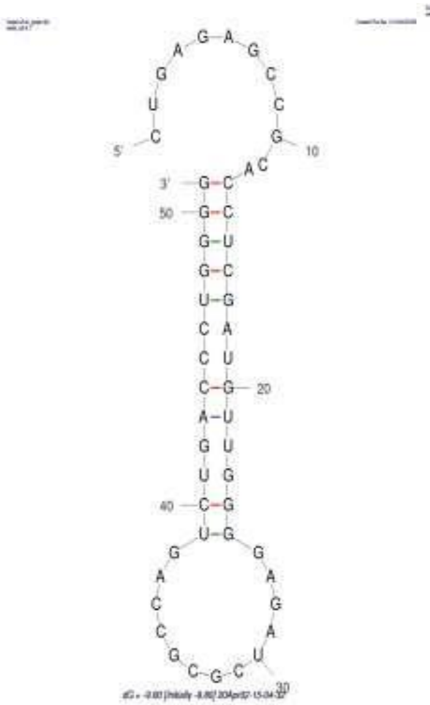

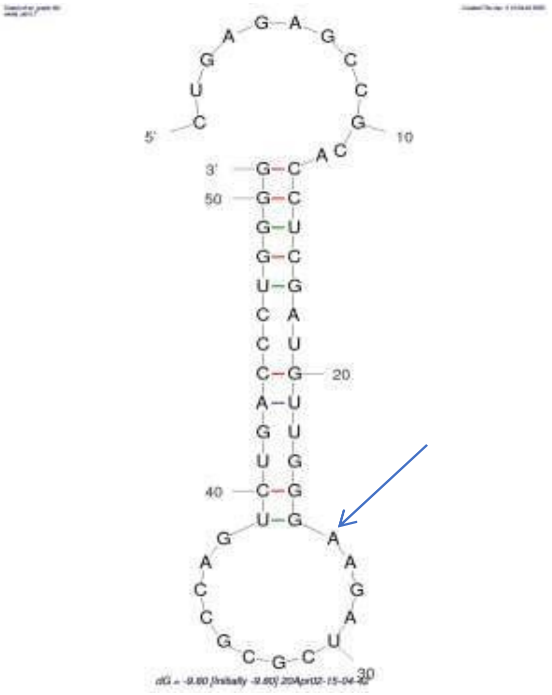

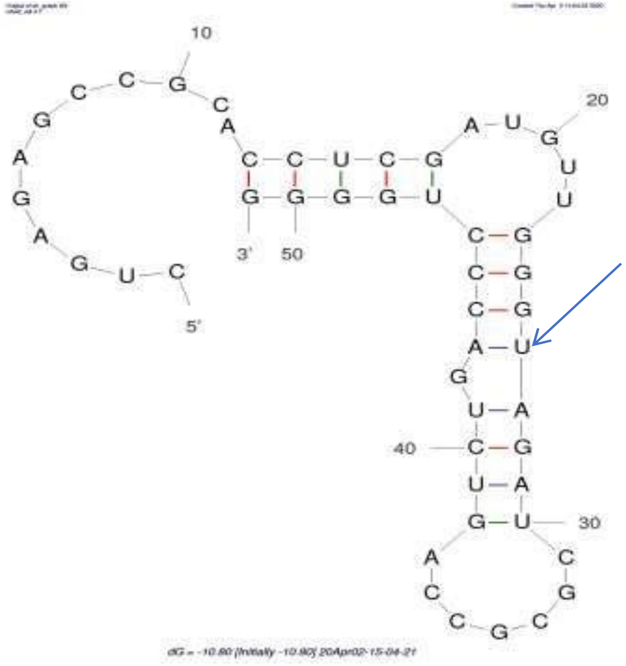

rs1371570796

C

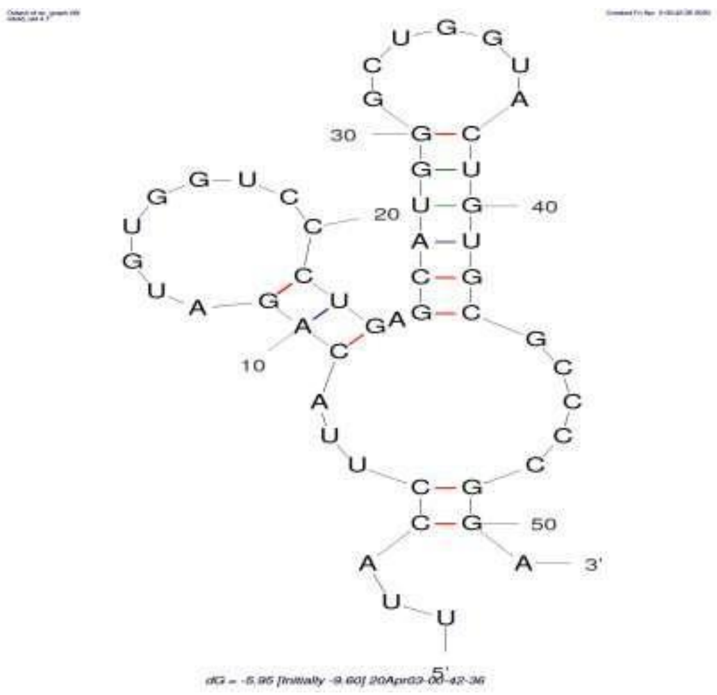

A

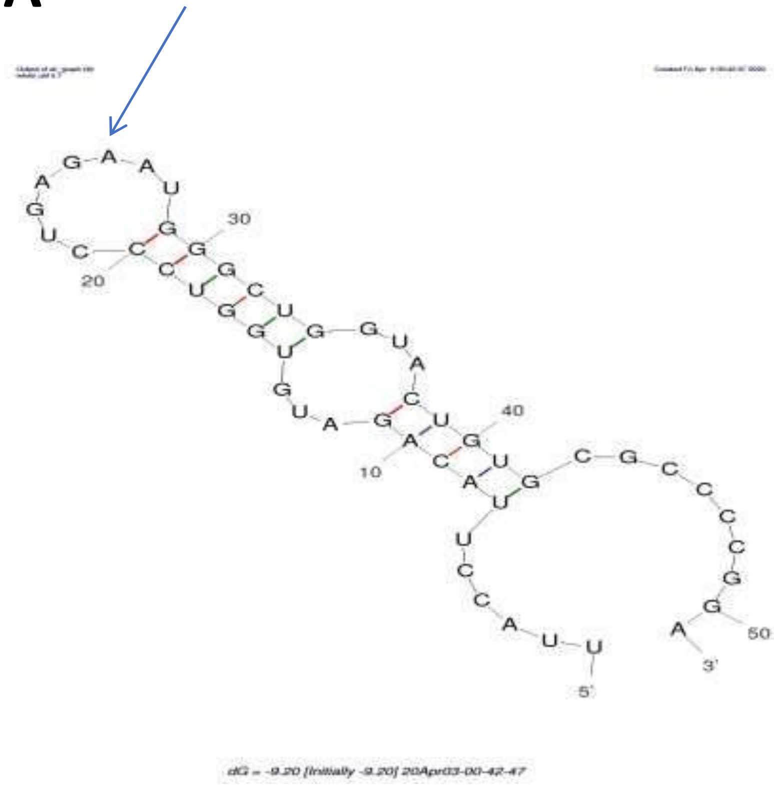

rs1243858519

G

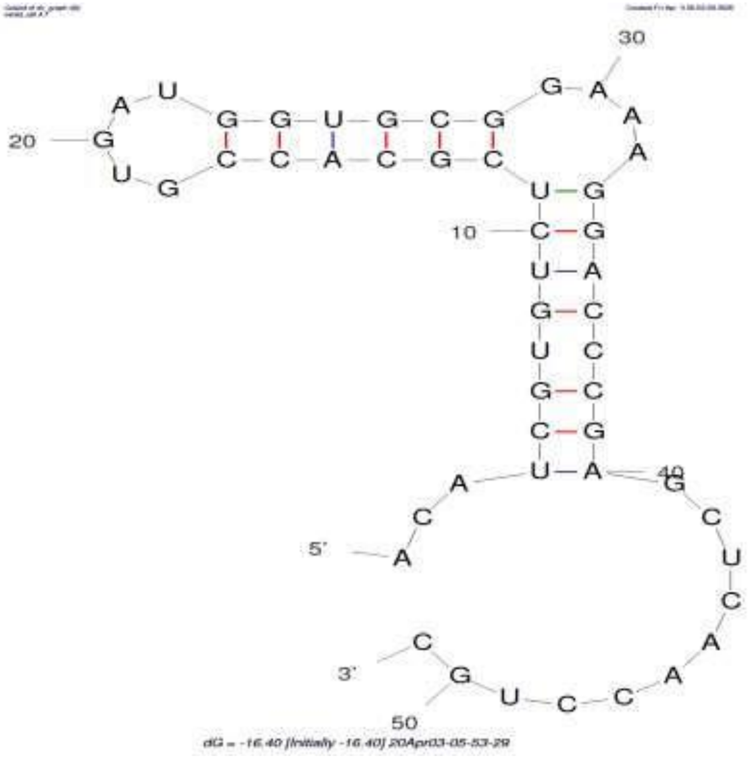

T

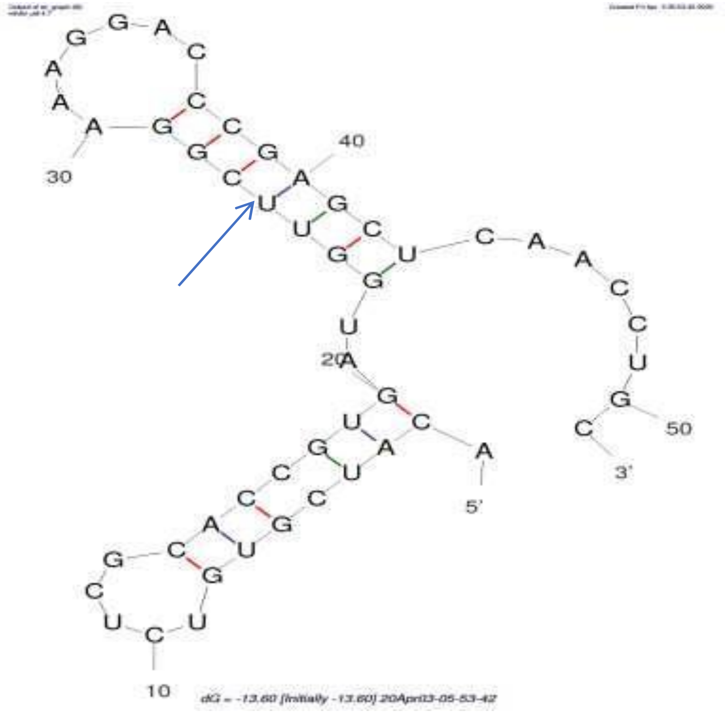

rs1476199849

G

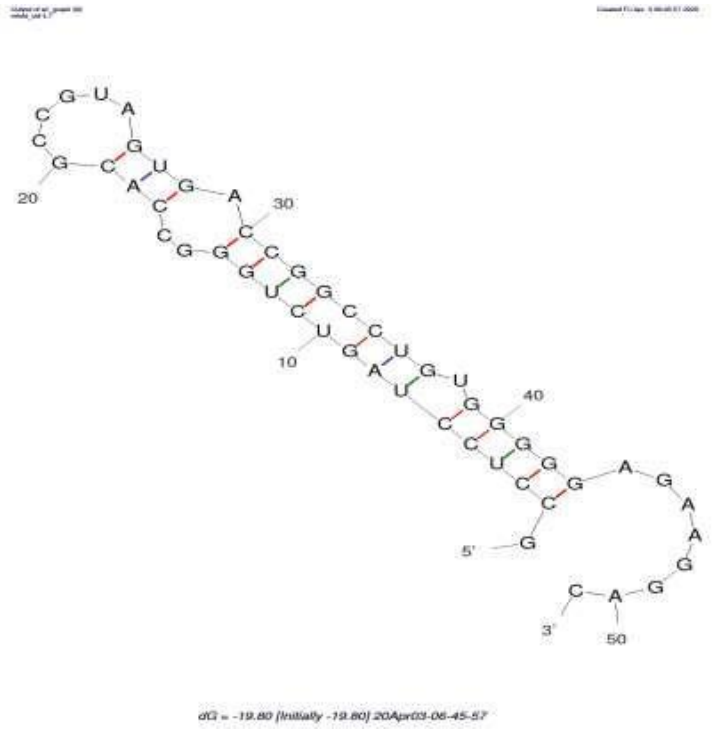

A

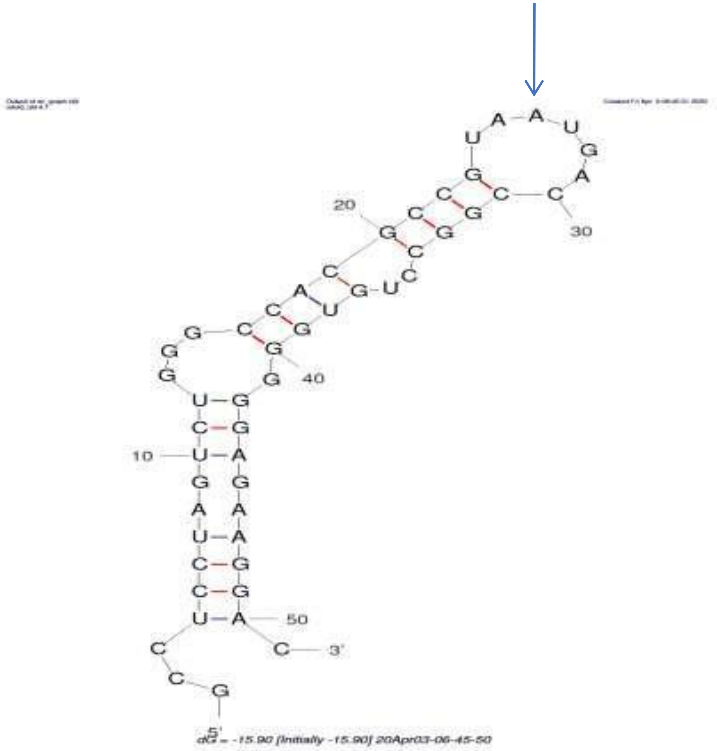

rs1436631668

C

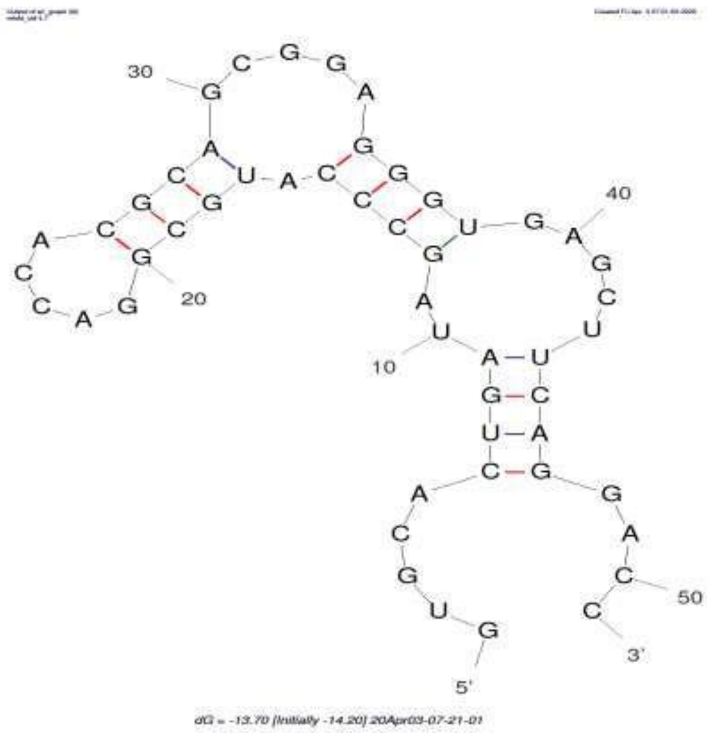

T

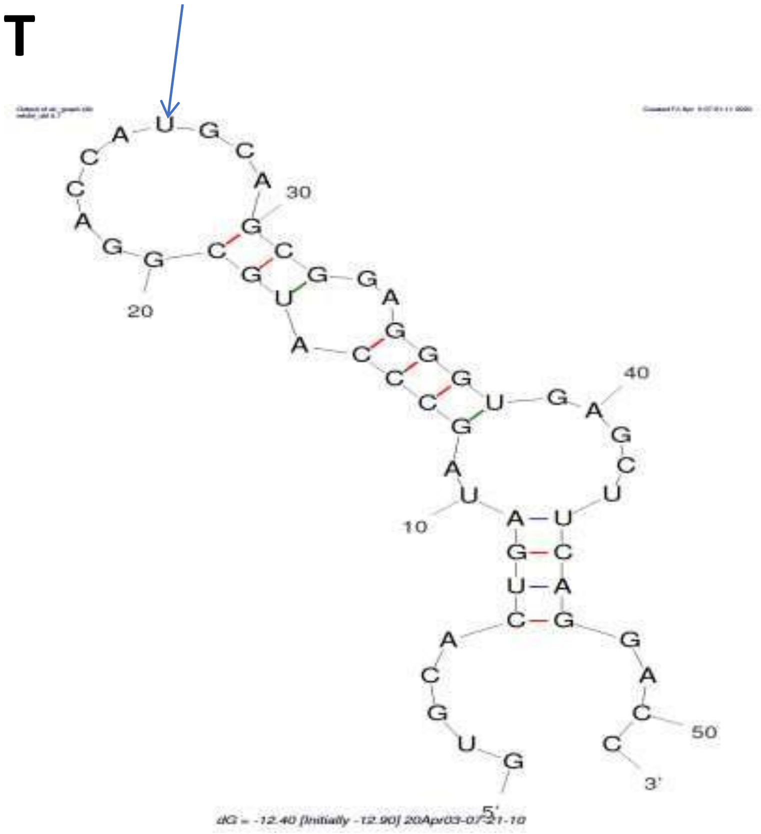

rs748976355

C

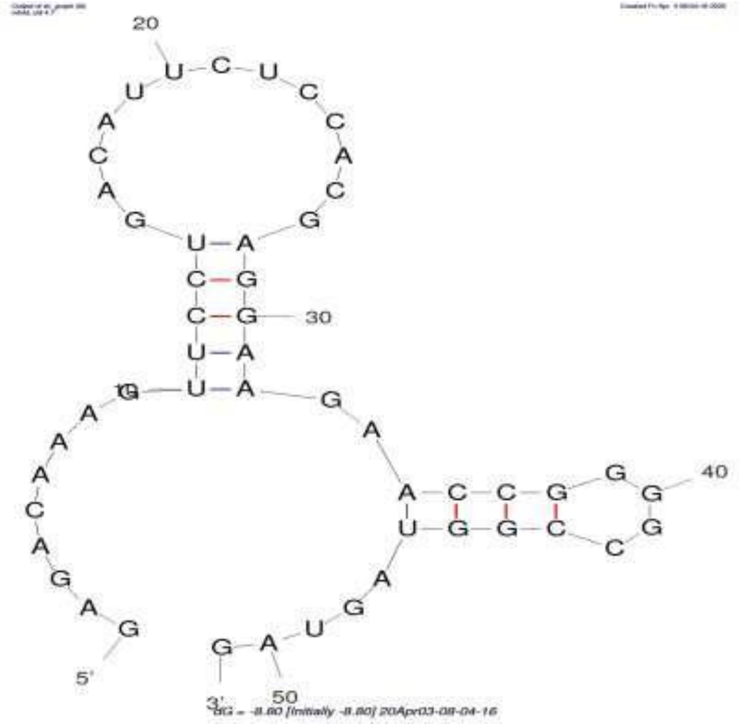

T

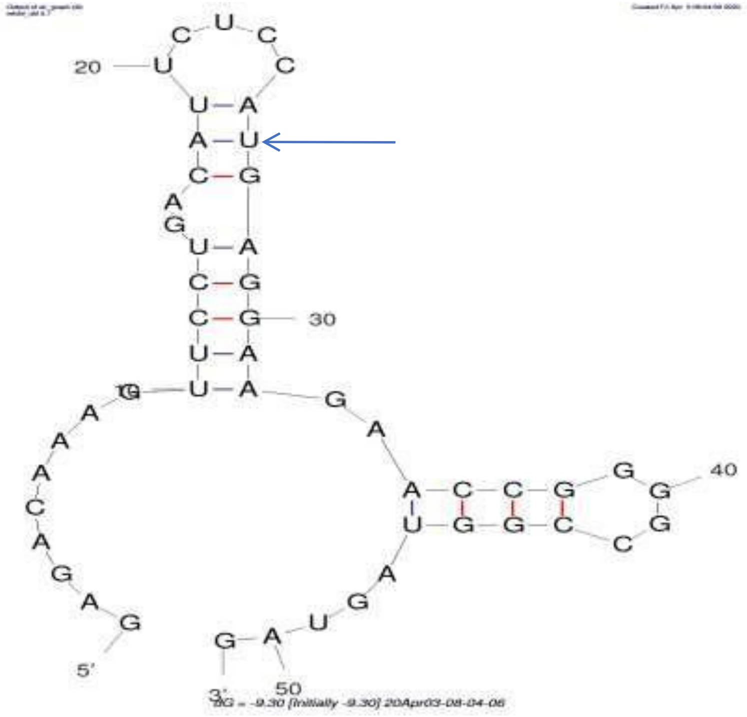

rs1405376714

G

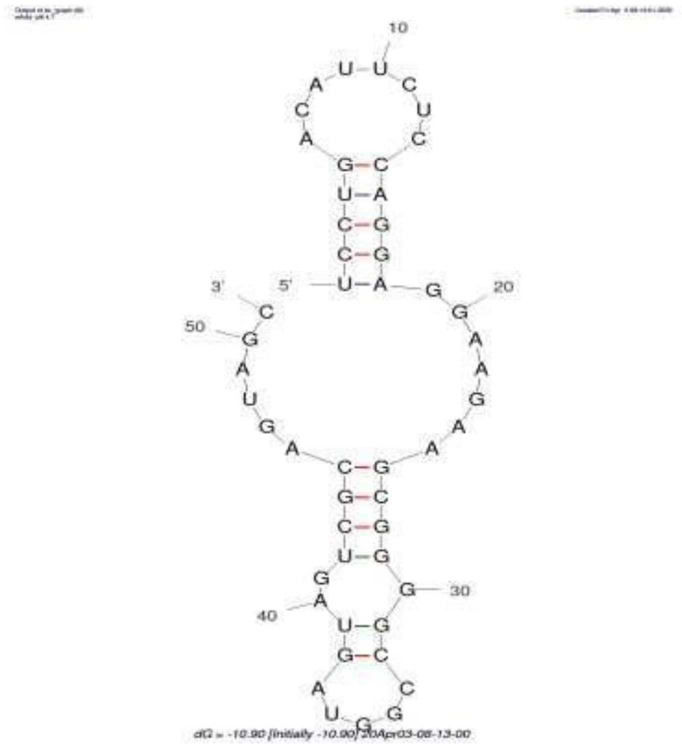

T

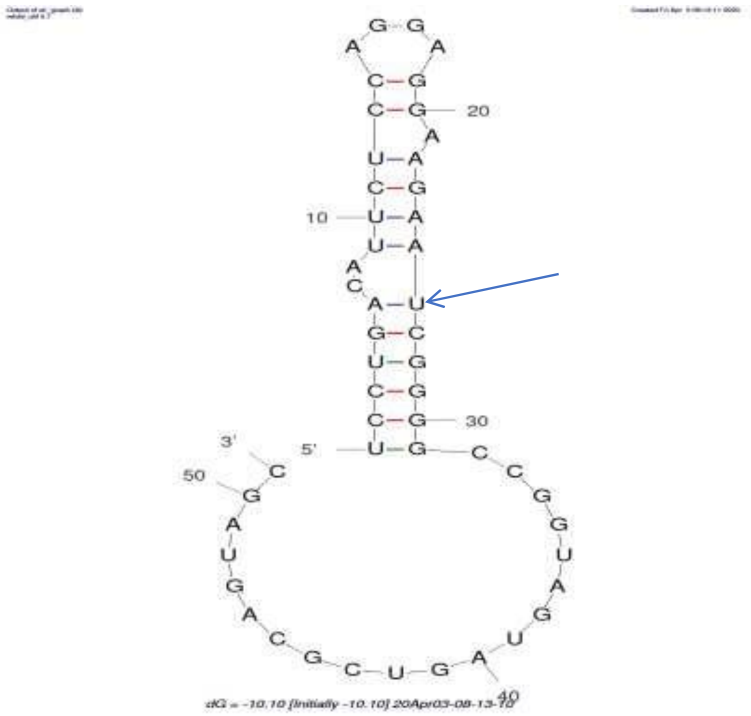

rs772090333

C

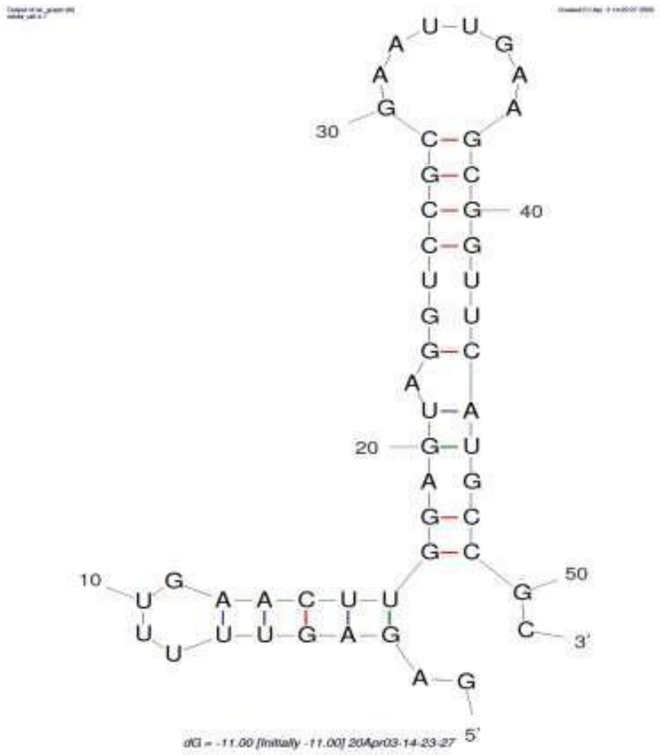

T

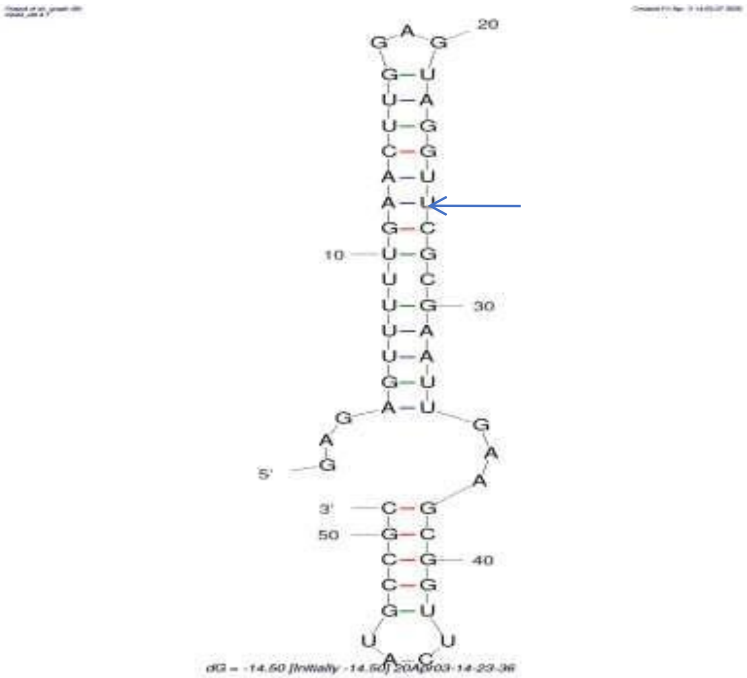

rs768760115

G

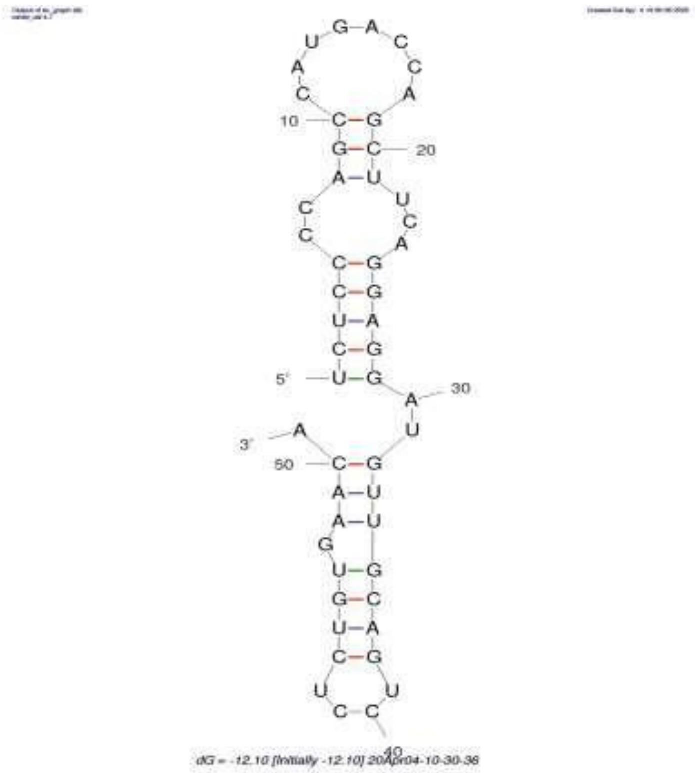

A

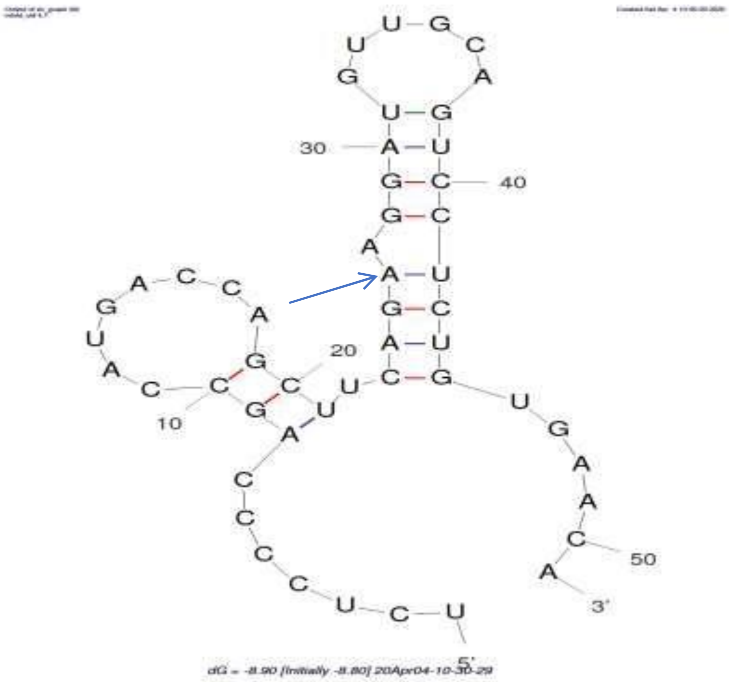

rs1489027030

G

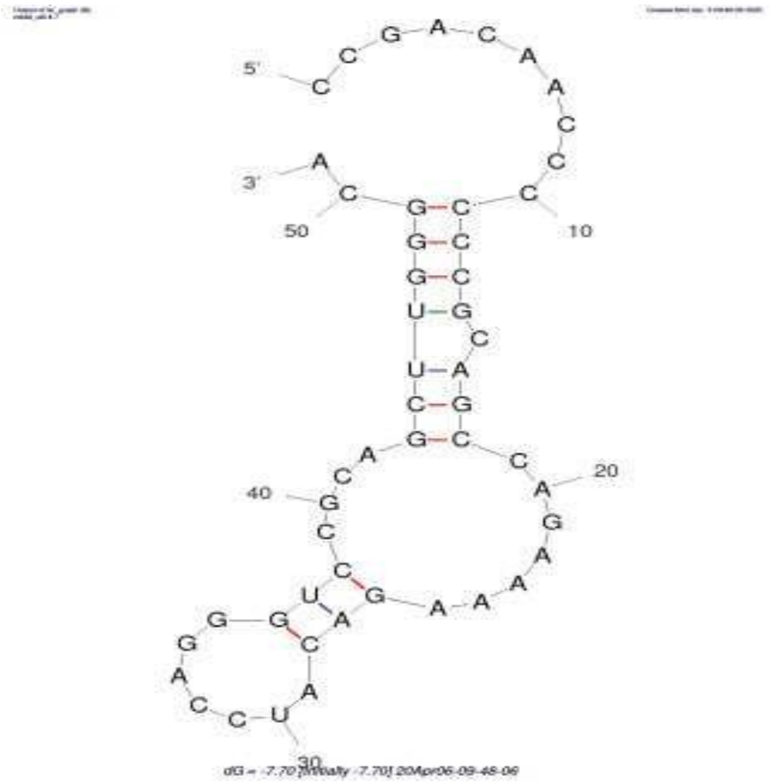

T

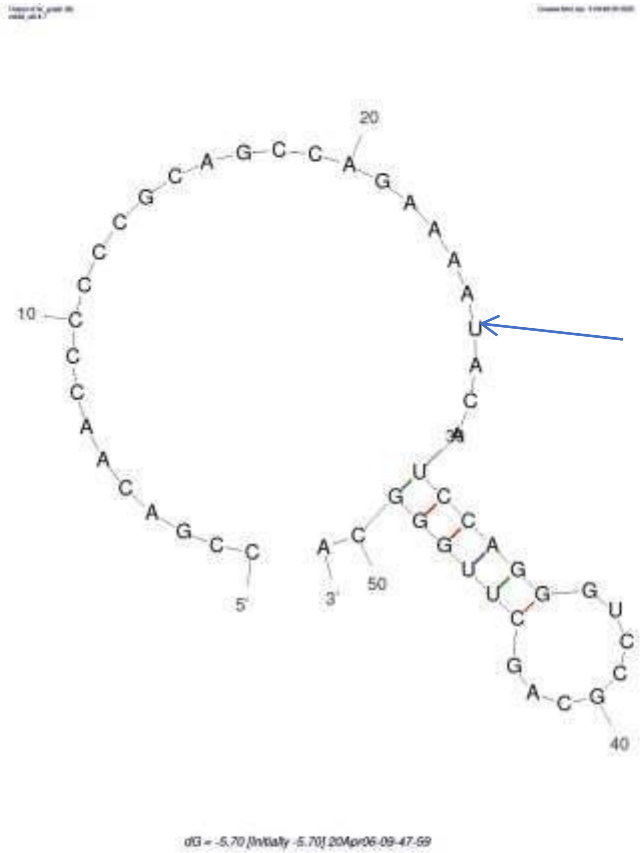

rs1263434012

C

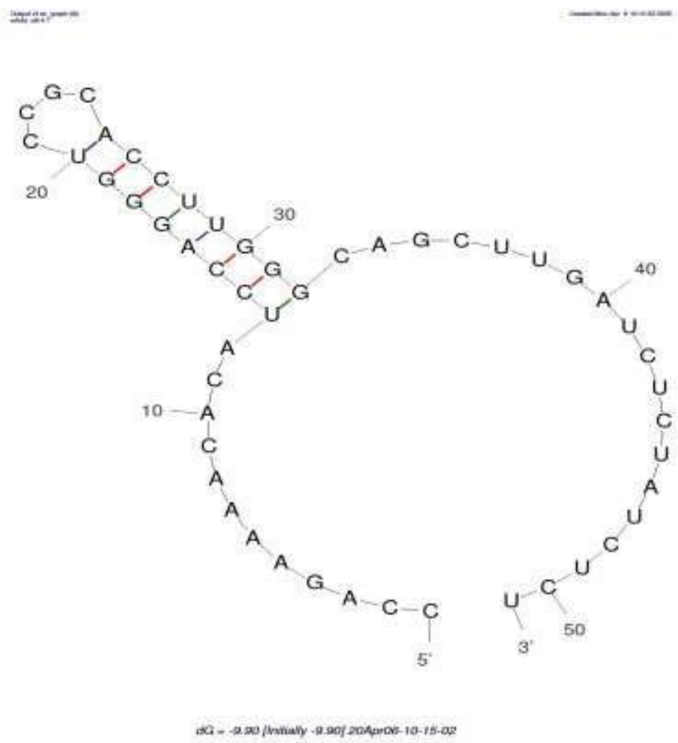

T

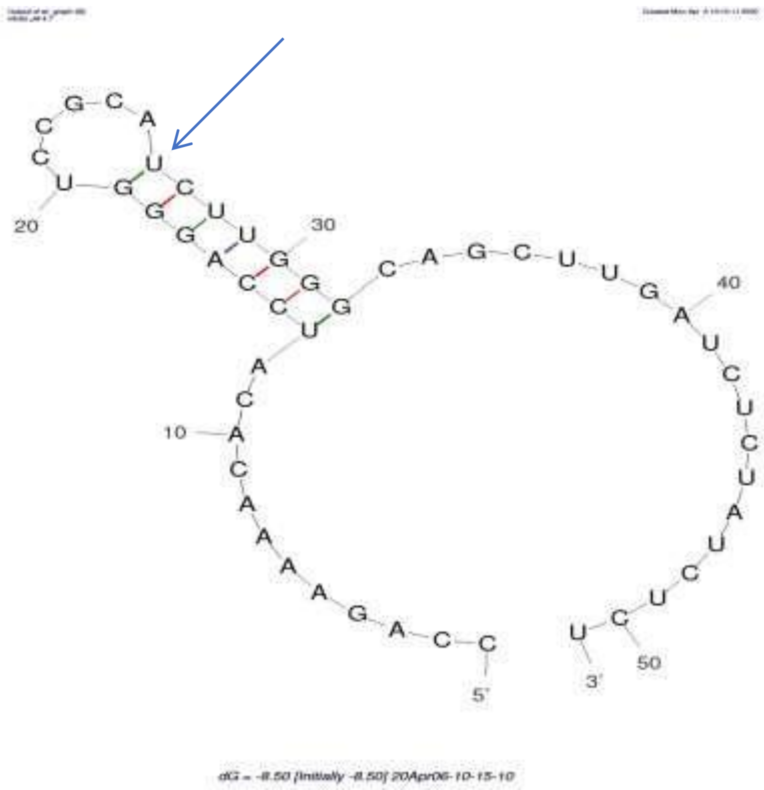

rs144675407

C

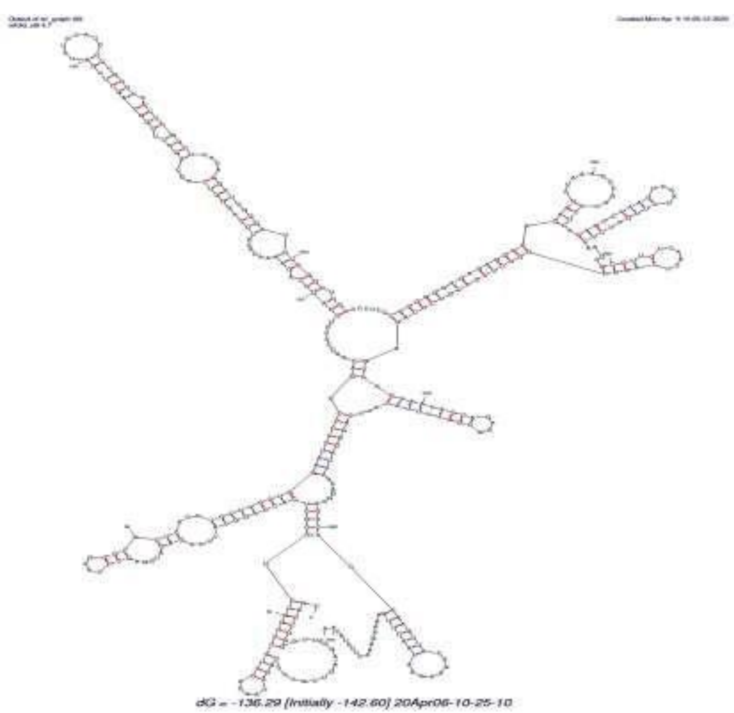

G

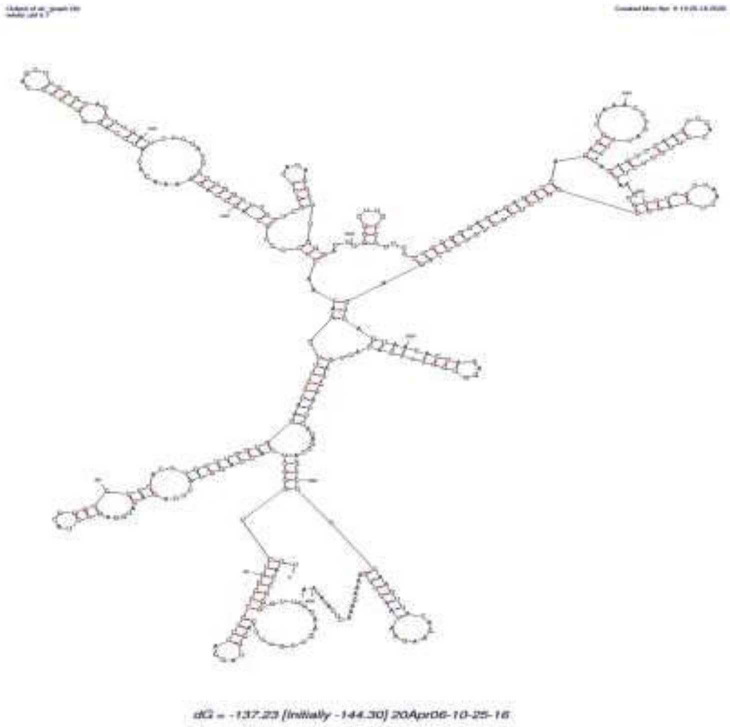

rs1265124271

G

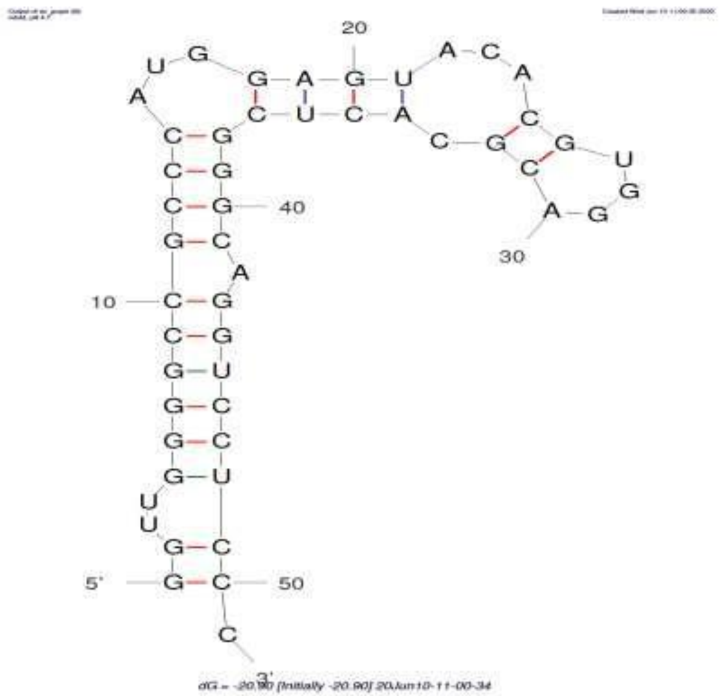

A

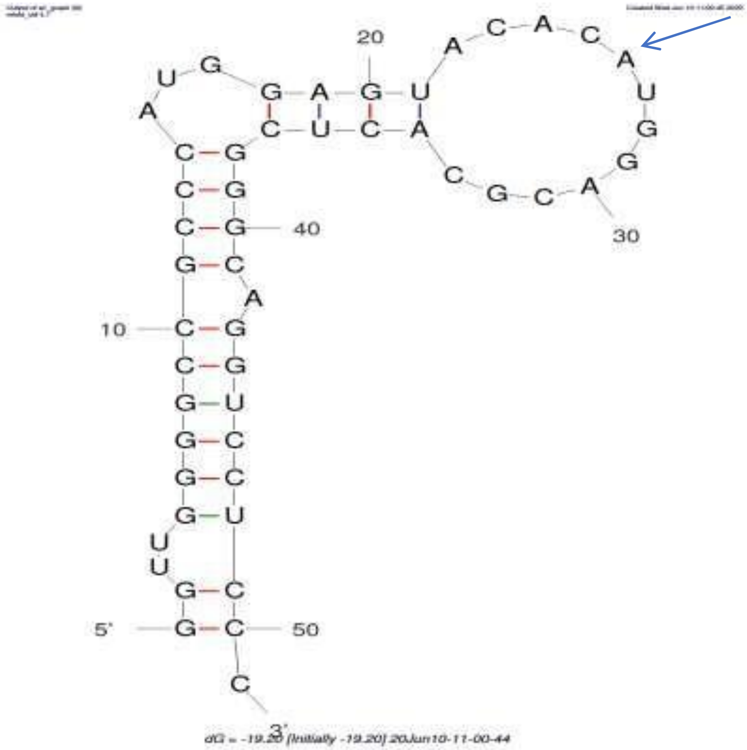

rs1459002984

G

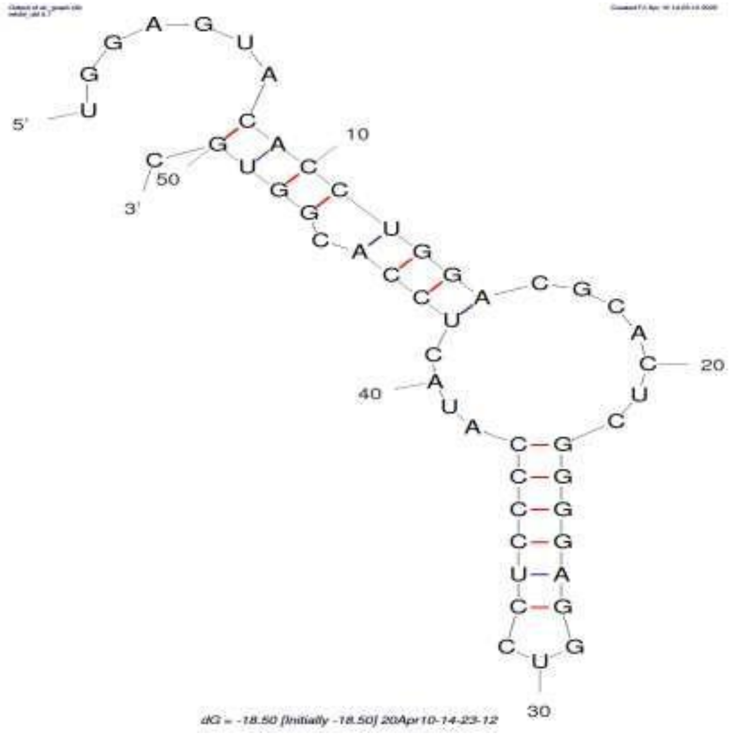

A

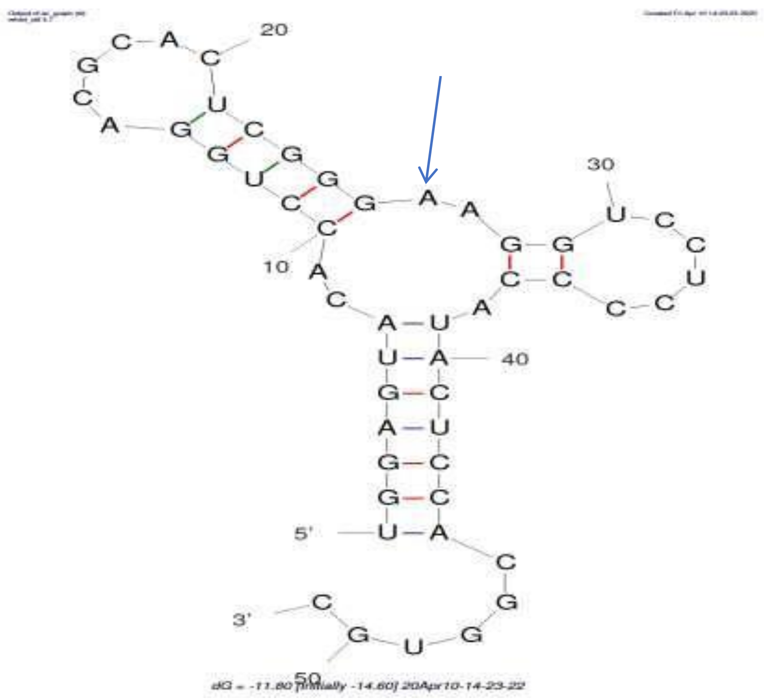

rs780059022

G

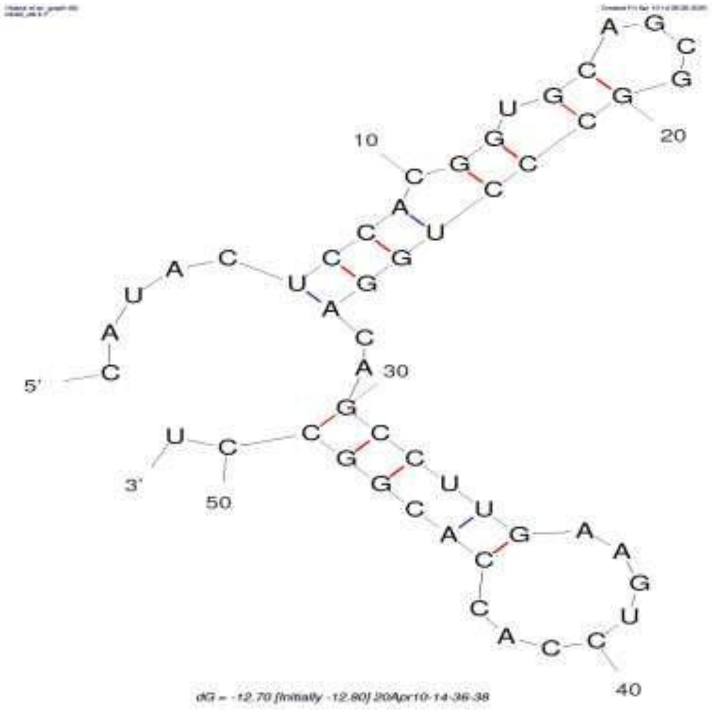

T

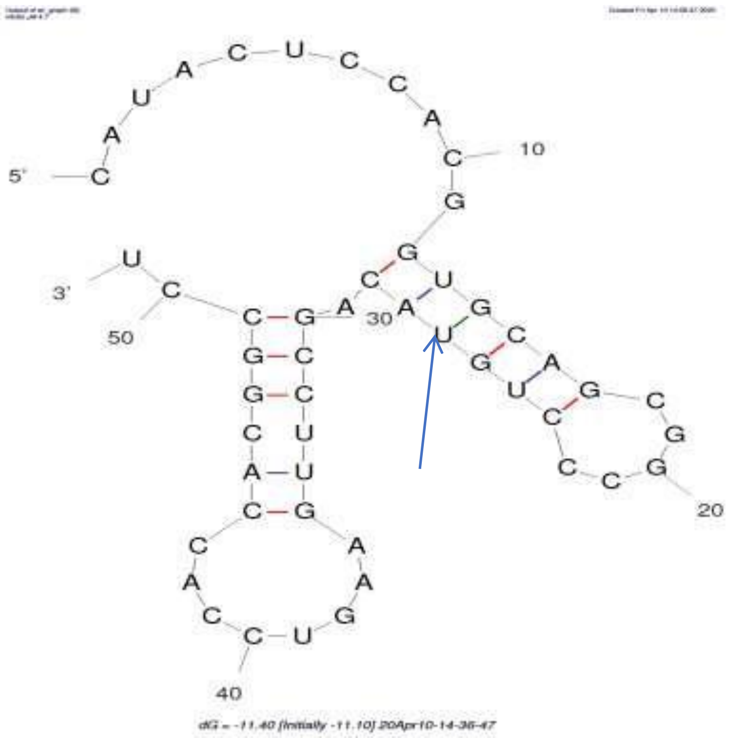

rs150359172

C

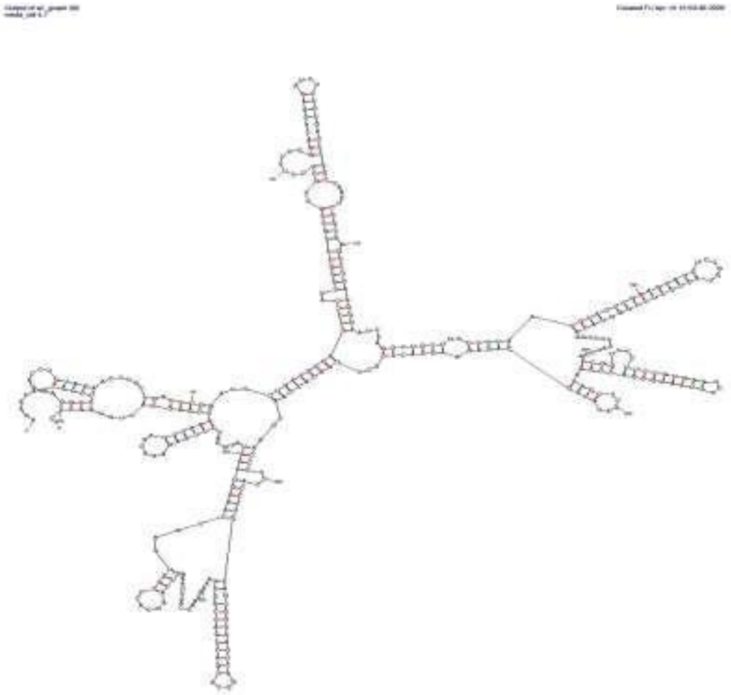

dG = -128.01 [Initially -137.40] 20Apr10-15-53-44

G

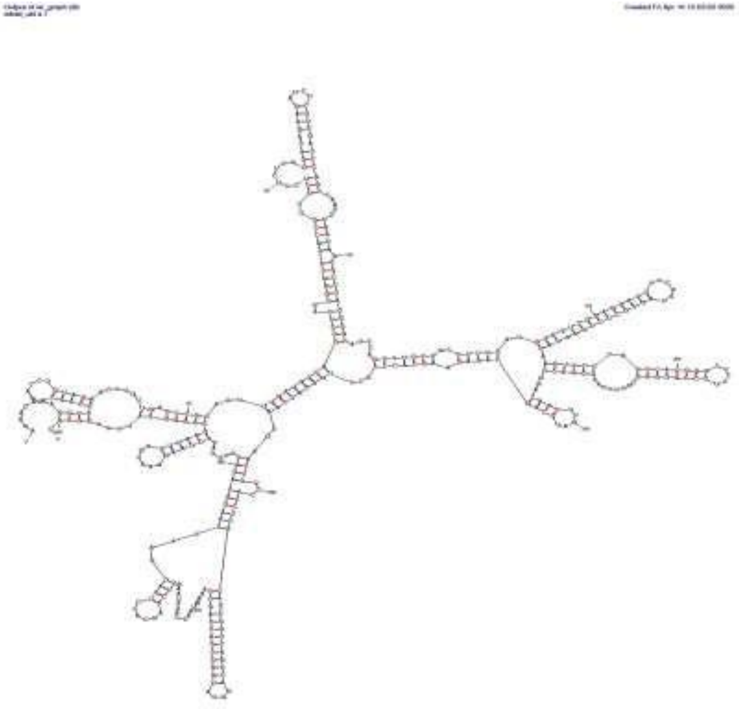

dG = -133.84 [Initially -140.60] 20Apr10-15-53-51

rs1183694434

A

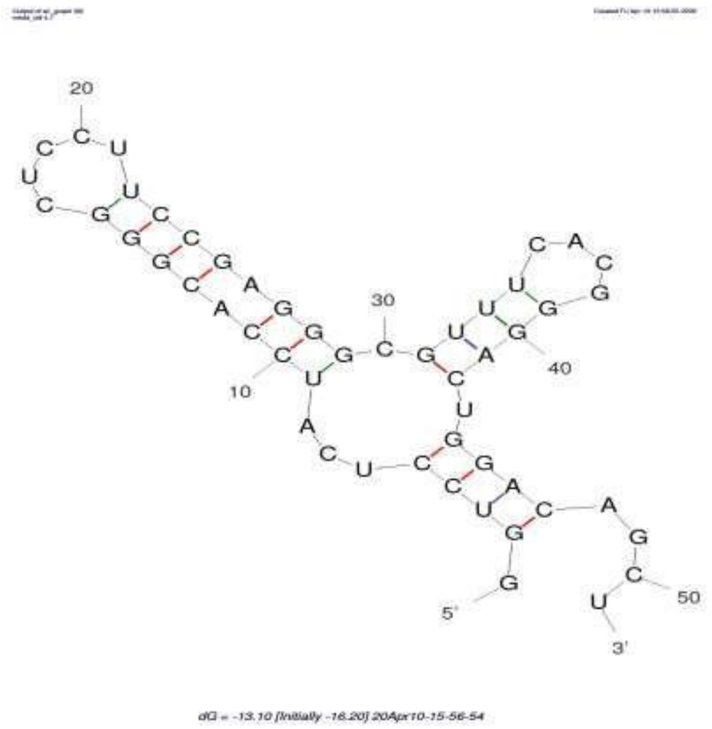

G

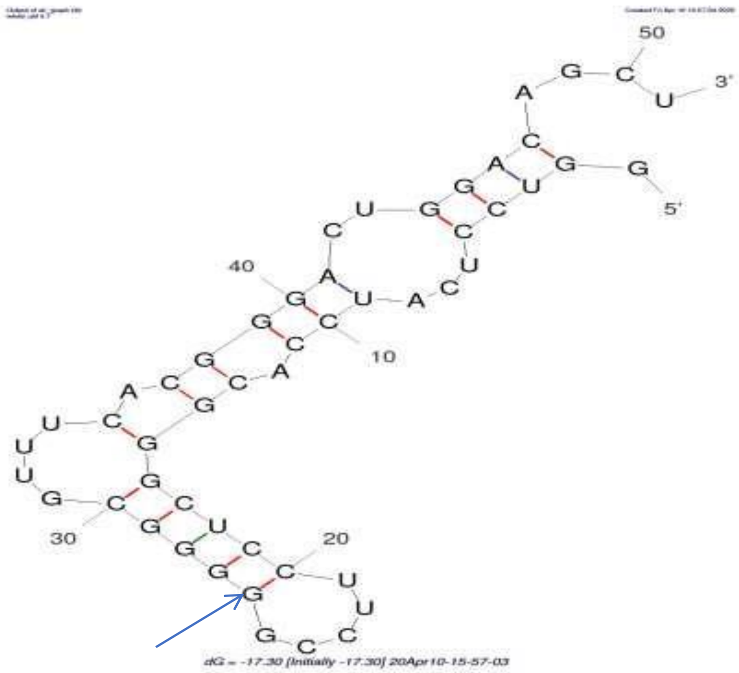

rs771873513

C

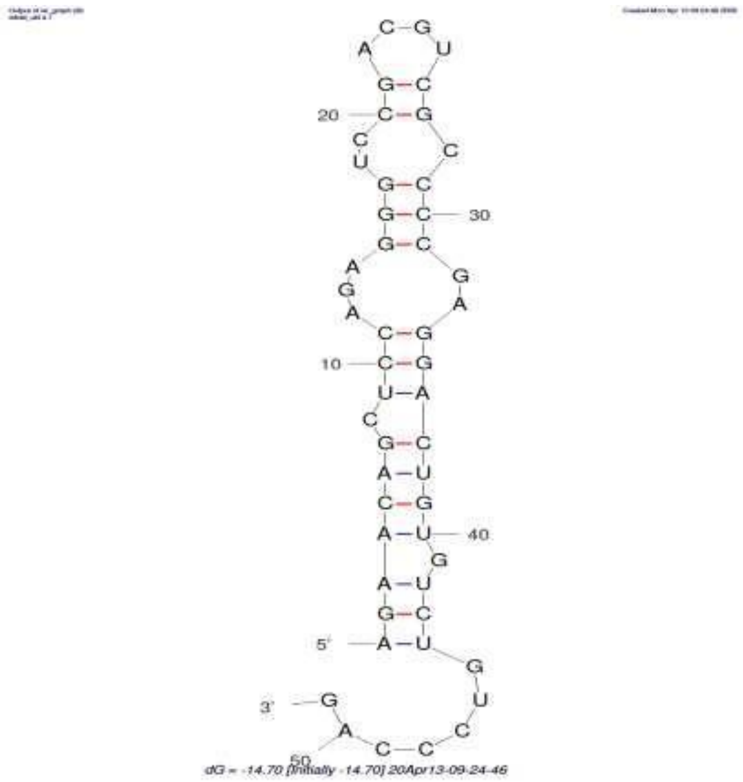

G

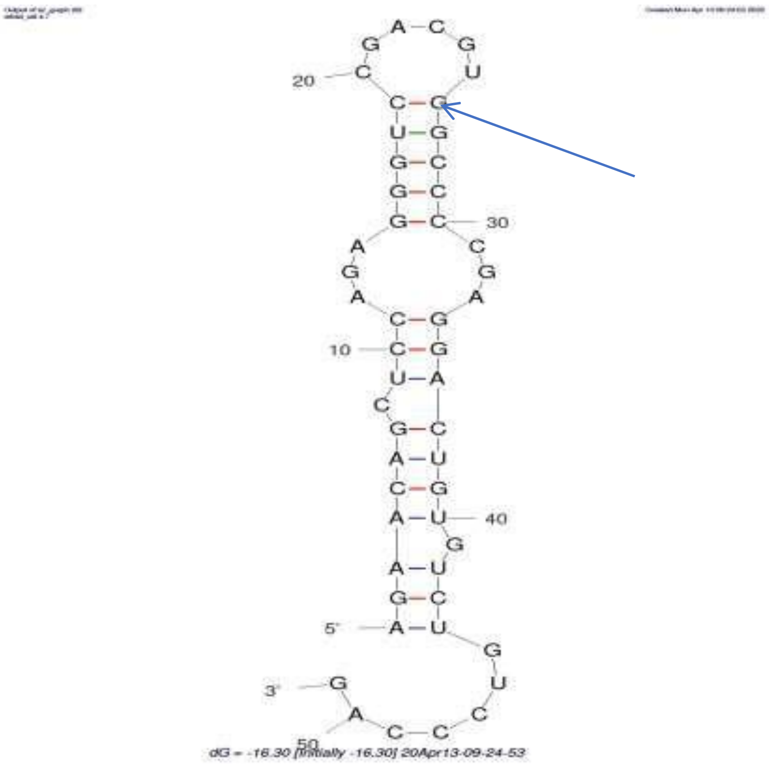

rs1175415967

C

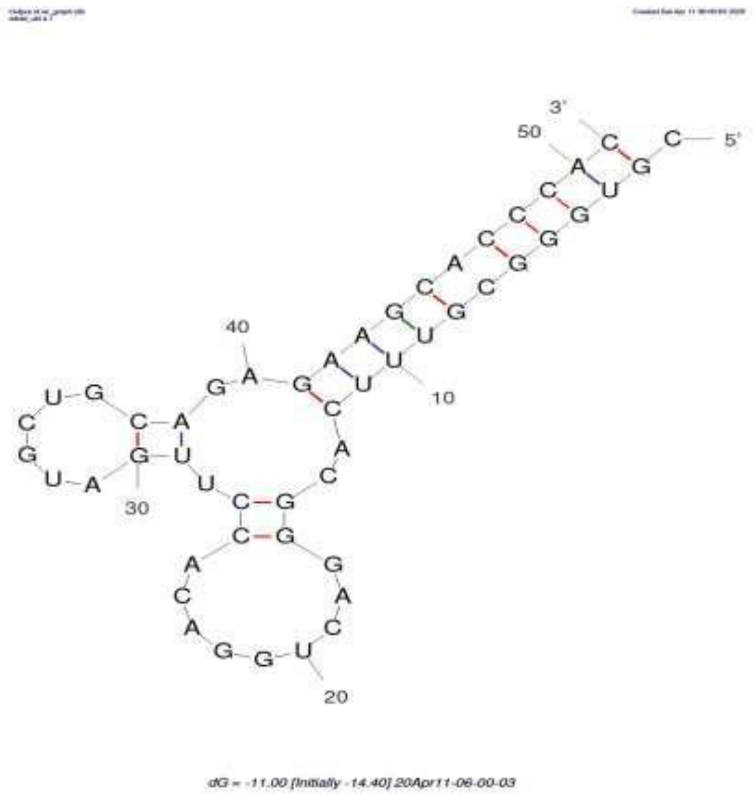

T

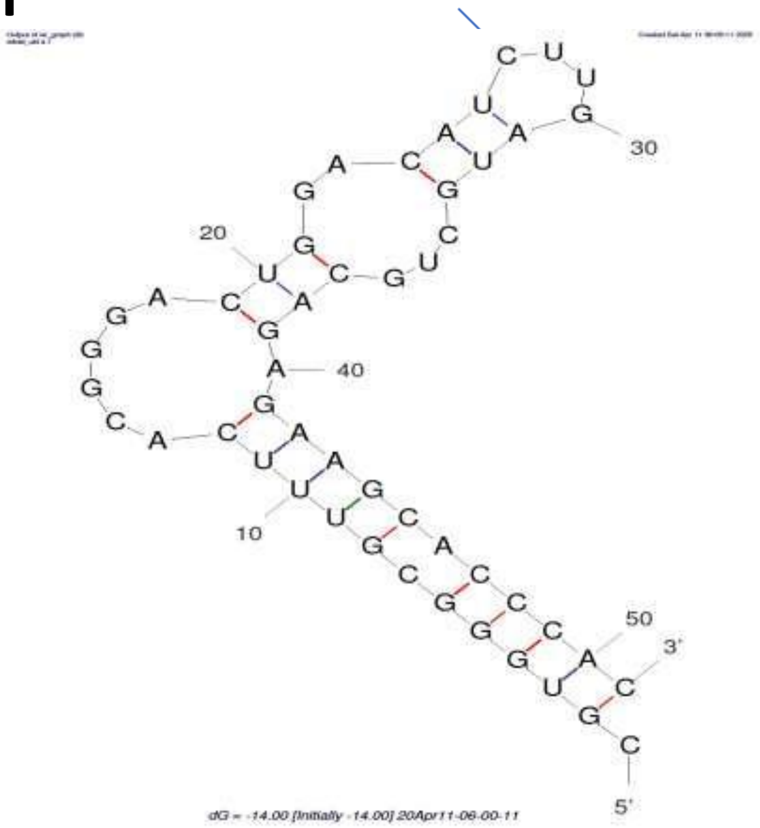

rs1427221135

T

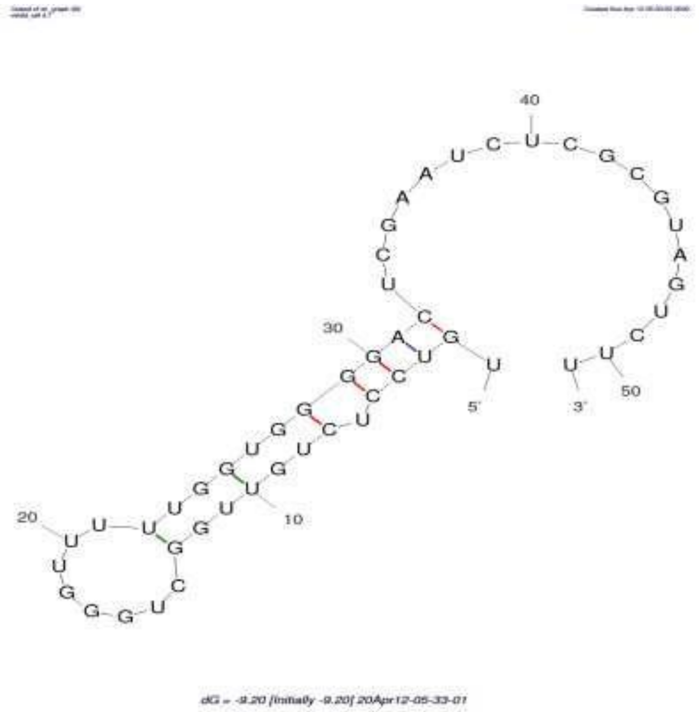

G

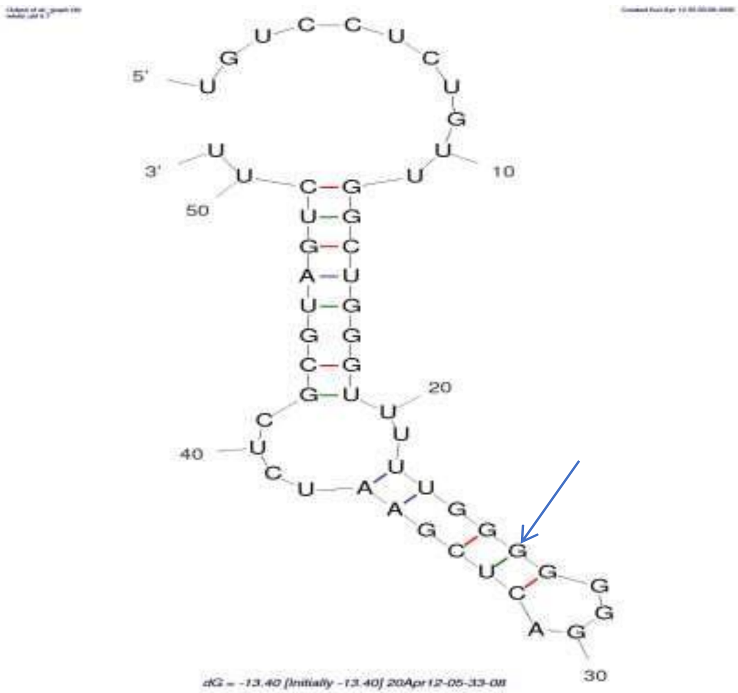

rs1419289846

C

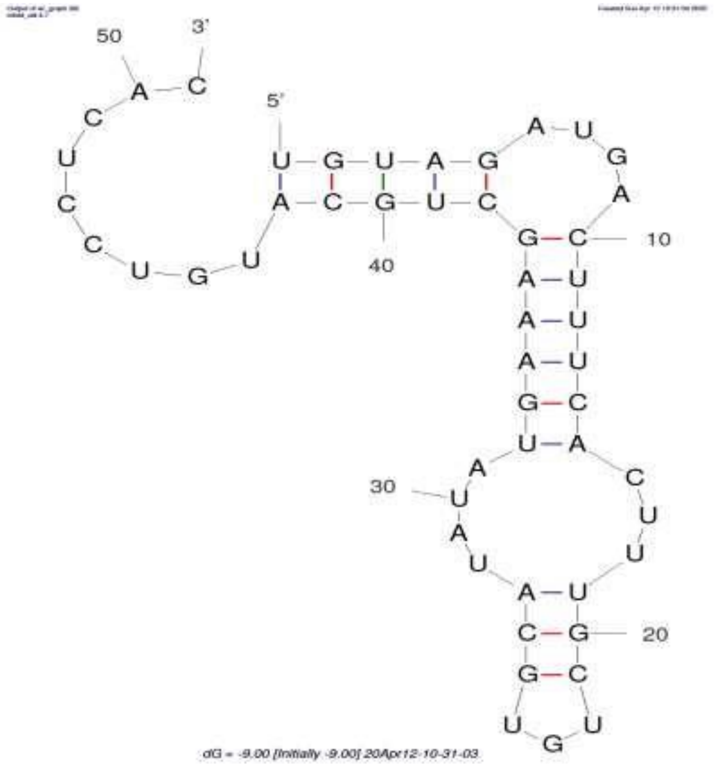

A

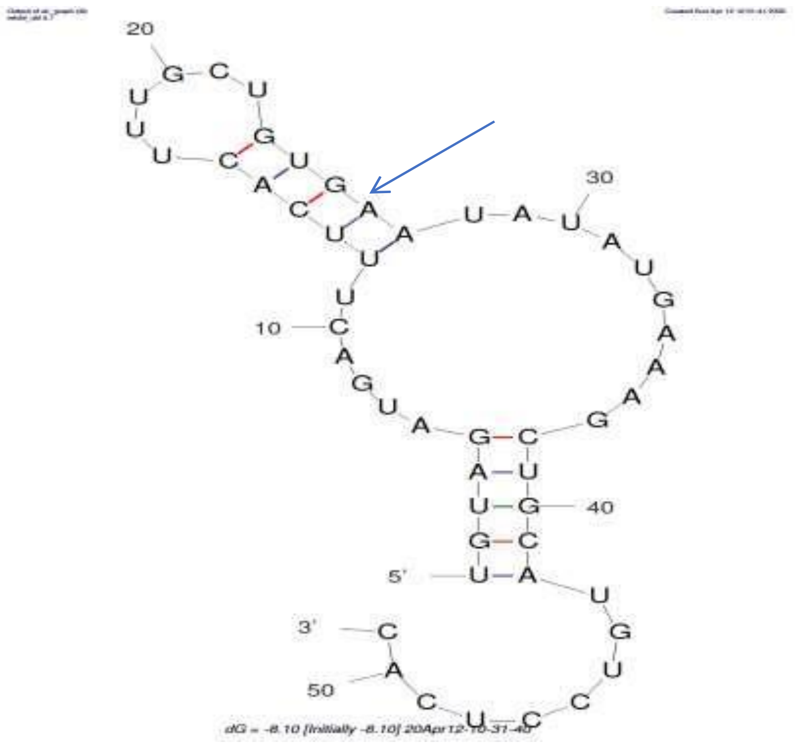

rs749306478

G

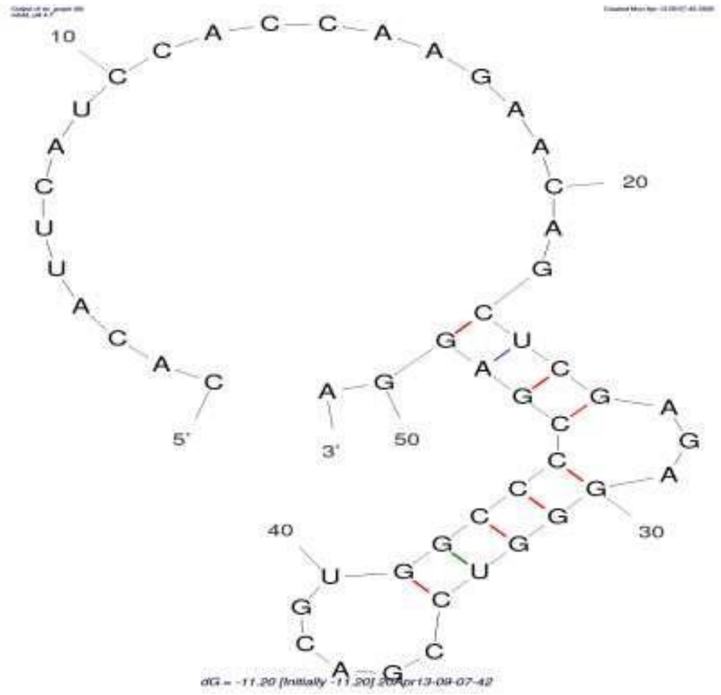

A

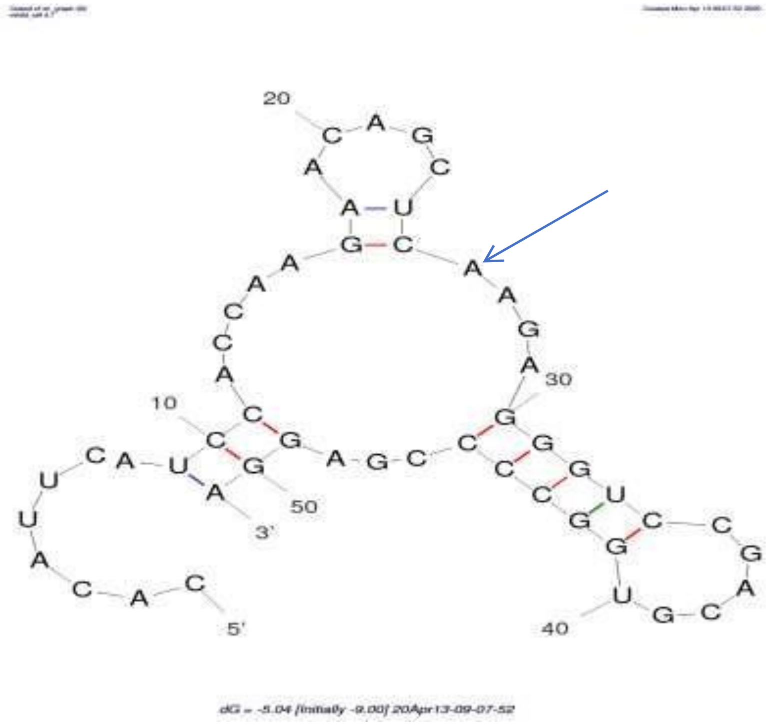

rs763497211

A

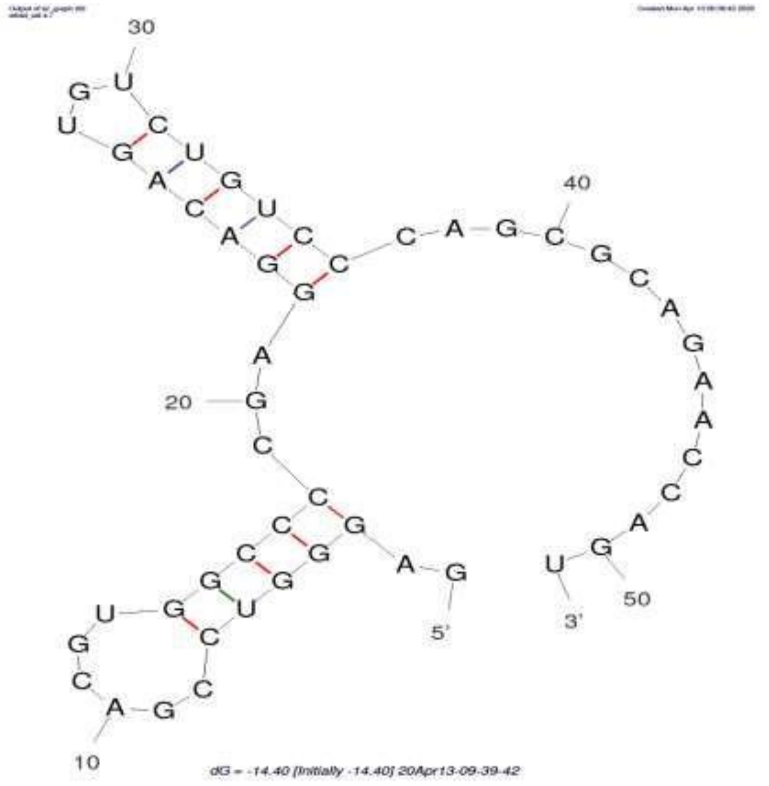

G

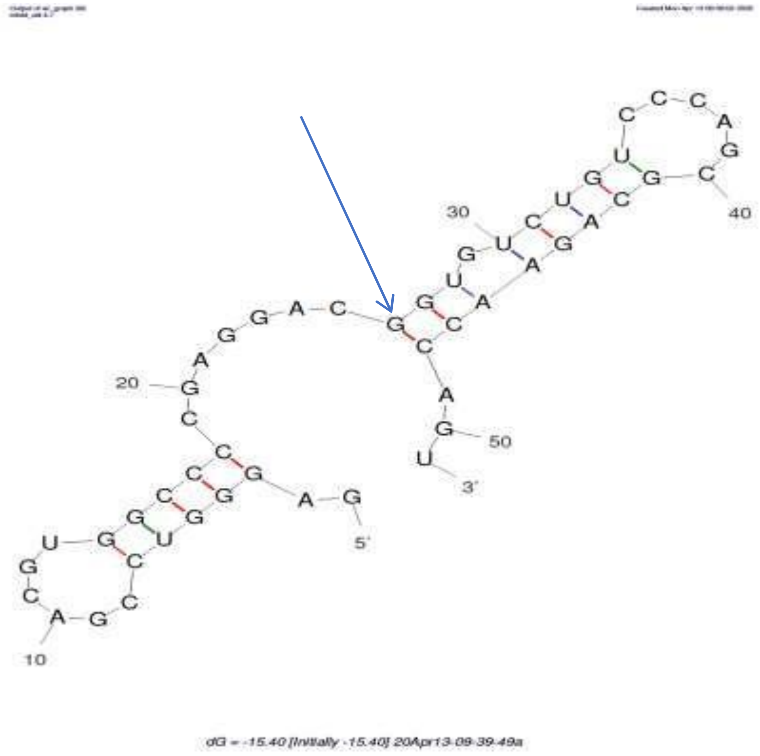

rs112405538

A

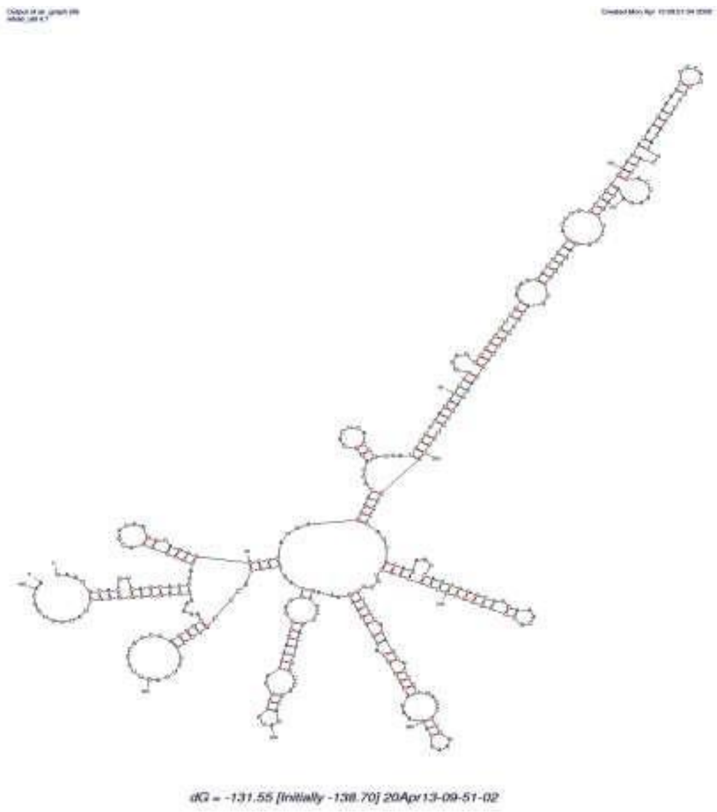

G

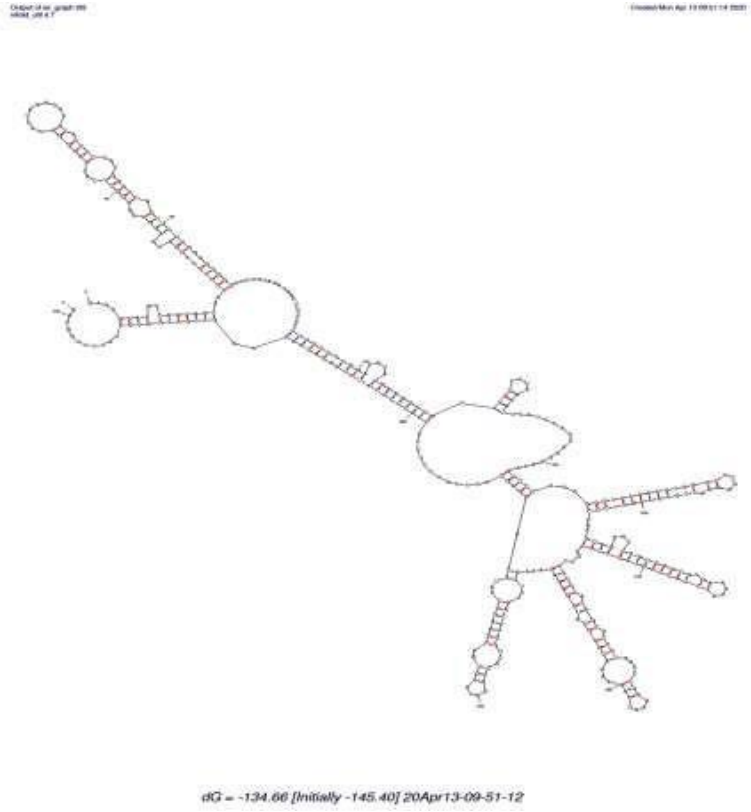

rs915110490

C

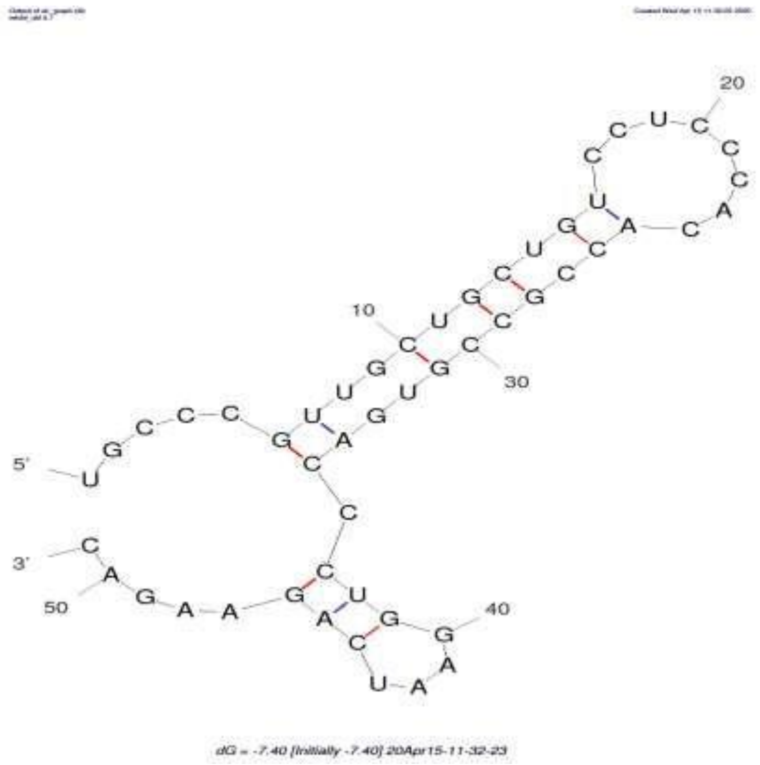

T

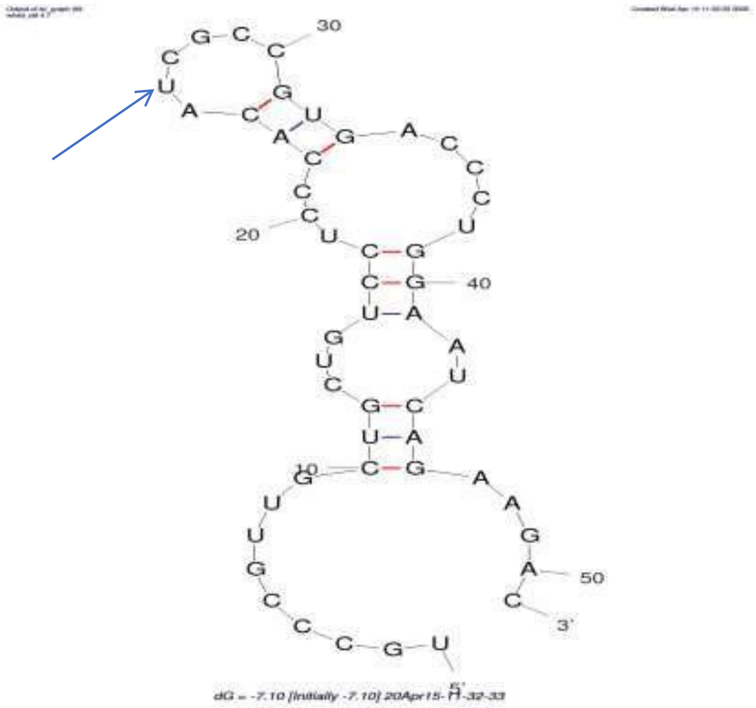

rs757830475

A

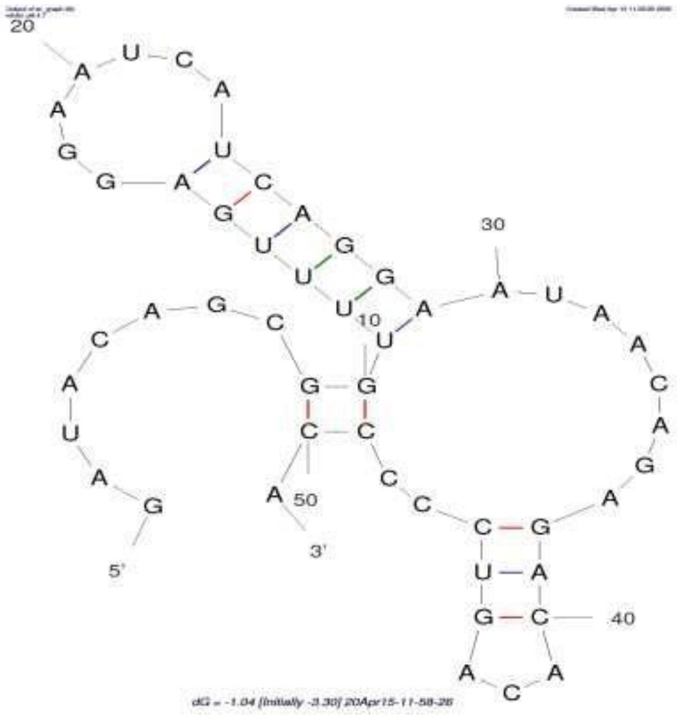

G

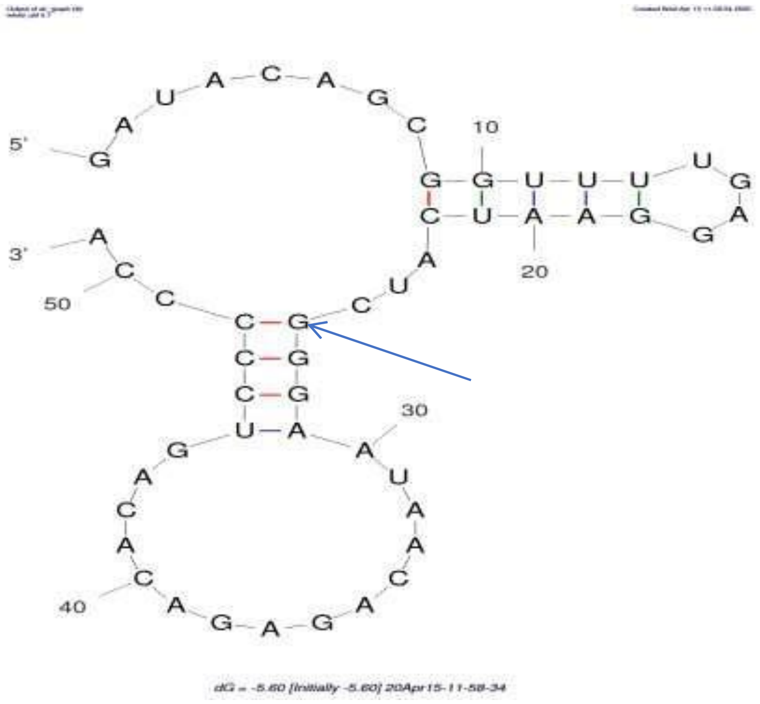

rs532599778

C

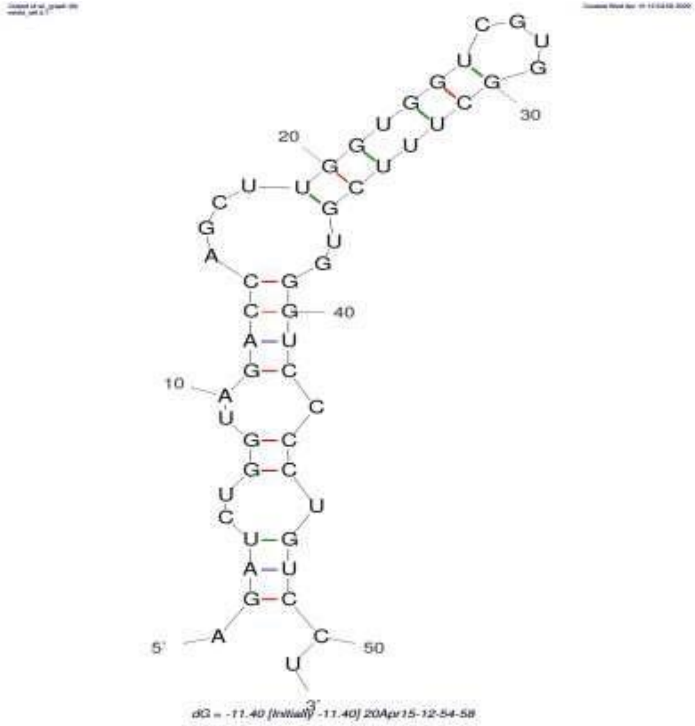

G

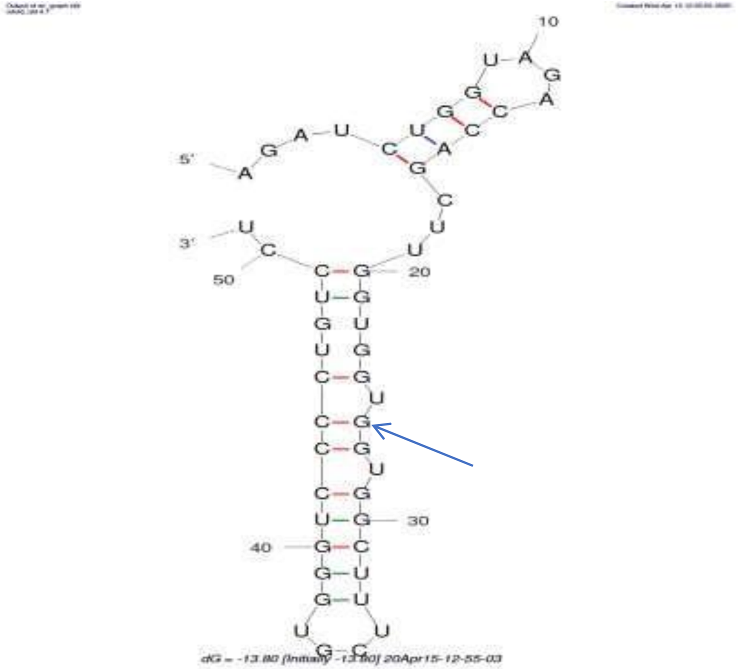

rs756623224

G

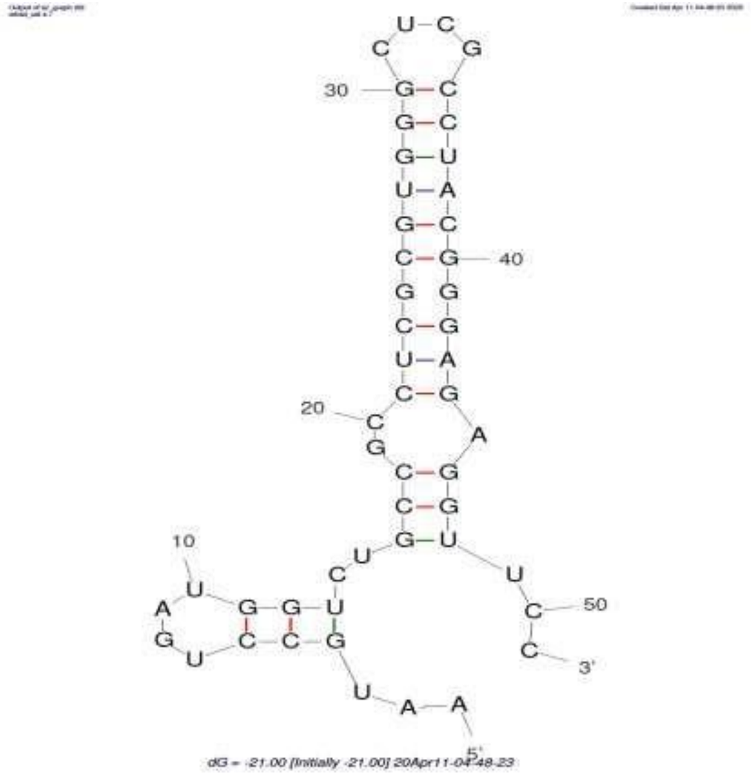

A

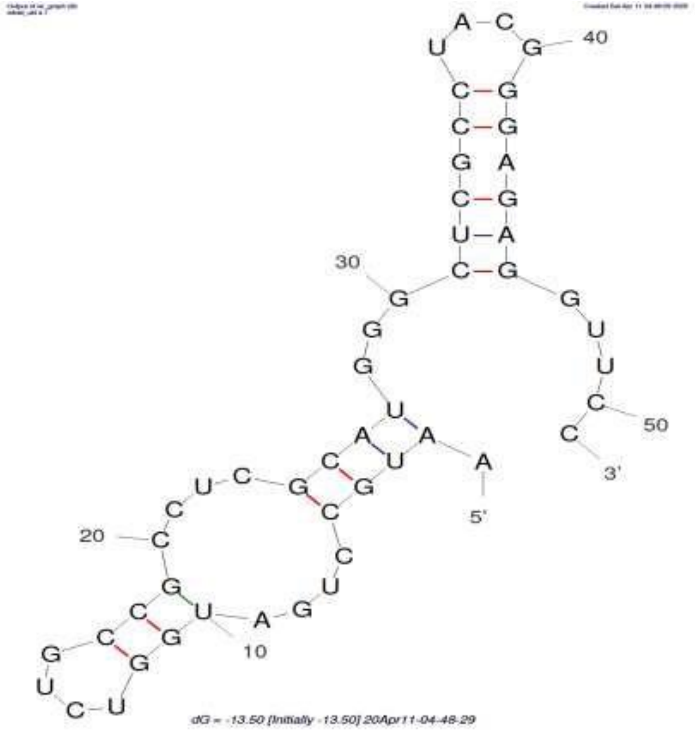

rs779773075

G

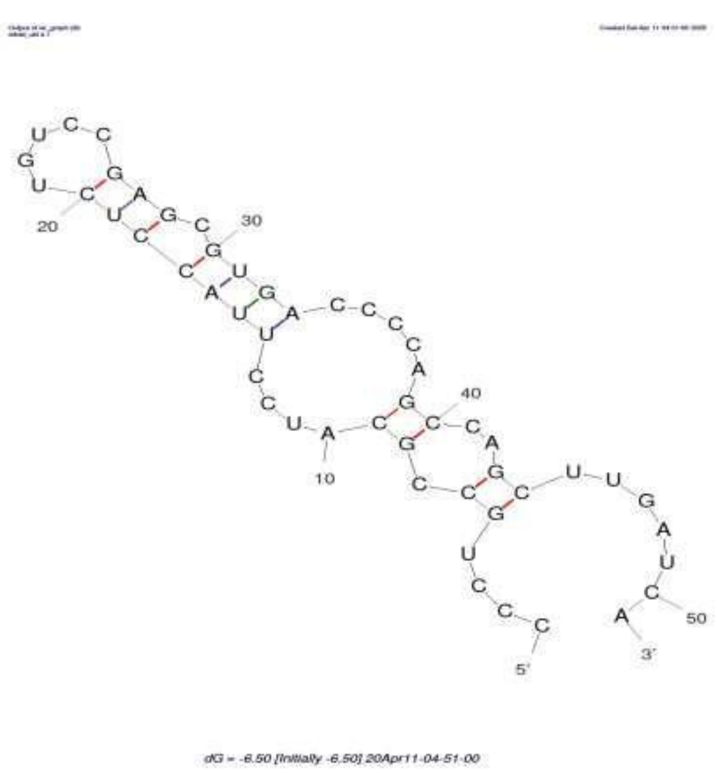

A

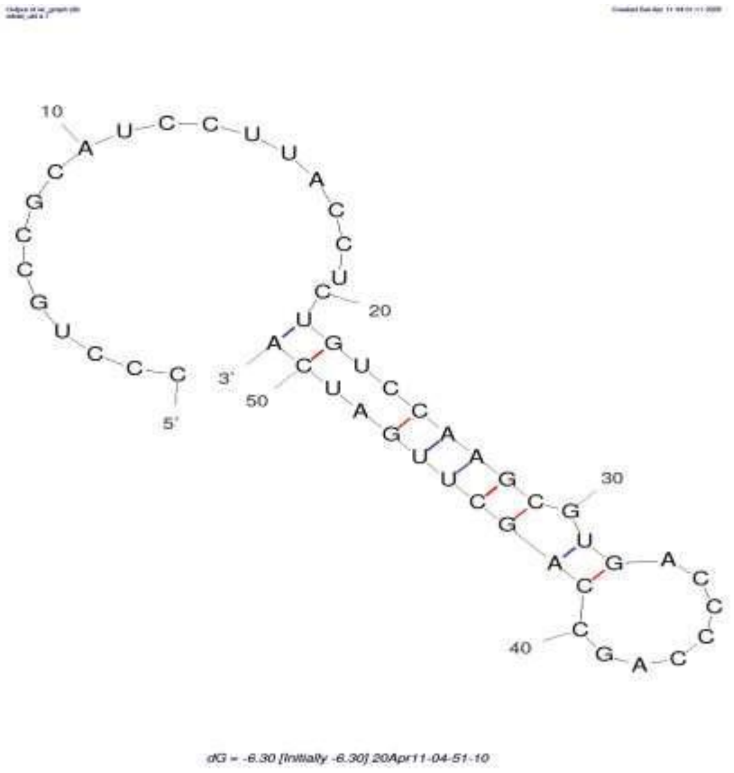

rs721186

A

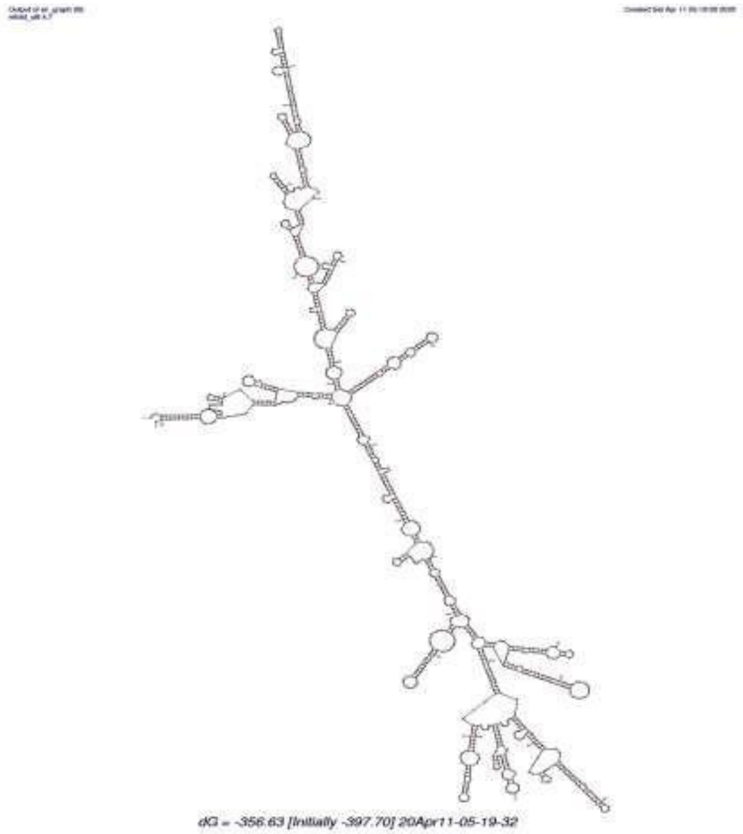
dG = -356.63 [Initially -397.70] 20Apr11-05-19-32

G

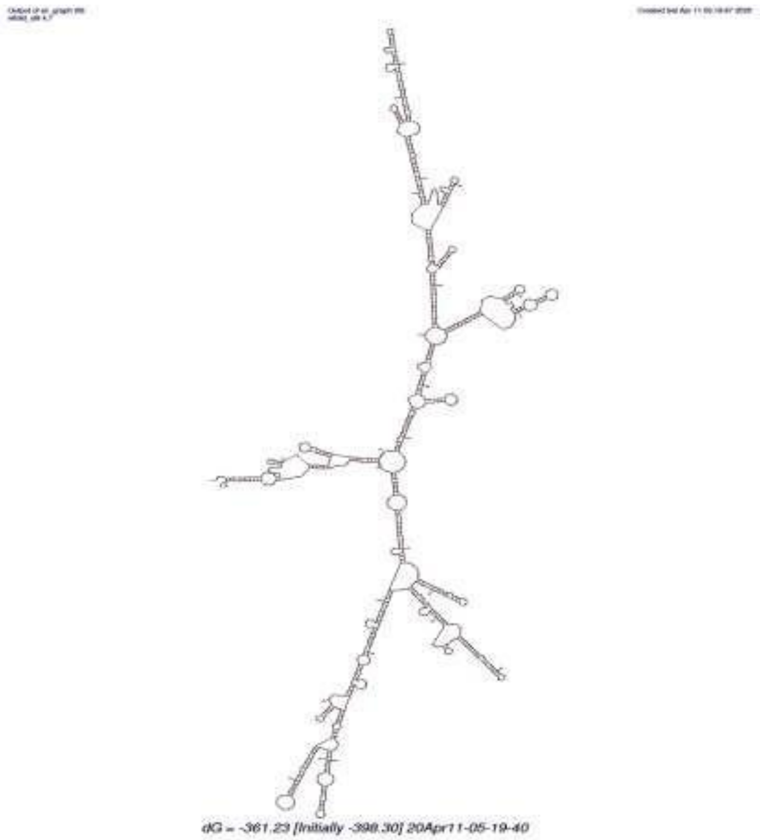
dG = -361.23 [Initially -398.30] 20Apr11-05-19-40

rs765260449

C

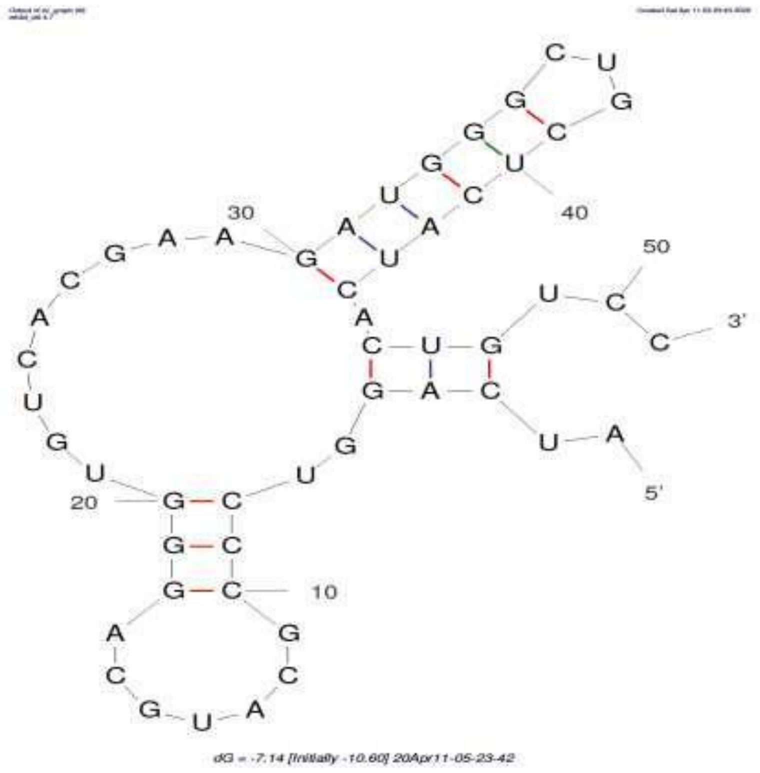

T

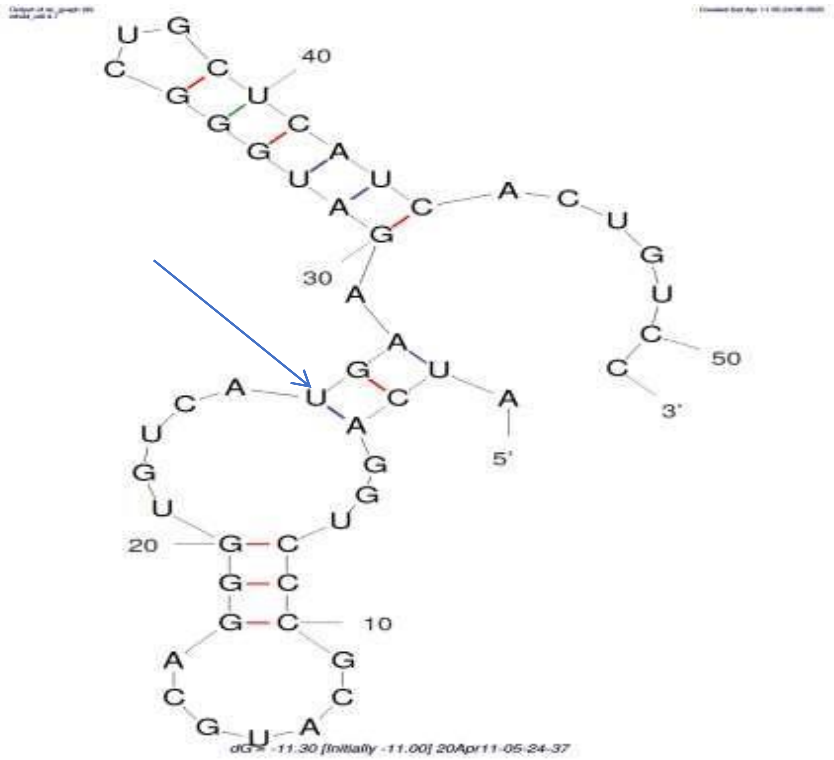

rs2228613

C

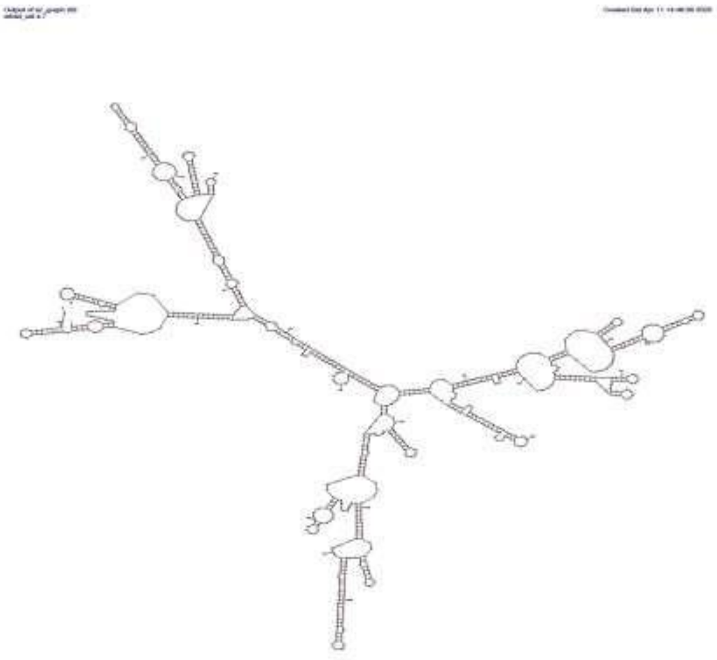

dG = -258.40 [Initially -293.30] 20Apr11-14-48-34

A

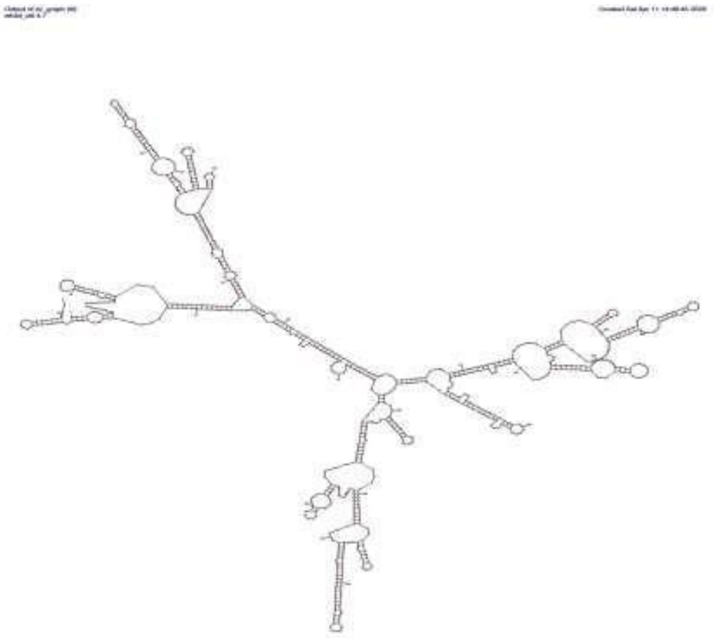

dG = -260.60 [Initially -292.40] 20Apr11-14-48-41

rs372722081

G

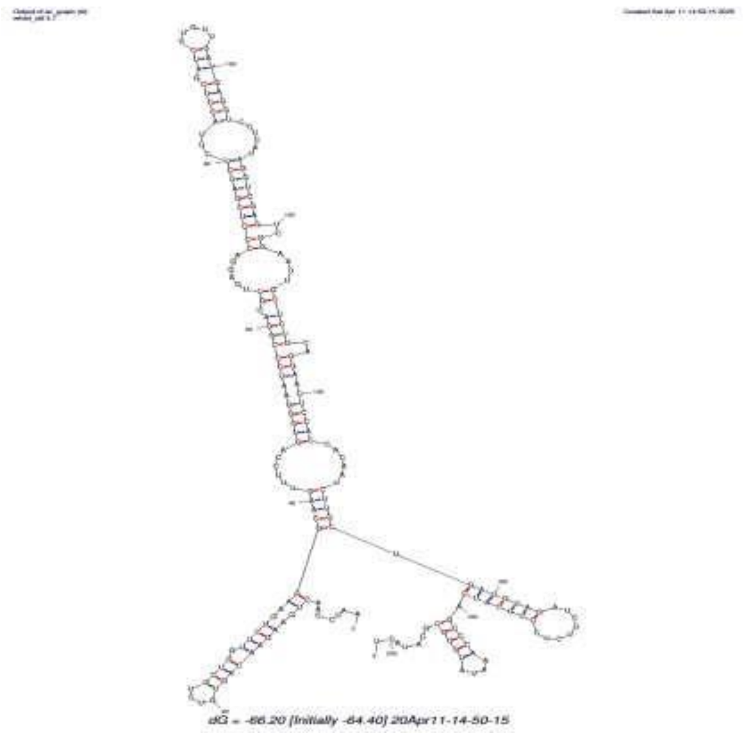

A

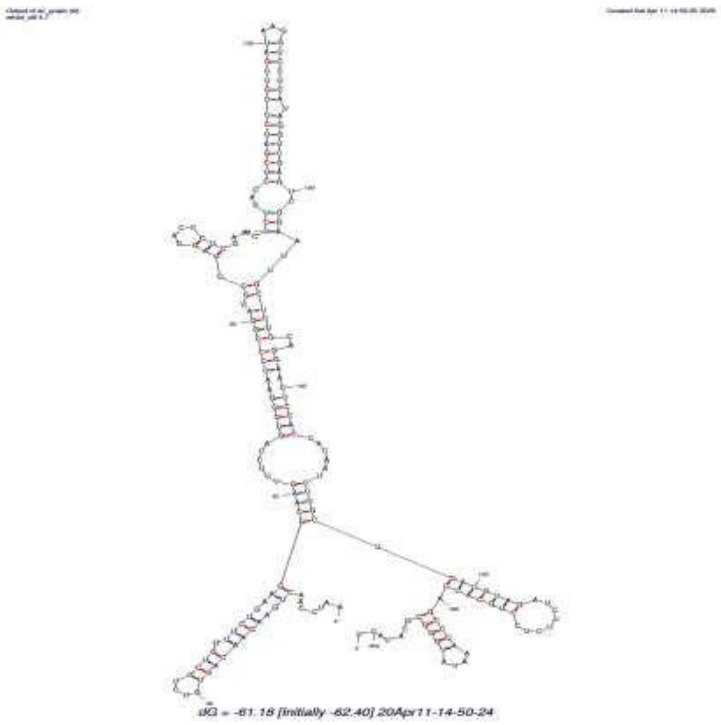

rs1348242844

G

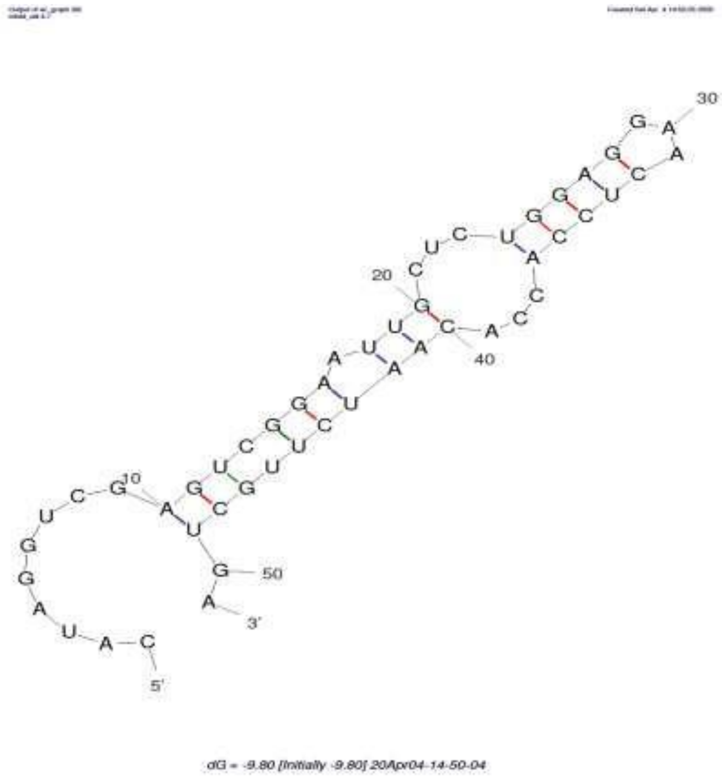

A

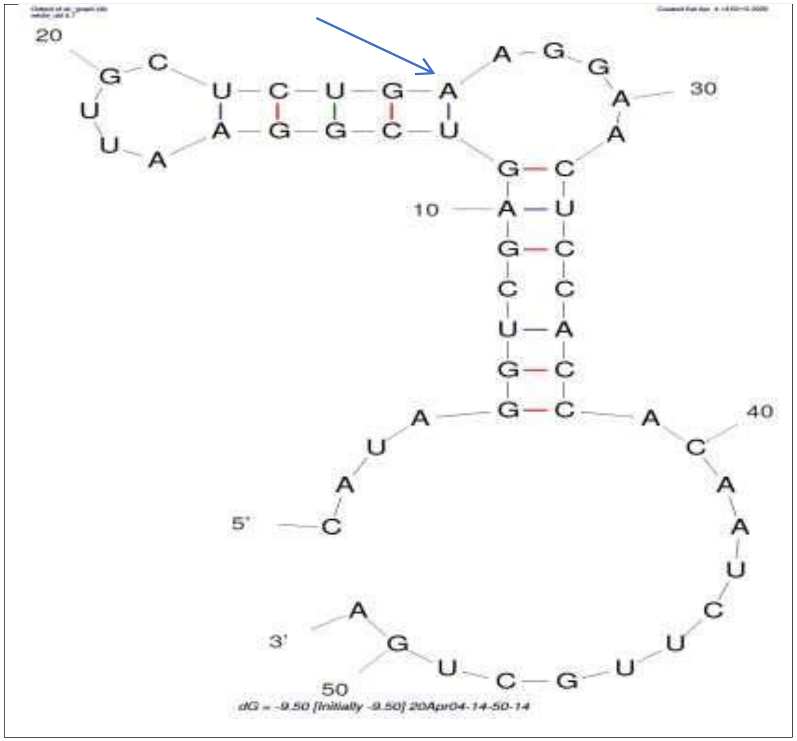

rs2228611

A

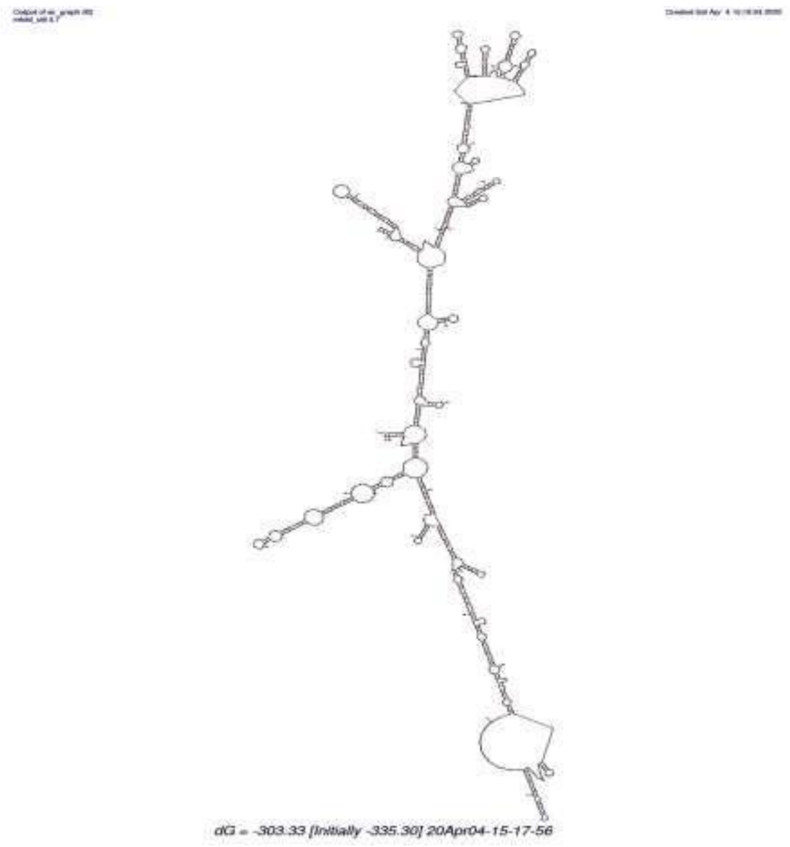

G

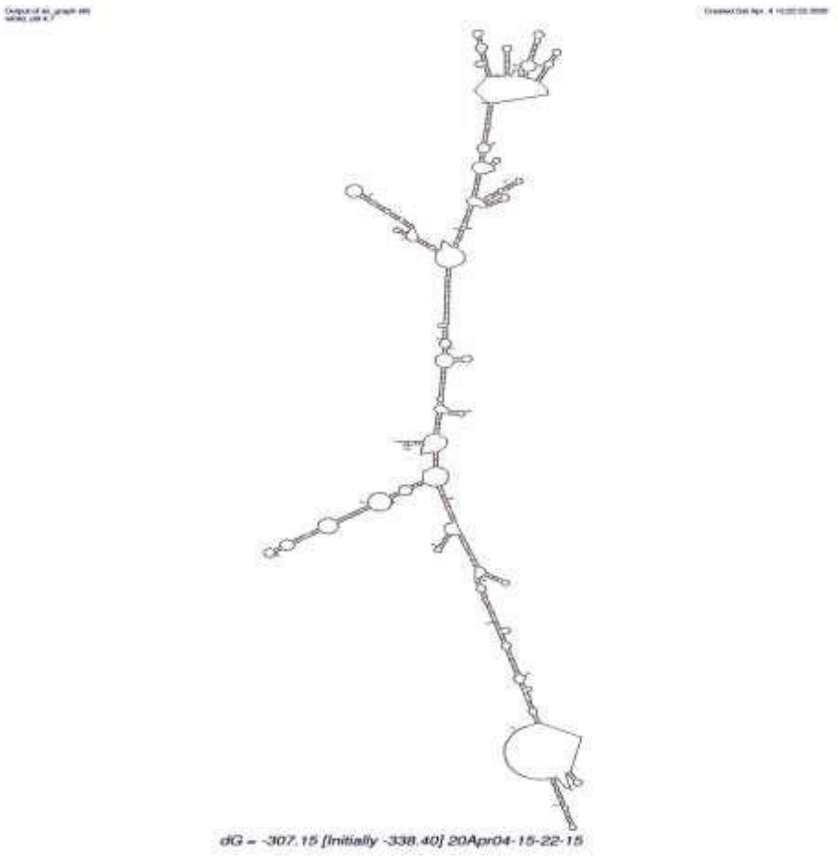

rs1331327607

A

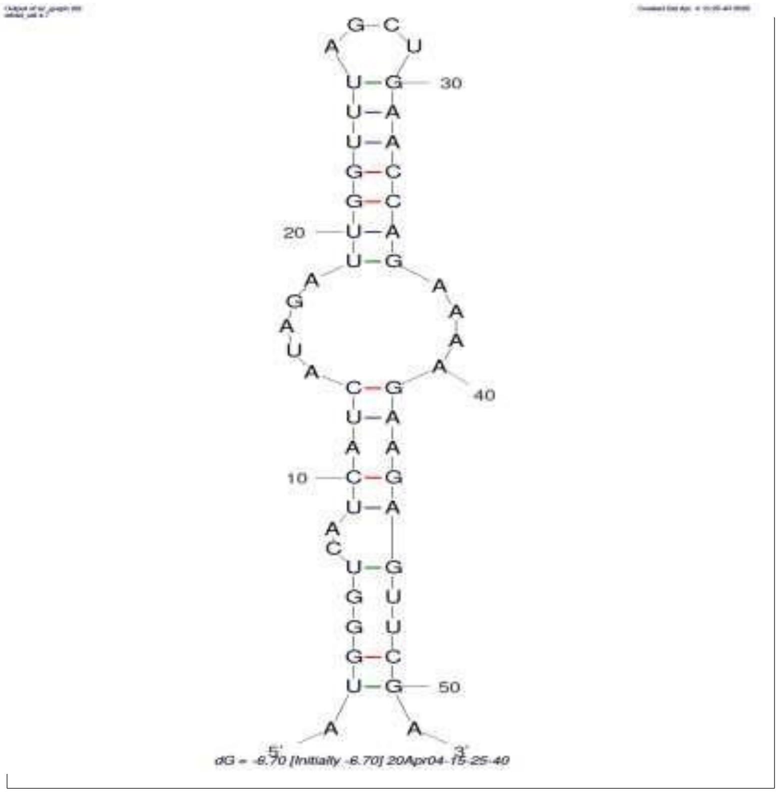

G

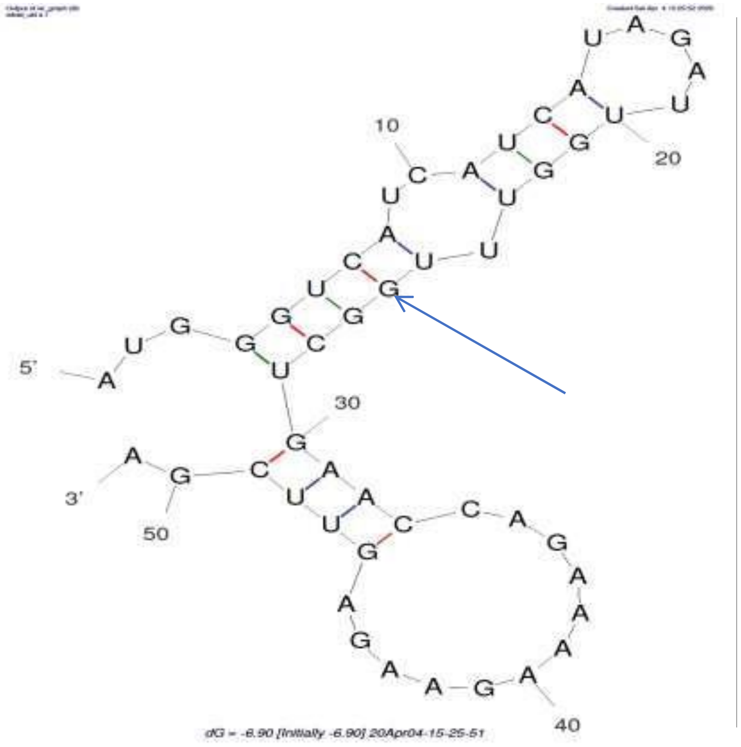

rs201487376

C

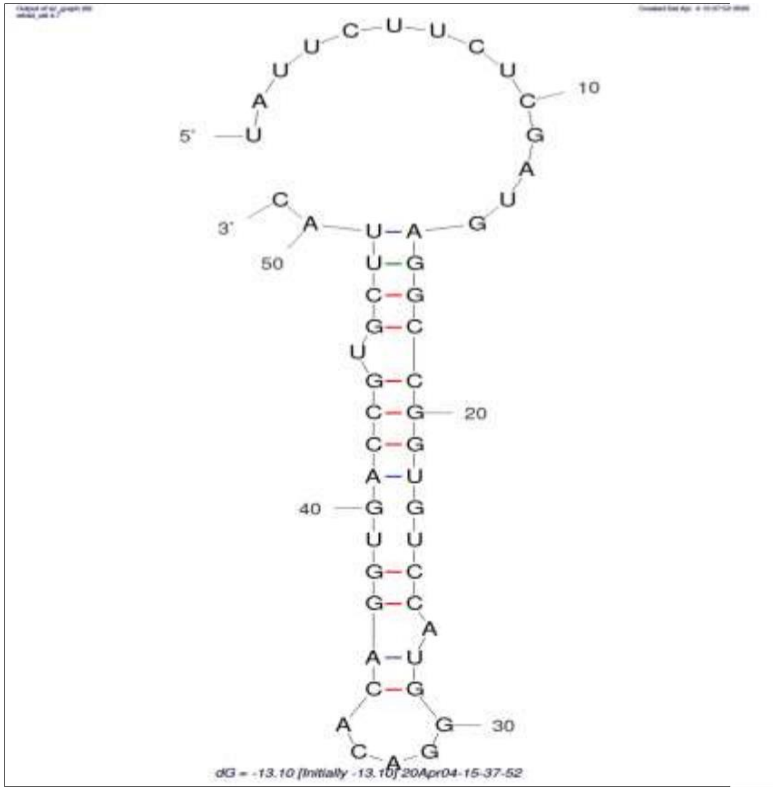

A

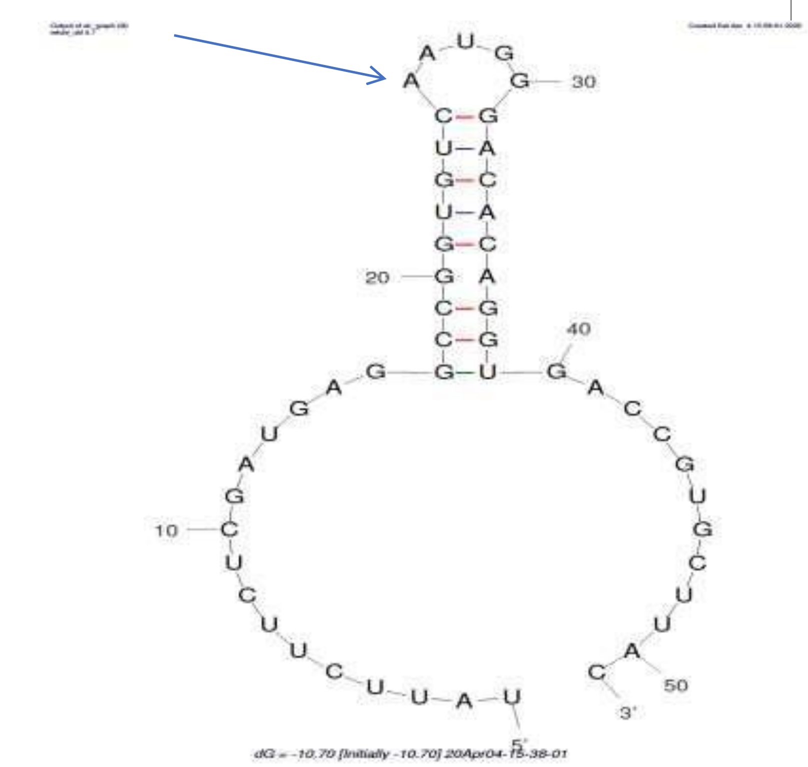

rs1269449523

C

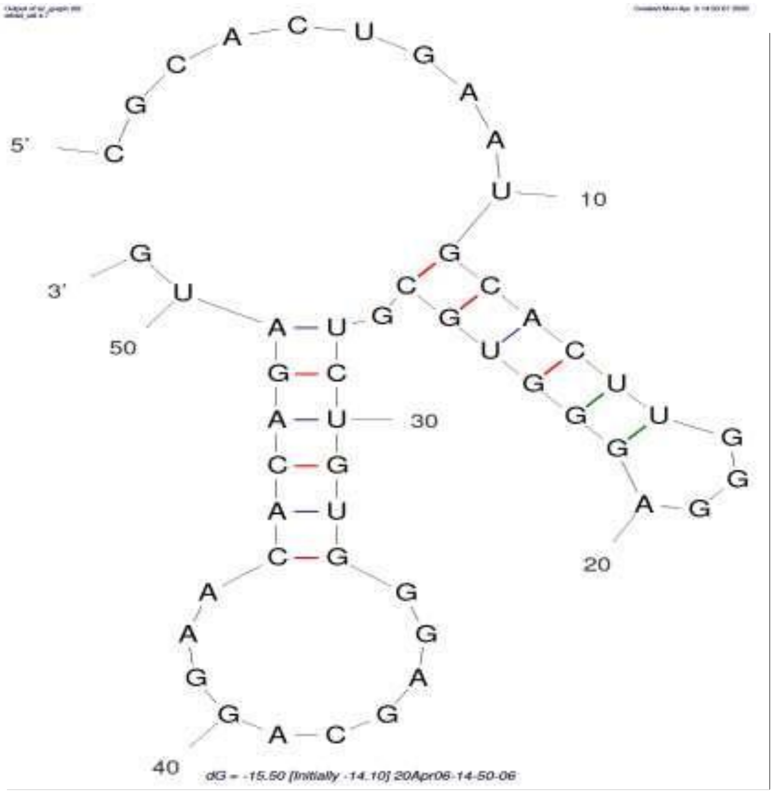

G

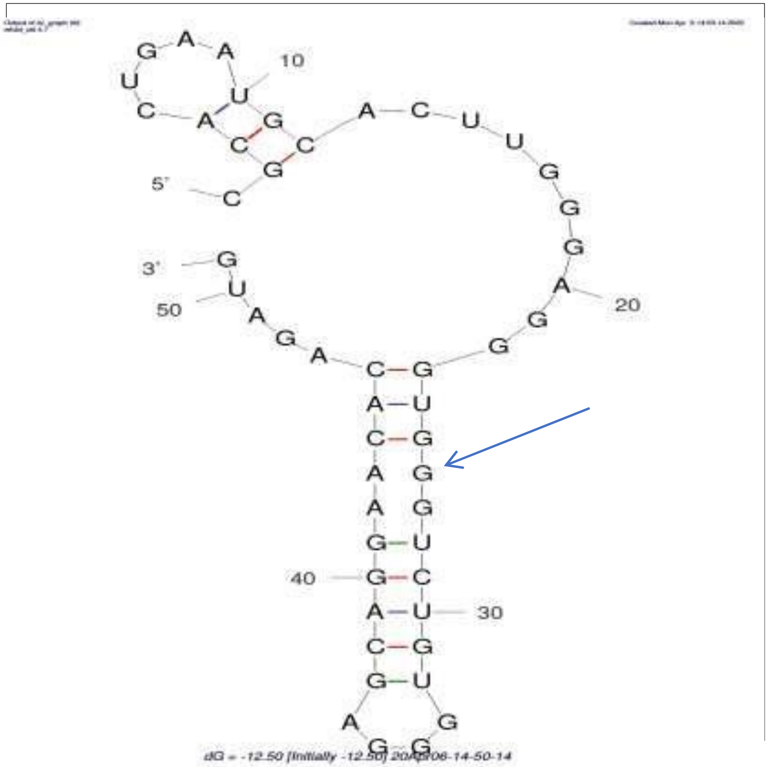

rs1483440897

G

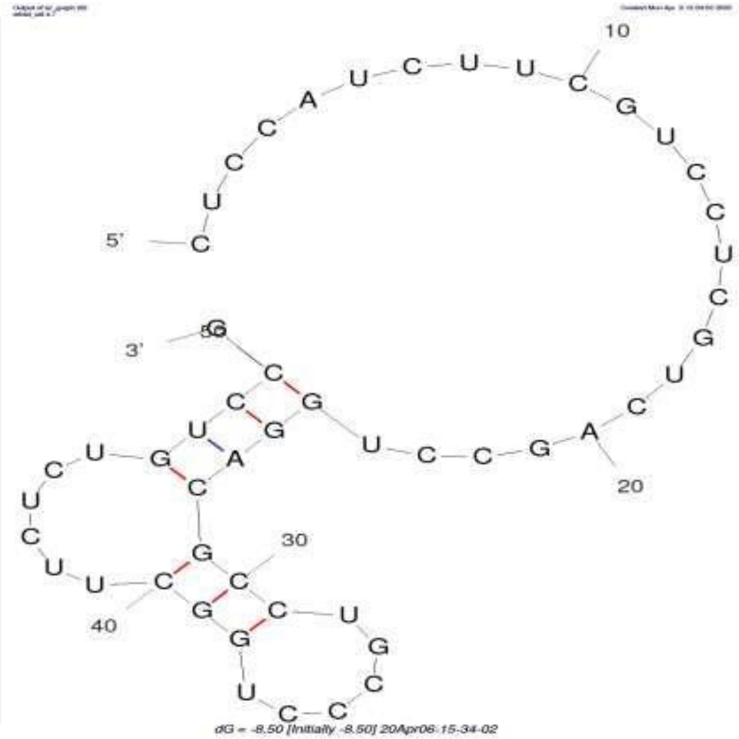

T

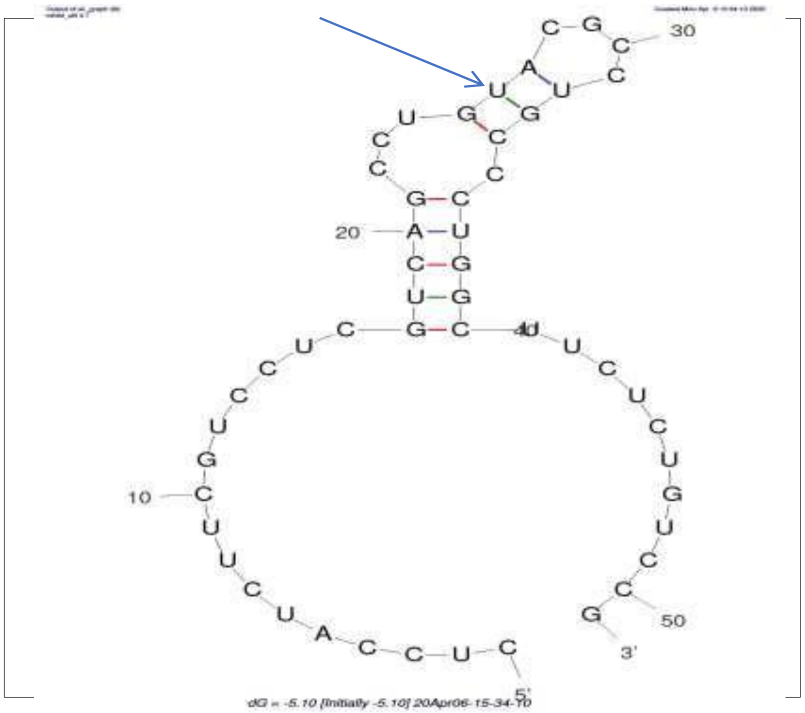

rs777578400

A

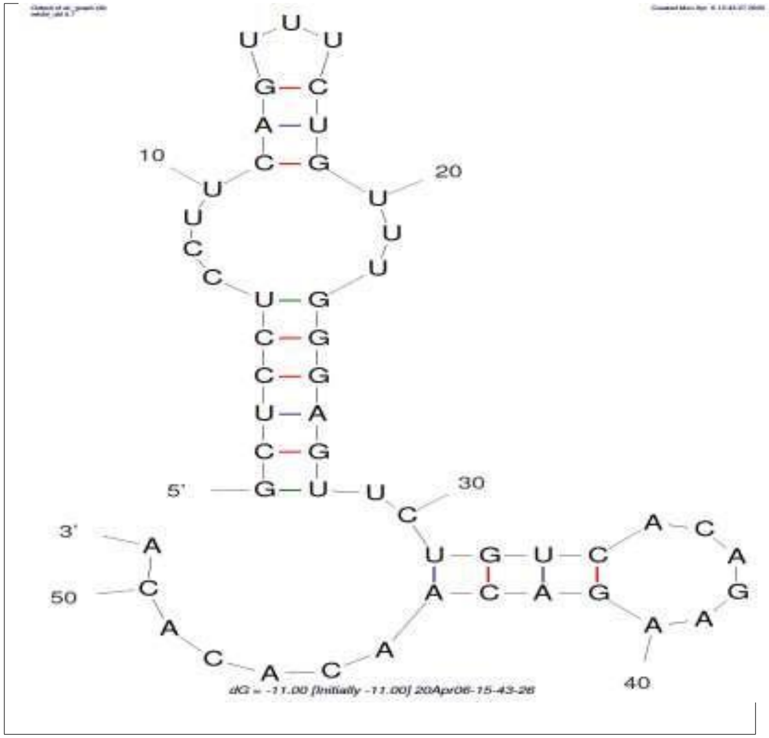

G

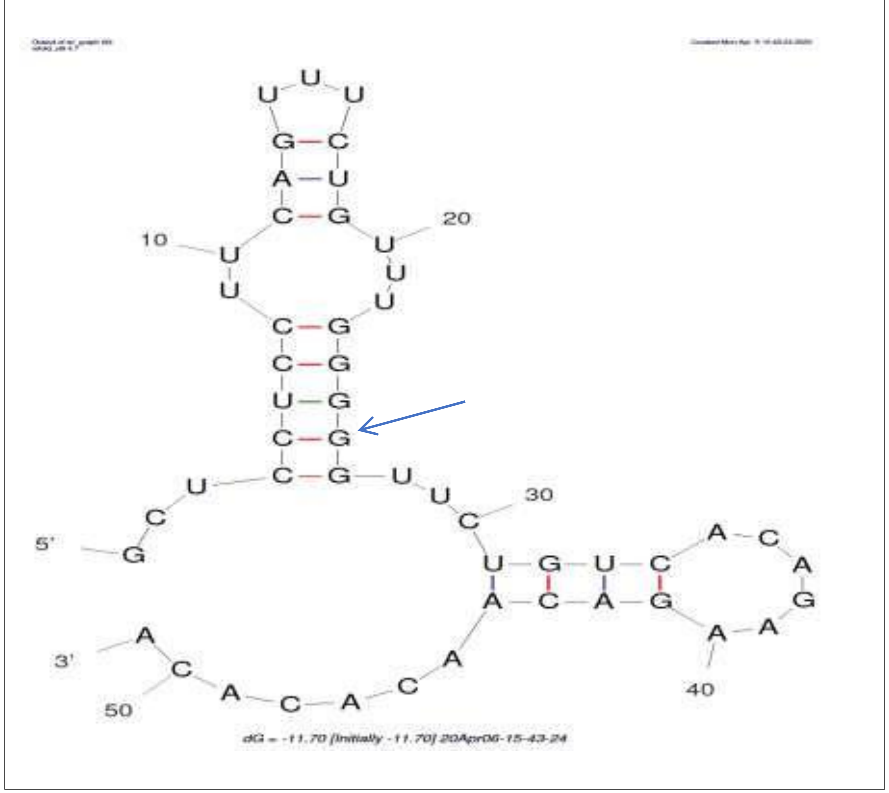

rs1393946866

T

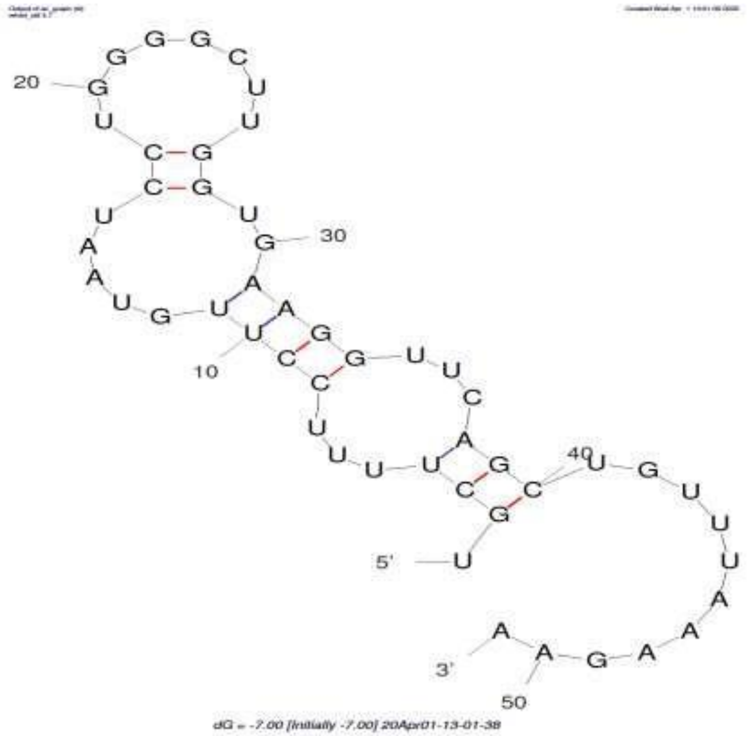

G

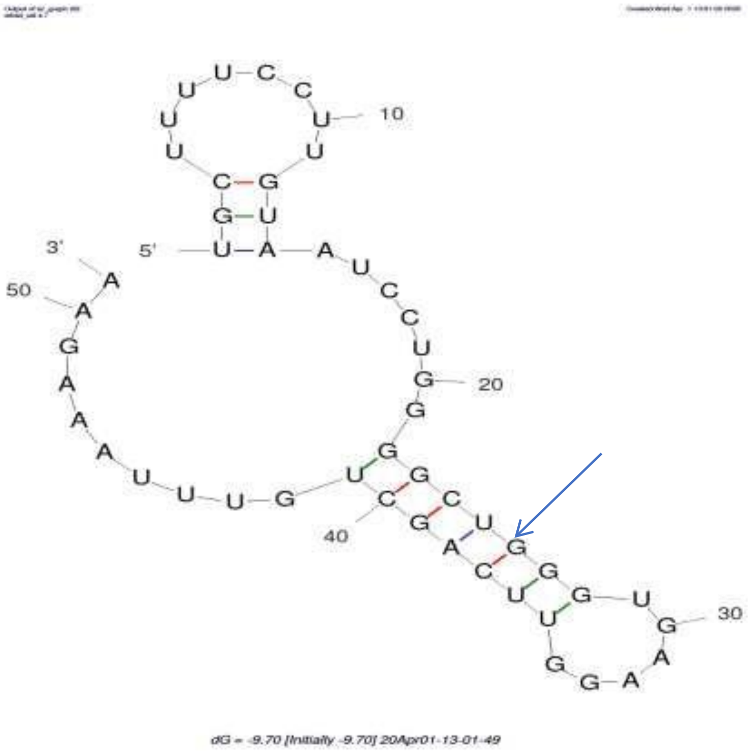

rs771543838

C

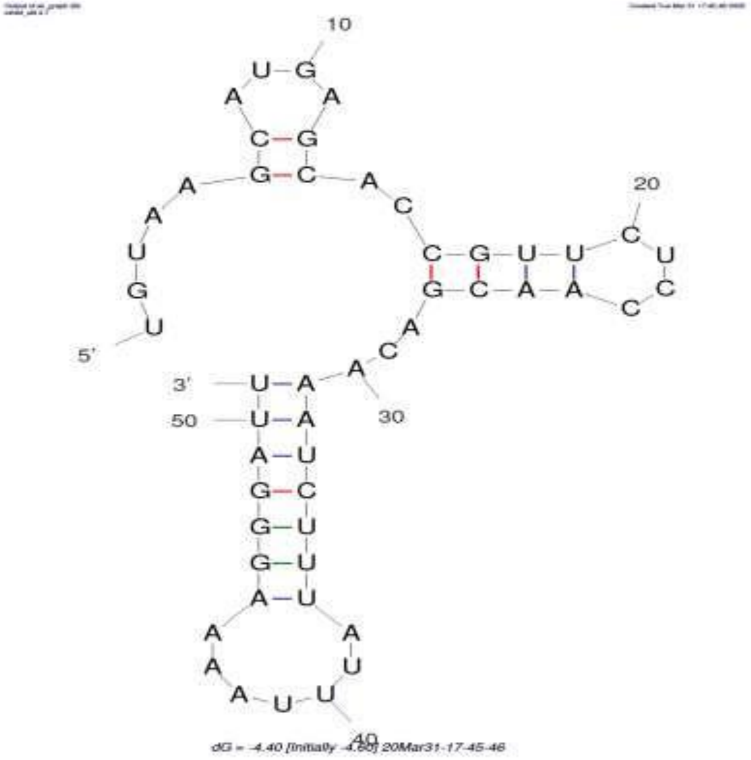

G

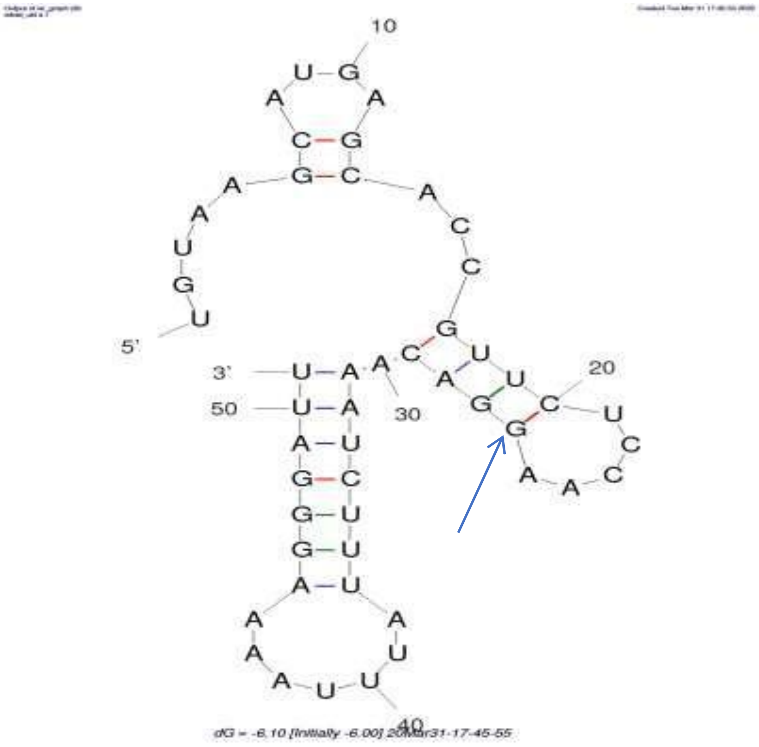

rs1233113560

C

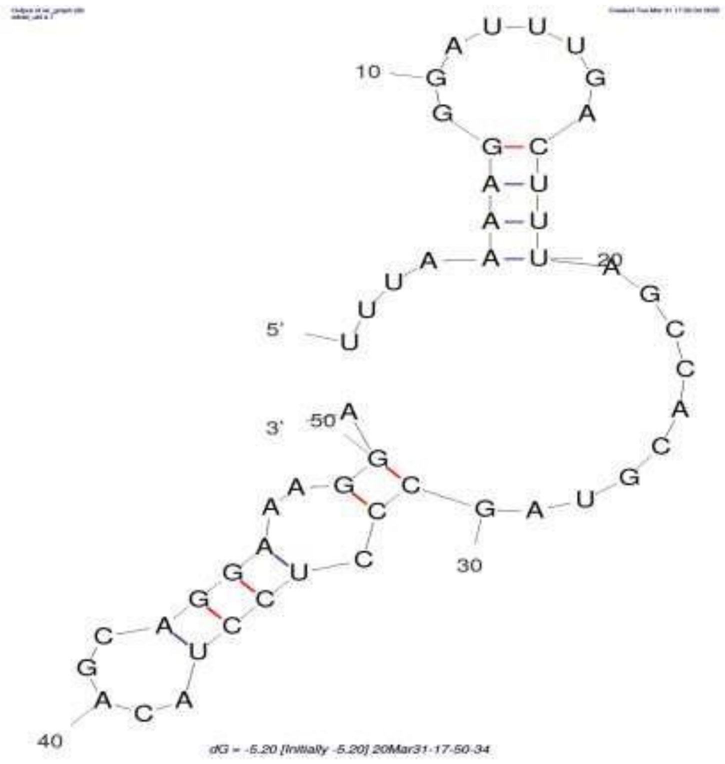

T

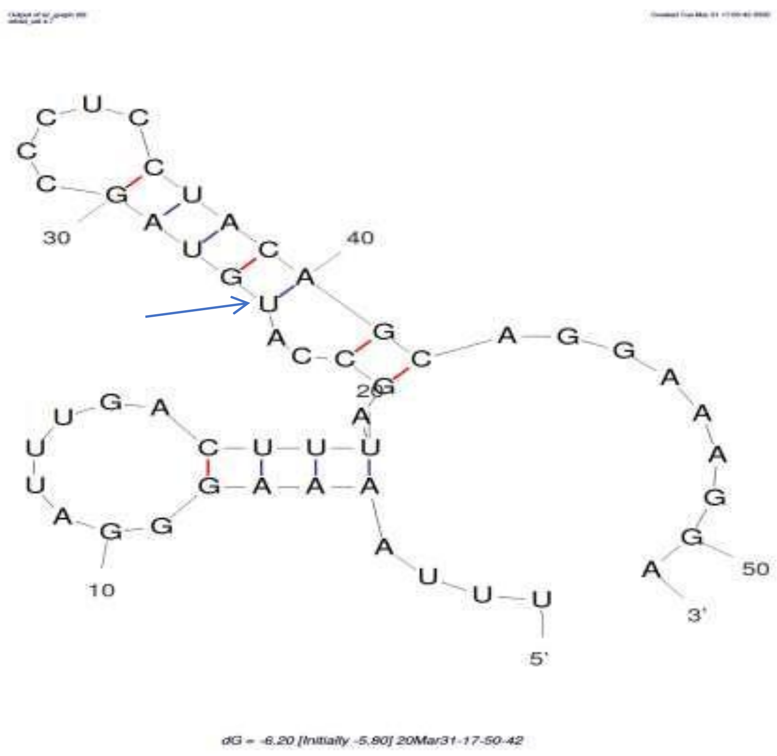

rs770898949

A

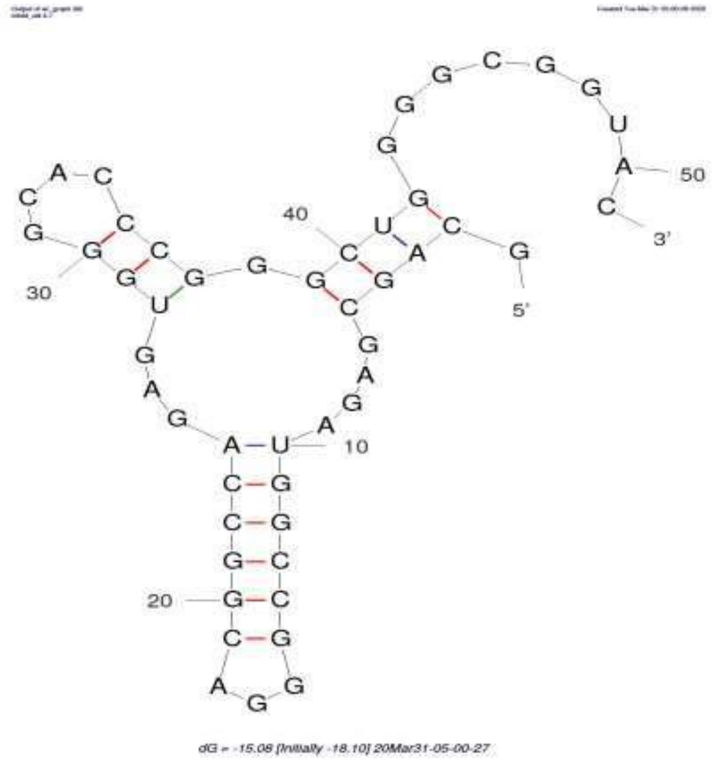

G

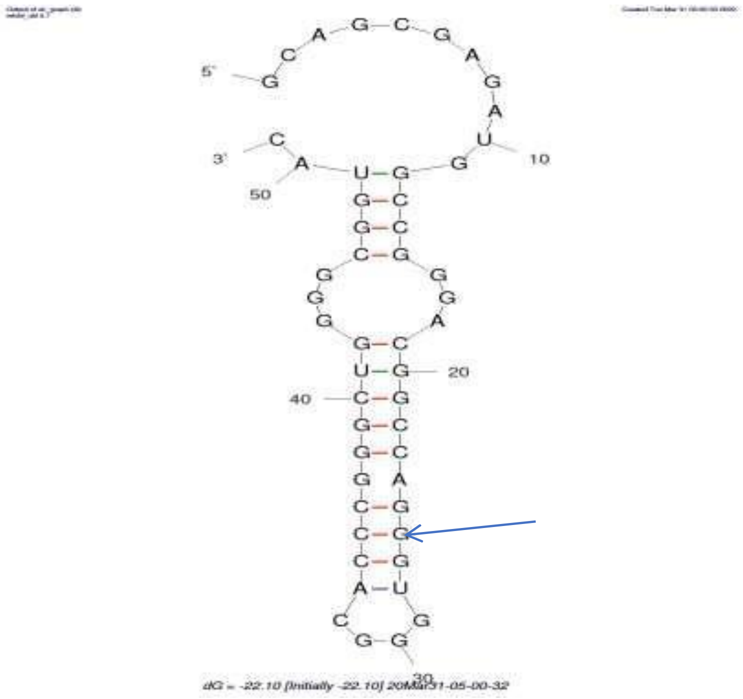

rs577069147

G

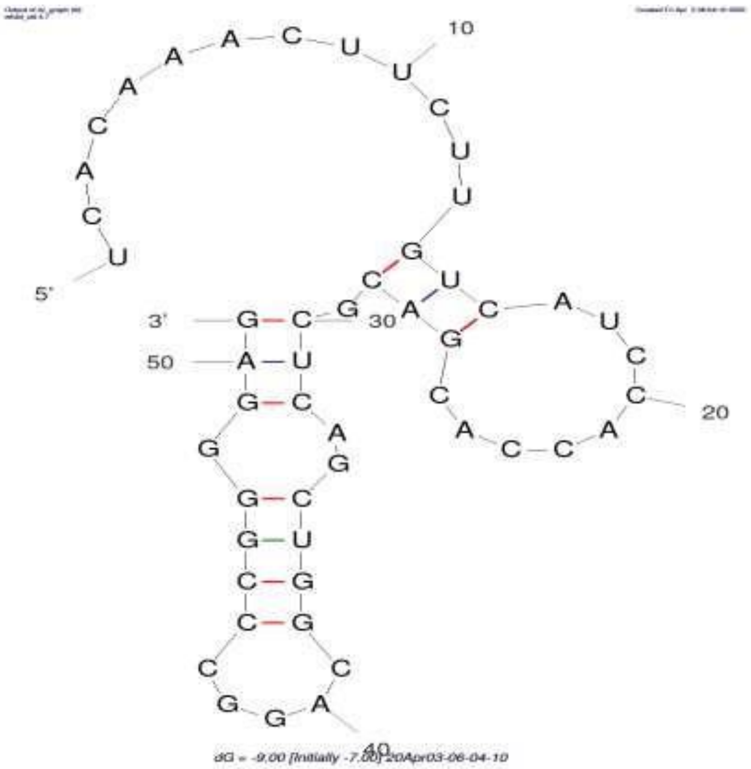

A

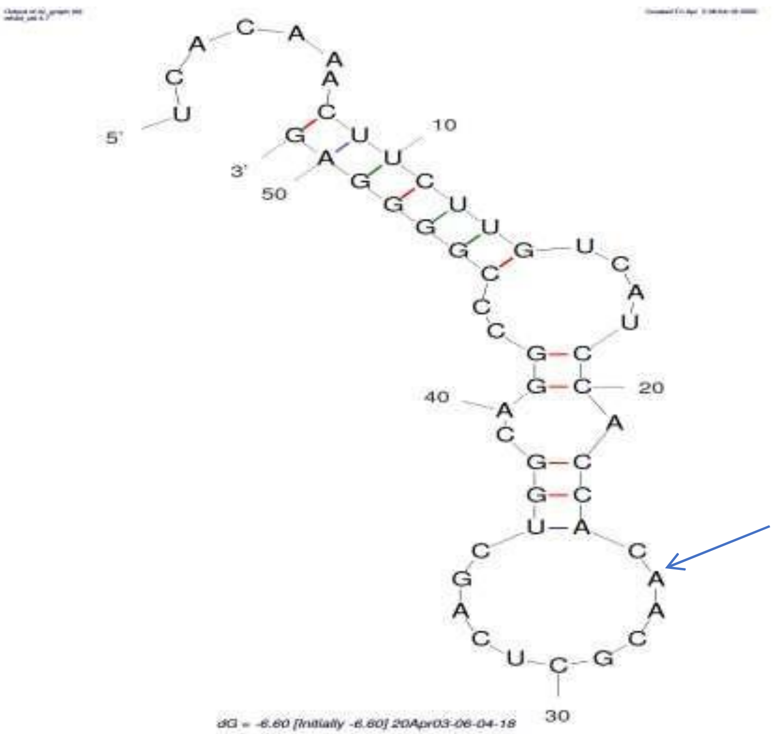

rs760372702

C

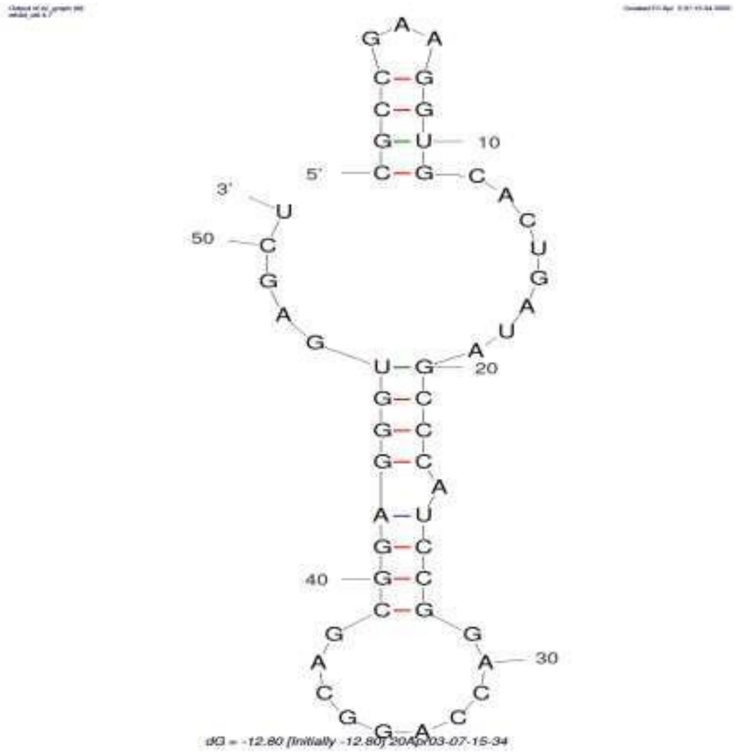

T

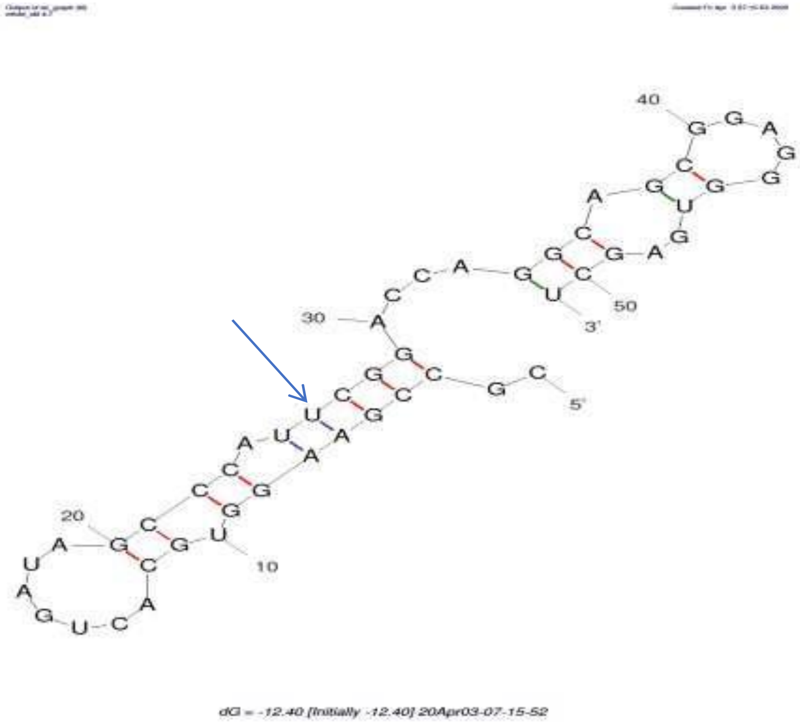

rs770372702

C

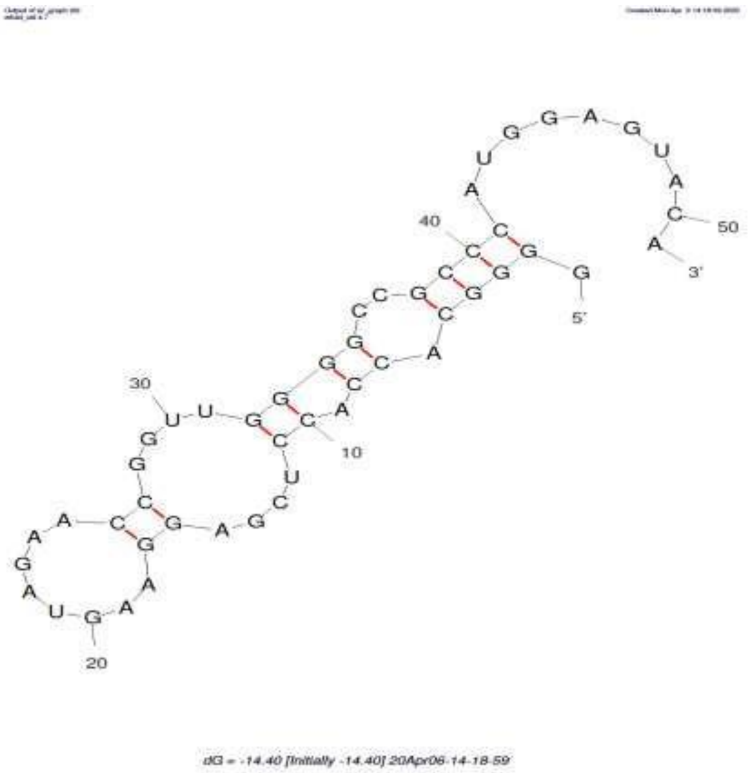

T

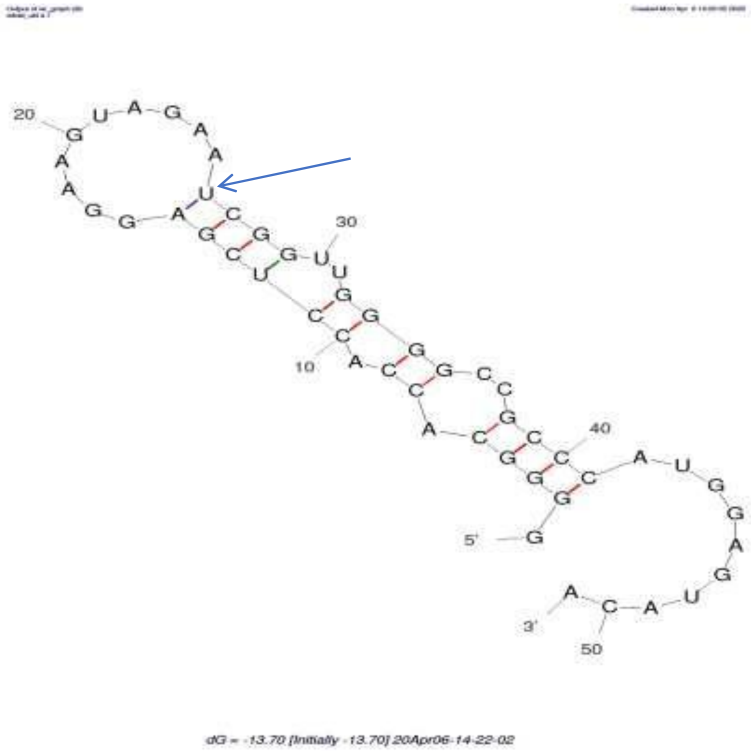

Analysis of DNMT1 Gene Variants in Progression of Neural Tube Defects-an *insilico* to *invitro* approach

Susanta Sadhukhan[1†], Nirvika Paul[1†], Sudakshina Ghosh[2], Dinesh Munian[3], Kausik Ganguly[4], Krishnendu Ghosh[1], Mainak Sengupta[4], Madhusudan Das[1,*]

[1]Department of Zoology, University of Calcutta, 35 Ballygunge Circular Road,Kolkata-700019,India[2]
Department of Zoology, Vidyasagar College for Women, 39 Sankar Ghosh Lane, Kolkata-700006, India[3]
Department of Neonatology, Institute of Postgraduate Medical Education & Research, 244 AcharyaJagadishChandraBose Road,Kolkata,700020,India
[4]Department of Genetics, University of Calcutta, 35 Ballygunge Circular Road, Kolkata-700019,India
[*]Corresponding author at the Dept. of Zoology, University of Calcutta: madhuzoo@yahoo.com /mdzoo@caluniv.ac.in (mail id)
†Authors contributed equally

Supplementary Figure 2: Results procured after STRING analyses. The interactome shows DNMT1 and its targets (as predicted after rSNPBase analyses)HIST1H3H, EIF3G, MRPL4, EEF2 to reside within the same interactome having functional data in support of their interactions (red connecting lines, see figure legends).

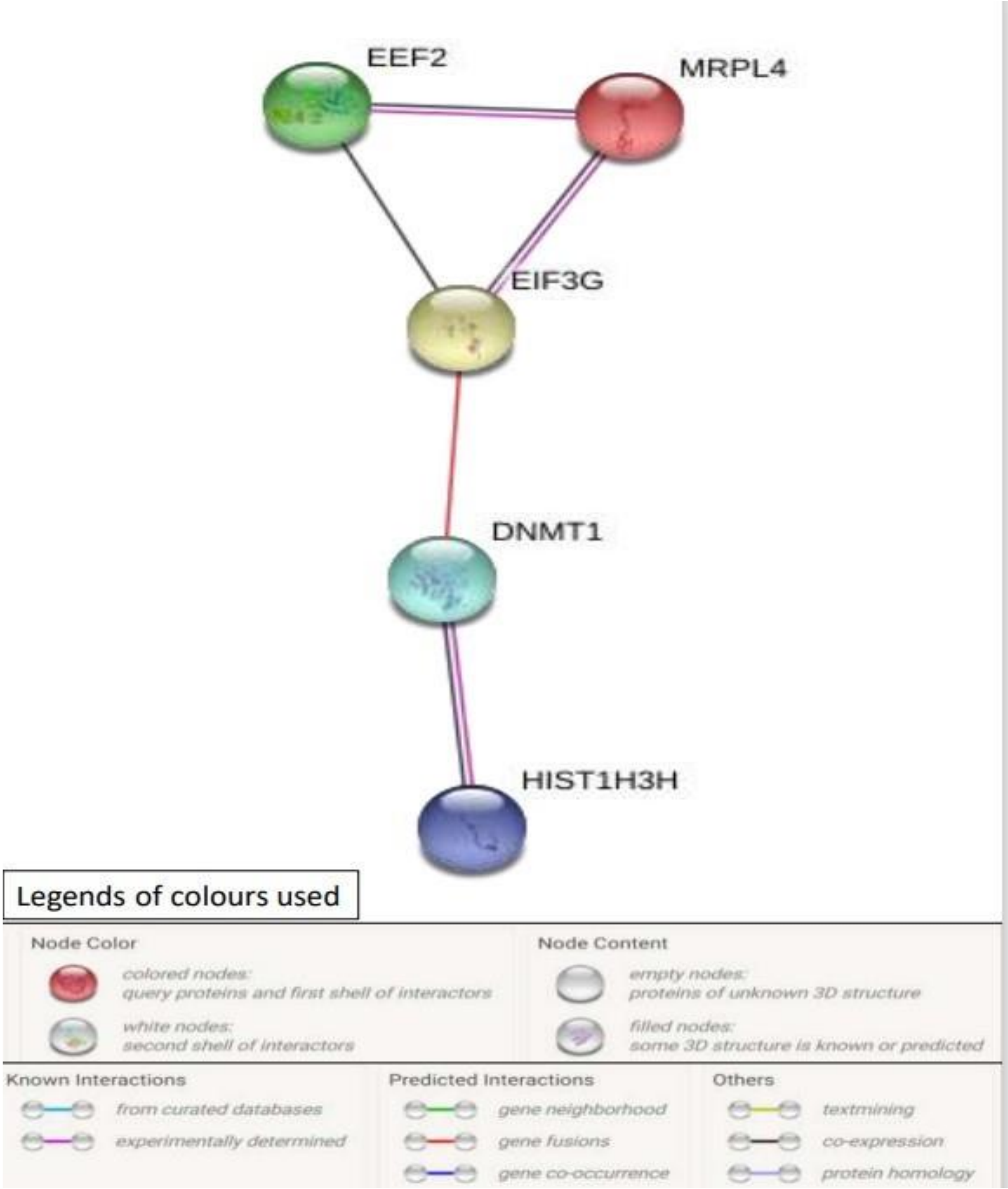
EEF2
MRPL4
EIF3G
DNMT1
HIST1H3H
Legends of colours used
Node Color
colored nodes:
query proteins and first shell of interactors
white nodes:
second shell of interactors
Node Content
empty nodes:
proteins of unknown 3D structure
filled nodes:
some 3D structure is known or predicted
Known Interactions
from curated databases
experimentally determined
Predicted Interactions
gene neighborhood
gene fusions
gene co-occurrence
Others
textmining
co-expression
protein homology

Analysis of DNMT1 Gene Variants in Progression of Neural Tube Defects-an *insilico* to *invitro* Approach

Susanta Sadhukhan[1†], Nirvika Paul[1†], Sudakshina Ghosh[2], Dinesh Munian[3], Kausik Ganguly[4], Krishnendu Ghosh[1], Mainak Sengupta[4], Madhusudan Das[1,*]

[1]Department of Zoology, University of Calcutta, 35 Ballygunge Circular Road, Kolkata-700019, India
[2]Department of Zoology, Vidyasagar College for Women, 39 Sankar Ghosh Lane, Kolkata-700006, India
[3]Department of Neonatology, Institute of Postgraduate Medical Education & Research, 244 Acharya Jagadish Chandra Bose Road, Kolkata, 700020,India
[4]Department of Genetics, University of Calcutta, 35 Ballygunge Circular Road, Kolkata-700019, India
[*]Corresponding author at the Dept. of Zoology, University of Calcutta: madhuzoo@yahoo.com / mdzoo@caluniv.ac.in (mail id)
†Authors contributed equally

Supplementary Figure 3: DNMT1 expression as procured from SCREEN interface of ENCODE browser. Note: DNMT1 expression is maximum in blood, embryo and uterus in decreasing order though our findings showed marked differences between blood samples procured from mothers and their respective newborn babies born with spina bifida (a form of Neural Tube Defect, NTD).

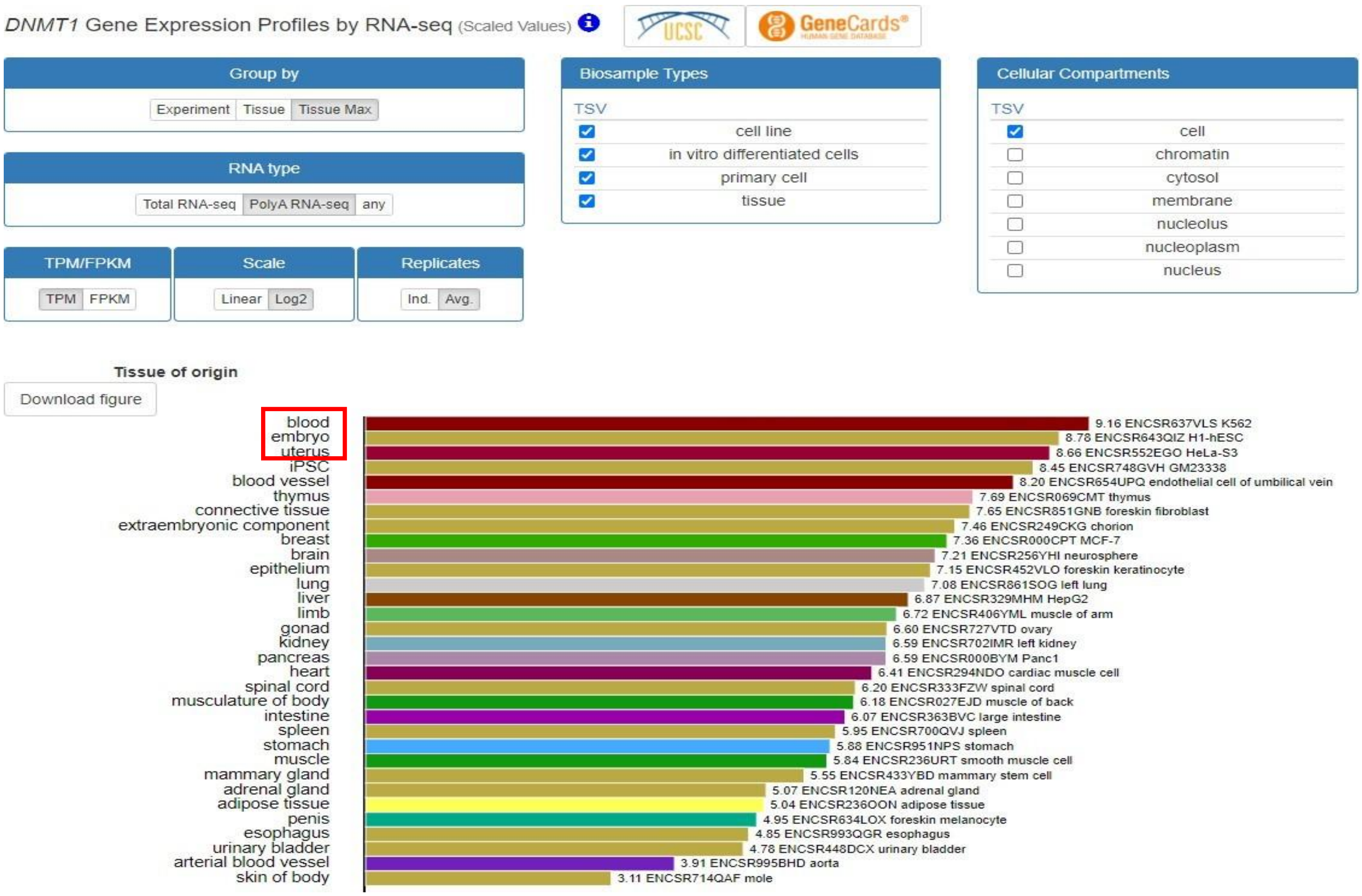
DNMT1 Gene Expression Profiles by RNA-seq (Scaled Values)
UCSC
GeneCards
Group by
Experiment
Tissue
Tissue Max
RNA type
Total RNA-seq
PolyA RNA-seq
any
TPM/FPKM
TPM
FPKM
Scale
Linear
Log2
Replicates
Ind.
Avg.
Biosample Types
TSV
cell line
in vitro differentiated cells
primary cell
tissue
Cellular Compartments
TSV
cell
chromatin
cytosol
membrane
nucleolus
nucleoplasm
nucleus
Tissue of origin
Download figure
blood
embryo
uterus
iPSC
blood vessel
thymus
connective tissue
extraembryonic component
breast
brain
epithelium
lung
liver
limb
gonad
kidney
pancreas
heart
spinal cord
musculature of body
intestine
spleen
stomach
muscle
mammary gland
adrenal gland
adipose tissue
penis
esophagus
urinary bladder
arterial blood vessel
skin of body
9.16 ENCSR637VLS K562
8.78 ENCSR643QIZ H1-hESC
8.66 ENCSR552EGO HeLa-S3
8.45 ENCSR748GVH GM23338
8.20 ENCSR654UPQ endothelial cell of umbilical vein
7.69 ENCSR069CMT thymus
7.65 ENCSR851GNB foreskin fibroblast
7.46 ENCSR249CKG chorion
7.36 ENCSR000CPT MCF-7
7.21 ENCSR256YHI neurosphere
7.15 ENCSR452VLO foreskin keratinocyte
7.08 ENCSR861SOG left lung
6.87 ENCSR329MHM HepG2
6.72 ENCSR406YML muscle of arm
6.60 ENCSR727VTD ovary
6.59 ENCSR702IMR left kidney
6.59 ENCSR000BYM Panc1
6.41 ENCSR294NDO cardiac muscle cell
6.20 ENCSR333FZW spinal cord
6.18 ENCSR027EJD muscle of back
6.07 ENCSR363BVC large intestine
5.95 ENCSR700QVJ spleen
5.88 ENCSR951NPS stomach
5.84 ENCSR236URT smooth muscle cell
5.55 ENCSR433YBD mammary stem cell
5.07 ENCSR120NEA adrenal gland
5.04 ENCSR236OON adipose tissue
4.95 ENCSR634LOX foreskin melanocyte
4.85 ENCSR993QGR esophagus
4.78 ENCSR448DCX urinary bladder
3.91 ENCSR995BHD aorta
3.11 ENCSR714QAF mole

Analysis of DNMT1 Gene Variants in Progression of Neural Tube Defects- an *insilico* to *invitro* approach

Susanta Sadhukhan[1,†], Nirvika Paul[1,†], Sudakshina Ghosh[2], Dinesh Munian[3], Kausik Ganguly[4], Krishnendu Ghosh[1], Mainak Sengupta[4], Madhusudan Das[1,*]

[1]Department of Zoology, University of Calcutta, 35 Ballygunge Circular Road, Kolkata-700019, India
[2]Department of Zoology, Vidyasagar College for Women, 39 Sankar Ghosh Lane, Kolkata-700006, India
[3]Department of Neonatology, Institute of Postgraduate Medical Education & Research, 244 Acharya Jagadish Chandra Bose Road, Kolkata, 700020, India
[4]Department of Genetics, University of Calcutta, 35 Ballygunge Circular Road, Kolkata-700019, India
[*]Corresponding author at the Dept. of Zoology, University of Calcutta: madhuzoo@yahoo.com/ mdzoo@caluniv.ac.in (mail id)
†Authors contributed equally

Supplementary figure 4:

Summarized results of DeepView - Swiss-PdbViewer analysis. Out of 17 SNVs, 6 non-syn SNVs [S1556F (rs1388362405), R1555C (rs1461695373), G1449R (rs770571074), R1261W (rs1052868434), G806R (rs183555527), D785H (rs1244845928)] were found to have most potent and their roles can further be assessed using functional experimentations (DeepView Swiss-PdbViewer).

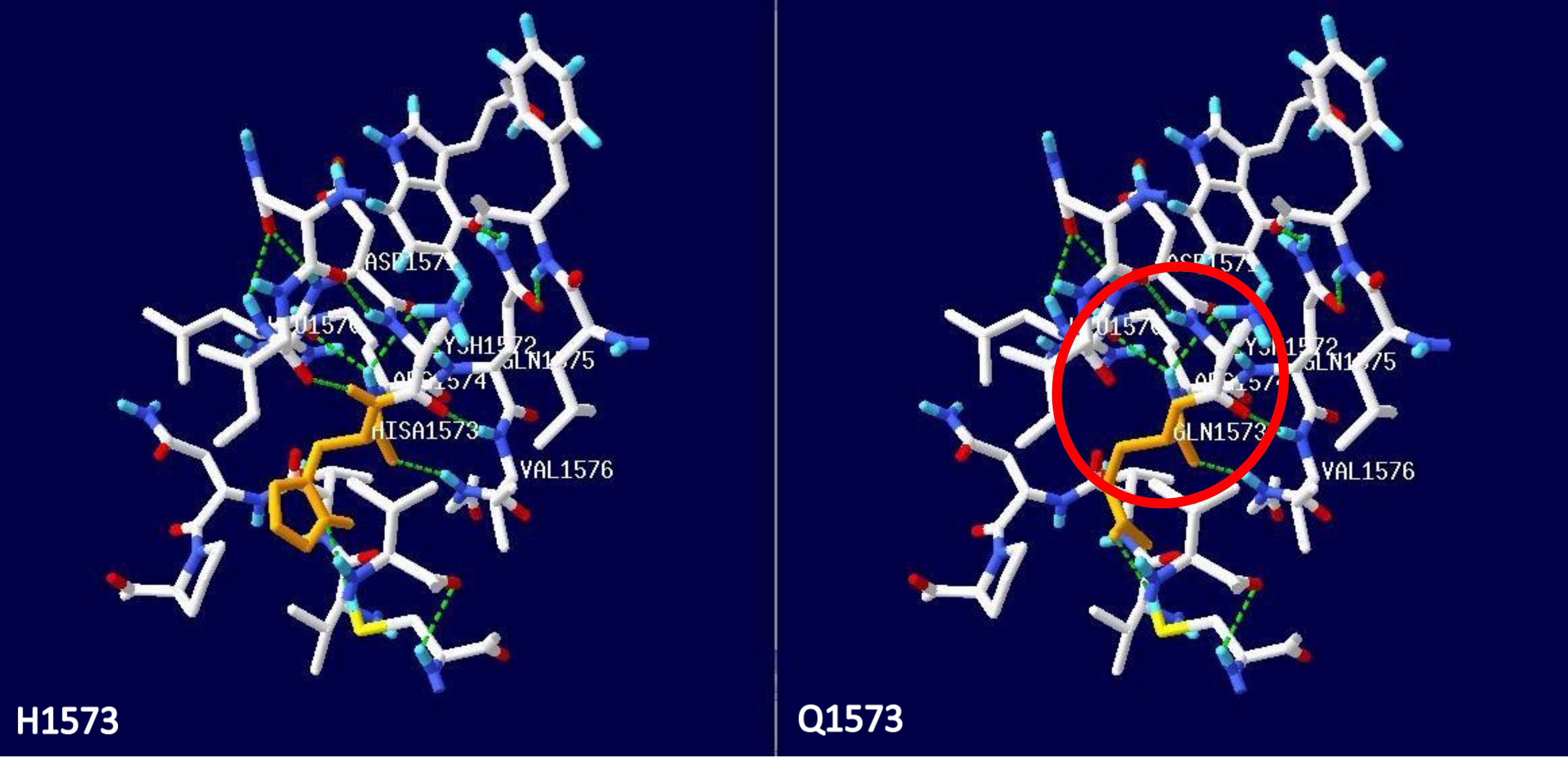

Result: Upon computing hydrogen bonds: Q1573 lost hydrogen bond compared to H1573

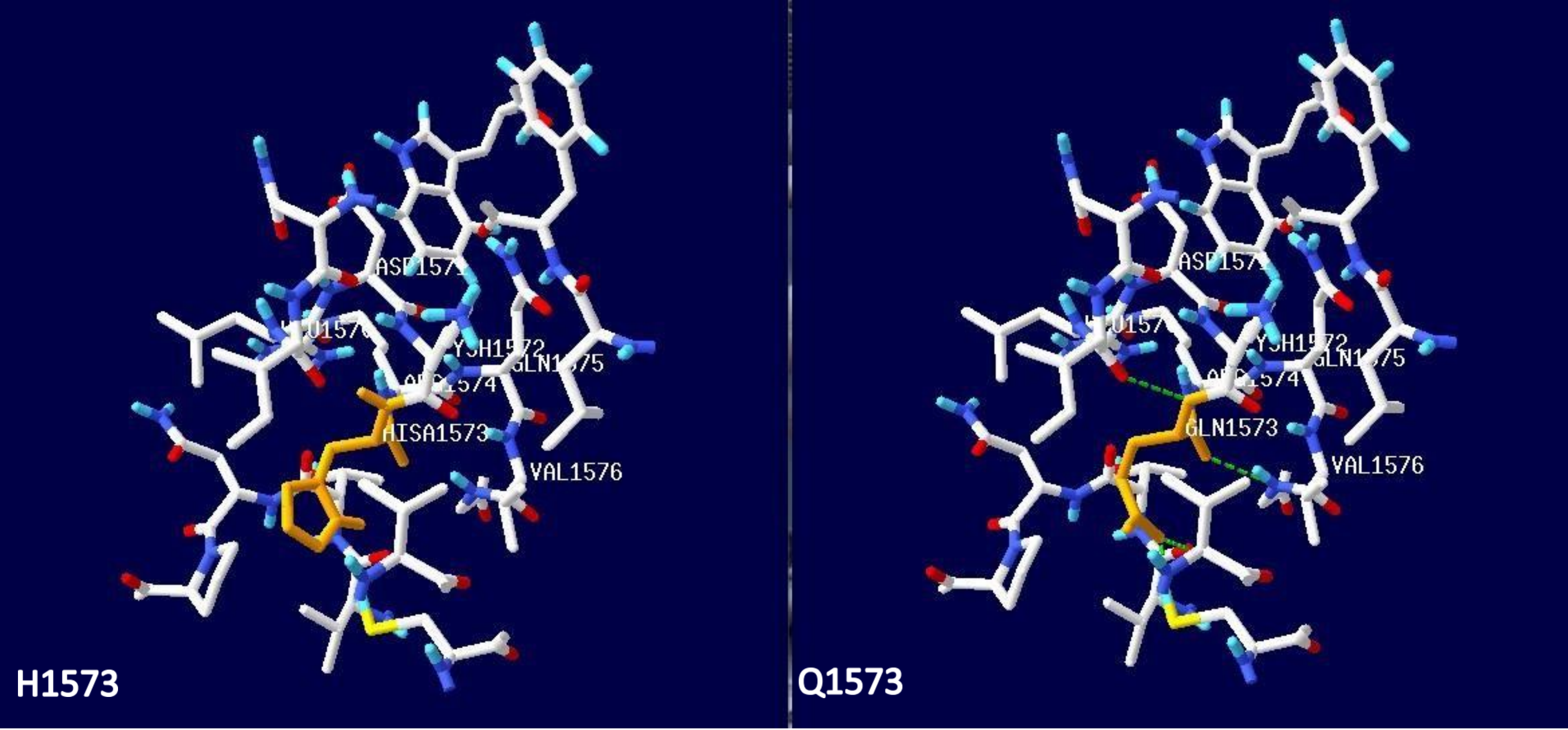

Result: Upon computing "mutation" effect:
Q1573 gained 4 hydrogen bonds that were absent in H1573

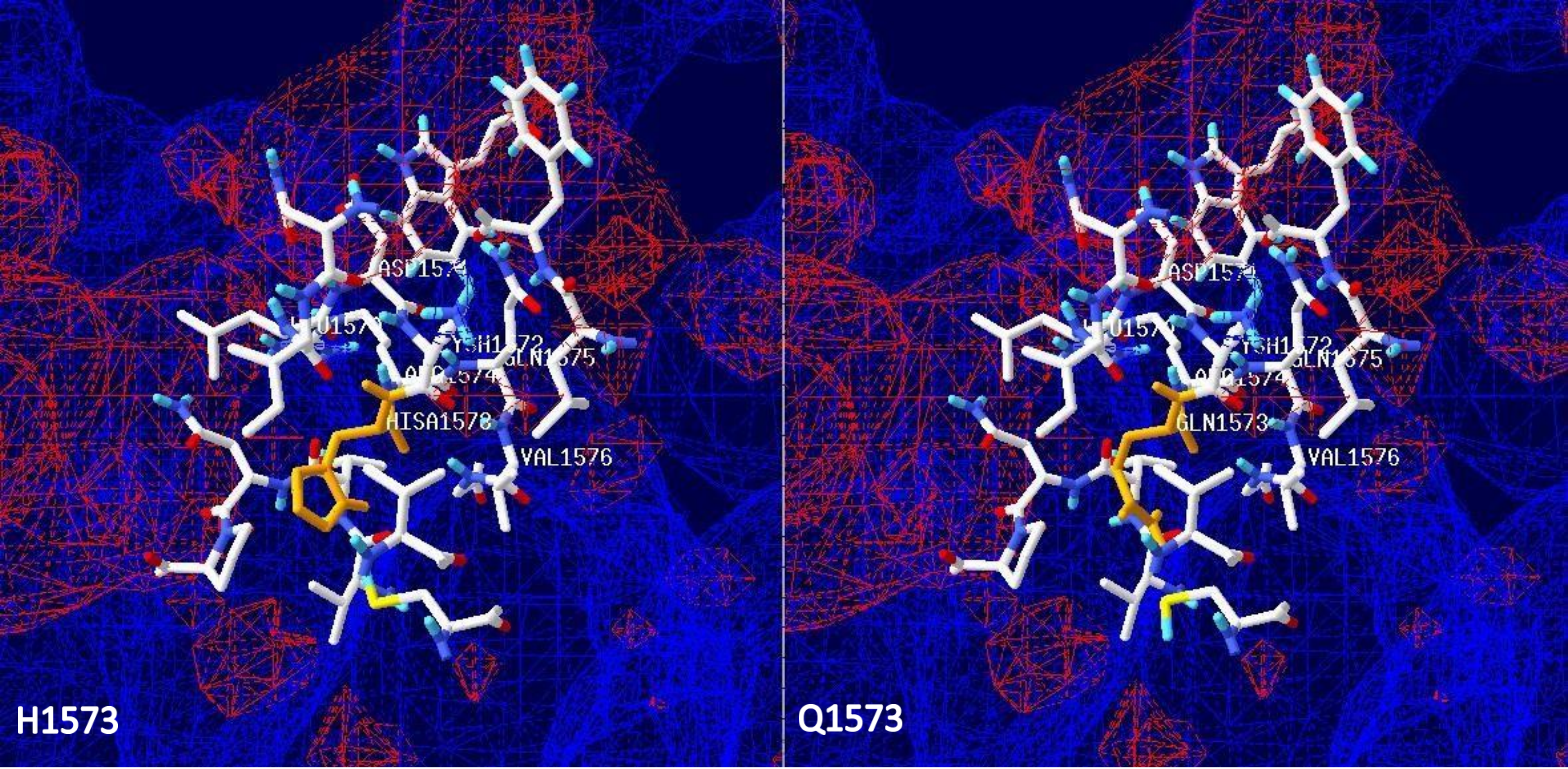

Result: Upon computing electrostatic potential: There is no change between Q1573 and H1573

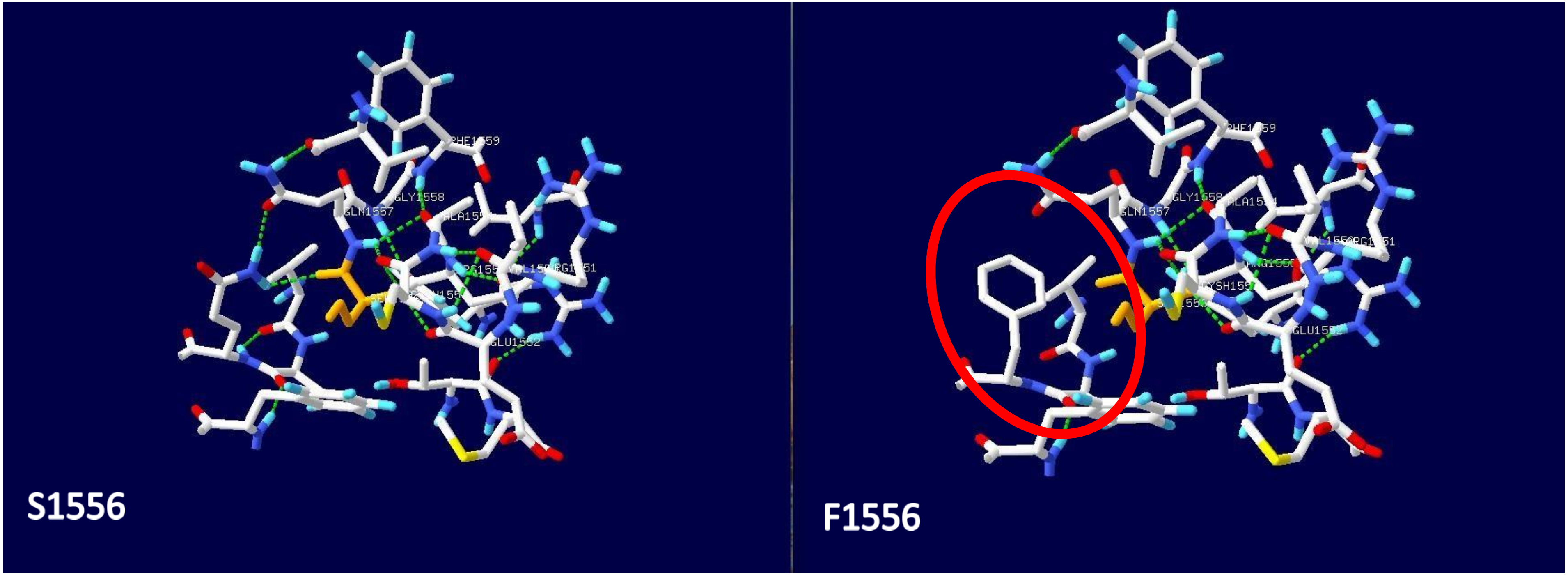

Result: Upon computing hydrogen bonds: F1556 lost 3 hydrogen bonds compared to S1556

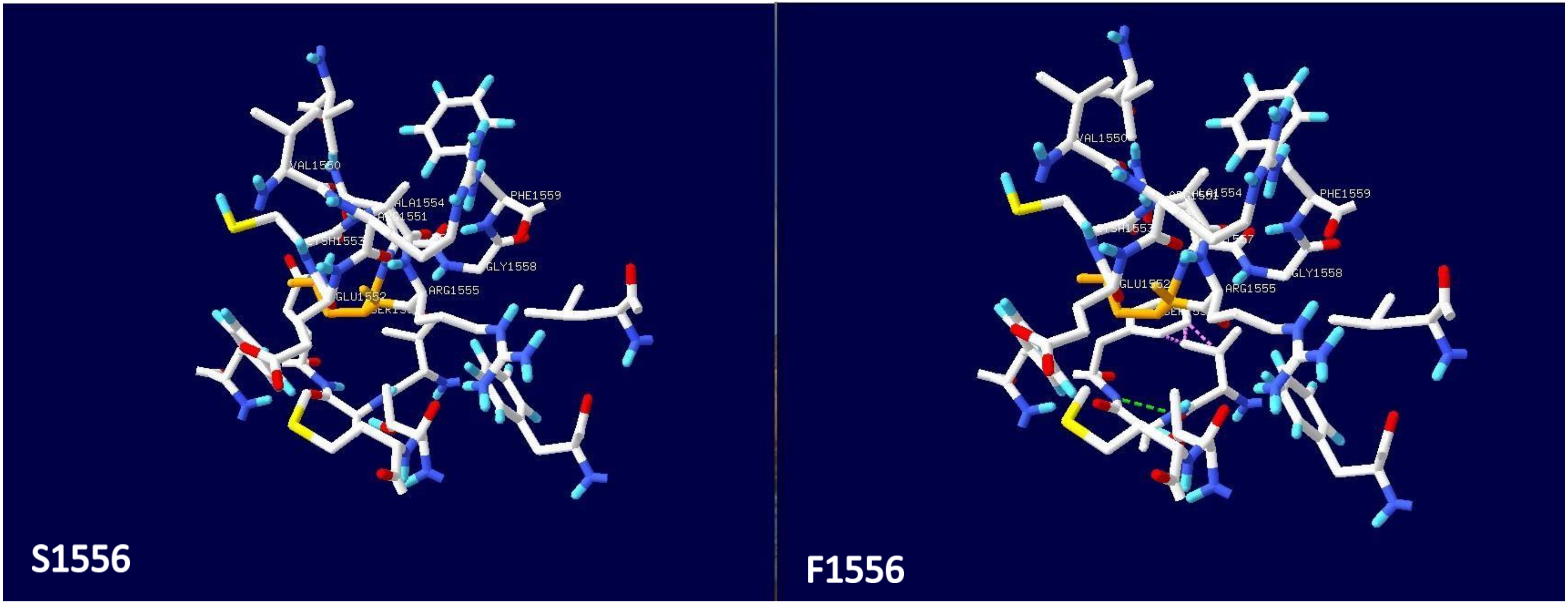

Result upon computing “mutation” effect:
F1556 gained 3 steric clashes and 1 hydrogen bonds that were absent in S1556

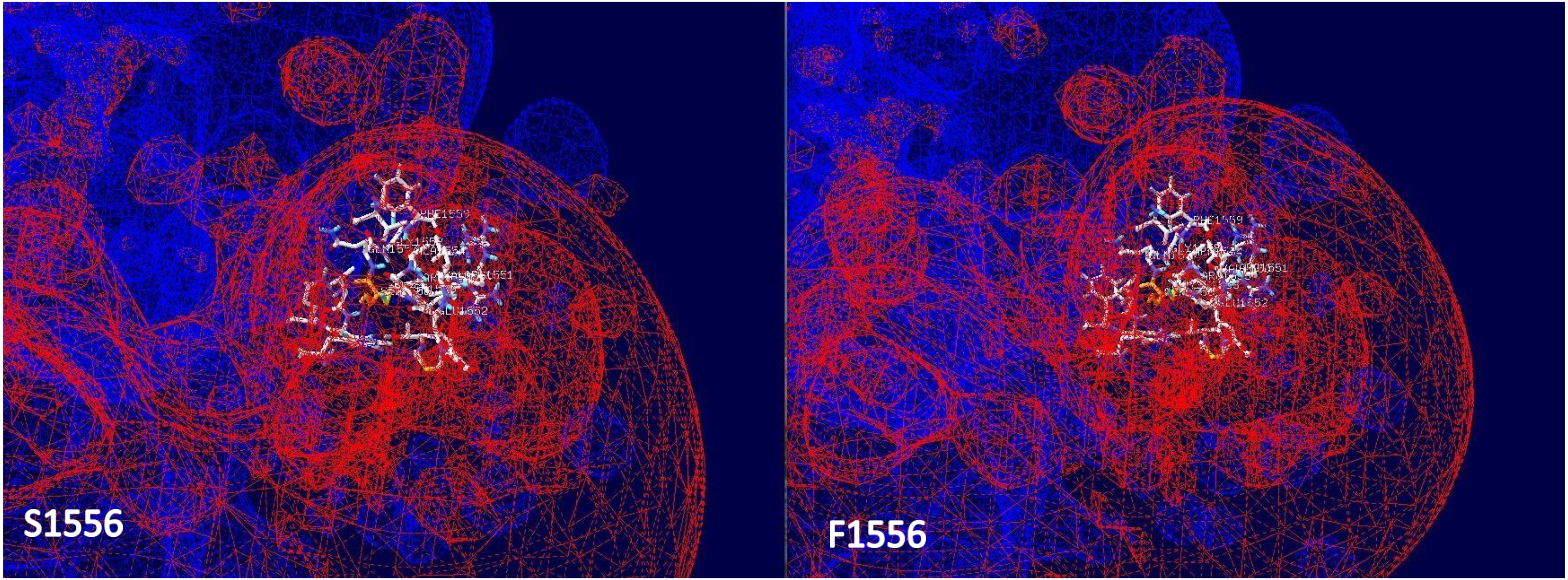

Result: Upon computing electrostatic potential: Acidic area (red mesh) around the F1556 residue became denser than the S1556 residue

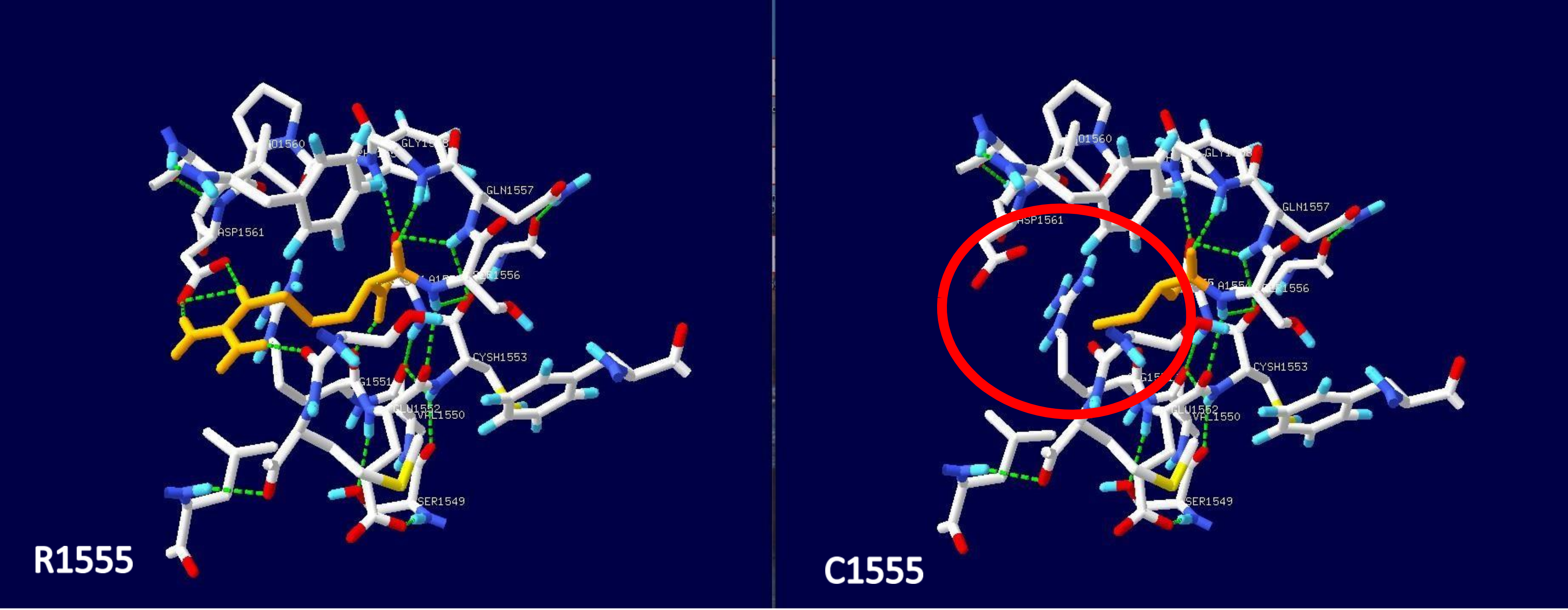

Result: Upon computing hydrogen bonds: C1555 lost 5 hydrogen bonds compared to R1555

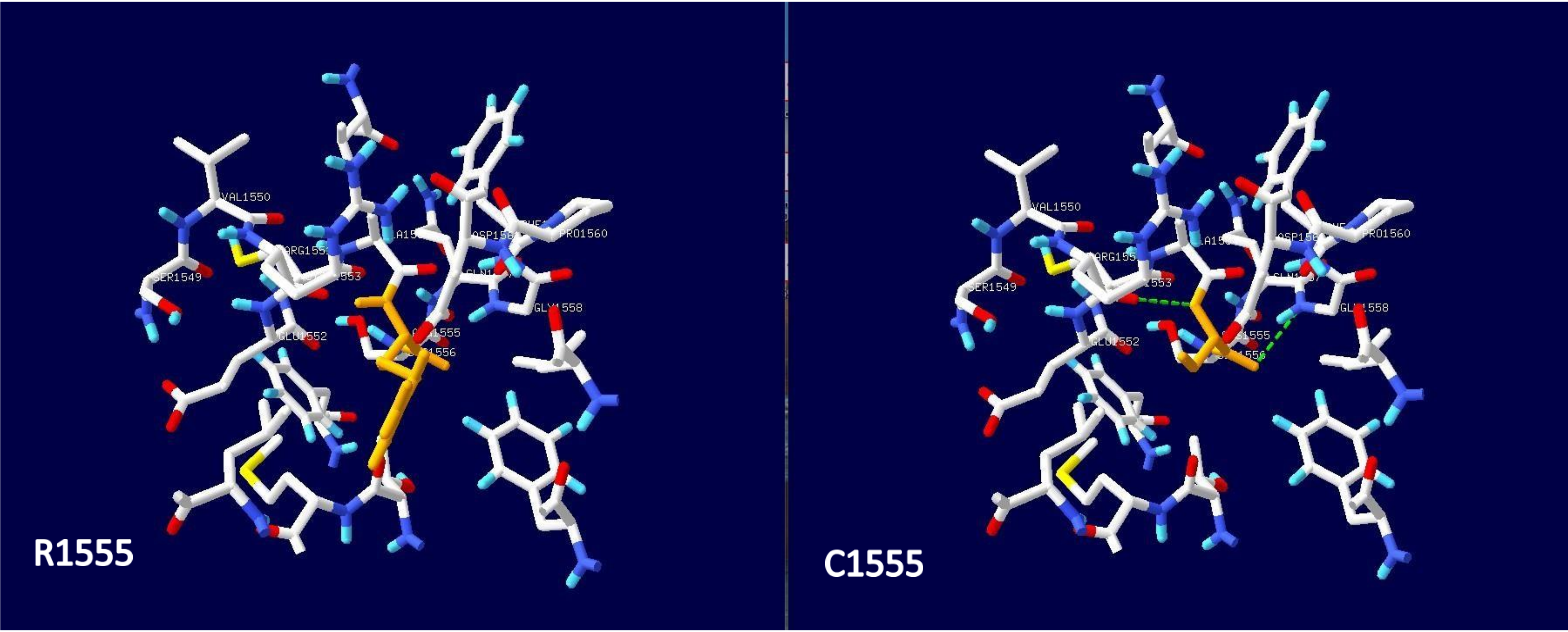

Result upon computing “mutation” effect:
C1555 gained 2 hydrogen bonds that were absent in R1555

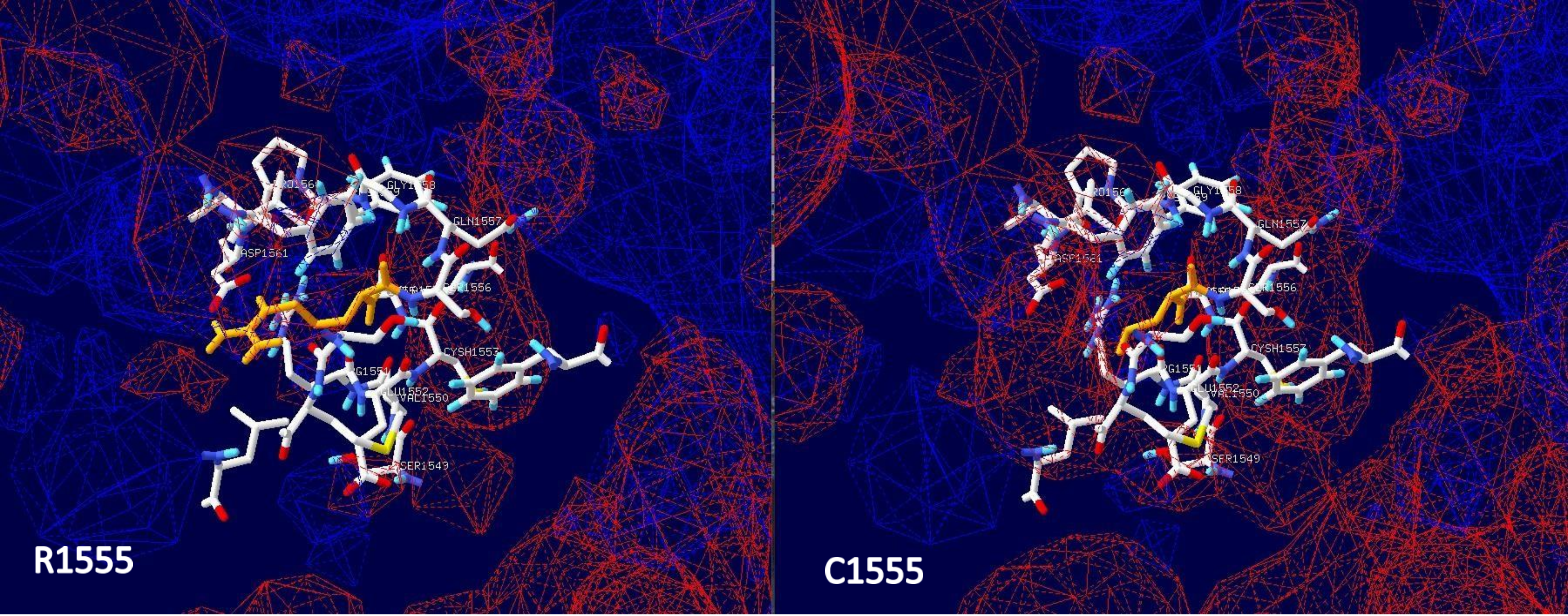

Result: Upon computing electrostatic potential: Acidic area (red mesh) around the C1555 residue became denser than the R1555 residue

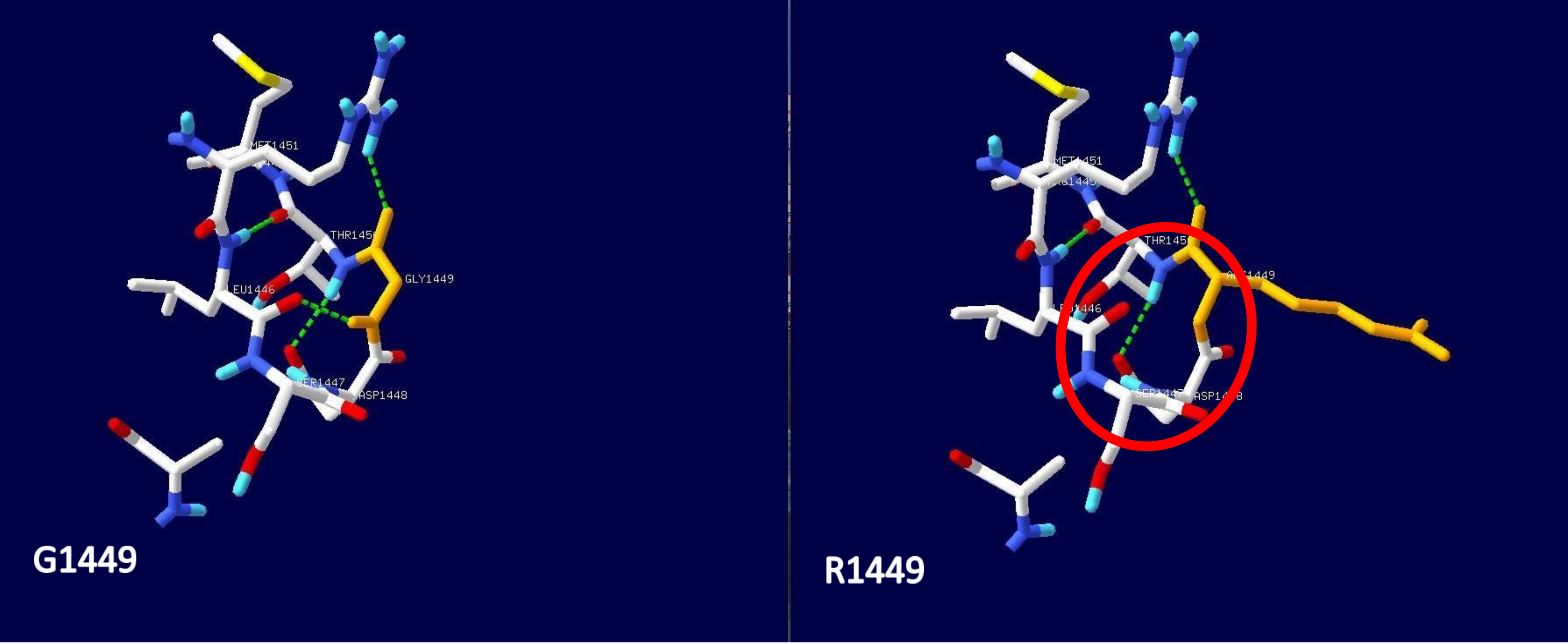

Result: Upon computing hydrogen bonds: R1449 lost hydrogen bond compared to G1449

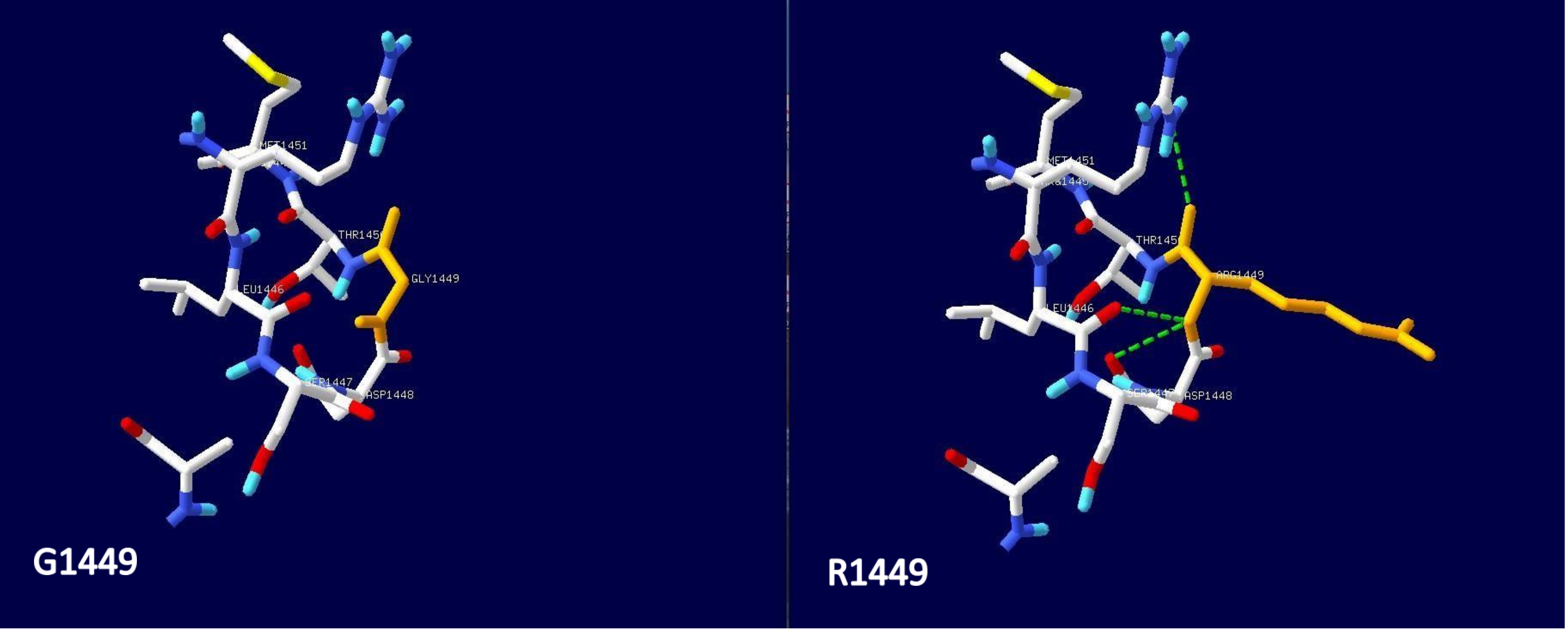

Result upon computing “mutation” effect:
R1449 gained 3 hydrogen bonds that were absent in G1449

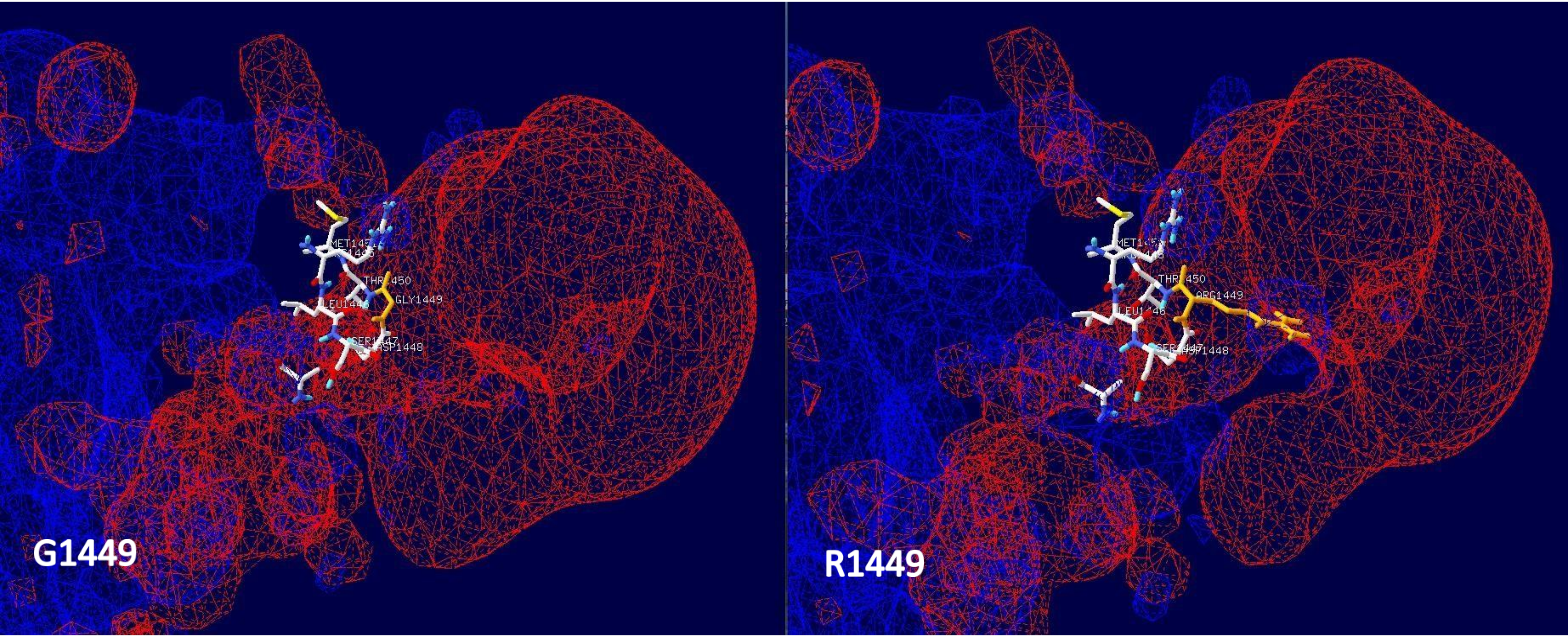

Result: Upon computing electrostatic potential: Acidic area (red mesh) around the R1449 residue became lighter than the G1449 residue and Basic area(blue mesh) is created around the R1449 residue.

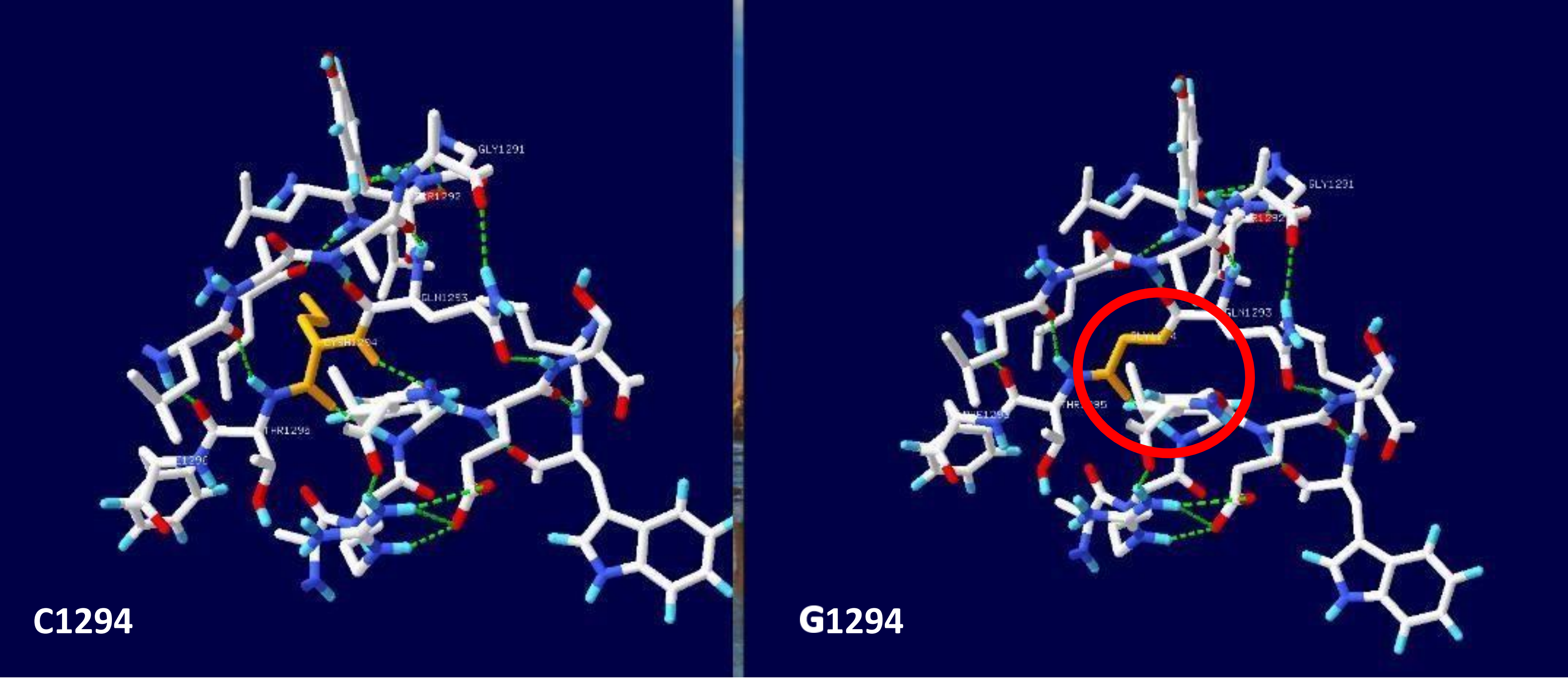

Result: Upon computing hydrogen bonds: G1294 lost 1 hydrogen bond compared to C1294

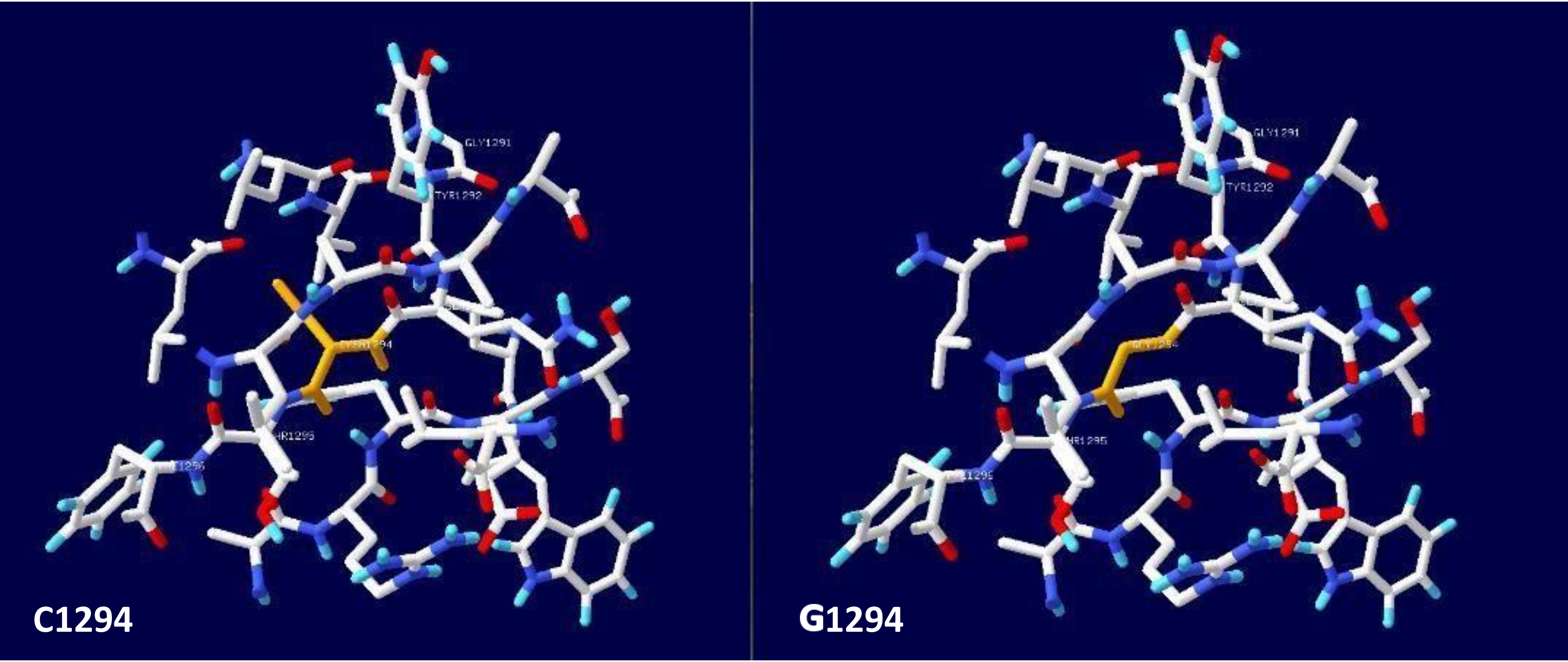

Result upon computing “mutation” effect:
There is no change between G1294 and C1294

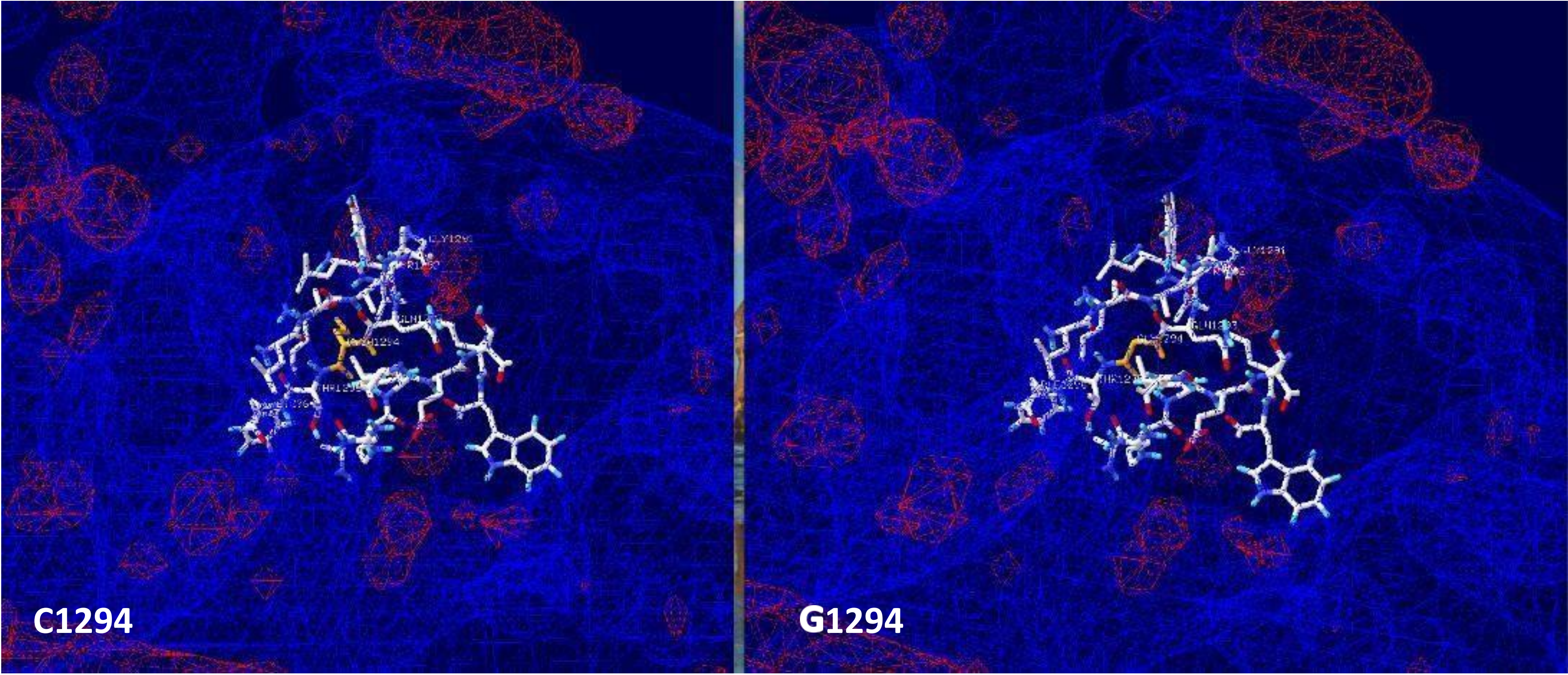

Result: Upon computing electrostatic potential: There is no change between G1294 and C1294

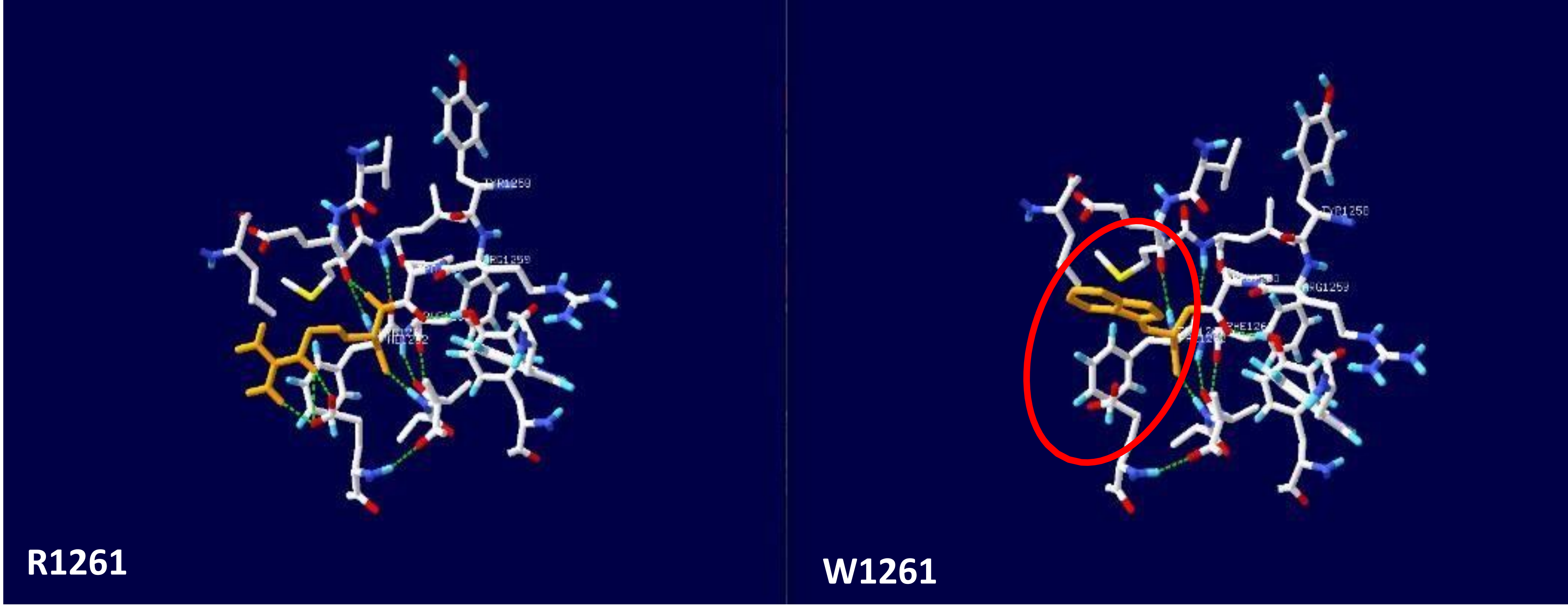

Result: Upon computing hydrogen bonds: W1261 lost 4 hydrogen bonds compared to R1261

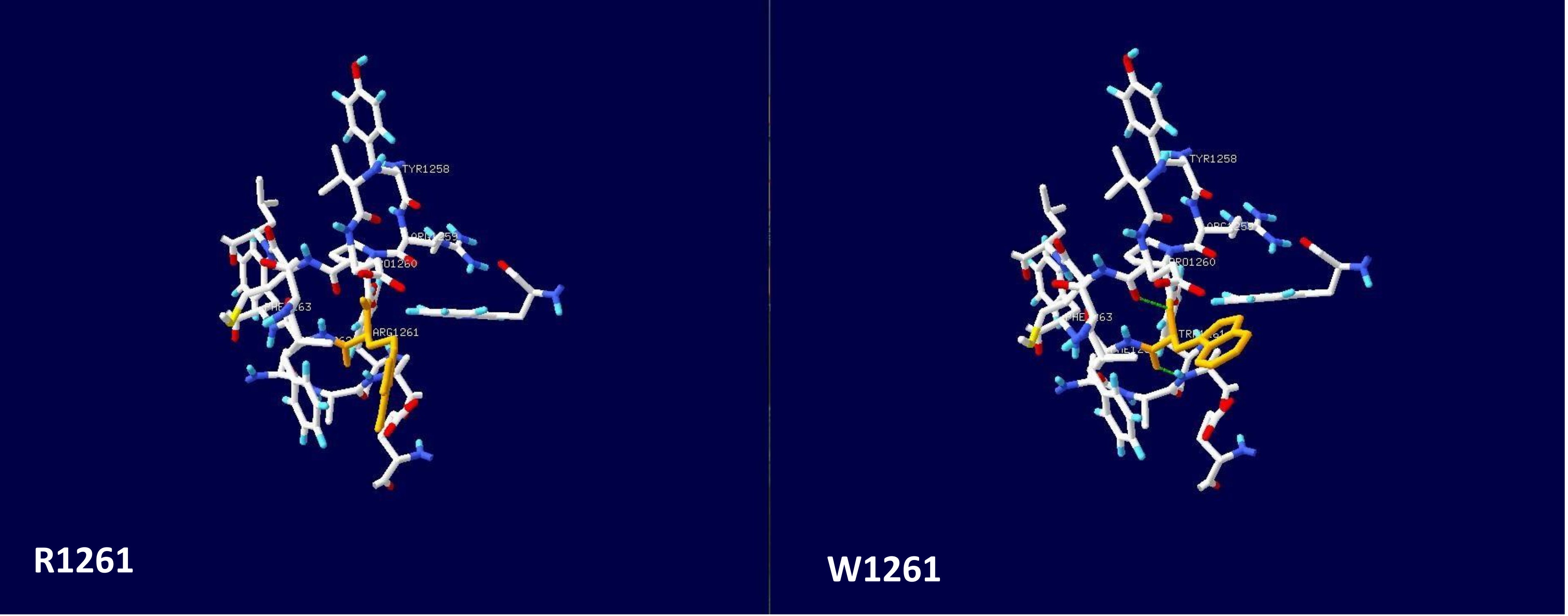

Result upon computing “mutation” effect:
W1261 gained 2 hydrogen bonds that were absent in R1261

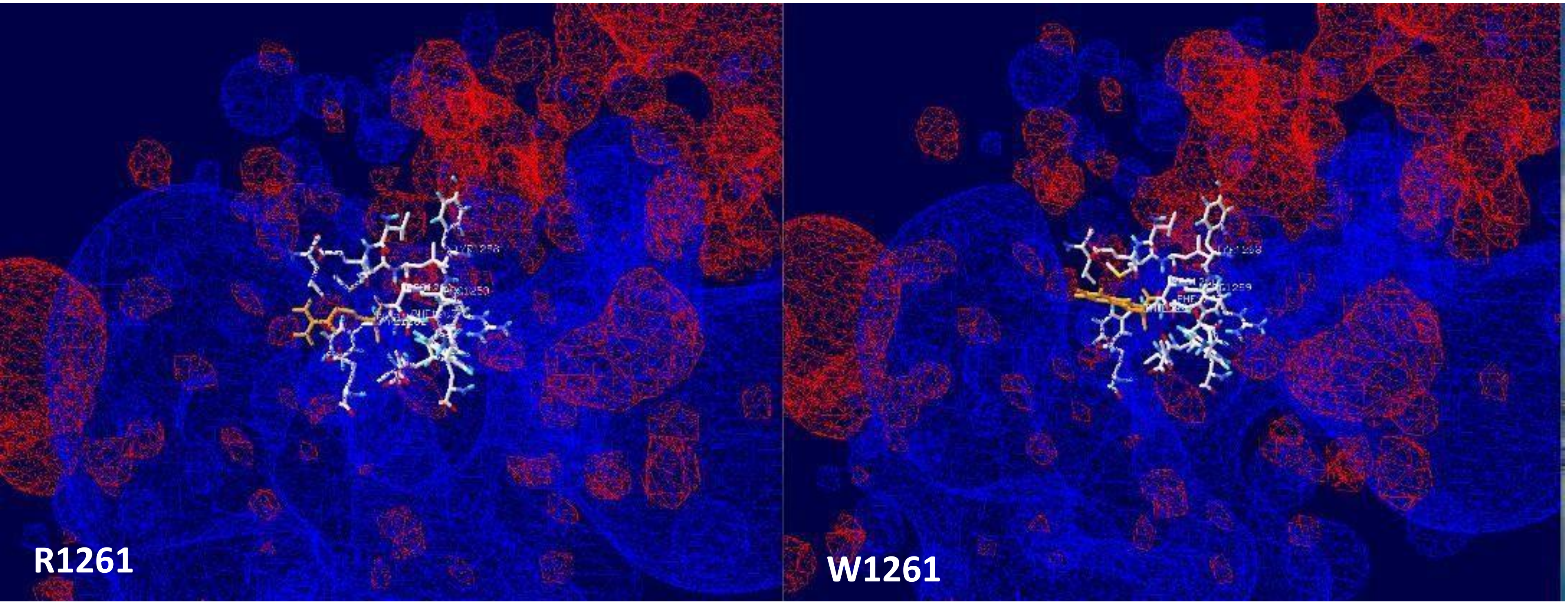

Result: Upon computing electrostatic potential: Basic area(blue mesh) around the W1261 residue became lighter than the R1261 residue.

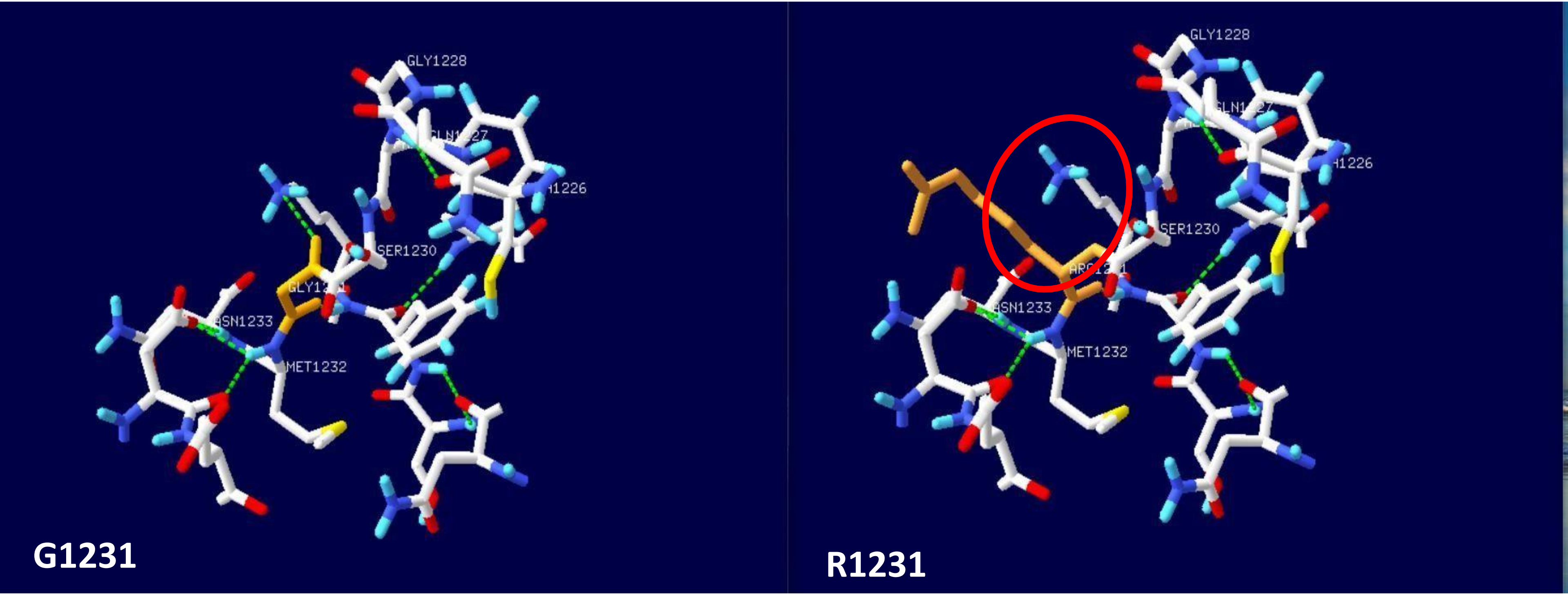

Result: Upon computing hydrogen bonds: R1231 lost 1 hydrogen bond compared to G1231

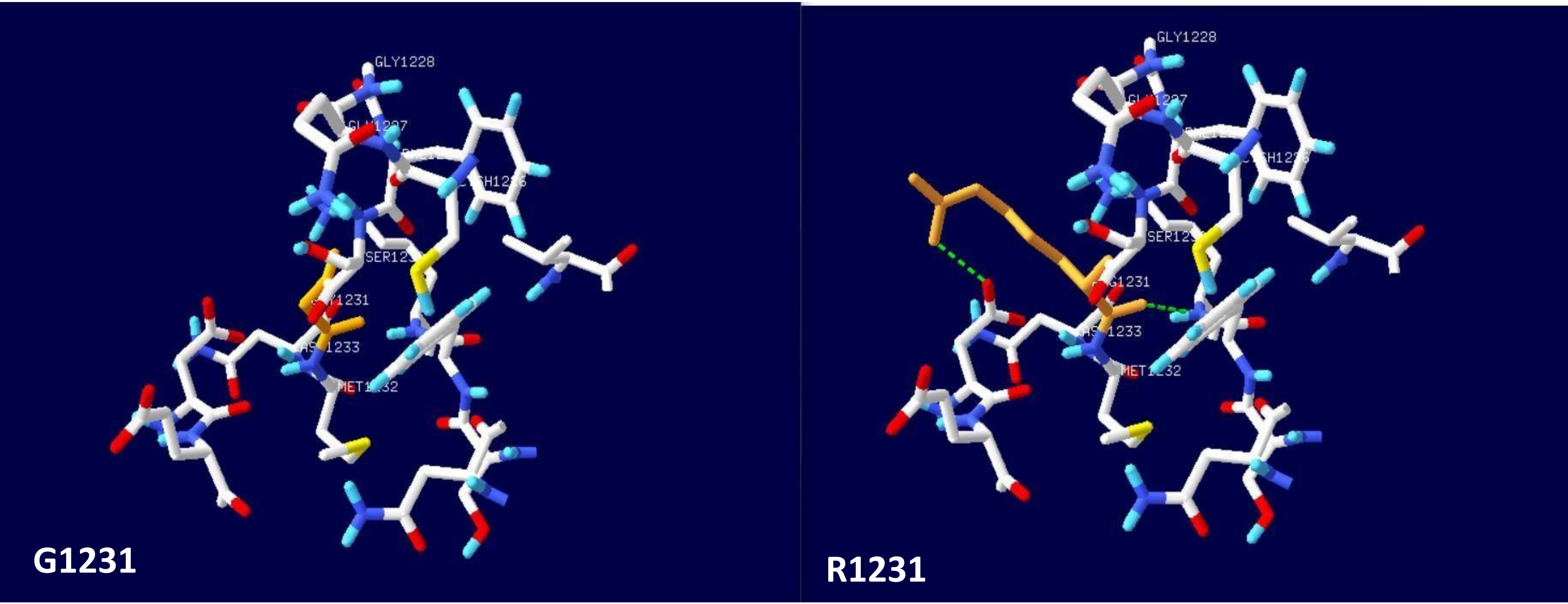

Result upon computing "mutation" effect:
R1231 gained 2 hydrogen bonds that were absent in G1231

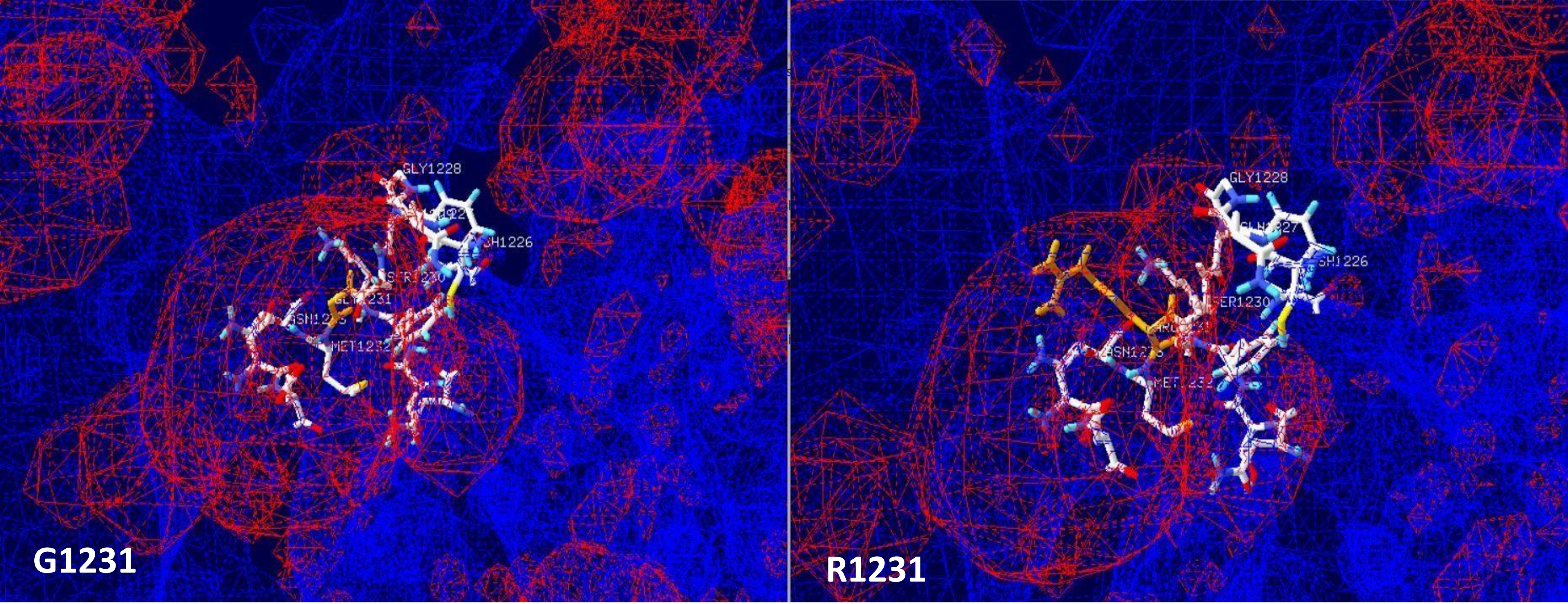

Result: Upon computing electrostatic potential: There is no change between R1231 and G1231

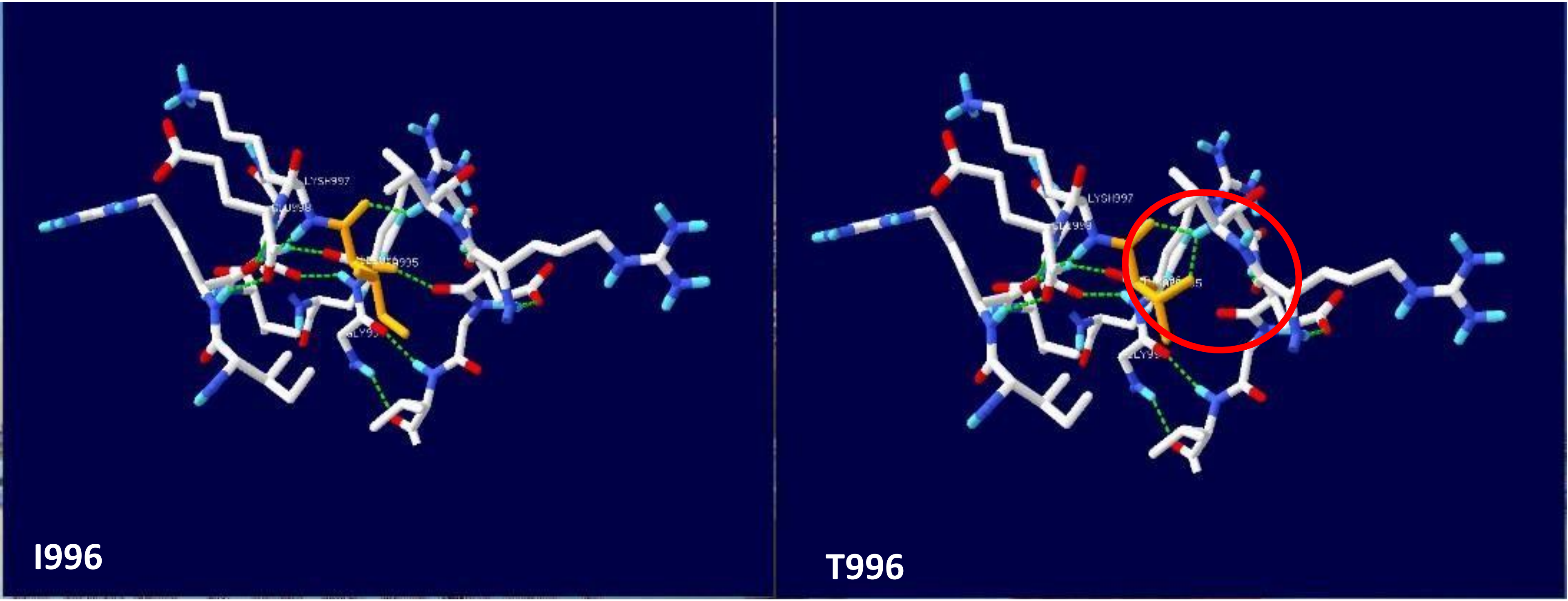

Result: Upon computing hydrogen bonds: T996 lost 1 hydrogen bond and gained 1 hydrogen bond compared to I996

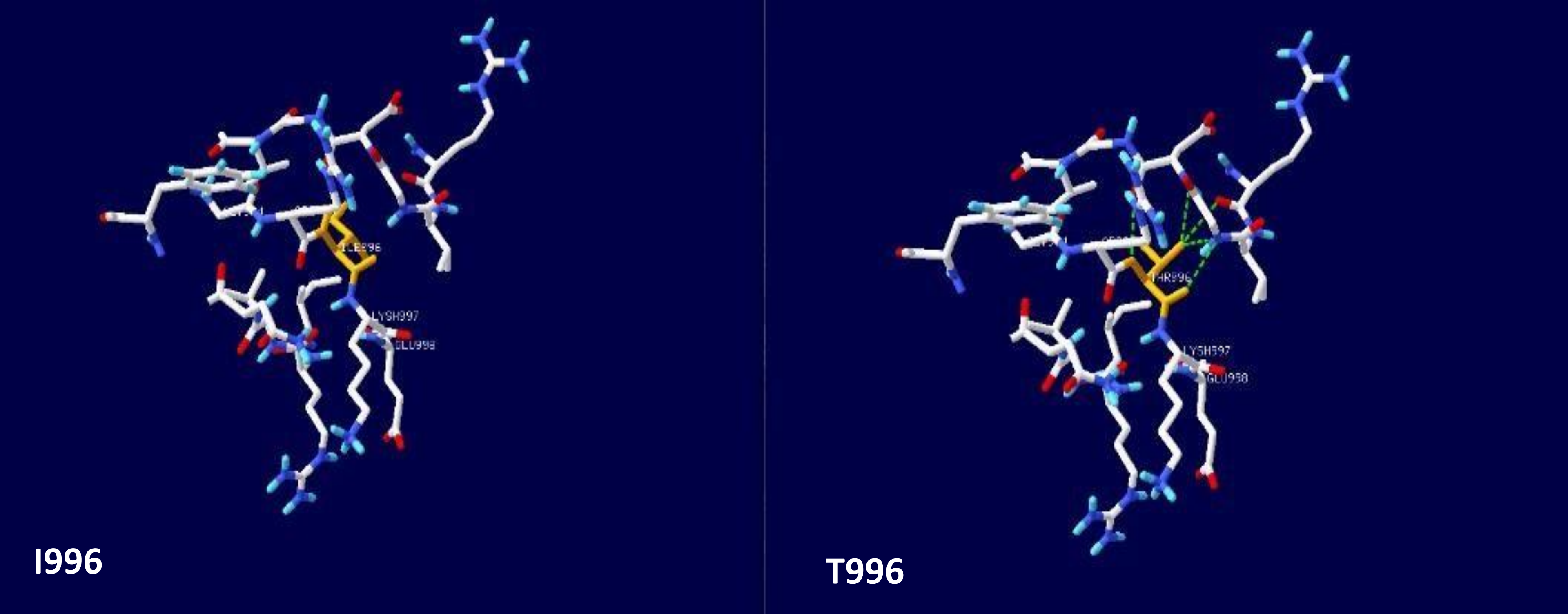

Result upon computing "mutation" effect:
T996 gained 5 hydrogen bonds that were absent in I996

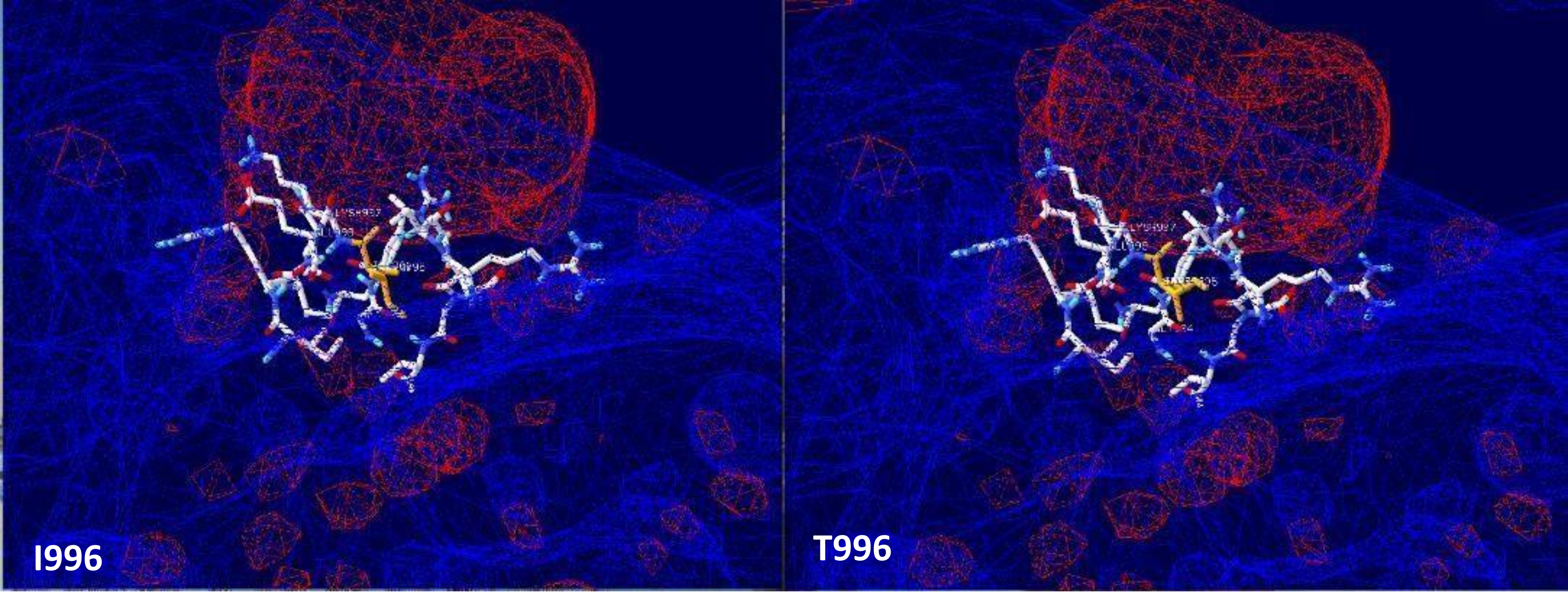

Result: Upon computing electrostatic potential: There is no change between T996 and I996

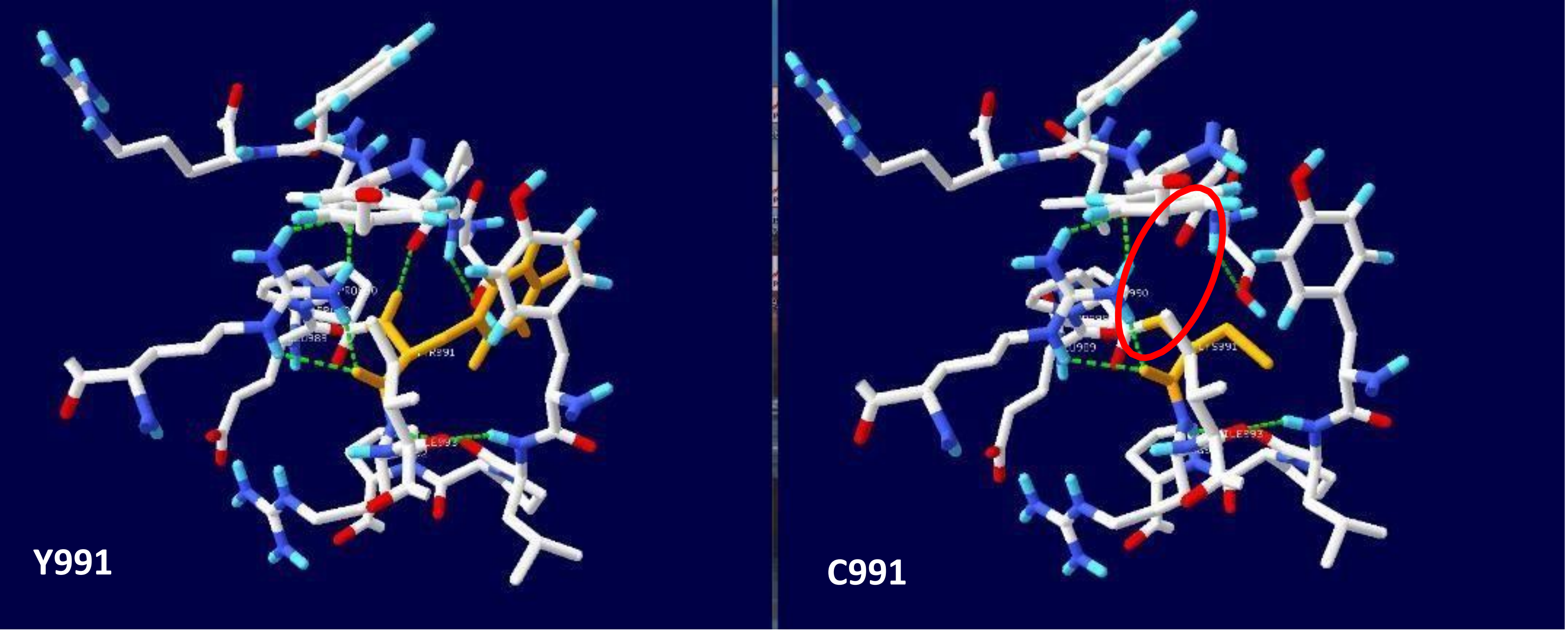

Result: Upon computing hydrogen bonds: C991 lost 1 hydrogen bond compared to C991

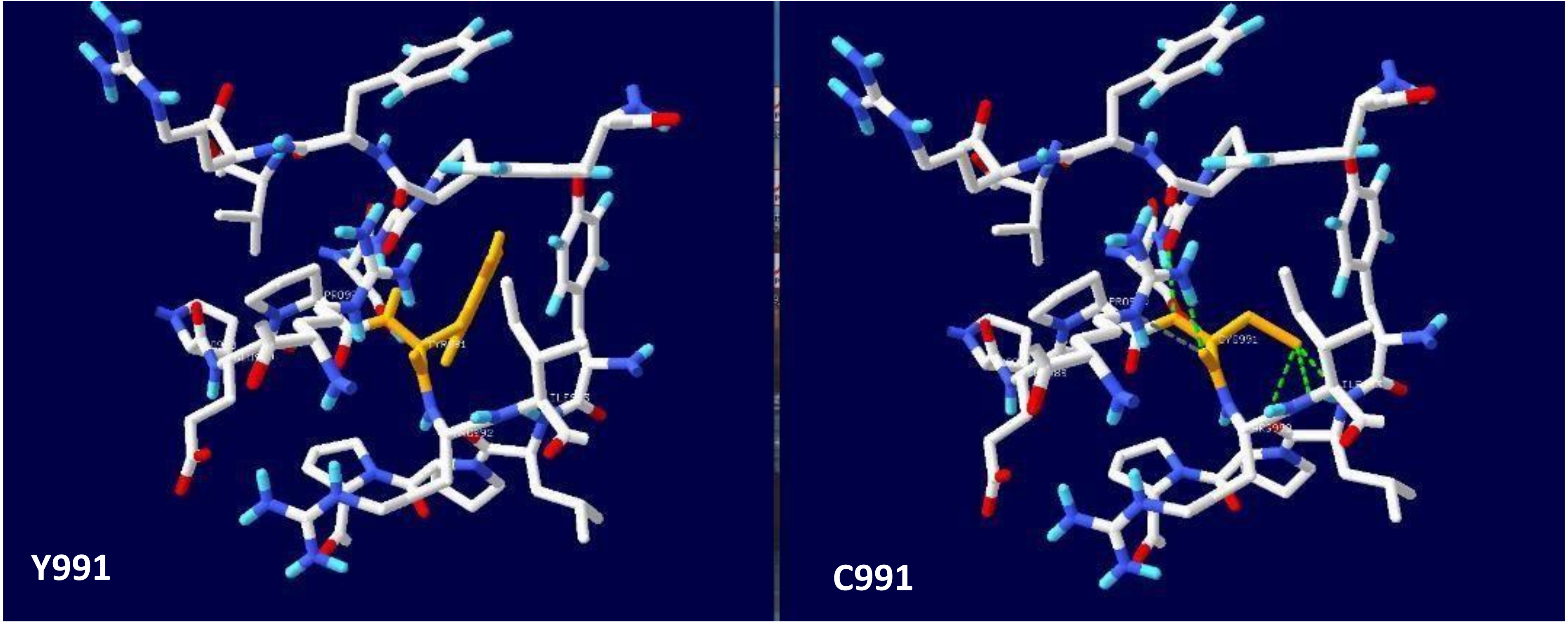

Result upon computing “mutation” effect:
C991 gained a steric clash and 5 hydrogen bonds that were absent in Y991

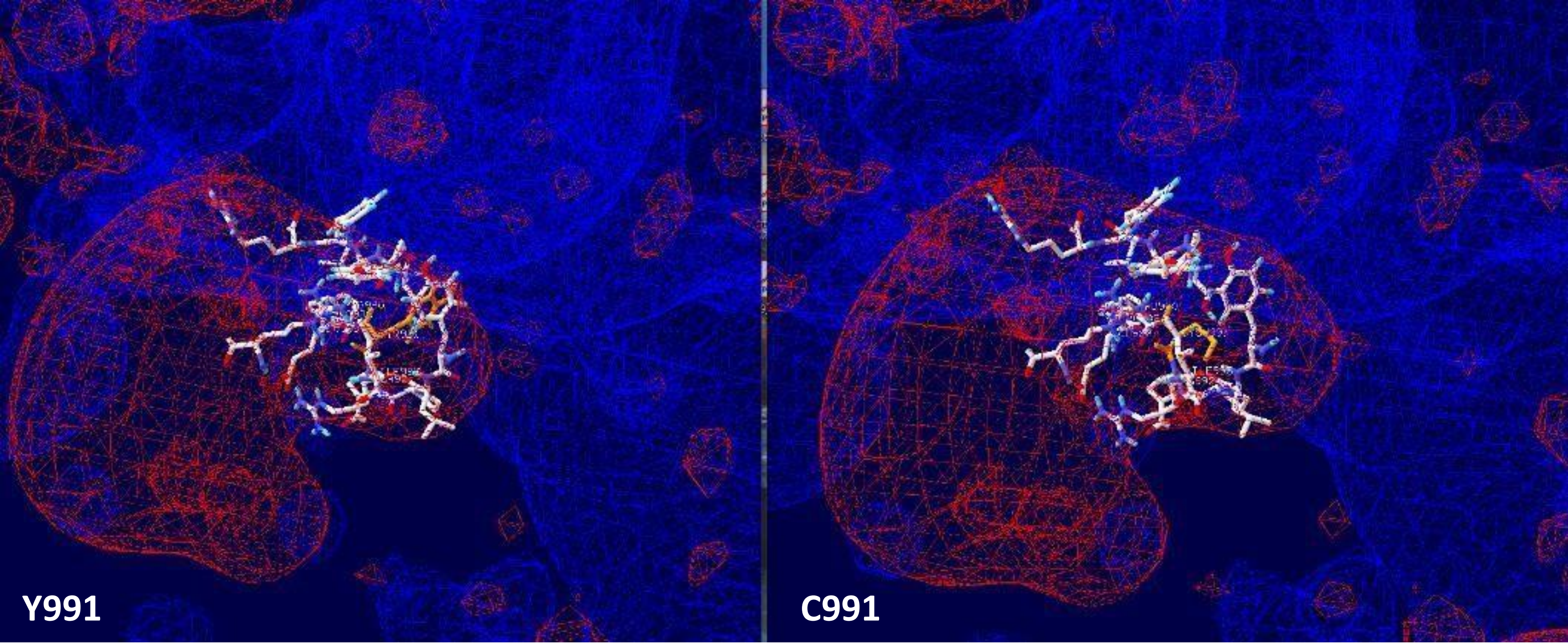

Result: Upon computing electrostatic potential: There is no change between T996 and I996

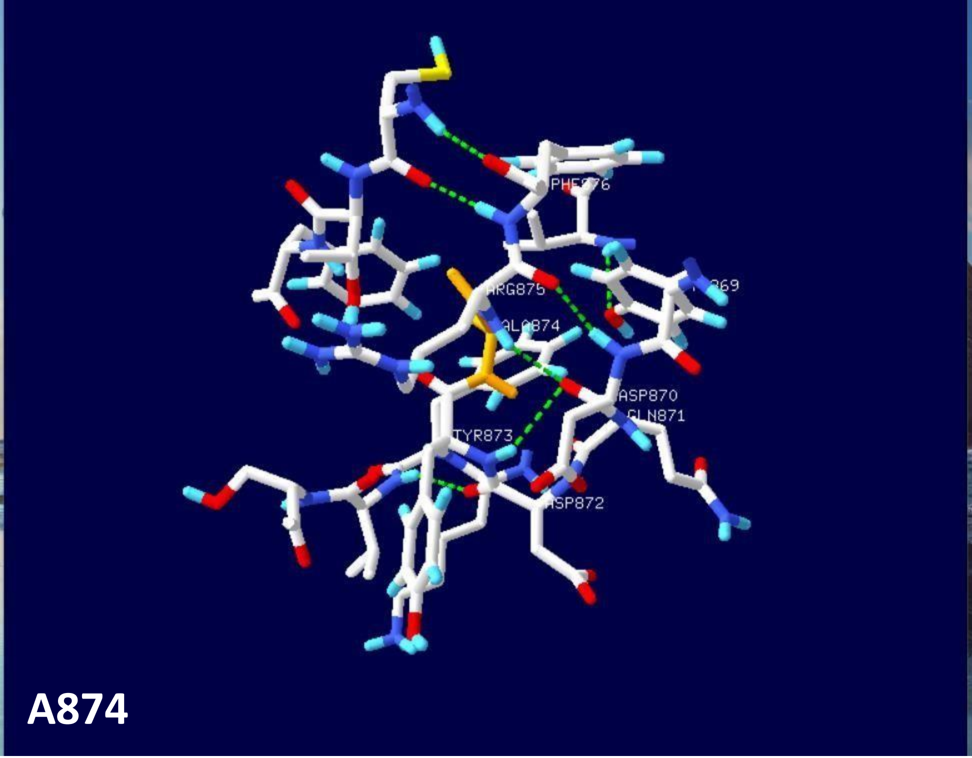

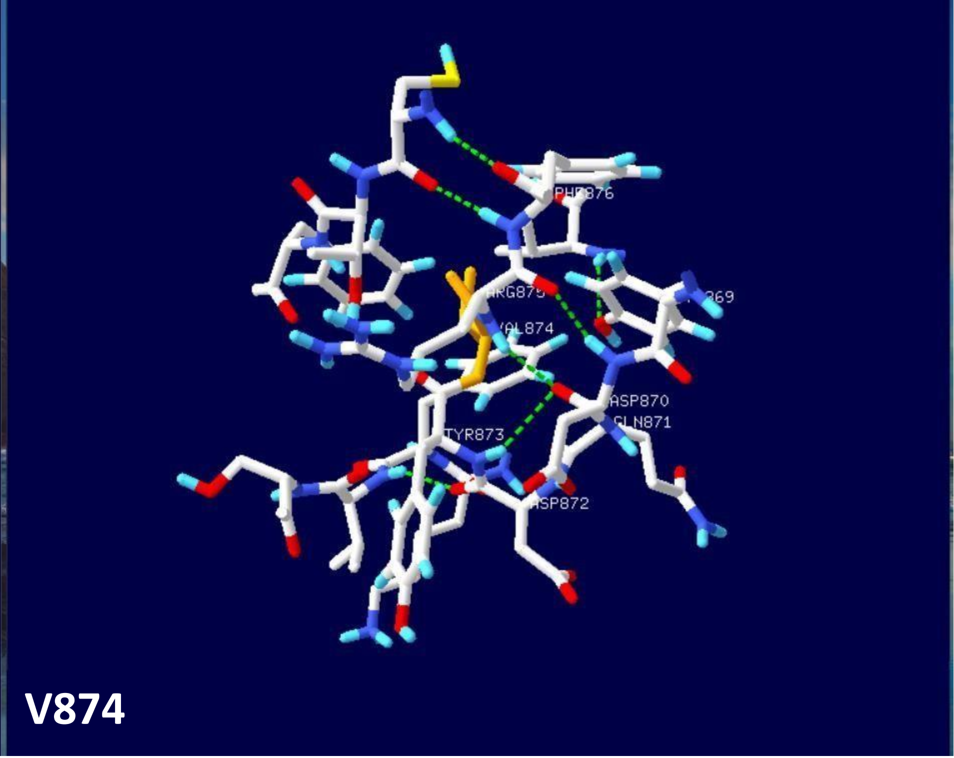

Result: Upon computing hydrogen bonds: There is no change between V874 and A874

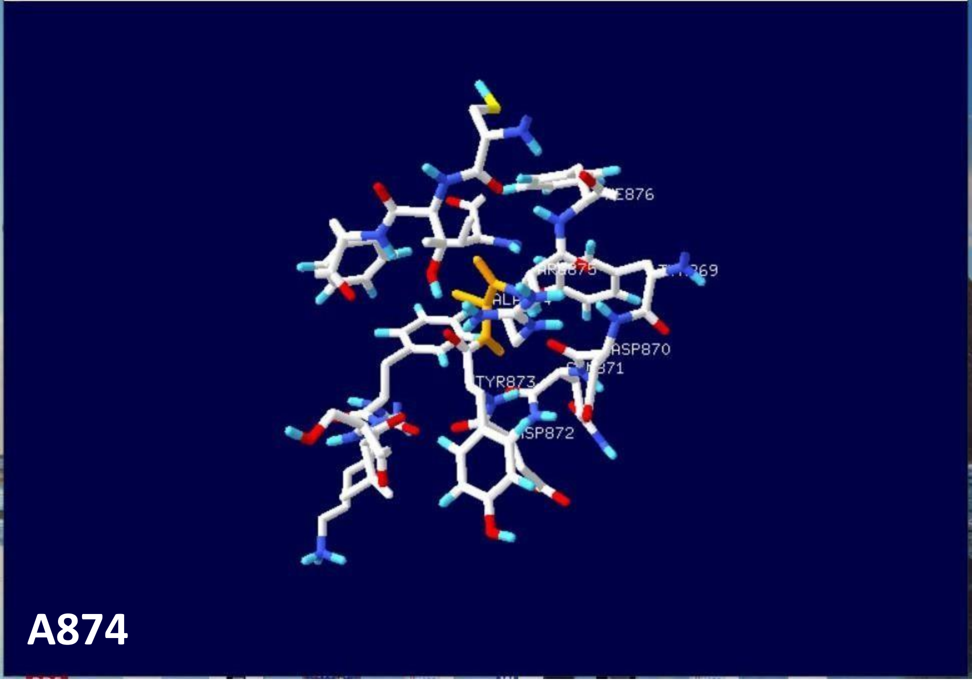

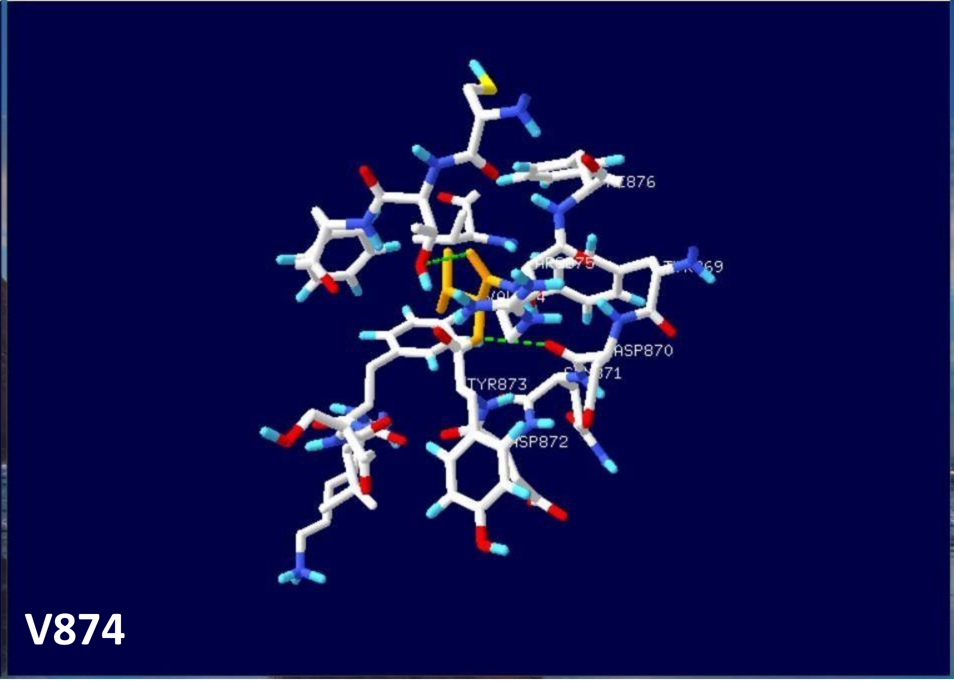

Result upon computing “mutation” effect:
V874 gained 2hydrogen bonds that were absent in A874

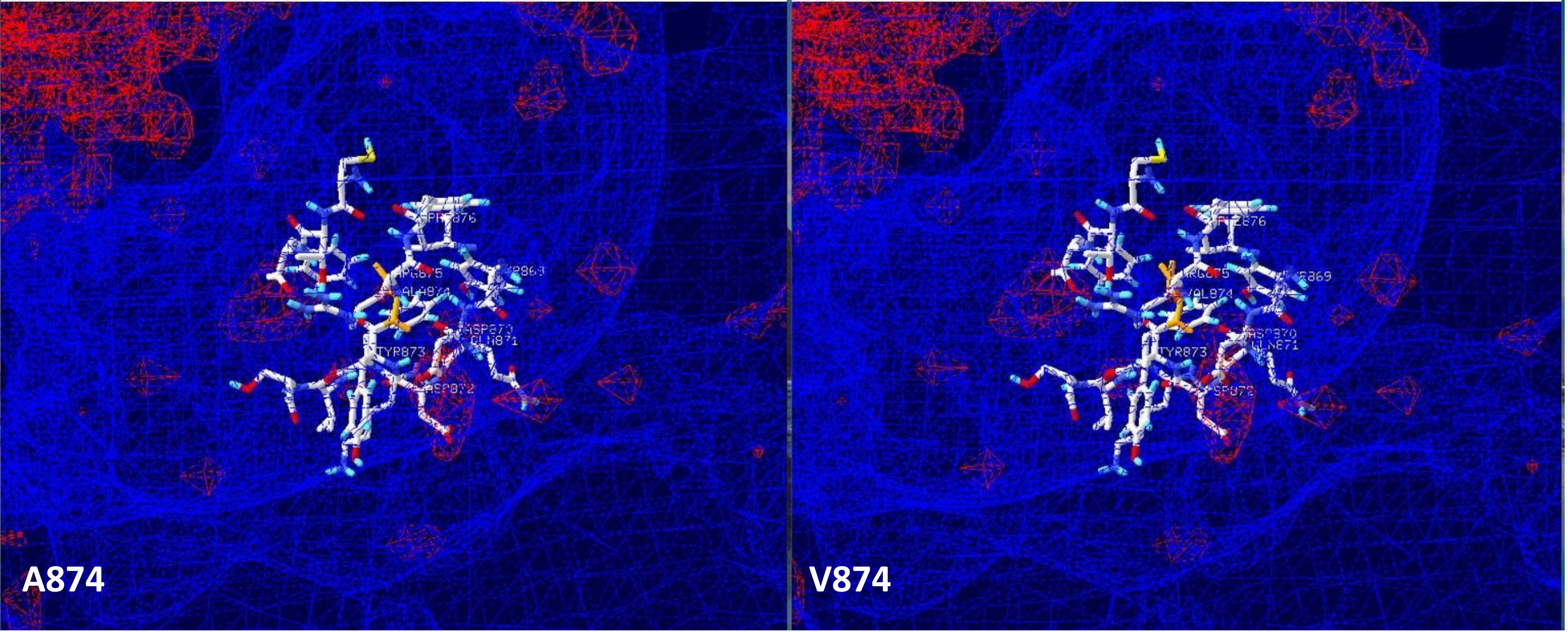

Result: Upon computing electrostatic potential: There is no change between V874 and A874

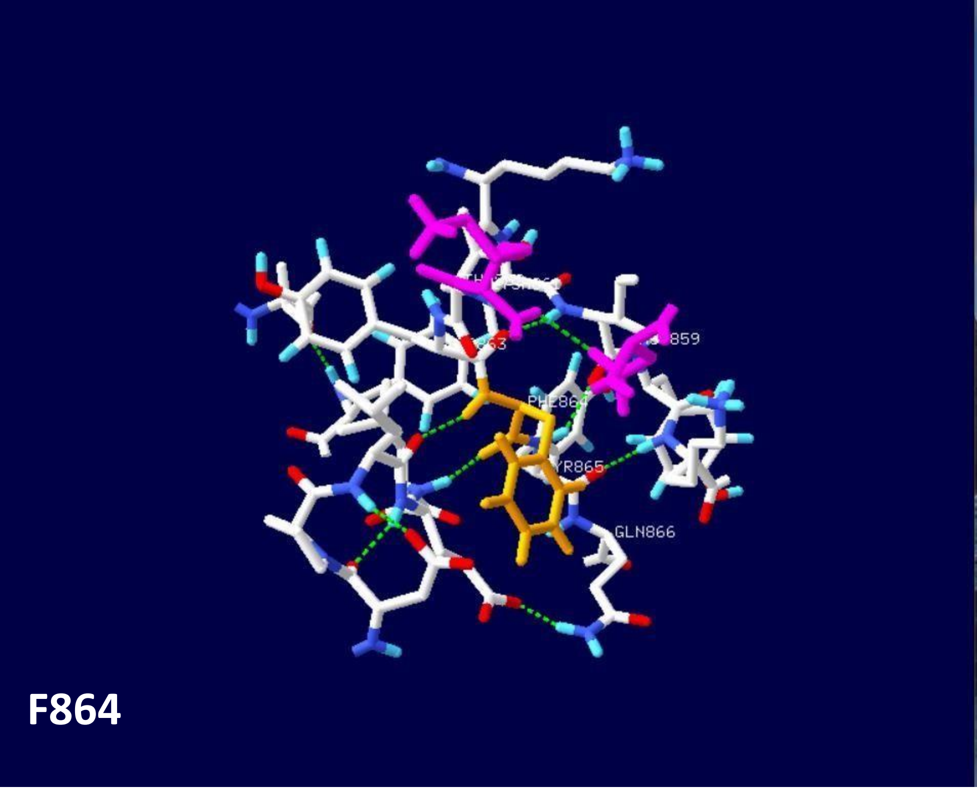

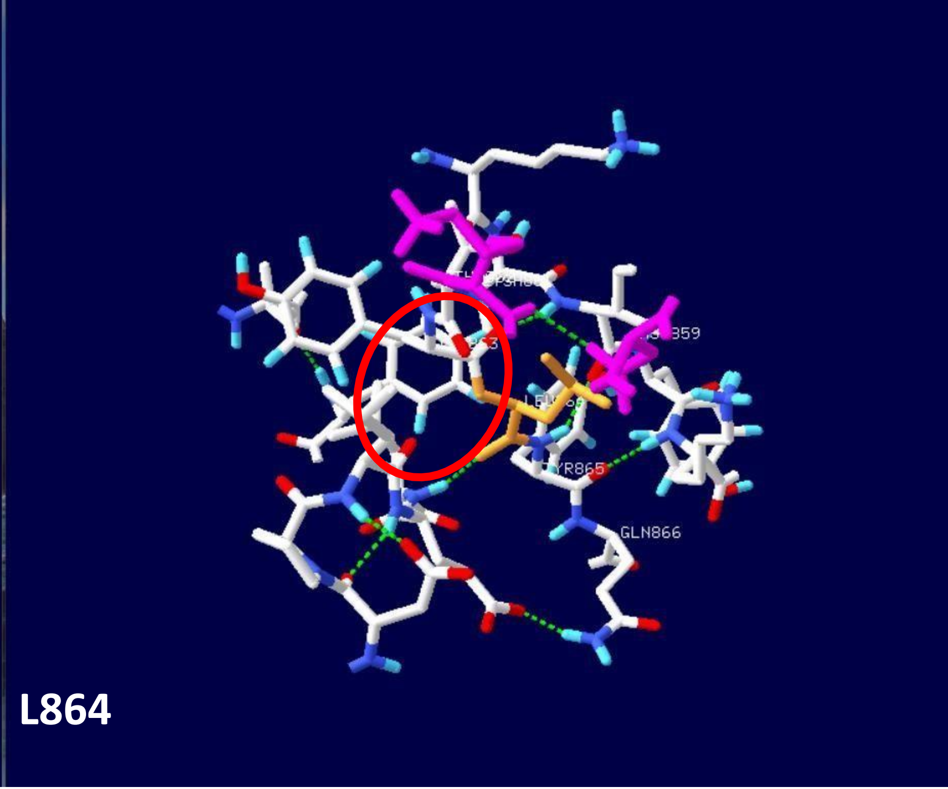

Result: Upon computing hydrogen bonds: L864 lost 1 hydrogen bond compared to F864

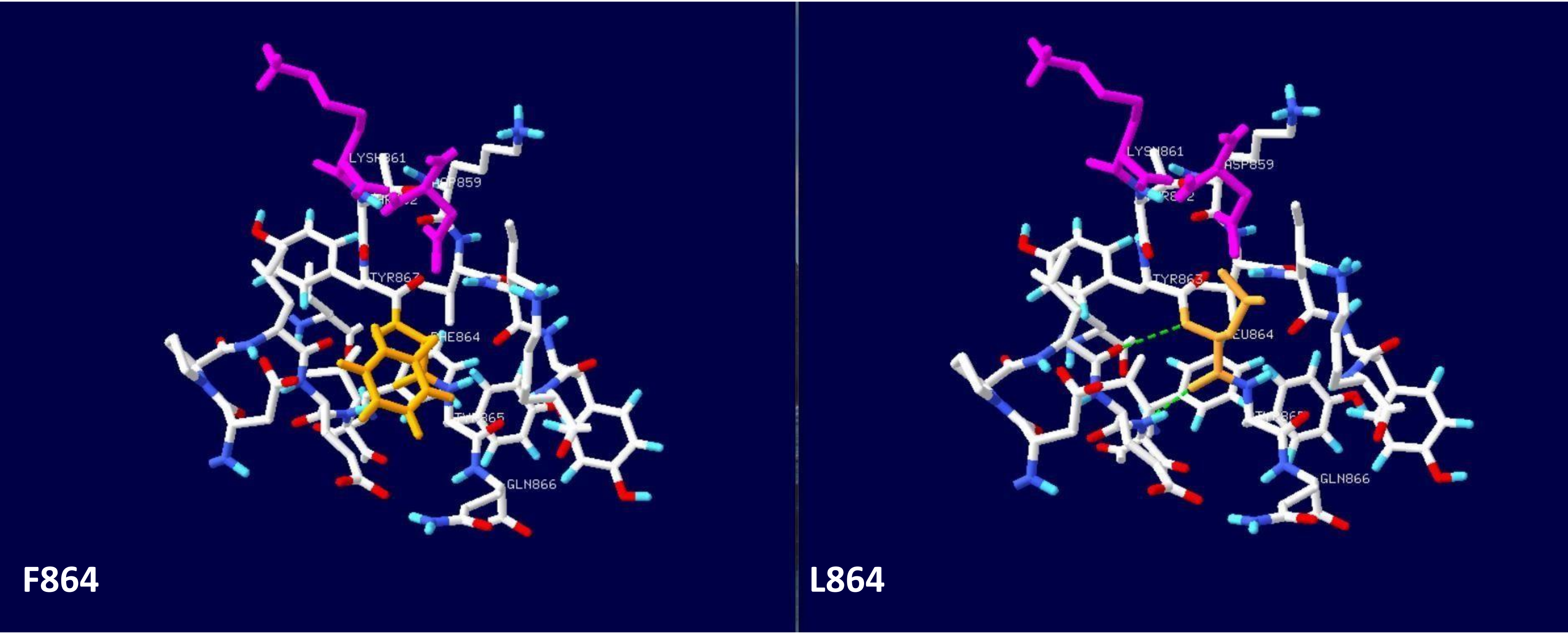

Result upon computing “mutation” effect:
L864 gained 2hydrogen bonds that were absent in F864

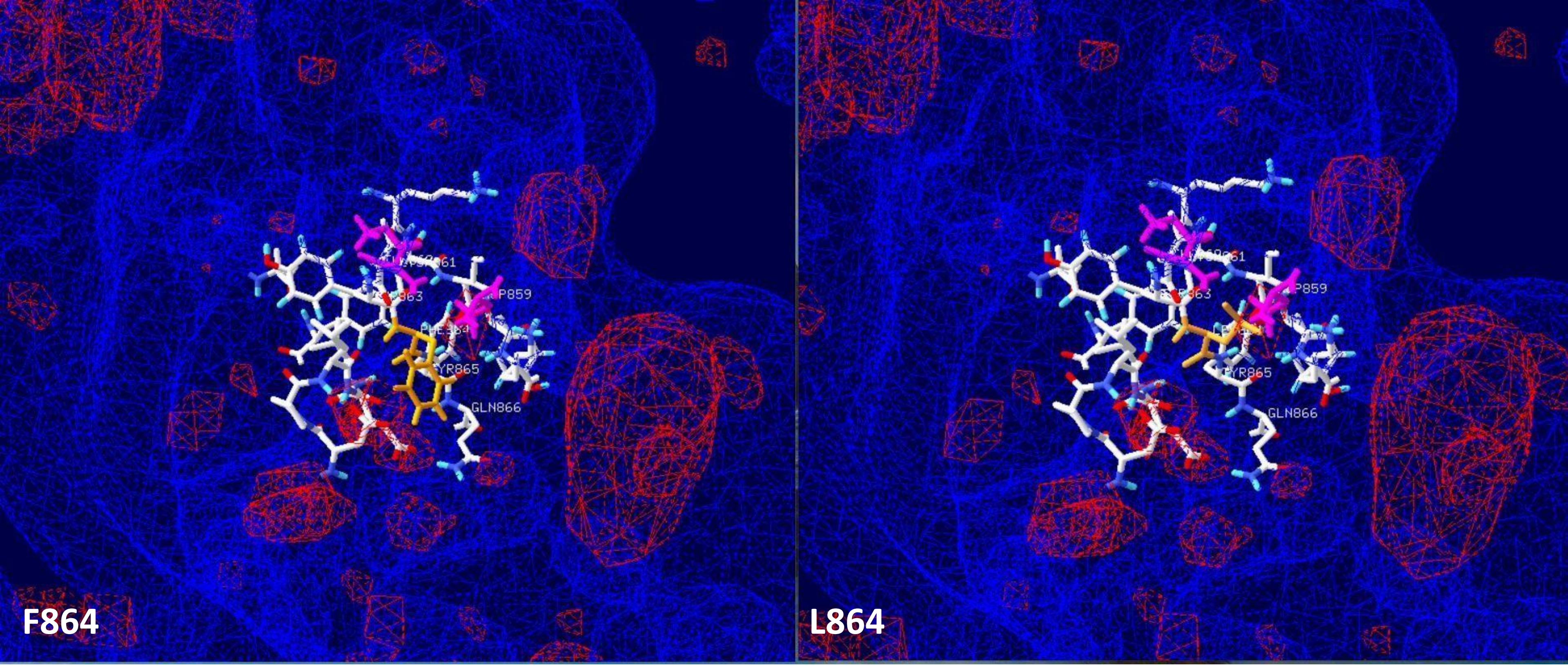

Result: Upon computing electrostatic potential: There is no change between L864 and F864

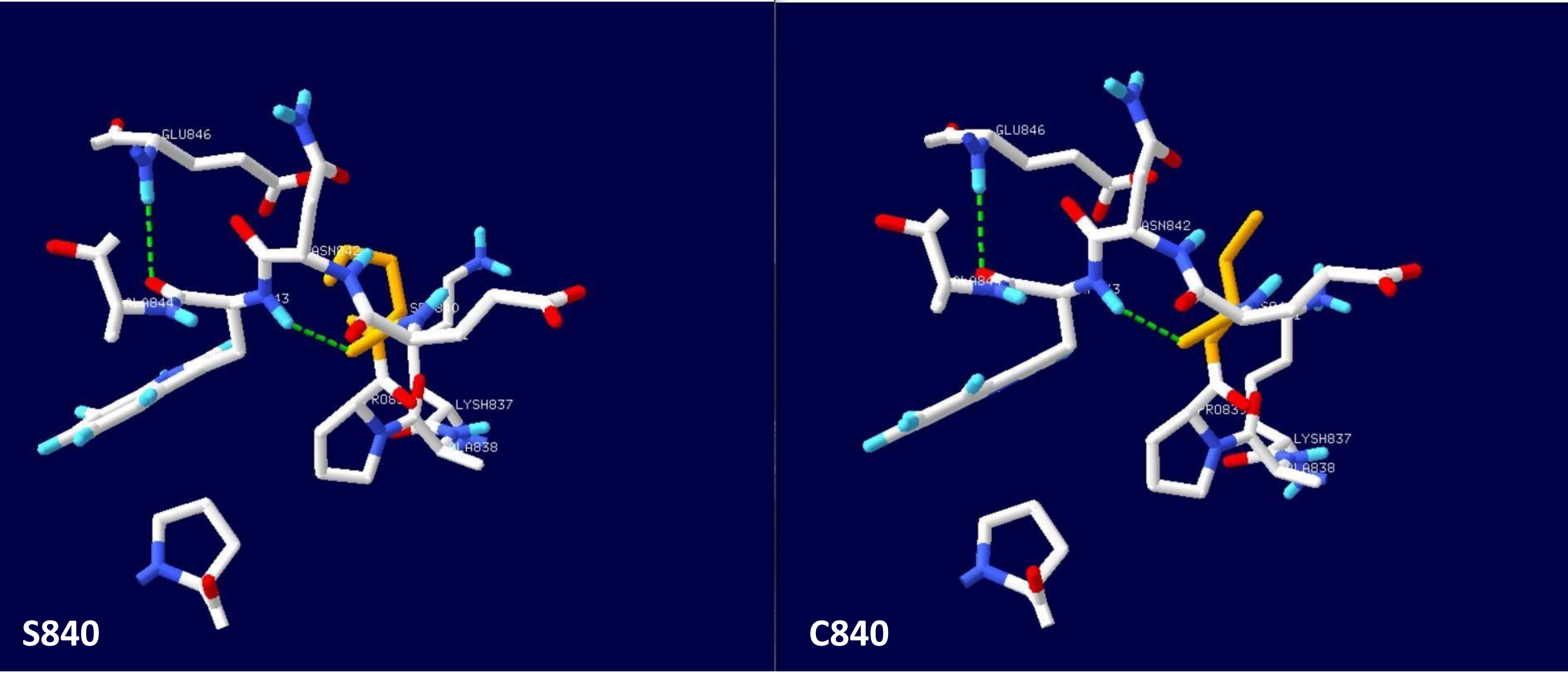

Result: Upon computing hydrogen bonds: There is no change between C840 and S840

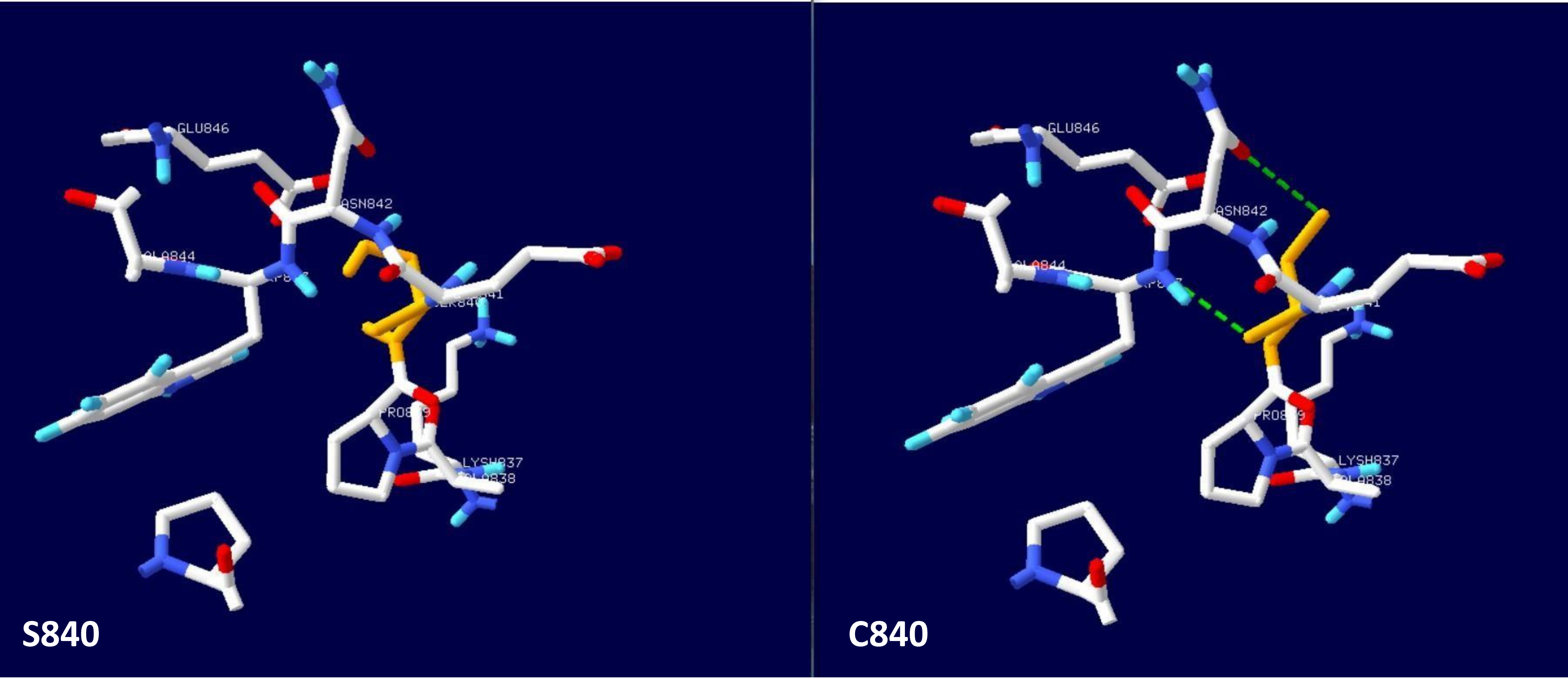

Result upon computing “mutation” effect:
C840 gained 2 hydrogen bonds that were absent in S840

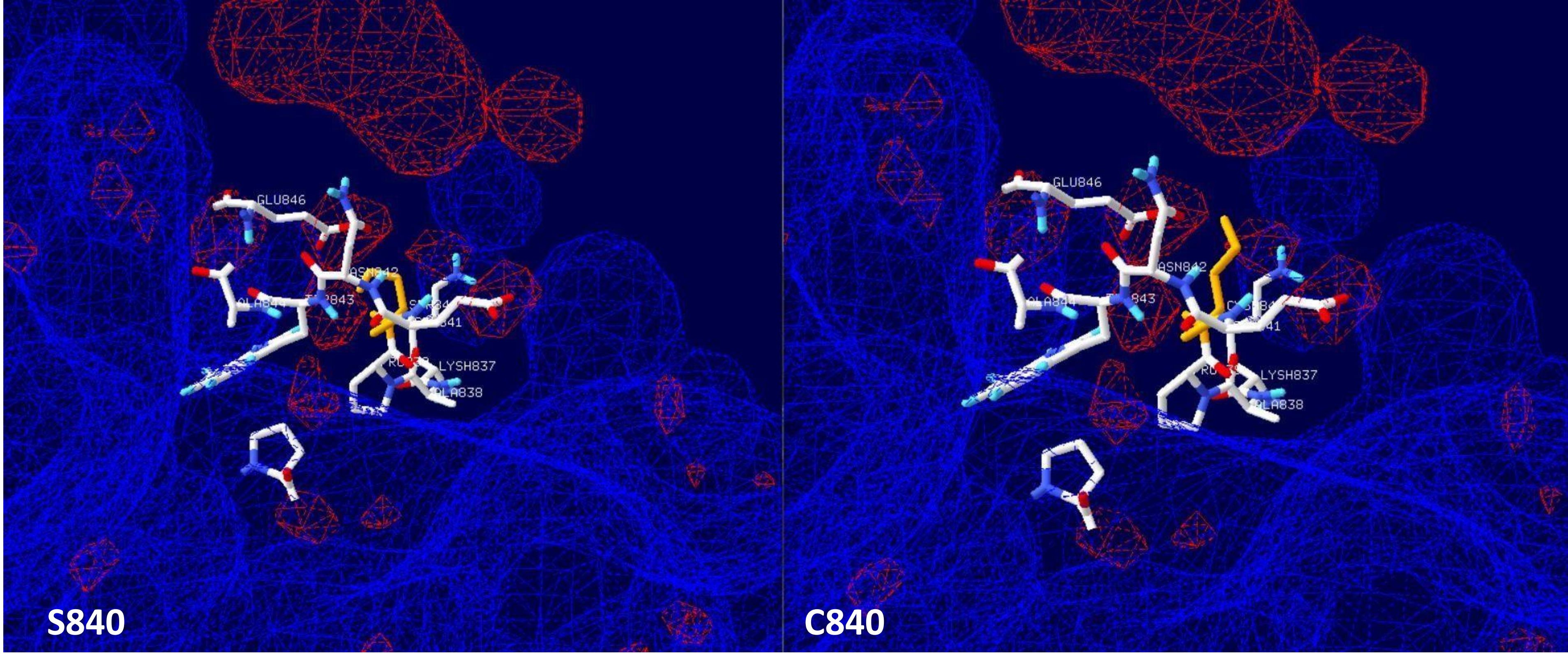

Result: Upon computing electrostatic potential: There is no change between C840 and S840

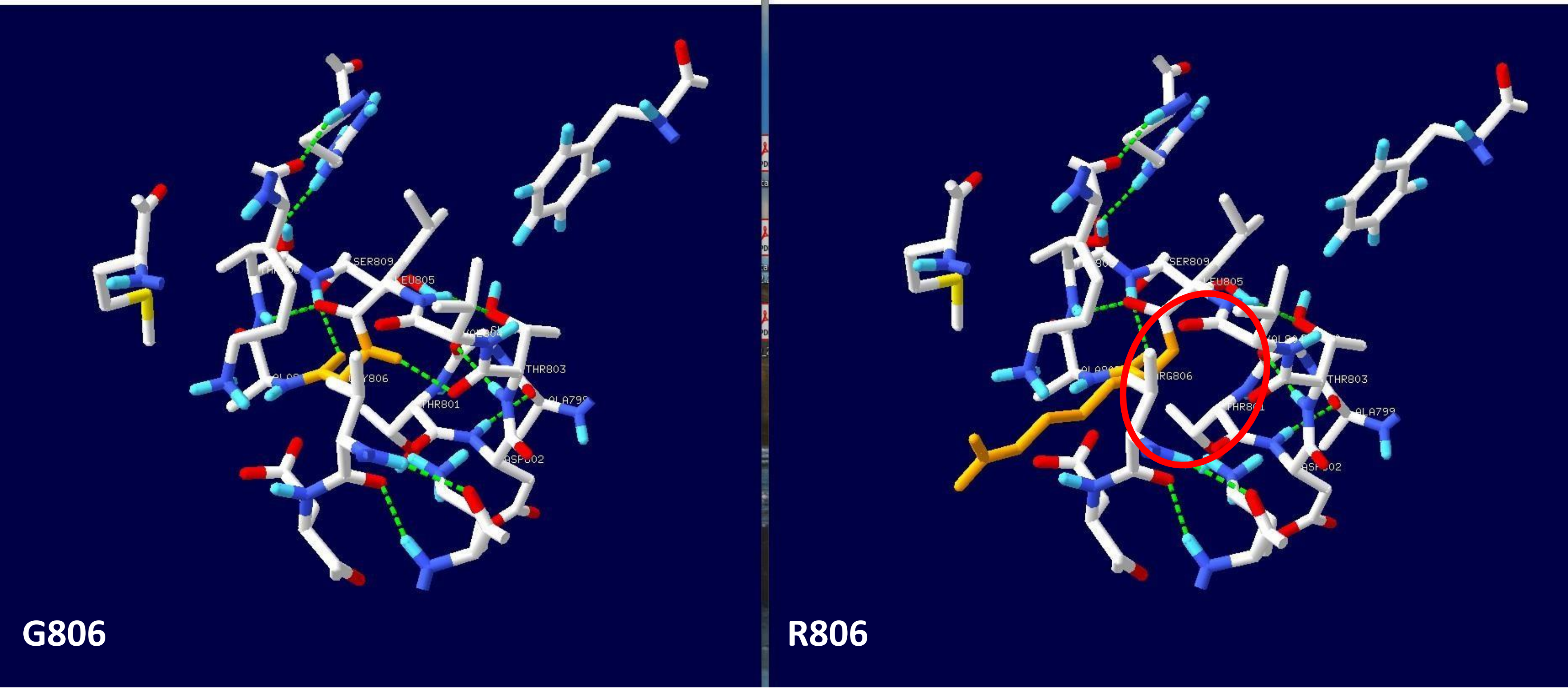

Result: Upon computing hydrogen bonds: R806 lost 1 hydrogen bond compared to G806

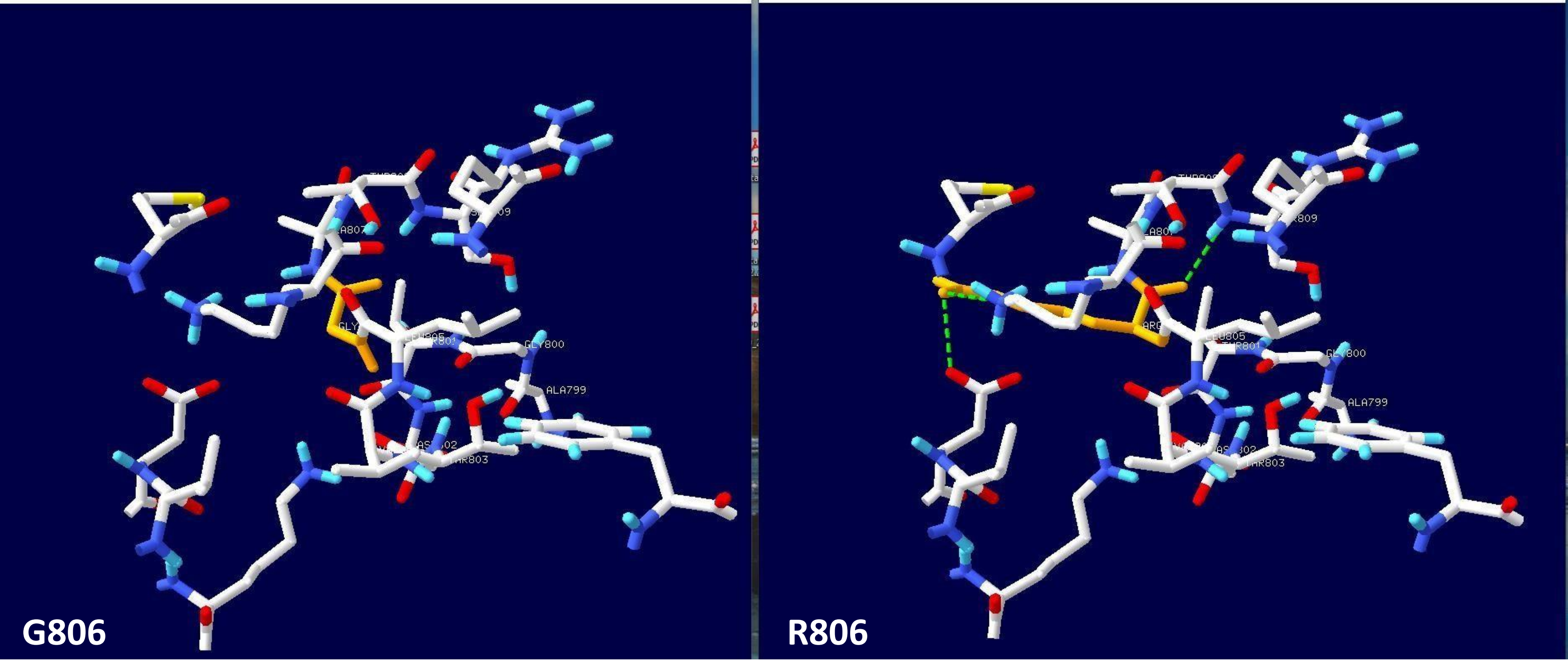

Result upon computing "mutation" effect:
C806 gained 3 hydrogen bonds that were absent in G806

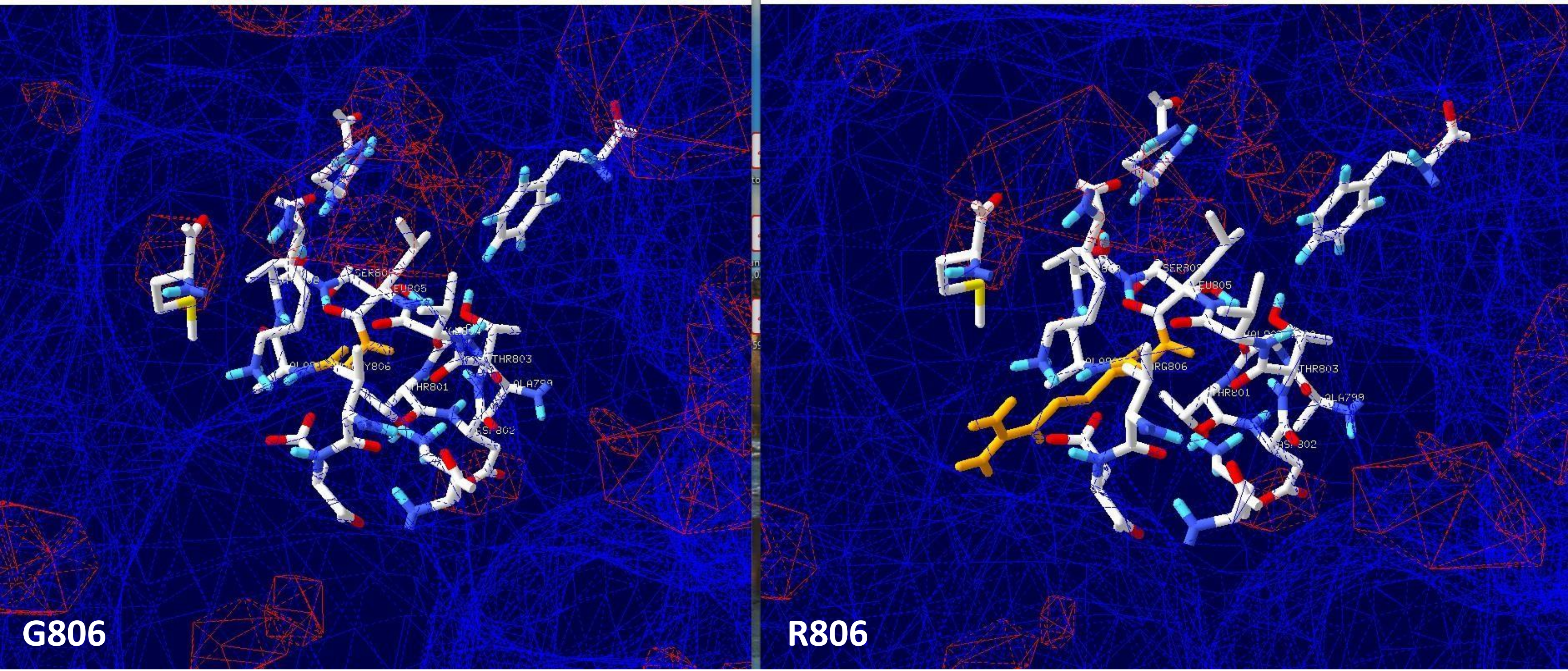

Result: Upon computing electrostatic potential: Basic area(Blue mesh) become lighter around the R806 residue compared to G806 residue

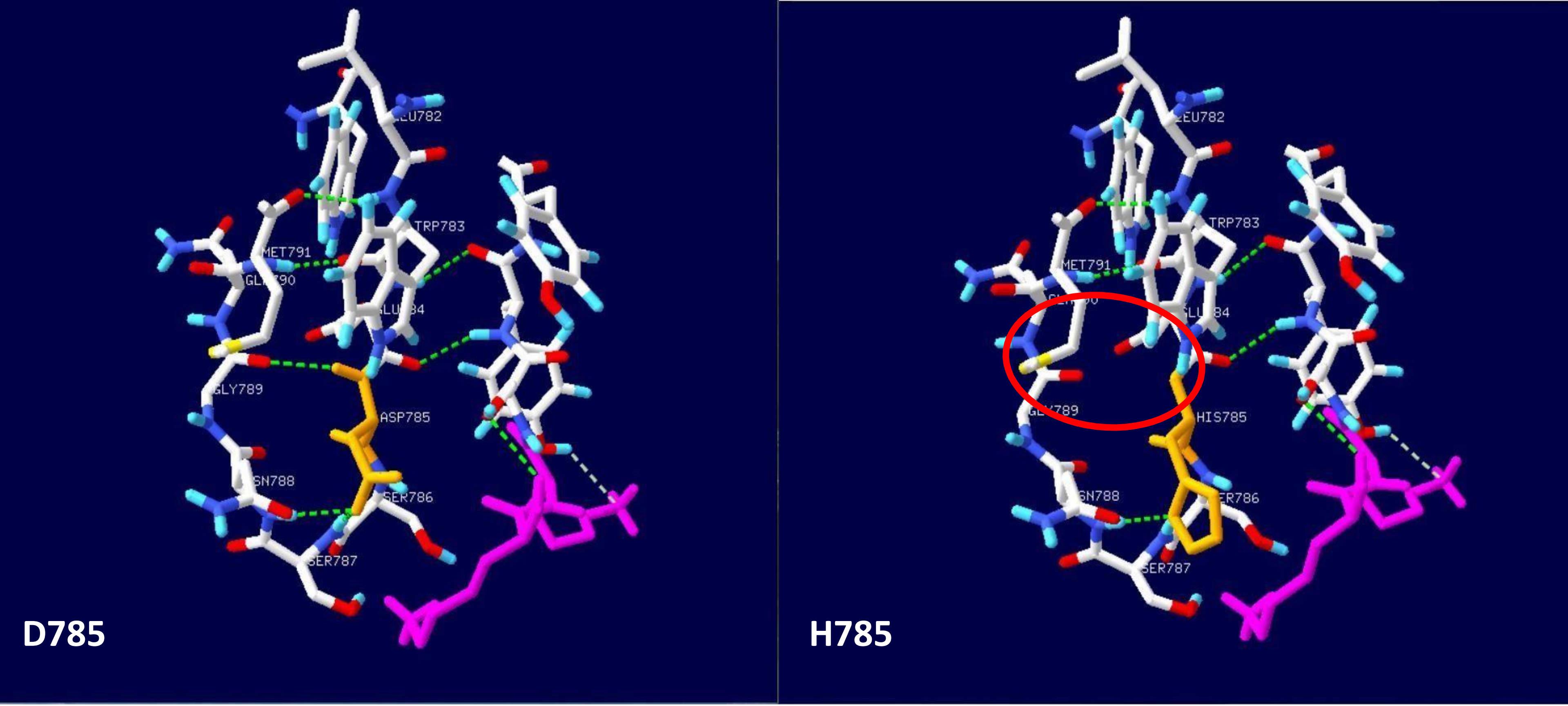

Result: Upon computing hydrogen bonds: H785 lost 1 hydrogen bond compared to D785

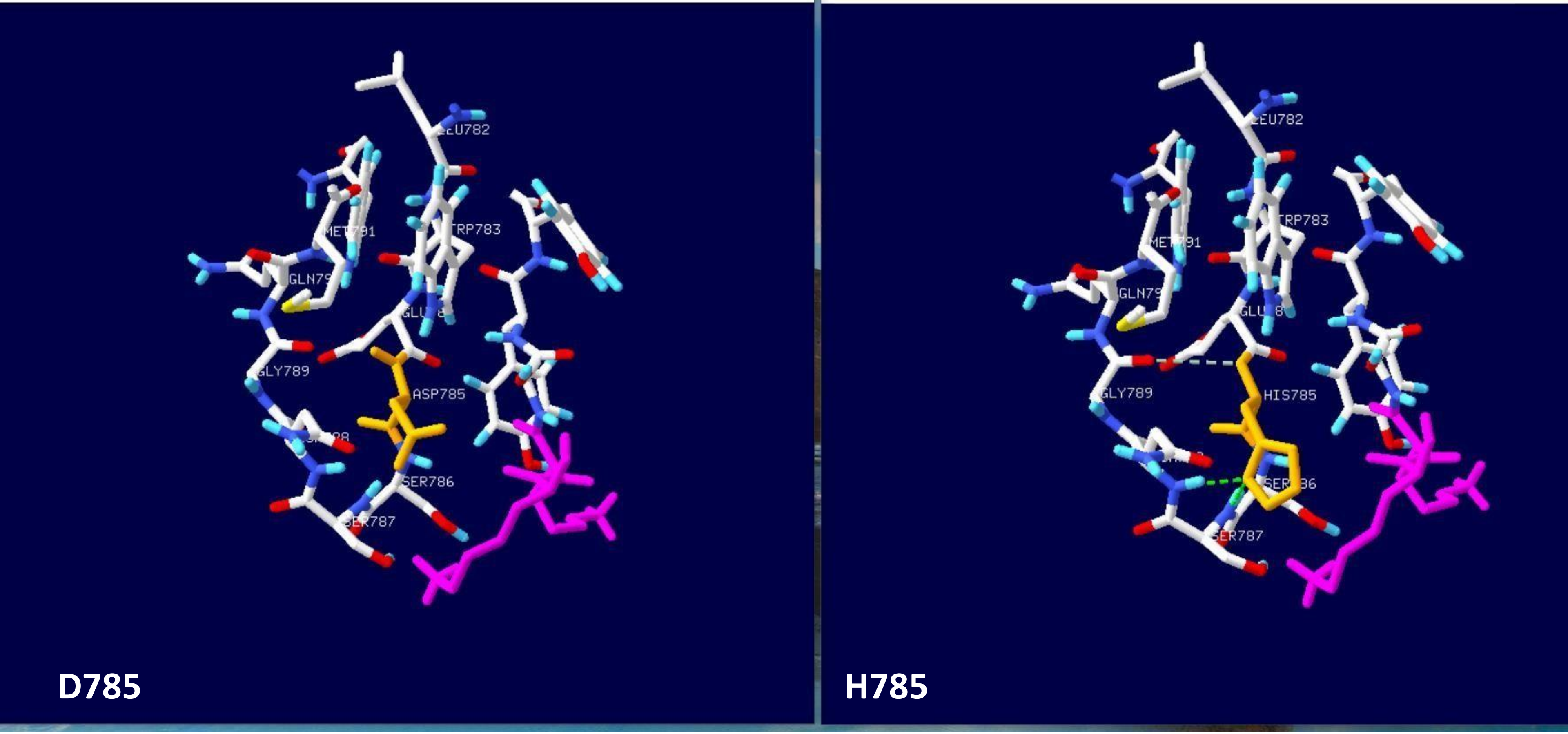

Result upon computing “mutation” effect:
H785 gained A Steric clash and 1 hydrogen bond that were absent in D785

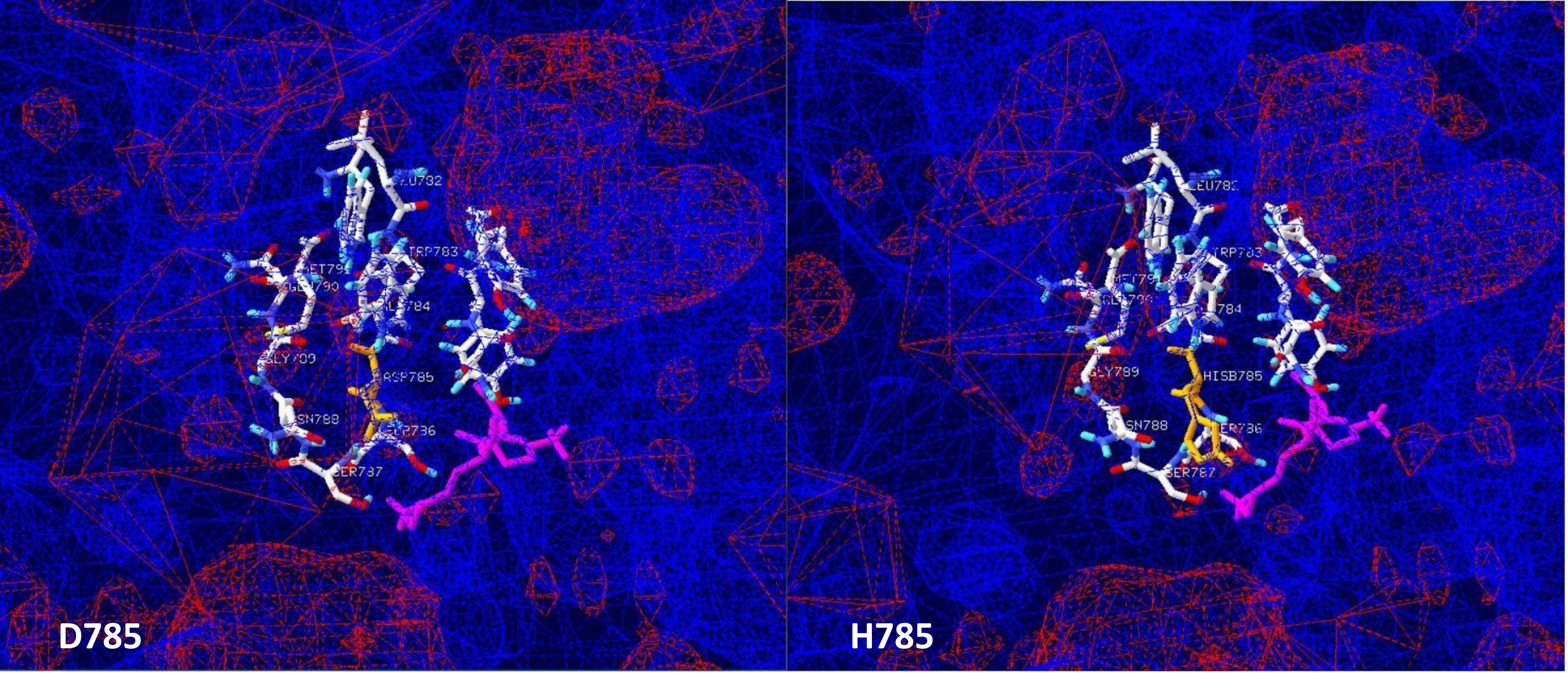

Result: Upon computing electrostatic potential: Acidic area (Red mesh) become less around the H785 residue compared to D785 residue

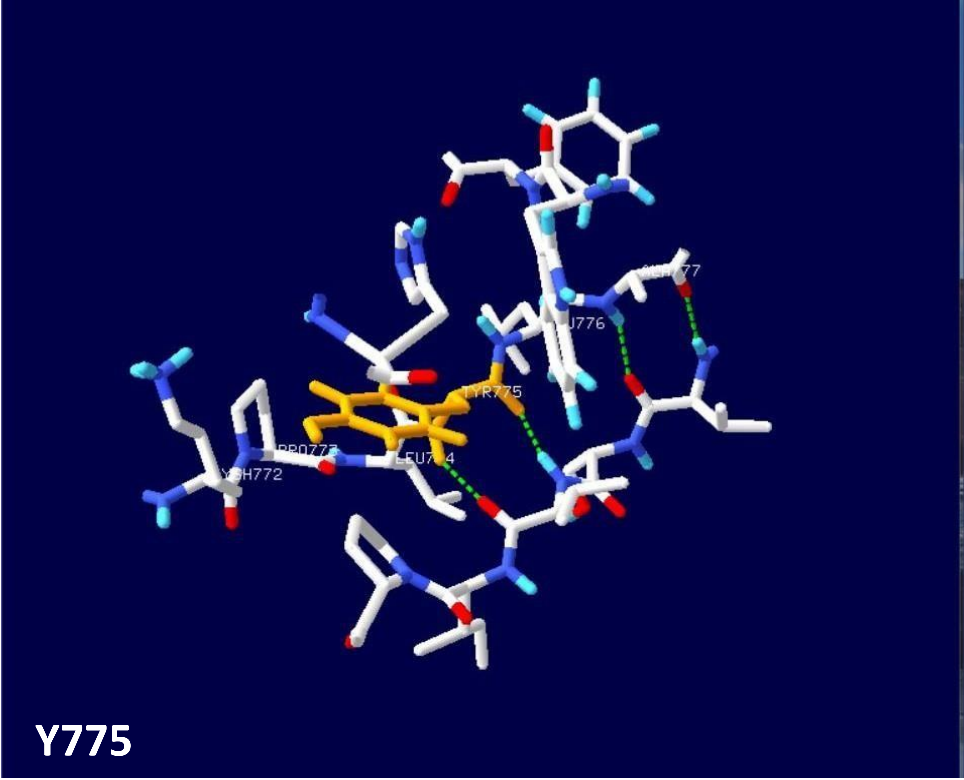

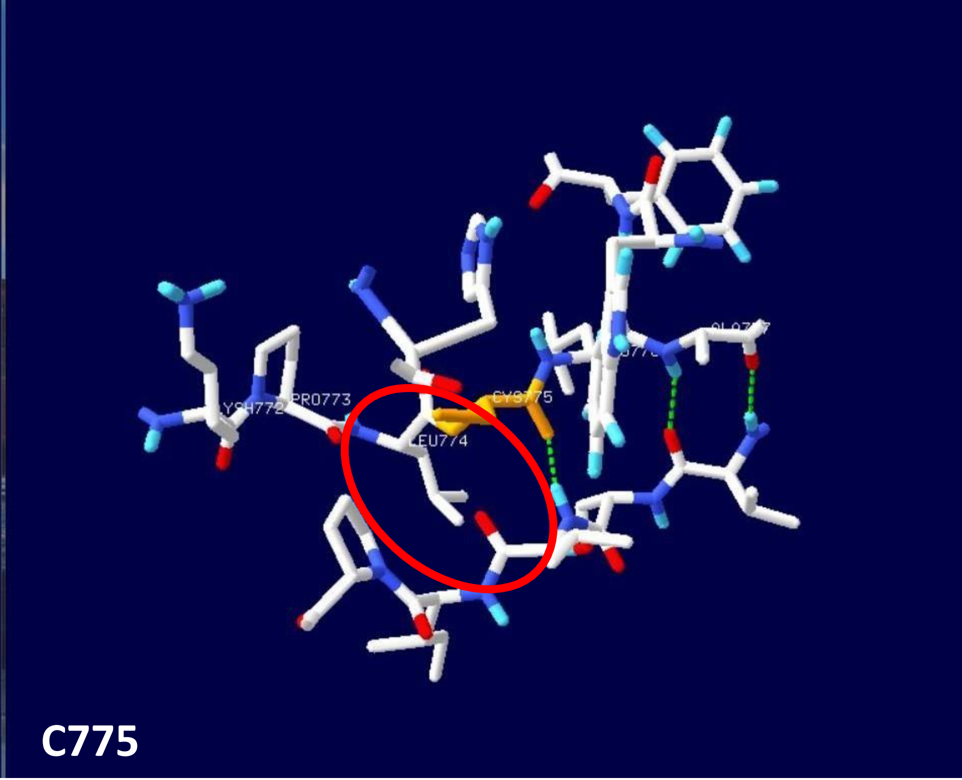

Result: Upon computing hydrogen bonds: C775 lost 1 hydrogen bond compared to Y775

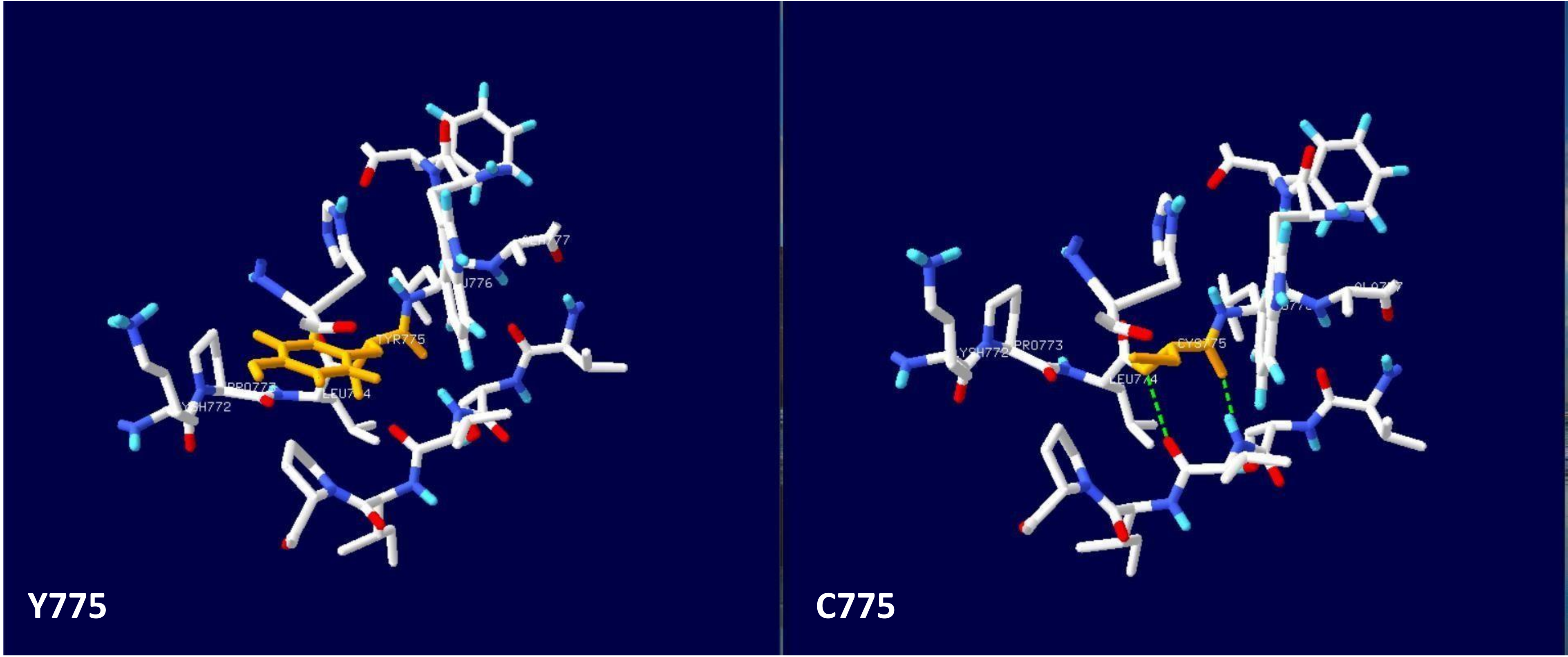

Result upon computing “mutation” effect:
C775 gained 2 hydrogen bonds that were absent in Y775

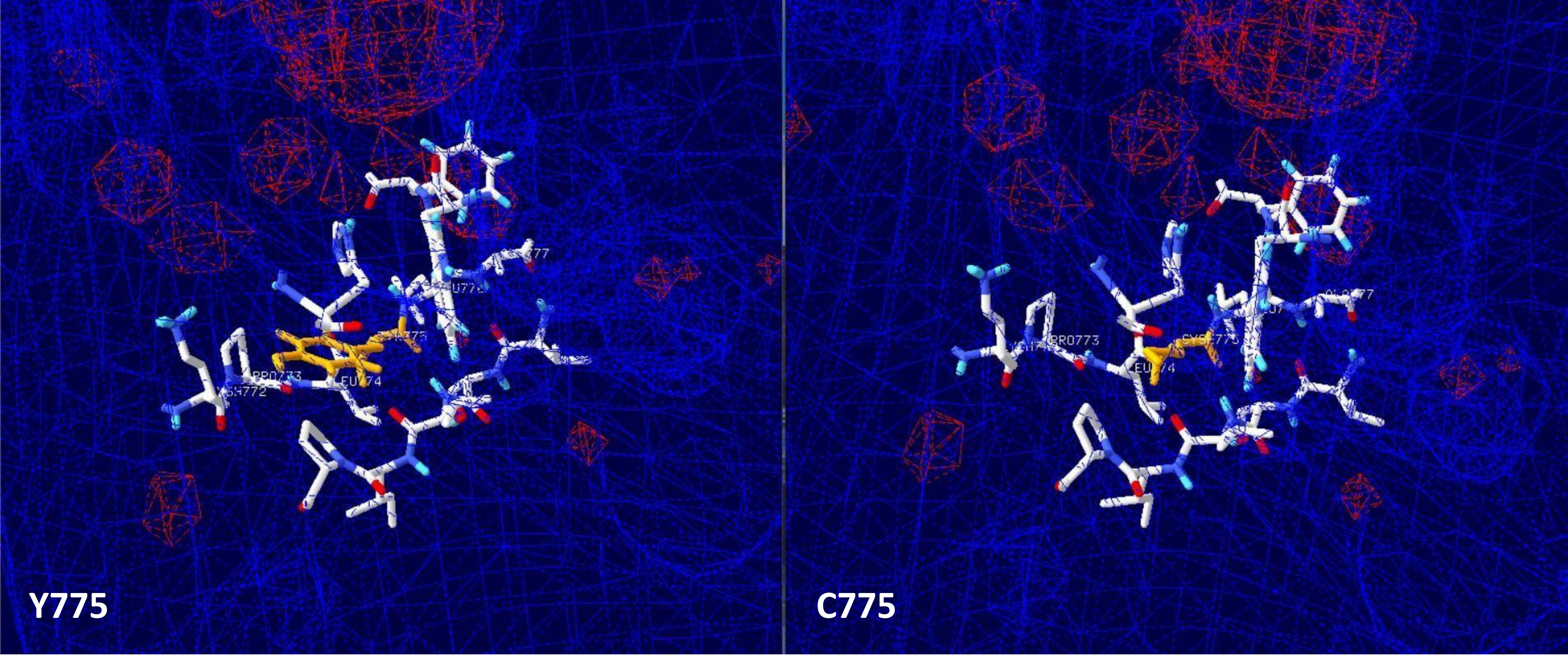

Result: Upon computing electrostatic potential: There is no change between C775 and Y775

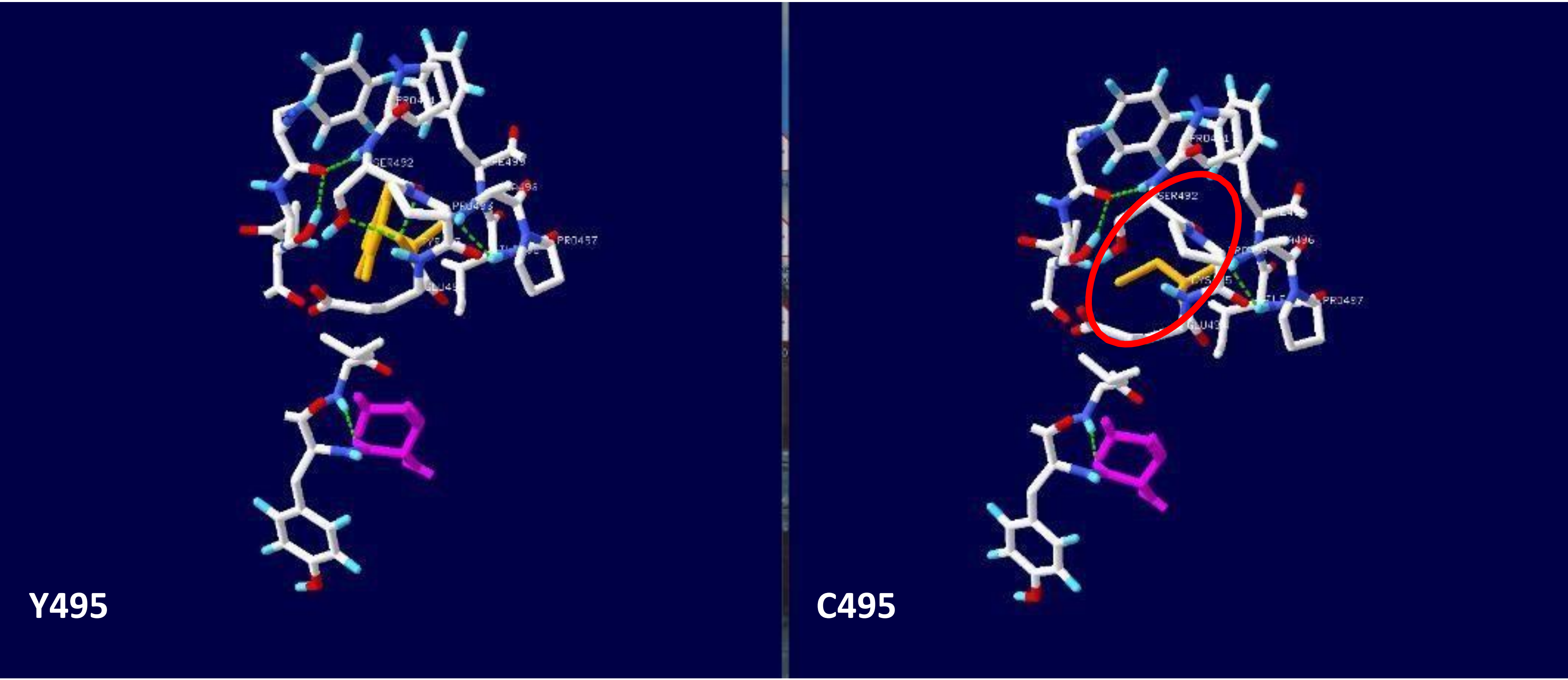

Result: Upon computing hydrogen bonds: C495 lost 2 hydrogen bonds compared to Y495

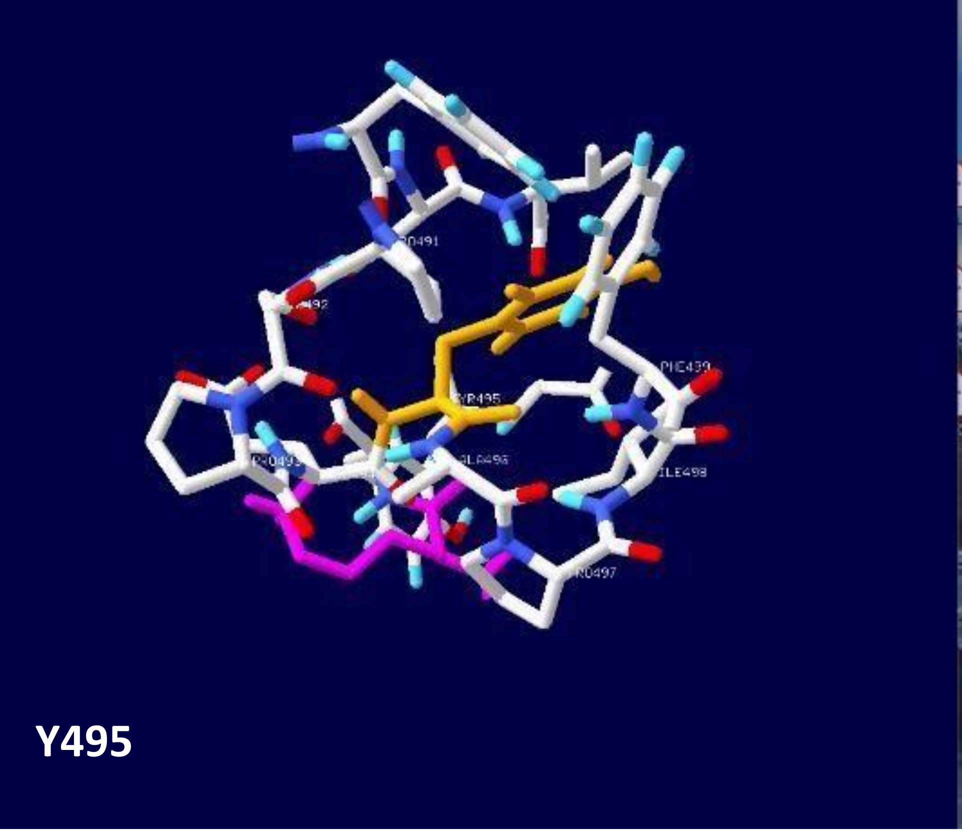

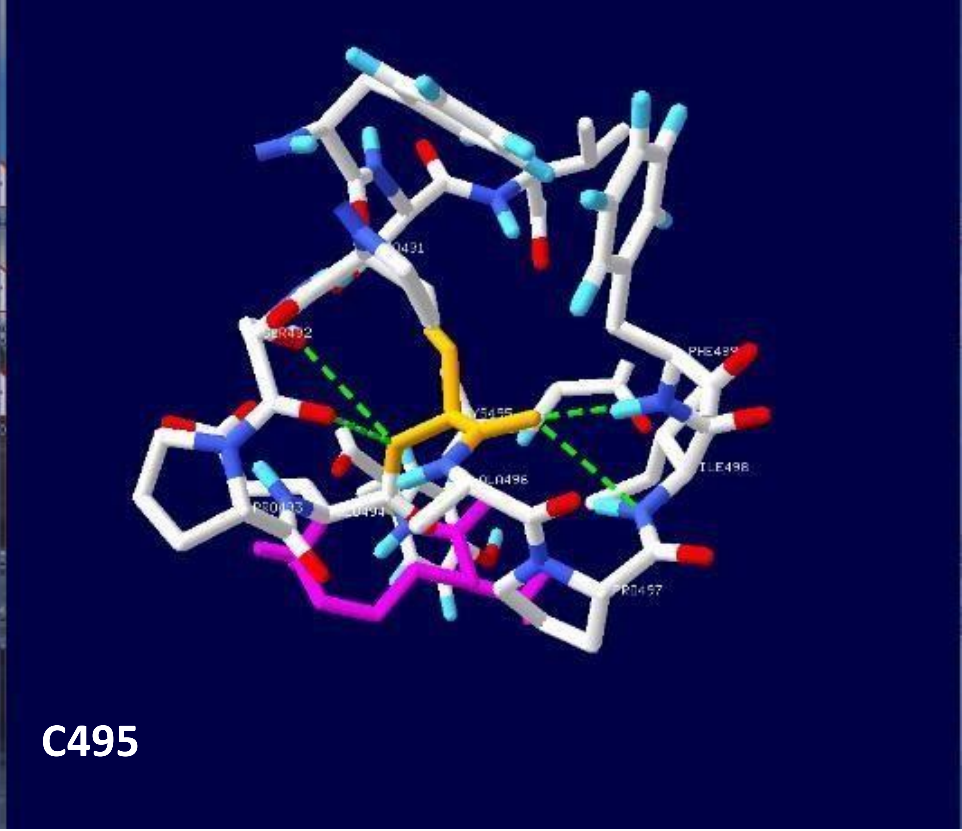

Result upon computing “mutation” effect:
C495 gained 4 hydrogen bonds that were absent in Y495

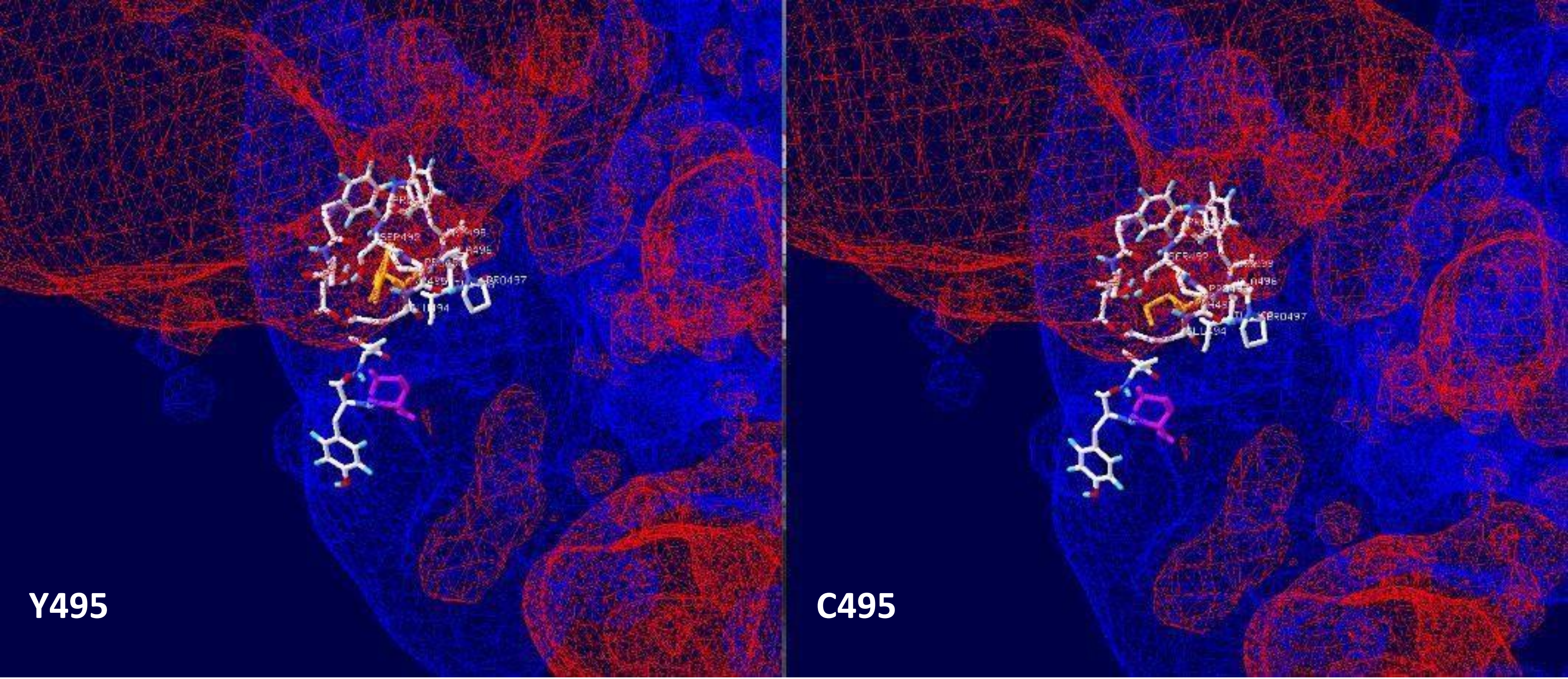

Result: Upon computing electrostatic potential: There is no change between C495 and Y495

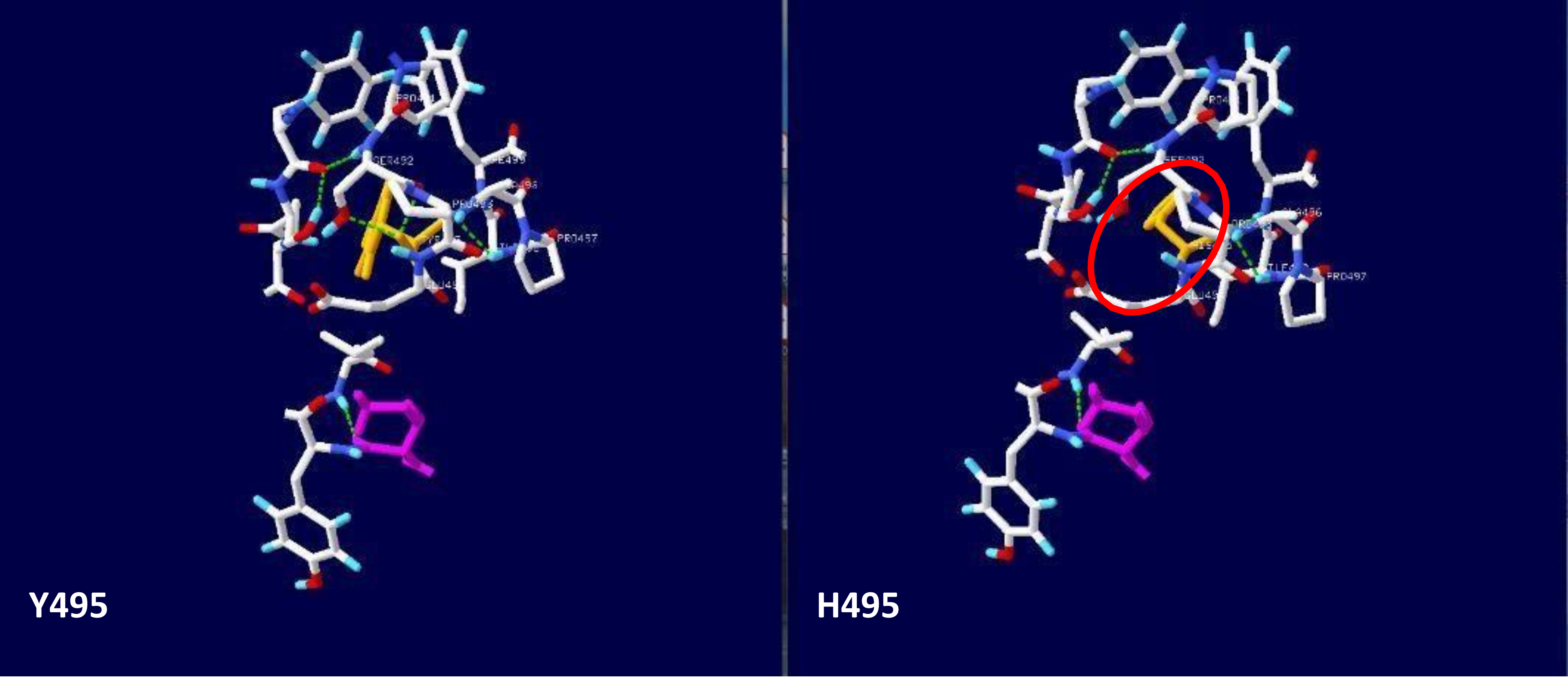

Result: Upon computing hydrogen bonds: H495 lost 2 hydrogen bonds compared to Y495

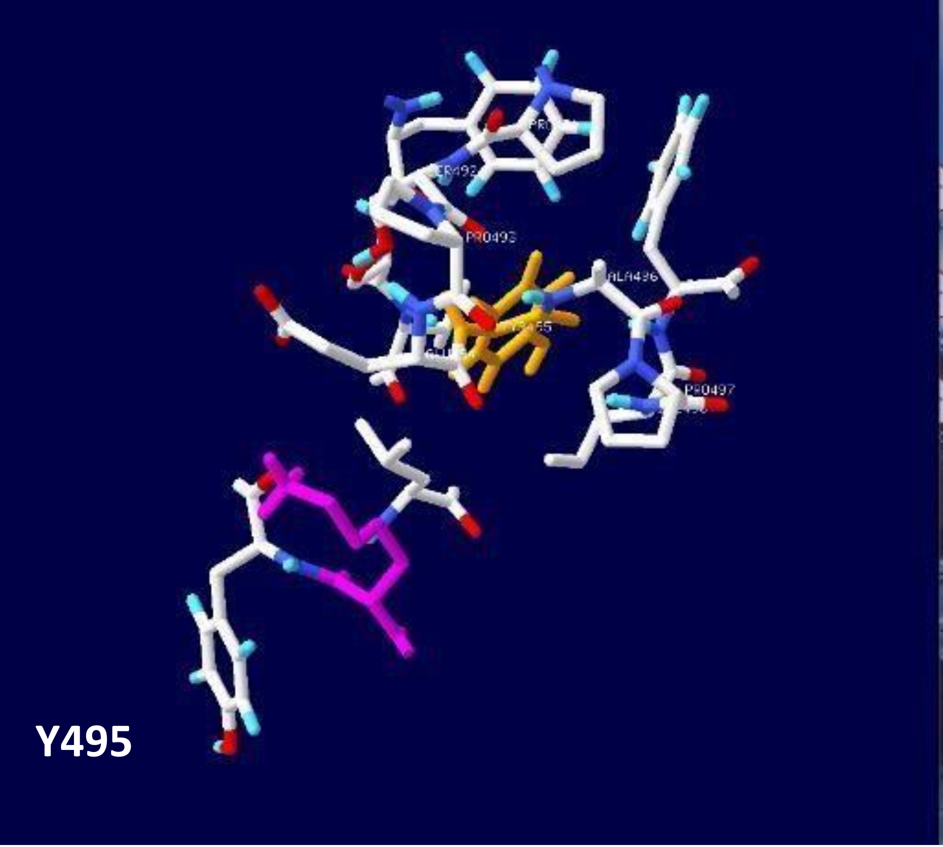

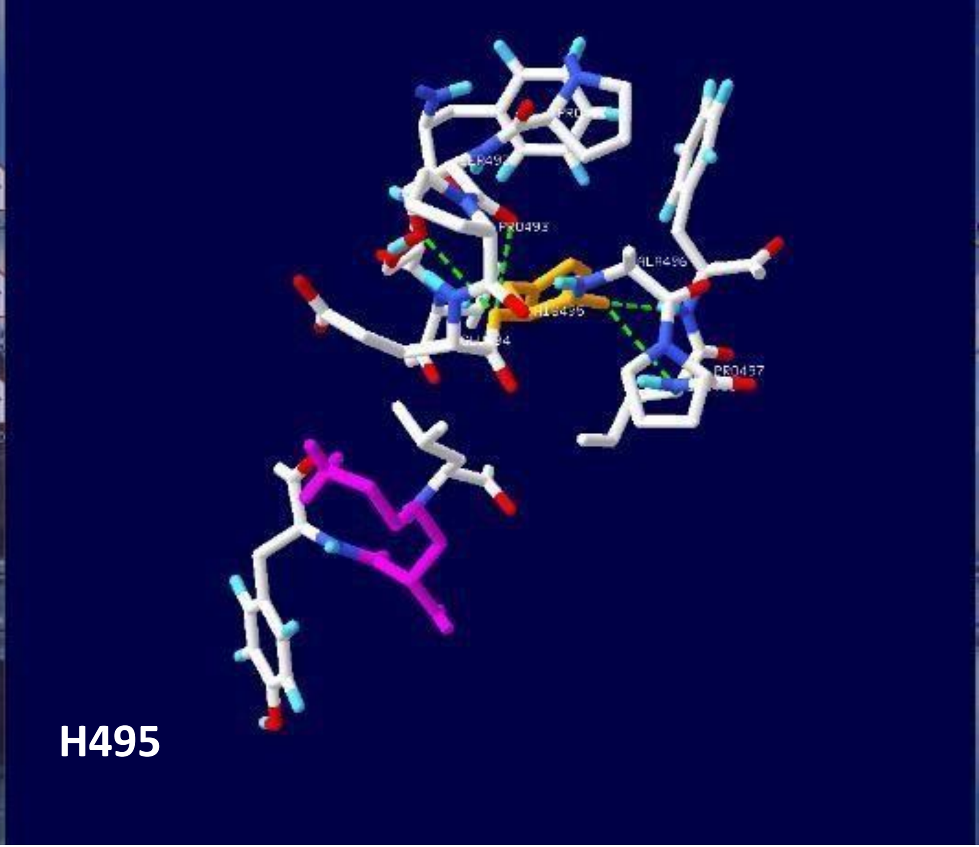

Result upon computing “mutation” effect:
H495 gained 4 hydrogen bonds that were absent in Y495

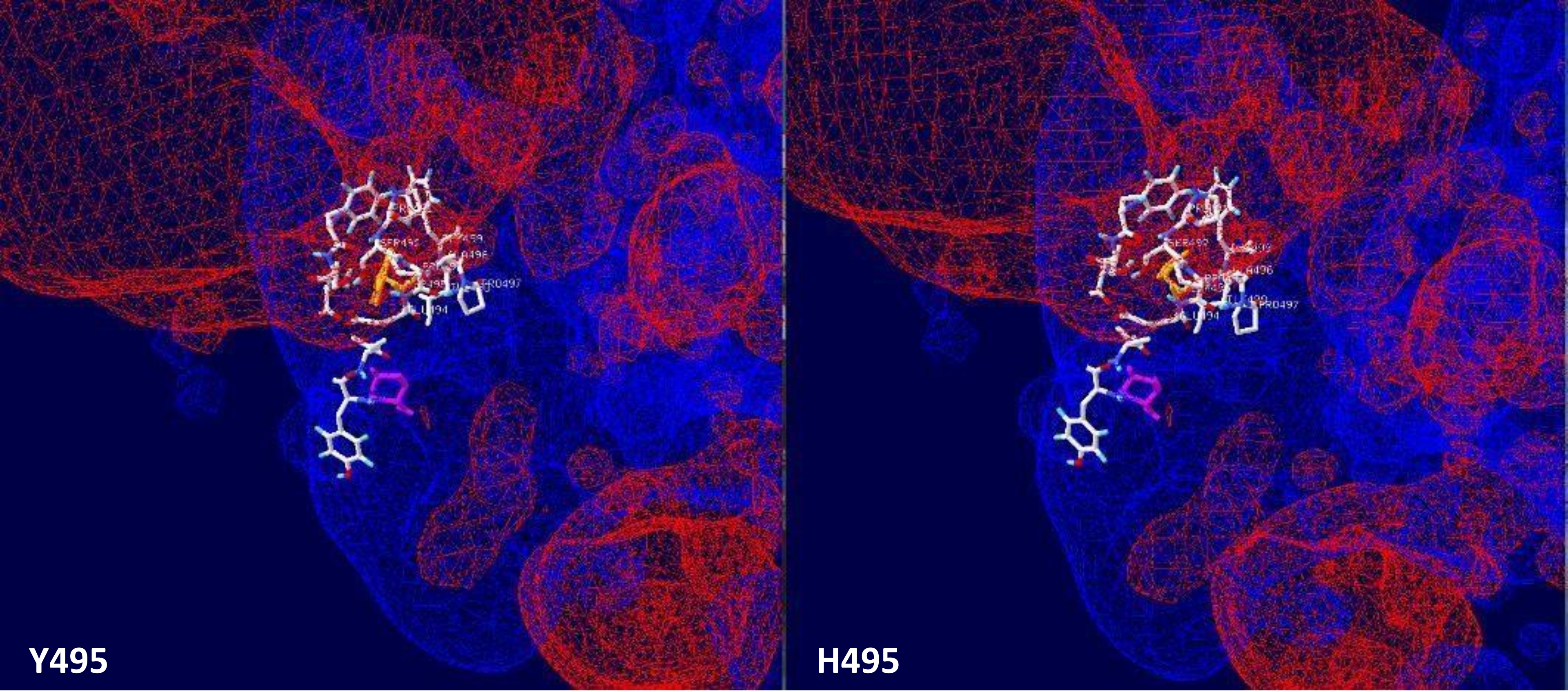

Result: Upon computing electrostatic potential: There is no change between H495 and Y495

Analysis of DNMT1 Gene Variants in Progression of Neural Tube Defects- an *insilico* to *invitro* approach

Susanta Sadhukhan[1†], Nirvika Paul[1†], Sudakshina Ghosh[2], Dinesh Munian[3], Kausik Ganguly[4], Krishnendu Ghosh[1], Mainak Sengupta[4], Madhusudan Das[1,*]

[1]Department of Zoology, University of Calcutta, 35 Ballygunge Circular Road, Kolkata-700019, India
[2]Department of Zoology, Vidyasagar College for Women, 39 Sankar Ghosh Lane, Kolkata-700006, India
[3]Department of Neonatology, Institute of Postgraduate Medical Education & Research, 244 Acharya Jagadish Chandra Bose Road, Kolkata, 700020, India
[4]Department of Genetics, University of Calcutta, 35 Ballygunge Circular Road, Kolkata-700019, India

[*]Corresponding author at the Dept. of Zoology, University of Calcutta:madhuzoo@yahoo.com/mdzoo@caluniv.ac.in (mail id)
†Authors contributed equally

Supplementary Table 1: Results of *in silico* analyses of non-synonymous variants (non-syn SNVs) of *DNMT1*. Results are shown for those non-syn SNVs only for which “deleterious”, “damaging” or “disease” causing effects were seen.

Supplementary Table 1A: List of non-synonymous single nucleotide variants (Non-syn-SNVs) for which “Deleterious” resultswere predicted using PROVEAN

dbSNP ID	aa substitution 1	PROVEAN Results	
rs1203183469	G1589C	-7.54	Deleterious
rs750979984	A1587T	-3.14	Deleterious
rs1248265917	P1582L	-8.38	Deleterious
rs930531589	H1573Q	-6.57	Deleterious
rs1301210022	K1572N	-3.91	Deleterious
rs750769453	I1569T	-4.02	Deleterious
rs747467776	R1564W	-5.76	Deleterious
rs1170573036	G1558S	-5.12	Deleterious
rs1388362405	S1556F	-5.02	Deleterious
rs1461695373	R1555C	-6.82	Deleterious
rs1393111051	R1551Q	-3.41	Deleterious
rs1388371493	R1551W	-6.82	Deleterious
rs1299212888	V1548M	-2.53	Deleterious
rs919486255	V1539M	-2.56	Deleterious
rs1316990553	N1529I	-7.42	Deleterious
rs1441106688	G1537S	-5.12	Deleterious
rs1316848811	E1518K	-2.96	Deleterious
rs1236455214	D1486H	-4.98	Deleterious
rs746932608	G1482V	-5.34	Deleterious
rs768764350	G1482S	-4.25	Deleterious
rs769347864	S1477F	-4.22	Deleterious
rs745789285	R1473H	-4.46	Deleterious
rs772660711	L1472R	-3.81	Deleterious
rs1363710824	S1469C	-2.67	Deleterious
rs1167554550	S1467I	-4.85	Deleterious
rs770535972	R1466H	-2.56	Deleterious
rs147984942	R1466C	-3.59	Deleterious
rs1368186167	G1465S	-4.51	Deleterious
rs142647321	H1460Q	-2.71	Deleterious
rs765347113	H1460R	-2.94	Deleterious
rs1274835966	H1459Q	-5.13	Deleterious
rs1265513327	H1459R	-5.47	Deleterious
rs1361579493	T1458I	-3	Deleterious

rs1415174324	T1450I	-4.29	Deleterious
rs754833277	G1465S	-4.51	Deleterious
rs1387794444	S1447L	-4.22	Deleterious
rs769675693	R1445W	-4.82	Deleterious
rs1341093379	V1444A	-3.32	Deleterious
rs1488470434	R1453C	-4.25	Deleterious
rs770571074	G1449R	-6.64	Deleterious
rs774010132	P1432S	-3.87	Deleterious
rs1442538027	P1429L	-9	Deleterious
rs1255037718	H1427N	-4.21	Deleterious
rs139918621	R1426G	-2.71	Deleterious
rs1183358759	R1424H	-4.53	Deleterious
rs1365871222	R1424C	-7.22	Deleterious
rs555878888	A1423D	-4.56	Deleterious
rs1031456207	M1417V	-3.09	Deleterious
rs1375081003	D1416N	-2.6	Deleterious
rs1386402643	C1414S	-8.5	Deleterious
rs1450243310	D1411N	-4.17	Deleterious
rs1241729369	G1402D	-4.78	Deleterious
rs1442803580	R1401Q	-3.62	Deleterious
rs1323856557	R1401W	-7.14	Deleterious
rs766558026	F1396L	-5.49	Deleterious
rs1324326054	F1396S	-7.3	Deleterious
rs1226189469	W1395C	-4.35	Deleterious
rs886054127	I1386F	-2.57	Deleterious
rs375225009	S1382L	-3.82	Deleterious
rs1454184730	E1376G	-4.28	Deleterious
rs1382078791	P1375R	-8.59	Deleterious
rs1356963122	D1373N	-4.77	Deleterious
rs1292991293	S1372F	-4.53	Deleterious
rs1259943763	S1359L	-5.6	Deleterious
rs771611874	S1359T	-2.8	Deleterious
rs774538556	S1358I	-2.56	Deleterious
rs1265788604	N1353S	-3.24	Deleterious
rs1164503433	N1353H	-3.51	Deleterious
rs141791913	A1338V	-2.75	Deleterious
rs1254786385	P1336L	-3.86	Deleterious
rs867399027	P1328L	-9.06	Deleterious
rs1328566140	E1322K	-2.8	Deleterious
rs757703243	G1321E	-6.67	Deleterious
rs766051225	A1318V	-3.57	Deleterious
rs779967013	A1318T	-3.47	Deleterious
rs1197552228	G1321S	-5.06	Deleterious
rs767041788	G1297S	-5.18	Deleterious
rs1442277728	C1294S	-8.98	Deleterious
rs1182970957	C1294G	-10.83	Deleterious
rs564106859	R1289H	-3.36	Deleterious
rs200312526	R1289C	-5.49	Deleterious
rs1010743136	R1285H	-3.65	Deleterious
rs755492225	R1285C	-5.6	Deleterious

rs748262646	R1276H	-3.35	Deleterious
rs1343782138	E1266D	-2.88	Deleterious
rs535946725	F1262L	-4.9	Deleterious
rs1052868434	R1261W	-5.85	Deleterious
rs1296808828	R1259W	-7.47	Deleterious
rs1168306248	D1256N	-4.7	Deleterious
rs1408962462	V1248M	-2.62	Deleterious
rs1085307800	Y1240C	-8.33	Deleterious
rs780156714	R1238H	-4.33	Deleterious
rs746456488	R1238C	-6.87	Deleterious
rs1290055474	S1253L	-5.76	Deleterious
rs768280180	N1236S	-4.63	Deleterious
rs1312709316	G1222S	-5.52	Deleterious
rs1353293786	D1216N	-3.26	Deleterious
rs759915904	G1231R	-7.37	Deleterious
rs767953016	P1212A	-7.67	Deleterious
rs1269778997	R1210W	-4.96	Deleterious
rs1255396898	G1208D	-6.15	Deleterious
rs764137405	G1208S	-5.23	Deleterious
rs142648642	T1204I	-2.93	Deleterious
rs1367702161	T1185I	-3.42	Deleterious
rs1255153711	R1178W	-6.74	Deleterious
rs1434985283	A1176V	-3.57	Deleterious
rs772419584	A1173V	-3.84	Deleterious
rs1193652618	P1172R	-5.47	Deleterious
rs1412363660	A1166T	-3.84	Deleterious
rs764541314	T1163M	-3.41	Deleterious
rs151305495	Q1157H	-4.33	Deleterious
rs754742754	V1144L	-2.54	Deleterious
rs767421908	R1140Q	-3.18	Deleterious
rs375976847	P1127L	-3.4	Deleterious
rs770370709	G1118D	-3.79	Deleterious
rs1284939504	G1116R	-5.09	Deleterious
rs200950656	R1104C	-2.64	Deleterious
rs150331990	H1102R	-3.19	Deleterious
rs759653796	P1099L	-7.03	Deleterious
rs755865204	P1099S	-6.24	Deleterious
rs1479104782	F1096L	-5.09	Deleterious
rs377078524	R1082H	-3.67	Deleterious
rs1316623288	R1082C	-6.44	Deleterious
rs747265409	G1079S	-4.67	Deleterious
rs950686369	G1078C	-3.95	Deleterious
rs779489678	R1059H	-2.73	Deleterious
rs140852137	R1059C	-5.37	Deleterious
rs776374384	F1053L	-3.46	Deleterious
rs1478047497	V1051G	-6.25	Deleterious
rs1160158444	Y1043C	-8.47	Deleterious
rs767308840	N1040S	-4.8	Deleterious
rs144533539	R1017W	-5.32	Deleterious
rs373940840	N1026S	-4.24	Deleterious

rs1248096623	K1020T	-4.63	Deleterious
rs1199169323	F1000S	-5.1	Deleterious
rs376854079	I996M	-2.63	Deleterious
rs1167927296	I996T	-4.58	Deleterious
rs776149246	Y991C	-5.77	Deleterious
rs747897637	E989K	-3.09	Deleterious
rs775590756	P970A	-7.47	Deleterious
rs1056948381	Y969H	-4.8	Deleterious
rs753992945	D965A	-7.2	Deleterious
rs1489509959	D965N	-3.9	Deleterious
rs895501426	E962G	-3.39	Deleterious
rs978444139	F946L	-4.76	Deleterious
rs780533354	F946I	-4.76	Deleterious
rs755712065	V939L	-2.73	Deleterious
rs977832207	G930V	-6.28	Deleterious
rs777416084	G930S	-3.65	Deleterious
rs1389160994	Y923S	-3.92	Deleterious
rs762002107	L922P	-3.22	Deleterious
rs550380640	R920G	-3.39	Deleterious
rs767511749	E915K	-2.52	Deleterious
rs1453506357	Q913L	-2.62	Deleterious
rs756944531	R898H	-3.37	Deleterious
rs745753378	R898C	-6.28	Deleterious
rs1460029697	S895R	-3.31	Deleterious
rs768990499	F892C	-5.79	Deleterious
rs1379893711	P880S	-4.41	Deleterious
rs771879227	P879L	-3.42	Deleterious
rs775305891	P879S	-4.11	Deleterious
rs1160065459	S878F	-2.74	Deleterious
rs1303994790	A874V	-3.65	Deleterious
rs367681882	D870H	-4.17	Deleterious
rs750081202	Q866R	-2.62	Deleterious
rs1314966532	F864L	-5.36	Deleterious
rs753140952	G860E	-4.94	Deleterious
rs62621087	G860R	-5.14	Deleterious
rs749258509	D859N	-3.37	Deleterious
rs571299133	D858G	-4.05	Deleterious
rs868584117	S840C	-2.98	Deleterious
rs1228333897	P811L	-8.71	Deleterious
rs963570572	P811S	-6.53	Deleterious
rs140993011	T808M	-3.24	Deleterious
rs183555527	G806R	-7.13	Deleterious
rs988229673	A794G	-2.52	Deleterious
rs1244845928	D785H	-3.46	Deleterious
rs1181332562	Y775C	-5.11	Deleterious
rs113497353	L774P	-4.05	Deleterious
rs1201583942	P773L	-8.27	Deleterious
rs865989923	S786F	-2.76	Deleterious
rs1313976937	S786C	-2.5	Deleterious
rs1330899035	C762G	-4.93	Deleterious

rs370056750	D761E	-2.99	Deleterious
rs1346601061	E758G	-3.33	Deleterious
rs1251285527	I752T	-3.19	Deleterious
rs764389789	D741G	-3.68	Deleterious
rs1381758934	G735R	-5.7	Deleterious
rs752326785	D706G	-4.7	Deleterious
rs1254979538	D706N	-2.76	Deleterious
rs1336324296	N693S	-4.04	Deleterious
rs766157757	R689W	-7.24	Deleterious
rs1029809799	S682C	-3.29	Deleterious
rs767317190	F676Y	-2.61	Deleterious
rs760173233	G654S	-4.96	Deleterious
rs763570334	R651W	-4.81	Deleterious
rs753503985	R650H	-4.3	Deleterious
rs761410935	R650C	-6.5	Deleterious
rs1357755689	D639V	-2.51	Deleterious
rs397509391	V590F	-4.08	Deleterious
rs397509393	G589E	-7.21	Deleterious
rs775941265	M581T	-4.64	Deleterious
rs760952579	P579S	-6.28	Deleterious
rs762421501	T578I	-2.91	Deleterious
rs959058396	D569H	-4.11	Deleterious
rs397509392	A554G	-3.61	Deleterious
rs1003447106	A554T	-3.61	Deleterious
rs768869946	D548N	-4.01	Deleterious
rs375474222	R544C	-3.51	Deleterious
rs1315979941	G539V	-3.26	Deleterious
rs763014417	T534M	-2.54	Deleterious
rs556213169	T533I	-4.11	Deleterious
rs1269876059	I531F	-2.62	Deleterious
rs774523299	T523I	-3.17	Deleterious
rs760542365	F515V	-5.47	Deleterious
rs1405357614	P497L	-7.76	Deleterious
rs199473690	Y495C	-8.11	Deleterious
rs199473692	Y495H	-4.51	Deleterious
rs1190635928	E473K	-3.04	Deleterious
rs374027926	E463A	-3.81	Deleterious
rs1308265840	P447S	-3.95	Deleterious
rs1403539644	D444H	-4.13	Deleterious
rs762835601	F434L	-3.62	Deleterious
rs1181209213	D423N	-4.34	Deleterious
rs758850158	G417S	-2.94	Deleterious
rs1294787183	Q358H	-3.91	Deleterious
rs1333537251	P350L	-3.49	Deleterious

Supplementary Table 1B: List of Non-syn-SNVsfor which "Damaging" results were predicted using SIFT

dbSNP ID	aa substitution 1	SIFT Results

rs770148933	E1612G	0.008	Damaging	3.48
rs1203183469	G1589C	0	Damaging	2.84
rs750979984	A1587T	0.012	Damaging	2.84
rs1248265917	P1582L	0	Damaging	2.84
rs930531589	H1573Q	0.001	Damaging	2.84
rs1301210022	K1572N	0.001	Damaging	2.84
rs750769453	I1569T	0.011	Damaging	2.84
rs747467776	R1564W	0.001	Damaging	2.84
rs1170573036	G1558S	0	Damaging	2.84
rs1388362405	S1556F	0	Damaging	2.84
rs1461695373	R1555C	0	Damaging	2.84
rs1393111051	R1551Q	0	Damaging	2.84
rs1388371493	R1551W	0	Damaging	2.84
rs1299212888	V1548M	0.001	Damaging	2.84
rs1305204736	V1547M	0.001	Damaging	2.84
rs919486255	V1539M	0.002	Damaging	2.84
rs1316990553	N1529I	0	Damaging	2.84
rs1441106688	G1537S	0	Damaging	2.84
rs1316848811	E1518K	0.007	Damaging	2.84
rs1236455214	D1486H	0.006	Damaging	3.01
rs746932608	G1482V	0	Damaging	3.01
rs768764350	G1482S	0.004	Damaging	3.01
rs768672451	E1480Q	0.011	Damaging	3.25
rs781301028	V1479M	0.003	Damaging	3.05
rs769347864	S1477F	0.001	Damaging	3.01
rs745789285	R1473H	0.001	Damaging	3.01
rs772660711	L1472R	0.009	Damaging	3.04
rs866729887	L1472F	0.032	Damaging	3.04
rs536916632	A1471T	0.015	Damaging	3.04
rs1405528301	S1468N	0.05	Damaging	3.01
rs1167554550	S1467I	0.001	Damaging	3.01
rs1368186167	G1465S	0.023	Damaging	3.01
rs755902999	R1462S	0.02	Damaging	3.02
rs1274835966	H1459Q	0.009	Damaging	3.01
rs1265513327	H1459R	0.011	Damaging	3.01
rs1361579493	T1458I	0.015	Damaging	3.01
rs1415174324	T1450I	0.025	Damaging	2.9
rs754833277	G1465S	0.023	Damaging	3.01
rs1387794444	S1447L	0.014	Damaging	2.84
rs769675693	R1445W	0.001	Damaging	2.84
rs1341093379	V1444A	0.003	Damaging	2.84
rs1488470434	R1453C	0.003	Damaging	2.99
rs770571074	G1449R	0.001	Damaging	2.84
rs1442538027	P1429L	0	Damaging	2.84
rs1183358759	R1424H	0	Damaging	2.84
rs1365871222	R1424C	0	Damaging	2.84
rs555878888	A1423D	0.009	Damaging	2.84
rs1031456207	M1417V	0.002	Damaging	2.84
rs1450243310	D1411N	0.045	Damaging	2.85
rs374047326	A1419T	0.013	Damaging	2.85

rs1241729369	G1402D	0.027	Damaging	2.85
rs1442803580	R1401Q	0.002	Damaging	2.85
rs1323856557	R1401W	0	Damaging	2.85
rs766558026	F1396L	0.028	Damaging	2.86
rs1324326054	F1396S	0	Damaging	2.86
rs1226189469	W1395C	0.011	Damaging	2.85
rs886054127	I1386F	0.008	Damaging	2.86
rs375225009	S1382L	0.004	Damaging	2.85
rs1382078791	P1375R	0.001	Damaging	2.85
rs1356963122	D1373N	0.001	Damaging	2.85
rs1259943763	S1359L	0.002	Damaging	2.85
rs771611874	S1359T	0.024	Damaging	2.85
rs1164503433	N1353H	0.027	Damaging	2.83
rs765098980	V1343M	0.002	Damaging	2.83
rs765793648	A1335D	0.01	Damaging	2.85
rs867399027	P1328L	0	Damaging	2.85
rs1328566140	E1322K	0.008	Damaging	2.85
rs766051225	A1318V	0.001	Damaging	2.85
rs779967013	A1318T	0.001	Damaging	2.85
rs1044312254	I1315M	0.001	Damaging	2.85
rs1197552228	G1321S	0.01	Damaging	2.85
rs767041788	G1297S	0.035	Damaging	2.85
rs1442277728	C1294S	0.002	Damaging	2.85
rs1182970957	C1294G	0.004	Damaging	2.85
rs564106859	R1289H	0.003	Damaging	2.85
rs200312526	R1289C	0.001	Damaging	2.85
rs1010743136	R1285H	0.012	Damaging	2.85
rs755492225	R1285C	0.031	Damaging	2.85
rs748262646	R1276H	0.005	Damaging	2.85
rs1235846614	V1268I	0.003	Damaging	2.85
rs1343782138	E1266D	0	Damaging	2.85
rs1178617228	L1280I	0.008	Damaging	2.85
rs774073234	R1261Q	0.01	Damaging	2.85
rs1052868434	R1261W	0.001	Damaging	2.85
rs1296808828	R1259W	0	Damaging	2.85
rs1168306248	D1256N	0.001	Damaging	2.85
rs1408962462	V1248M	0.001	Damaging	2.85
rs1085307800	Y1240C	0.001	Damaging	2.86
rs780156714	R1238H	0.007	Damaging	2.86
rs746456488	R1238C	0.002	Damaging	2.86
rs1290055474	S1253L	0	Damaging	2.85
rs768280180	N1236S	0.023	Damaging	2.86
rs1312709316	G1222S	0.003	Damaging	2.85
rs1353293786	D1216N	0.004	Damaging	2.85
rs759915904	G1231R	0.001	Damaging	2.85
rs767953016	P1212A	0.002	Damaging	2.85
rs1269778997	R1210W	0.004	Damaging	2.86
rs1255153711	R1178W	0	Damaging	2.85
rs1434985283	A1176V	0.001	Damaging	2.85
rs772419584	A1173V	0.001	Damaging	2.85

rs1193652618	P1172R	0.001	Damaging	2.85
rs1412363660	A1166T	0	Damaging	2.85
rs764541314	T1163M	0.001	Damaging	2.85
rs776723837	D1162H	0.026	Damaging	2.85
rs151305495	Q1157H	0.015	Damaging	2.85
rs754742754	V1144L	0.016	Damaging	2.84
rs767421908	R1140Q	0.021	Damaging	2.84
rs375976847	P1127L	0.05	Damaging	2.86
rs1284939504	G1116R	0.038	Damaging	2.88
rs150331990	H1102R	0.033	Damaging	2.86
rs755865204	P1099S	0.019	Damaging	2.86
rs1316623288	R1082C	0.018	Damaging	2.84
rs950686369	G1078C	0.006	Damaging	2.87
rs560179619	V1062M	0.001	Damaging	2.84
rs140852137	R1059C	0	Damaging	2.84
rs1478047497	V1051G	0	Damaging	2.84
rs1160158444	Y1043C	0.01	Damaging	2.85
rs767308840	N1040S	0.009	Damaging	2.85
rs1350013741	A1037G	0.021	Damaging	2.85
rs144533539	R1017W	0.001	Damaging	2.85
rs1282355438	T1012I	0.02	Damaging	2.89
rs373940840	N1026S	0.004	Damaging	2.85
rs1248096623	K1020T	0.004	Damaging	2.86
rs376854079	I996M	0.024	Damaging	2.85
rs1167927296	I996T	0.001	Damaging	2.85
rs876661227	R995W	0.009	Damaging	2.85
rs776149246	Y991C	0.001	Damaging	2.84
rs775590756	P970A	0.006	Damaging	2.99
rs1056948381	Y969H	0.018	Damaging	2.98
rs753992945	D965A	0.002	Damaging	2.89
rs757550838	V964M	0.05	Damaging	2.9
rs754990148	T947M	0.049	Damaging	2.85
rs978444139	F946L	0.05	Damaging	2.84
rs780533354	F946I	0.021	Damaging	2.84
rs977832207	G930V	0.001	Damaging	2.87
rs777416084	G930S	0.017	Damaging	2.87
rs745753378	R898C	0.008	Damaging	2.89
rs1460029697	S895R	0.005	Damaging	2.84
rs768990499	F892C	0.005	Damaging	2.92
rs1379893711	P880S	0.049	Damaging	2.86
rs1303994790	A874V	0.002	Damaging	2.85
rs1314966532	F864L	0.045	Damaging	2.85
rs868584117	S840C	0.021	Damaging	2.85
rs1228333897	P811L	0.001	Damaging	2.85
rs963570572	P811S	0.042	Damaging	2.85
rs140993011	T808M	0.047	Damaging	2.85
rs183555527	G806R	0.005	Damaging	2.85
rs1244845928	D785H	0.004	Damaging	2.86
rs1181332562	Y775C	0.05	Damaging	2.86
rs1313976937	S786C	0.042	Damaging	2.84

rs778066229	V759M	0.006	Damaging	2.85
rs1251285527	I752T	0.006	Damaging	2.85
rs764389789	D741G	0.048	Damaging	2.88
rs1381758934	G735R	0.01	Damaging	2.87
rs548322208	K722Q	0.041	Damaging	2.86
rs752326785	D706G	0.003	Damaging	2.95
rs368319266	D702N	0.011	Damaging	2.96
rs1336324296	N693S	0.008	Damaging	2.94
rs766157757	R689W	0	Damaging	2.96
rs1029809799	S682C	0.002	Damaging	2.95
rs767317190	F676Y	0.004	Damaging	3.01
rs760173233	G654S	0.006	Damaging	2.97
rs763570334	R651W	0.002	Damaging	2.97
rs753503985	R650H	0.002	Damaging	2.97
rs761410935	R650C	0	Damaging	2.97
rs777870040	A597D	0.028	Damaging	2.89
rs397509391	V590F	0.001	Damaging	2.88
rs397509393	G589E	0.003	Damaging	2.88
rs775941265	M581T	0.001	Damaging	2.85
rs760952579	P579S	0.044	Damaging	2.85
rs762421501	T578I	0.009	Damaging	2.85
rs959058396	D569H	0.005	Damaging	2.88
rs1003447106	A554T	0.002	Damaging	2.85
rs768869946	D548N	0.004	Damaging	2.86
rs763014417	T534M	0.015	Damaging	2.85
rs556213169	T533I	0.025	Damaging	2.85
rs865891586	E532K	0.035	Damaging	2.85
rs760542365	F515V	0.008	Damaging	2.85
rs1405357614	P497L	0.001	Damaging	2.85
rs199473690	Y495C	0	Damaging	2.85
rs199473692	Y495H	0	Damaging	2.85
rs1190635928	E473K	0.007	Damaging	2.86
rs1403539644	D444H	0.002	Damaging	2.86
rs1181209213	D423N	0.002	Damaging	2.86
rs777569395	S411N	0.044	Damaging	2.86
rs867003508	E400K	0.006	Damaging	2.92
rs966116269	K385N	0.013	Damaging	2.88
rs1294787183	Q358H	0.001	Damaging	3.06
rs1333537251	P350L	0.033	Damaging	3.13
rs758106190	K341Q	0.018	Damaging	3.16
rs747668607	R339C	0.032	Damaging	3.12
rs781477930	E317A	0.028	Damaging	3.17
rs1440048632	D316G	0.048	Damaging	3.17
rs996476593	D313Y	0.007	Damaging	3.17
rs143287044	Q310P	0.028	Damaging	3.17
rs1478185698	E317V	0.008	Damaging	3.17
rs1434425247	R298G	0.039	Damaging	3.59
rs775360871	R297Q	0.008	Damaging	3.59
rs1372902099	R297W	0.001	Damaging	3.59
rs1301997168	R287G	0.049	Damaging	3.54

rs1419316960	R264G	0.002	Damaging	3.67
rs1430311136	P262L	0.044	Damaging	3.77
rs1393687942	E261V	0.005	Damaging	3.77
rs1391845033	E251V	0.004	Damaging	3.77
rs1419372864	R246Q	0.007	Damaging	4.14
rs746582737	E242G	0.022	Damaging	4.14
rs1163672521	E241G	0.015	Damaging	4.14
rs768339415	E251D	0.034	Damaging	3.77
rs1353668988	E234K	0.009	Damaging	3.77
rs771381056	R246H	0.003	Damaging	4.14
rs201749864	R246C	0	Damaging	4.14
rs143598088	T229M	0.002	Damaging	3.77
rs150999369	G228E	0.005	Damaging	3.77
rs1319952912	E221V	0.009	Damaging	3.78
rs747469397	R205H	0.006	Damaging	4.05
rs1194542262	R205C	0.001	Damaging	4.05
rs1244292821	R204K	0.017	Damaging	4.05
rs1474694064	E202G	0.01	Damaging	4.09
rs756886439	K198N	0.03	Damaging	4.05
rs1476219241	P180R	0.005	Damaging	3.98
rs900075322	R178W	0	Damaging	3.98
rs201319352	T158P	0.011	Damaging	3.98
rs886054129	P151R	0.049	Damaging	3.98
rs1140470	A147G	0.017	Damaging	3.98
rs1414937646	S141R	0.002	Damaging	3.98
rs377146700	T137M	0.048	Damaging	3.98
rs775139340	R136H	0.033	Damaging	3.98
rs775139341	R136L	0.036	Damaging	3.98
rs138841970	R136C	0.028	Damaging	3.98
rs1361938846	P135H	0.031	Damaging	3.98
rs370207020	P129H	0.036	Damaging	3.98
rs370207021	P129R	0.025	Damaging	3.98
rs1085307725	A123G	0.033	Damaging	3.98
rs777666240	R119T	0.045	Damaging	3.98
rs770318428	N111K	0.005	Damaging	3.98
rs376894659	G110R	0.001	Damaging	3.98
rs1157913728	N109D	0	Damaging	3.98
rs1170262960	N104D	0	Damaging	3.98
rs1039555172	E102Q	0.047	Damaging	3.98
rs752966527	N100K	0.047	Damaging	3.97
rs1268674999	S91F	0.008	Damaging	3.98
rs779818604	L90V	0	Damaging	3.98
rs1456240920	K88E	0.004	Damaging	3.98
rs953187775	N87S	0.039	Damaging	3.98
rs1477841278	E71G	0.009	Damaging	3.98
rs1279416770	K70T	0.048	Damaging	3.98
rs61750054	R69L	0.037	Damaging	3.98
rs753673660	R69C	0.004	Damaging	3.98
rs1351910032	N59K	0.016	Damaging	3.98
rs755995375	E51K	0.03	Damaging	3.98

rs1234060339	L46F	0	Damaging	3.98
rs757938210	K45R	0	Damaging	3.98
rs1242559756	E44K	0.04	Damaging	3.98
rs779730617	C41W	0.003	Damaging	3.98
rs1379799242	E38K	0.005	Damaging	3.97
rs374817622	R33G	0.004	Damaging	3.97
rs753721329	R27Q	0.007	Damaging	3.97
rs780503694	R26S	0.005	Damaging	3.97
rs1206120198	R25C	0	Damaging	3.97
rs1271779036	V24I	0.012	Damaging	3.97
rs1257821053	D23N	0.043	Damaging	3.97
rs1224343919	D22N	0.009	Damaging	3.99
rs747559452	S19L	0.004	Damaging	3.99
rs768745074	A17V	0.005	Damaging	4.03
rs1476955893	P16Q	0.042	Damaging	4.14
rs1324769959	P16S	0.042	Damaging	4.14
rs1348019056	L13Q	0.003	Damaging	4.14
rs762812098	L13M	0.019	Damaging	4.14
rs1216341078	P11S	0.024	Damaging	4.14
rs1269997956	V10M	0.004	Damaging	4.14
rs994411260	A8G	0	Damaging	4.14
rs1217509888	A8S	0	Damaging	4.14
rs766984573	P7S	0.013	Damaging	4.14
rs760288360	A3V	0.01	Damaging	3.99
rs1248298976	P2R	0	Damaging	3.99
rs763647098	P2A	0	Damaging	3.99
rs753525461	M1V	0	Damaging	3.99

Supplementary Table 1C: List of Non-syn-SNVsfor which "Disease" results were predicted using SNPs&GO

rs numbers	aa substitution	SNPs&GO Prediction	Reliability index
rs930531589	His1573Gln	Disease	1
rs1388362405	Ser1556Phe	Disease	0
rs1461695373	.Arg1555Cys	Disease	4
rs1388371493	.Arg1551Trp	Disease	2
rs1316848811	Glu1518Lys	Disease	1
rs770571074	Gly1449Arg	Disease	1
rs774010132	Pro1432Ser	Disease	3
rs1442538027	Pro1429Leu	Disease	1
rs1382078791	Pro1375Arg	Disease	4
rs748486662	Arg1368Gln	Disease	0
rs867399027	Pro1328Leu	Disease	1
rs1182970957	.Cys1294Gly	Disease	2
rs535946725	Phe1262Leu	Disease	2
rs1052868434	Arg1261Trp	Disease	2
rs759915904	G1231R	Disease	4
rs779029407	Arg1207Cys	Disease	2
rs142648642	Thr1204Ile	Disease	2
rs1412363660	Ala1166Thr	Disease	2

rs745780816	Glu998Asp	Disease	1
rs758453625	.Lys997Glu	Disease	1
rs1167927296	Ile996Thr	Disease	4
rs876661227	Arg995Trp	Disease	3
rs776149246	Tyr991Cys	Disease	4
rs775590756	Pro970Ala	Disease	0
rs1056948381	Tyr969His	Disease	3
rs753992945	Asp965Ala	Disease	2
rs978444139	Phe946Leu	Disease	1
rs780533354	Phe946Leu	Disease	2
rs755712065	Val939Leu	Disease	4
rs1181438211	.Asp937Asn	Disease	7
rs1378414629	Ile931Met	Disease	2
rs1478243877	Ile931Asn	Disease	5
rs1174048074	Ile931Leu	Disease	5
rs977832207	Gly930Val	Disease	9
rs777416084	Gly930Ser	Disease	8
rs1302114385	Asn929Asp	Disease	6
rs1349231023	Thr927Ala	Disease	5
rs747445212	Ser925Ala	Disease	1
rs1389160994	Tyr923Ser	Disease	4
rs762002107	Leu922Pro	Disease	5
rs550380640	Arg920Gly	Disease	2
rs767511749	Glu915Lys	Disease	4
rs777323413	Leu911Pro	Disease	2
rs973184667	Arg909Thr	Disease	0
rs1360494258	Glu906Lys	Disease	3
rs753467933	Glu901Lys	Disease	0
rs756944531	Arg898His	Disease	4
rs745753378	Arg898Cys	Disease	6
rs1460029697	Ser895Arg	Disease	5
rs768990499	Phe892Cys	Disease	4
rs1303994790	Ala874Val	Disease	2
rs569867388	Tyr873Cys	Disease	3
rs367681882	Asp870His	Disease	1
rs750081202	Gln866Arg	Disease	7
rs1305123223	Gln866Lys	Disease	1
rs1314966532	Phe864Leu	Disease	4
rs758032585	Lys861Asn	Disease	1
rs753140952	Gly860Glu	Disease	5
rs62621087	Gly860Arg	Disease	2
rs571299133	Asp858Gly	Disease	3
rs779777304	Glu856Gly	Disease	1
rs768517249	Leu854Pro	Disease	3
rs761717775	Glu852Lys	Disease	1
rs751754286	Glu841Lys	Disease	1
rs868584117	Ser840Cys	Disease	2
rs183555527	Gly806Arg	Disease	7
rs1402220036	His795Tyr	Disease	1
rs1379915323	Ser786Arg	Disease	1

rs1468958840	Ser786Arg	Disease	1
rs1244845928	Asp785His	Disease	1
rs1181332562	Tyr775Cys	Disease	2
rs113497353	Leu774Pro	Disease	0
rs865989923	S786F	Disease	1
rs1330899035	Cys762Gly	Disease	1
rs370056750	Asp761Glu	Disease	1
rs1407084701	Glu758Lys	Disease	1
rs573248196	Ala754Val	Disease	1
rs1251285527	Ile752Thr	Disease	3
rs1402672876	Cys751Tyr	Disease	1
rs760149381	Cys751Gly	Disease	1
rs1381758934	Gly735Arg	Disease	6
rs1336324296	Asn693Ser	Disease	2
rs766157757	Arg689Trp	Disease	4
rs767317190	Phe676Tyr	Disease	4
rs760173233	Gly654Ser	Disease	7
rs1359313210	Arg651Gln	Disease	0
rs763570334	Arg651Trp	Disease	5
rs753503985	Arg650His	Disease	5
rs761410935	Arg650Cys	Disease	6
rs1181179755	Tyr625Cys	Disease	1
rs777870040	Ala597Asp	Disease	0
rs397509391	Val590Phe	Disease	3
rs397509393	Gly589Glu	Disease	7
rs775941265	Met581Thr	Disease	5
rs1420508762	Met581Leu	Disease	3
rs760952579	Pro579Ser	Disease	3
rs762421501	Thr578Ile	Disease	3
rs959058396	Asp569His	Disease	1
rs1003447106	Ala554Thr	Disease	2
rs748821501	Arg552Gln	Disease	3
rs768869946	Asp548Asn	Disease	4
rs375474222	Arg544Cys	Disease	1
rs1315979941	Gly539Val	Disease	2
rs556213169	Thr533Ile	Disease	4
rs865891586	Glu532Lys	Disease	4
rs1269876059	Ile531Phe	Disease	2
rs760542365	F515V	Disease	4
rs1405357614	Pro497Leu	Disease	4
rs1064796687	Ala496Glu	Disease	2
rs199473690	Tyr495Cys	Disease	6
rs199473692	Tyr495His	Disease	6
rs561762200	Glu494Asp	Disease	3
rs750390469	Ile477Leu	Disease	4
rs1487562713	Ala475Ser	Disease	3
rs1190635928	Glu473Lys	Disease	6
rs1403539644	Asp444His	Disease	1
rs1345721072	L449R	Disease	4
rs751093624	Glu432Lys	Disease	5

rs1181209213	Asp423Asn	Disease	6
rs758850158	Gly417Ser	Disease	7
rs181300723	His416Tyr	Disease	2
rs777569395	.Ser411Asn	Disease	3
rs867003508	.Glu400Lys	Disease	3
rs141562679	Lys366Ile	Disease	2
rs774875858	Asp362Asn	Disease	3
rs1294787183	Gln358His	Disease	4
rs1333537251	Pro350Leu	Disease	3
rs1372902099	Arg297Trp	Disease	1
rs1194542262	Arg205Cys	Disease	0
rs1375806732	Gly174Ser	Disease	1
rs777666240	R119T	Disease	0
rs146516082	R119G	Disease	1
rs745455817	R118C	Disease	2
rs376894659	G110R	Disease	0
rs1170262960	N104D	Disease	3
rs369196079	R101W	Disease	4
rs752966527	N100K	Disease	2
rs16999593	H97R	Disease	2
rs753670606	H97Y	Disease	2
rs1338494594	A96P	Disease	2
rs778222019	N94D	Disease	6
rs1268674999	S91F	Disease	3
rs779818604	L90V	Disease	4
rs1456240920	K88E	Disease	4
rs953187775	N87S	Disease	2
rs984728792	G77V	Disease	0
rs746687493	G77S	Disease	3
rs746687494	G77R	Disease	2
rs1206145138	E75K	Disease	3
rs1477841278	E71G	Disease	3
rs61750054	R69L	Disease	1
rs753673660	R69C	Disease	0
rs1351910032	N59K	Disease	1
rs1401965330	E51D	Disease	0
rs755995375	E51K	Disease	3
rs1234060339	L46F	Disease	3
rs757938210	K45R	Disease	0
rs1242559756	E44K	Disease	2
rs779730617	C41W	Disease	6
rs1218241317	C41R	Disease	6
rs1379799242	E38K	Disease	4
rs374817622	R33G	Disease	2
rs753721329	R27Q	Disease	1
rs780503694	R26S	Disease	2
rs1206120198	R25C	Disease	2
rs1224343919	D22N	Disease	1
rs747559452	S19L	Disease	2
rs768745074	A17V	Disease	0

rs1476955893	P16Q	Disease	2
rs1324769959	P16S	Disease	1
rs1348019056	L13Q	Disease	1

Supplementary Table 1D: List of Non-syn-SNVsfor which "Damaging" results were predicted using PolyPhen2

dbSNP ID	aa substitution	HumDiv	Score (Pph2_prob)	HumVar	Score (Pph2_prob)
rs1203183469	G1589C	probably damaging	1	probably damaging	1
rs750979984	A1587T	probably damaging	0.986	probably damaging	0.913
rs1248265917	P1582L	probably damaging	1	probably damaging	1
rs930531589	H1573Q	probably damaging	1	probably damaging	1
rs1301210022	K1572N	probably damaging	0.992	probably damaging	0.956
rs750769453	I1569T	probably damaging	0.996	probably damaging	0.959
rs747467776	R1564W	probably damaging	1	probably damaging	0.997
rs1170573036	G1558S	probably damaging	1	probably damaging	1
rs1388362405	S1556F	probably damaging	1	probably damaging	1
rs1461695373	R1555C	probably damaging	1	probably damaging	1
rs1393111051	R1551Q	probably damaging	1	probably damaging	1
rs1388371493	R1551W	probably damaging	1	probably damaging	1
rs1299212888	V1548M	probably damaging	1	probably damaging	1
rs1305204736	V1547M	probably damaging	1	probably damaging	1
rs919486255	V1539M	probably damaging	1	probably damaging	1
rs1316990553	N1529I	probably damaging	1	probably damaging	0.997
rs1292341520	V1527I	probably damaging	0.973	possibly damaging	0.683
rs1441106688	G1537S	probably damaging	1	probably damaging	1
rs1316848811	E1518K	probably damaging	0.999	probably damaging	0.981
rs1236455214	D1486H	probably damaging	0.995	probably damaging	0.975
rs746932608	G1482V	probably damaging	1	probably damaging	0.999
rs768764350	G1482S	probably damaging	1	probably damaging	0.996
rs768672451	E1480Q	possibly damaging	0.924	possibly damaging	0.756
rs781301028	V1479M	probably damaging	0.991	possibly damaging	0.806
rs769347864	S1477F	probably damaging	0.999	probably damaging	0.986
rs745789285	R1473H	probably damaging	1	probably damaging	0.999
rs772660711	L1472R	possibly damaging	0.571	possibly damaging	0.507
rs536916632	A1471T	probably damaging	0.963	possibly damaging	0.772
rs1363710824	S1469C	possibly damaging	0.901	possibly damaging	0.676
rs1167554550	S1467I	probably damaging	0.993	probably damaging	0.969
rs1368186167	G1465S	probably damaging	0.994	possibly damaging	0.7
rs764531629	N1464S	possibly damaging	0.877	possibly damaging	0.627
rs1274835966	H1459Q	possibly damaging	0.921	possibly damaging	0.892
rs1265513327	H1459R	possibly damaging	0.921	possibly damaging	0.857
rs1361579493	T1458I	possibly damaging	0.573	possibly damaging	0.493
rs754833277	G1465S	probably damaging	0.994	possibly damaging	0.7
rs1387794444	S1447L	possibly damaging	0.928	possibly damaging	0.772
rs769675693	R1445W	probably damaging	0.999	probably damaging	0.932
rs1341093379	V1444A	probably damaging	0.997	probably damaging	0.973

rs1488470434	R1453C	probably damaging	0.99	possibly damaging	0.846
rs770571074	G1449R	probably damaging	1	probably damaging	1
rs1442538027	P1429L	probably damaging	1	probably damaging	1
rs1187348790	R1426Q	probably damaging	0.983	possibly damaging	0.605
rs1183358759	R1424H	probably damaging	1	probably damaging	0.997
rs1365871222	R1424C	probably damaging	0.987	possibly damaging	0.751
rs555878888	A1423D	probably damaging	0.998	probably damaging	0.984
rs1158543755	A1422T	possibly damaging	0.929	possibly damaging	0.854
rs1031456207	M1417V	possibly damaging	0.949	possibly damaging	0.877
rs1375081003	D1416N	possibly damaging	0.898	possibly damaging	0.775
rs1386402643	C1414S	probably damaging	0.977	probably damaging	0.971
rs1252893841	I1413V	possibly damaging	0.763	possibly damaging	0.498
rs1450243310	D1411N	probably damaging	0.992	probably damaging	0.99
rs1442803580	R1401Q	probably damaging	0.999	probably damaging	0.989
rs1323856557	R1401W	probably damaging	1	probably damaging	0.999
rs766558026	F1396L	possibly damaging	0.73	possibly damaging	0.491
rs1324326054	F1396S	probably damaging	0.958	possibly damaging	0.904
rs1226189469	W1395C	probably damaging	0.96	possibly damaging	0.84
rs886054127	I1386F	probably damaging	0.997	probably damaging	0.978
rs375225009	S1382L	probably damaging	0.961	possibly damaging	0.643
rs1382078791	P1375R	probably damaging	1	probably damaging	1
rs1356963122	D1373N	probably damaging	1	probably damaging	0.996
rs1292991293	S1372F	possibly damaging	0.843	possibly damaging	0.737
rs748486662	R1368Q	probably damaging	0.98	possibly damaging	0.832
rs1259943763	S1359L	probably damaging	1	probably damaging	0.996
rs771611874	S1359T	probably damaging	0.999	probably damaging	0.994
rs1265788604	N1353S	possibly damaging	0.872	possibly damaging	0.759
rs1164503433	N1353H	probably damaging	1	probably damaging	0.996
rs765098980	V1343M	probably damaging	1	probably damaging	0.998
rs141791913	A1338V	probably damaging	0.996	probably damaging	0.977
rs765793648	A1335D	possibly damaging	0.79	possibly damaging	0.574
rs867399027	P1328L	probably damaging	1	probably damaging	1
rs1328566140	E1322K	possibly damaging	0.779	possibly damaging	0.794
rs757703243	G1321E	probably damaging	1	probably damaging	0.993
rs766051225	A1318V	probably damaging	0.999	probably damaging	0.973
rs779967013	A1318T	probably damaging	0.999	probably damaging	0.962
rs1044312254	I1315M	probably damaging	0.98	probably damaging	0.972
rs1197552228	G1321S	probably damaging	1	probably damaging	0.997
rs767041788	G1297S	probably damaging	1	probably damaging	0.995
rs1442277728	C1294S	probably damaging	0.998	probably damaging	0.99
rs1182970957	C1294G	probably damaging	0.998	probably damaging	0.993
rs564106859	R1289H	probably damaging	1	probably damaging	0.977
rs200312526	R1289C	probably damaging	1	probably damaging	0.977
rs1010743136	R1285H	probably damaging	1	probably damaging	0.967
rs748262646	R1276H	probably damaging	0.996	probably damaging	0.938
rs1235846614	V1268I	possibly damaging	0.848	possibly damaging	0.651
rs1343782138	E1266D	probably damaging	0.999	probably damaging	0.976
rs1178617228	L1280I	probably damaging	0.999	probably damaging	0.974
rs535946725	F1262L	possibly damaging	0.593	possibly damaging	0.626
rs774073234	R1261Q	probably damaging	0.985	possibly damaging	0.842

rs1052868434	R1261W	probably damaging	1	probably damaging	0.989
rs1296808828	R1259W	probably damaging	1	probably damaging	0.999
rs1168306248	D1256N	probably damaging	1	probably damaging	0.998
rs1488049319	V1249A	possibly damaging	0.756	possibly damaging	0.456
rs1408962462	V1248M	probably damaging	1	probably damaging	0.993
rs1085307800	Y1240C	probably damaging	1	probably damaging	1
rs780156714	R1238H	probably damaging	1	probably damaging	0.997
rs746456488	R1238C	probably damaging	1	probably damaging	0.997
rs1290055474	S1253L	probably damaging	1	probably damaging	1
rs768280180	N1236S	probably damaging	1	probably damaging	0.998
rs1312709316	G1222S	probably damaging	1	probably damaging	1
rs1353293786	D1216N	possibly damaging	0.85	possibly damaging	0.599
rs759915904	G1231R	probably damaging	1	probably damaging	1
rs767953016	P1212A	probably damaging	1	probably damaging	1
rs1269778997	R1210W	probably damaging	0.994	probably damaging	0.948
rs1255396898	G1208D	probably damaging	0.992	probably damaging	0.973
rs764137405	G1208S	probably damaging	0.997	probably damaging	0.962
rs757460628	R1207H	probably damaging	0.981	possibly damaging	0.735
rs746926863	L1197M	possibly damaging	0.885	possibly damaging	0.771
rs1367702161	T1185I	possibly damaging	0.877	possibly damaging	0.627
rs1255153711	R1178W	probably damaging	1	probably damaging	0.999
rs1434985283	A1176V	probably damaging	1	probably damaging	1
rs772419584	A1173V	probably damaging	1	probably damaging	0.996
rs1193652618	P1172R	probably damaging	0.985	possibly damaging	0.889
rs1412363660	A1166T	probably damaging	1	probably damaging	1
rs764541314	T1163M	probably damaging	1	probably damaging	0.982
rs776723837	D1162H	possibly damaging	0.708	possibly damaging	0.528
rs151305495	Q1157H	probably damaging	1	probably damaging	0.997
rs754742754	V1144L	probably damaging	0.995	probably damaging	0.951
rs767421908	R1140Q	probably damaging	1	probably damaging	0.984
rs1284939504	G1116R	probably damaging	0.972	possibly damaging	0.769
rs759653796	P1099L	probably damaging	0.984	possibly damaging	0.866
rs755865204	P1099S	probably damaging	1	probably damaging	0.999
rs1479104782	F1096L	probably damaging	0.997	probably damaging	0.967
rs747265409	G1079S	possibly damaging	0.935	possibly damaging	0.668
rs950686369	G1078C	probably damaging	0.999	probably damaging	0.971
rs560179619	V1062M	probably damaging	0.998	probably damaging	0.967
rs140852137	R1059C	probably damaging	0.999	probably damaging	0.93
rs1478047497	V1051G	probably damaging	0.994	probably damaging	0.974
rs1160158444	Y1043C	probably damaging	1	probably damaging	0.994
rs767308840	N1040S	possibly damaging	0.828	possibly damaging	0.654
rs144533539	R1017W	probably damaging	0.999	probably damaging	0.952
rs373940840	N1026S	possibly damaging	0.932	possibly damaging	0.731
rs1256073808	G1007S	probably damaging	0.991	possibly damaging	0.874
rs1248096623	K1020T	probably damaging	0.999	probably damaging	0.988
rs1199169323	F1000S	possibly damaging	0.866	possibly damaging	0.593
rs745780816	E998D	possibly damaging	0.929	possibly damaging	0.665
rs376854079	I996M	probably damaging	1	probably damaging	0.997
rs1167927296	I996T	probably damaging	1	probably damaging	0.992
rs876661227	R995W	probably damaging	1	probably damaging	0.985

rs776149246	Y991C	probably damaging	0.999	probably damaging	0.971
rs747897637	E989K	possibly damaging	0.787	possibly damaging	0.7
rs775590756	P970A	probably damaging	1	probably damaging	0.991
rs753992945	D965A	probably damaging	0.998	probably damaging	0.981
rs1489509959	D965N	possibly damaging	0.75	possibly damaging	0.558
rs757550838	V964M	probably damaging	1	probably damaging	0.994
rs1328974927	P955T	possibly damaging	0.872	possibly damaging	0.474
rs978444139	F946L	probably damaging	0.995	probably damaging	0.93
rs780533354	F946I	probably damaging	0.988	possibly damaging	0.838
rs755712065	V939L	probably damaging	0.997	probably damaging	0.98
rs977832207	G930V	possibly damaging	0.923	possibly damaging	0.739
rs1389160994	Y923S	probably damaging	0.957	possibly damaging	0.641
rs550380640	R920G	possibly damaging	0.771	possibly damaging	0.483
rs755625901	E912K	possibly damaging	0.895	possibly damaging	0.496
rs745753378	R898C	probably damaging	0.999	possibly damaging	0.88
rs1460029697	S895R	probably damaging	0.997	probably damaging	0.947
rs768990499	F892C	probably damaging	1	probably damaging	0.993
rs1379893711	P880S	probably damaging	0.967	possibly damaging	0.838
rs771879227	P879L	possibly damaging	0.655	possibly damaging	0.557
rs775305891	P879S	probably damaging	0.999	probably damaging	0.988
rs1160065459	S878F	probably damaging	0.966	possibly damaging	0.796
rs1303994790	A874V	probably damaging	0.999	probably damaging	0.946
rs750081202	Q866R	possibly damaging	0.536	possibly damaging	0.549
rs1314966532	F864L	probably damaging	0.997	probably damaging	0.977
rs753140952	G860E	probably damaging	0.998	probably damaging	0.968
rs62621087	G860R	probably damaging	1	probably damaging	0.99
rs761717775	E852K	probably damaging	0.99	possibly damaging	0.888
rs868584117	S840C	probably damaging	0.999	probably damaging	0.99
rs1228333897	P811L	probably damaging	1	probably damaging	0.998
rs963570572	P811S	probably damaging	0.998	probably damaging	0.951
rs140993011	T808M	probably damaging	0.979	possibly damaging	0.727
rs183555527	G806R	probably damaging	0.987	possibly damaging	0.751
rs373366822	A799T	probably damaging	0.989	possibly damaging	0.837
rs1402220036	H795Y	possibly damaging	0.827	possibly damaging	0.588
rs1244845928	D785H	probably damaging	0.993	probably damaging	0.93
rs757300501	A781G	probably damaging	0.962	possibly damaging	0.688
rs1223065154	A777S	probably damaging	0.987	probably damaging	0.949
rs1181332562	Y775C	probably damaging	0.999	probably damaging	0.981
rs113497353	L774P	possibly damaging	0.557	possibly damaging	0.617
rs1201583942	P773L	probably damaging	0.995	possibly damaging	0.858
rs1219579994	V765L	possibly damaging	0.701	possibly damaging	0.534
rs1330899035	C762G	probably damaging	0.998	probably damaging	0.968
rs370056750	D761E	possibly damaging	0.95	possibly damaging	0.823
rs778066229	V759M	probably damaging	0.969	possibly damaging	0.889
rs142562681	T756N	possibly damaging	0.757	possibly damaging	0.646
rs1381758934	G735R	probably damaging	1	probably damaging	0.99
rs548322208	K722Q	possibly damaging	0.848	possibly damaging	0.564
rs752326785	D706G	probably damaging	0.991	possibly damaging	0.83
rs1336324296	N693S	probably damaging	1	probably damaging	0.991
rs766157757	R689W	probably damaging	1	probably damaging	1

rs1029809799	S682C	probably damaging	1	probably damaging	0.986
rs767317190	F676Y	probably damaging	1	probably damaging	1
rs1486893166	V655I	probably damaging	0.995	possibly damaging	0.784
rs760173233	G654S	probably damaging	1	probably damaging	0.997
rs763570334	R651W	probably damaging	0.999	possibly damaging	0.893
rs753503985	R650H	probably damaging	1	probably damaging	0.986
rs761410935	R650C	probably damaging	1	probably damaging	0.995
rs756254318	E642Q	probably damaging	0.992	possibly damaging	0.864
rs397509391	V590F	probably damaging	0.999	probably damaging	0.98
rs397509393	G589E	probably damaging	1	probably damaging	1
rs775941265	M581T	probably damaging	0.996	probably damaging	0.933
rs1420508762	M581L	possibly damaging	0.768	possibly damaging	0.474
rs760952579	P579S	probably damaging	1	probably damaging	0.978
rs959058396	D569H	probably damaging	0.999	probably damaging	0.96
rs397509392	A554G	probably damaging	1	probably damaging	1
rs1003447106	A554T	probably damaging	1	probably damaging	1
rs748821501	R552Q	probably damaging	0.987	possibly damaging	0.701
rs768869946	D548N	probably damaging	0.999	probably damaging	0.971
rs763014417	T534M	probably damaging	0.999	probably damaging	0.947
rs556213169	T533I	probably damaging	0.996	probably damaging	0.968
rs865891586	E532K	probably damaging	0.975	possibly damaging	0.655
rs760542365	F515V	probably damaging	0.998	probably damaging	0.968
rs1405357614	P497L	probably damaging	0.996	probably damaging	0.959
rs199473690	Y495C	probably damaging	1	probably damaging	0.998
rs199473692	Y495H	probably damaging	1	probably damaging	0.998
rs1487562713	A475S	probably damaging	0.973	probably damaging	0.923
rs1190635928	E473K	probably damaging	0.993	probably damaging	0.963
rs1308265840	P447S	possibly damaging	0.846	possibly damaging	0.557
rs1403539644	D444H	probably damaging	0.987	probably damaging	0.954
rs1345721072	L449R	possibly damaging	0.708	possibly damaging	0.555
rs1181209213	D423N	probably damaging	1	probably damaging	1
rs758850158	G417S	probably damaging	1	probably damaging	0.992
rs777569395	S411N	possibly damaging	0.943	possibly damaging	0.775
rs1294787183	Q358H	probably damaging	0.991	possibly damaging	0.886
rs1372902099	R297W	probably damaging	0.99	possibly damaging	0.53
rs368346471	Q289H	probably damaging	0.996	possibly damaging	0.804
rs1301997168	R287G	probably damaging	0.979	possibly damaging	0.628
rs201749864	R246C	probably damaging	0.991	possibly damaging	0.645
rs143598088	T229M	probably damaging	0.999	possibly damaging	0.828
rs747469397	R205H	probably damaging	0.998	possibly damaging	0.791
rs1194542262	R205C	probably damaging	0.999	possibly damaging	0.791
rs1244292821	R204K	probably damaging	0.983	possibly damaging	0.621
rs900075322	R178W	probably damaging	1	probably damaging	0.965
rs978575291	P155R	possibly damaging	0.933	possibly damaging	0.462
rs1361938846	P135H	possibly damaging	0.948	possibly damaging	0.594
rs773200699	S133F	possibly damaging	0.906	possibly damaging	0.459
rs370207020	P129H	probably damaging	0.958	possibly damaging	0.525
rs376894659	G110R	probably damaging	1	probably damaging	0.999
rs1157913728	N109D	probably damaging	0.997	probably damaging	0.98
rs1170262960	N104D	probably damaging	0.999	probably damaging	0.995

rs369196079	R101W	probably damaging	0.998	possibly damaging	0.786
rs1338494594	A96P	probably damaging	0.984	possibly damaging	0.825
rs778222019	N94D	probably damaging	0.999	probably damaging	0.995
rs1268674999	S91F	probably damaging	0.984	possibly damaging	0.876
rs779818604	L90V	probably damaging	0.999	probably damaging	0.994
rs1456240920	K88E	probably damaging	0.999	probably damaging	0.995
rs953187775	N87S	probably damaging	0.999	probably damaging	0.992
rs984728792	G77V	probably damaging	1	probably damaging	1
rs746687493	G77S	probably damaging	1	probably damaging	1
rs746687494	G77R	probably damaging	1	probably damaging	1
rs1206145138	E75K	probably damaging	1	probably damaging	0.995
rs1477841278	E71G	probably damaging	1	probably damaging	0.996
rs1279416770	K70T	probably damaging	1	probably damaging	0.998
rs753673660	R69C	probably damaging	0.99	possibly damaging	0.536
rs375585911	T55A	probably damaging	0.997	probably damaging	0.992
rs1401965330	E51D	probably damaging	0.997	probably damaging	0.992
rs755995375	E51K	probably damaging	1	probably damaging	0.995
rs1234060339	L46F	probably damaging	1	probably damaging	0.998
rs757938210	K45R	probably damaging	0.999	probably damaging	0.995
rs1242559756	E44K	probably damaging	0.999	probably damaging	0.995
rs779730617	C41W	probably damaging	1	probably damaging	0.998
rs1218241317	C41R	probably damaging	0.999	probably damaging	0.998
rs1379799242	E38K	probably damaging	0.999	probably damaging	0.995
rs374817622	R33G	probably damaging	0.997	probably damaging	0.994
rs753721329	R27Q	probably damaging	0.999	probably damaging	0.994
rs780503694	R26S	probably damaging	0.997	probably damaging	0.994
rs1340824034	R26K	probably damaging	0.992	probably damaging	0.987
rs1206120198	R25C	probably damaging	1	probably damaging	0.98
rs1271779036	V24I	probably damaging	0.992	possibly damaging	0.89
rs1224343919	D22N	probably damaging	1	probably damaging	0.978
rs747559452	S19L	probably damaging	0.996	possibly damaging	0.726
rs768745074	A17V	probably damaging	0.999	probably damaging	0.996
rs1476955893	P16Q	possibly damaging	0.81	possibly damaging	0.574
rs1348019056	L13Q	probably damaging	0.999	probably damaging	0.997
rs762812098	L13M	probably damaging	0.999	probably damaging	0.996
rs1269997956	V10M	probably damaging	0.999	probably damaging	0.994
rs759577079	R9Q	probably damaging	0.999	probably damaging	0.978
rs994411260	A8G	probably damaging	0.997	probably damaging	0.985
rs1217509888	A8S	probably damaging	0.997	probably damaging	0.985
rs766984573	P7S	probably damaging	1	probably damaging	0.996
rs1487433053	A6T	probably damaging	1	probably damaging	0.99
rs760288360	A3V	probably damaging	1	probably damaging	0.986
rs1248298976	P2R	probably damaging	1	probably damaging	0.999
rs763647098	P2A	probably damaging	0.999	probably damaging	0.994
rs753525461	M1V	possibly damaging	0.865	possibly damaging	0.824

Supplementary Table 1E: List of Non-syn-SNVsfor which “Damaging” results were predicted using Fathmm

dbSNP ID	aa substitution 1	Prediction	Score
rs764265673	L1590F	DAMAGING	-1.85
rs1248265917	P1582L	DAMAGING	-1.95
rs930531589	H1573Q	DAMAGING	-1.79
rs750769453	I1569T	DAMAGING	-1.73
rs758849260	I1569V	DAMAGING	-1.81
rs747467776	R1564W	DAMAGING	-3.28
rs1388362405	S1556F	DAMAGING	-1.74
rs1461695373	R1555C	DAMAGING	-2.62
rs1299212888	V1548M	DAMAGING	-1.73
rs1305204736	V1547M	DAMAGING	-1.85
rs1316990553	N1529I	DAMAGING	-1.88
rs1441106688	G1537S	DAMAGING	-1.83
rs770535972	R1466H	DAMAGING	-1.85
rs147984942	R1466C	DAMAGING	-1.88
rs1368186167	G1465S	DAMAGING	-1.89
rs764531629	N1464S	DAMAGING	-1.79
rs755902999	R1462S	DAMAGING	-1.67
rs142647321	H1460Q	DAMAGING	-1.73
rs765347113	H1460R	DAMAGING	-1.51
rs1274835966	H1459Q	DAMAGING	-1.81
rs1265513327	H1459R	DAMAGING	-1.64
rs1361579493	T1458I	DAMAGING	-1.91
rs750520436	T1458A	DAMAGING	-1.92
rs780170131	R1456Q	DAMAGING	-1.84
rs1189691665	M1451I	DAMAGING	-1.83
rs1415174324	T1450I	DAMAGING	-1.82
rs754833277	G1465S	DAMAGING	-1.89
rs1387794444	S1447L	DAMAGING	-1.86
rs1488470434	R1453C	DAMAGING	-1.93
rs770571074	G1449R	DAMAGING	-1.86
rs1450243310	D1411N	DAMAGING	-1.72
rs770284928	Q1404E	DAMAGING	-1.78
rs1241729369	G1402D	DAMAGING	-1.87
rs1442803580	R1401Q	DAMAGING	-1.77
rs1323856557	R1401W	DAMAGING	-1.83
rs766558026	F1396L	DAMAGING	-1.77
rs1324326054	F1396S	DAMAGING	-1.73
rs1226189469	W1395C	DAMAGING	-1.83
rs759807018	Q1393H	DAMAGING	-1.84
rs367897930	E1391D	DAMAGING	-1.88
rs886054127	I1386F	DAMAGING	-1.77
rs375225009	S1382L	DAMAGING	-1.82
rs757243017	R1378Q	DAMAGING	-1.85
rs1454184730	E1376G	DAMAGING	-1.99
rs1356963122	D1373N	DAMAGING	-2.19
rs748486662	R1368Q	DAMAGING	-1.82
rs201167482	I1354L	DAMAGING	-1.78
rs1265788604	N1353S	DAMAGING	-1.83
rs1164503433	N1353H	DAMAGING	-1.88

rs1369475967	K1348R	DAMAGING	-1.83
rs1233616787	V1344L	DAMAGING	-1.8
rs765098980	V1343M	DAMAGING	-1.95
rs780926114	K1323N	DAMAGING	-1.78
rs754203974	K1323R	DAMAGING	-1.8
rs1328566140	E1322K	DAMAGING	-1.84
rs766051225	A1318V	DAMAGING	-1.8
rs779967013	A1318T	DAMAGING	-1.76
rs1044312254	I1315M	DAMAGING	-2.31
rs767041788	G1297S	DAMAGING	-1.74
rs1442277728	C1294S	DAMAGING	-1.8
rs1182970957	C1294G	DAMAGING	-1.81
rs1010743136	R1285H	DAMAGING	-1.84
rs755492225	R1285C	DAMAGING	-1.87
rs748262646	R1276H	DAMAGING	-1.77
rs1343782138	E1266D	DAMAGING	-4.74
rs774073234	R1261Q	DAMAGING	-1.78
rs1052868434	R1261W	DAMAGING	-1.87
rs1168306248	D1256N	DAMAGING	-1.81
rs1488049319	V1249A	DAMAGING	-1.79
rs1408962462	V1248M	DAMAGING	-1.86
rs1085307800	Y1240C	DAMAGING	-1.82
rs780156714	R1238H	DAMAGING	-1.87
rs746456488	R1238C	DAMAGING	-1.93
rs1290055474	S1253L	DAMAGING	-1.85
rs1353293786	D1216N	DAMAGING	-1.99
rs759915904	G1231R	DAMAGING	-1.79
rs767953016	P1212A	DAMAGING	-2
rs753205659	R1210Q	DAMAGING	-1.75
rs1269778997	R1210W	DAMAGING	-1.87
rs746926863	L1197M	DAMAGING	-1.84
rs1367702161	T1185I	DAMAGING	-1.83
rs1255153711	R1178W	DAMAGING	-1.94
rs1434985283	A1176V	DAMAGING	-1.66
rs772419584	A1173V	DAMAGING	-2.4
rs1193652618	P1172R	DAMAGING	-1.78
rs764541314	T1163M	DAMAGING	-1.86
rs151305495	Q1157H	DAMAGING	-1.93
rs767421908	R1140Q	DAMAGING	-1.93
rs759653796	P1099L	DAMAGING	-1.76
rs755865204	P1099S	DAMAGING	-2.02
rs1479104782	F1096L	DAMAGING	-2.19
rs377078524	R1082H	DAMAGING	-2.06
rs1316623288	R1082C	DAMAGING	-2.08
rs747265409	G1079S	DAMAGING	-1.98
rs370075258	M1077K	DAMAGING	-1.78
rs768431034	M1077V	DAMAGING	-1.97
rs1194144103	V1074L	DAMAGING	-2.03
rs776461147	V1072I	DAMAGING	-2.03
rs1276680499	L1068V	DAMAGING	-2.06

rs560179619	V1062M	DAMAGING	-3.45
rs757791181	T1061A	DAMAGING	-2.01
rs779489678	R1059H	DAMAGING	-2.07
rs140852137	R1059C	DAMAGING	-2.12
rs1217143527	Q1073E	DAMAGING	-1.92
rs747215182	A1055P	DAMAGING	-2.27
rs1426702144	K1054R	DAMAGING	-2.1
rs776374384	F1053L	DAMAGING	-1.66
rs1478047497	V1051G	DAMAGING	-2.26
rs187394074	V1050M	DAMAGING	-2.07
rs762862182	A1049V	DAMAGING	-2.11
rs548883904	D1046N	DAMAGING	-2.02
rs1160158444	Y1043C	DAMAGING	-2.32
rs767308840	N1040S	DAMAGING	-2.17
rs758882865	A1049V	DAMAGING	-2.11
rs780153466	T1031I	DAMAGING	-2.06
rs751674187	T1031A	DAMAGING	-1.99
rs774833250	N1019K	DAMAGING	-2.01
rs199827346	R1017Q	DAMAGING	-2.08
rs144533539	R1017W	DAMAGING	-2.13
rs764244867	I1014V	DAMAGING	-2.01
rs1282355438	T1012I	DAMAGING	-2.07
rs373940840	N1026S	DAMAGING	-2.02
rs1221584713	S1005T	DAMAGING	-2.03
rs1248096623	K1020T	DAMAGING	-2.01
rs1487276325	P1002L	DAMAGING	-1.98
rs1196329826	P1002S	DAMAGING	-1.93
rs1199169323	F1000S	DAMAGING	-2.1
rs745780816	E998D	DAMAGING	-2.43
rs1330228268	K997R	DAMAGING	-2.05
rs758453625	K997E	DAMAGING	-1.64
rs376854079	I996M	DAMAGING	-2.82
rs1167927296	I996T	DAMAGING	-2.77
rs876661227	R995W	DAMAGING	-2.31
rs776149246	Y991C	DAMAGING	-2.37
rs747897637	E989K	DAMAGING	-2.08
rs755712065	V939L	DAMAGING	-2.91
rs1181438211	D937N	DAMAGING	-2.53
rs771879227	P879L	DAMAGING	-1.76
rs775305891	P879S	DAMAGING	-2.02
rs1160065459	S878F	DAMAGING	-2.12
rs1303994790	A874V	DAMAGING	-2.08
rs569867388	Y873C	DAMAGING	-2.03
rs1336107599	D870E	DAMAGING	-2.29
rs367681882	D870H	DAMAGING	-2.42
rs750081202	Q866R	DAMAGING	-1.93
rs1305123223	Q866K	DAMAGING	-1.88
rs1314966532	F864L	DAMAGING	-2.02
rs758032585	K861N	DAMAGING	-2
rs753140952	G860E	DAMAGING	-1.84

rs62621087	G860R	DAMAGING	-2
rs749258509	D859N	DAMAGING	-2.03
rs772216000	G857E	DAMAGING	-1.95
rs758465656	M845I	DAMAGING	-1.96
rs751754286	E841K	DAMAGING	-2.25
rs868584117	S840C	DAMAGING	-2.04
rs781003939	P839T	DAMAGING	-1.96
rs1177086967	A838V	DAMAGING	-2.13
rs1363883264	K837R	DAMAGING	-2.06
rs1422521201	I835V	DAMAGING	-1.86
rs747921118	S830G	DAMAGING	-1.56
rs1345541911	M823T	DAMAGING	-1.97
rs1228333897	P811L	DAMAGING	-2.07
rs963570572	P811S	DAMAGING	-1.98
rs140993011	T808M	DAMAGING	-2.08
rs183555527	G806R	DAMAGING	-2.1
rs373366822	A799T	DAMAGING	-1.87
rs1402220036	H795Y	DAMAGING	-2.01
rs988229673	A794G	DAMAGING	-1.97
rs1379915323	S786R	DAMAGING	-2.05
rs1468958840	S786R	DAMAGING	-2.05
rs1244845928	D785H	DAMAGING	-2.1
rs757300501	A781G	DAMAGING	-2.05
rs148987580	T780M	DAMAGING	-2.04
rs899735320	T780A	DAMAGING	-2
rs1223065154	A777S	DAMAGING	-2.18
rs1181332562	Y775C	DAMAGING	-2.08
rs113497353	L774P	DAMAGING	-2
rs1201583942	P773L	DAMAGING	-2.29
rs865989923	S786F	DAMAGING	-2.12
rs1313976937	S786C	DAMAGING	-2.13
rs1219579994	V765L	DAMAGING	-2.15
rs1330899035	C762G	DAMAGING	-2.06
rs370056750	D761E	DAMAGING	-2.47
rs778066229	V759M	DAMAGING	-2.57
rs1346601061	E758G	DAMAGING	-2.03
rs1407084701	E758K	DAMAGING	-1.99
rs142562681	T756N	DAMAGING	-2.32
rs199473690	Y495C	DAMAGING	-3.34
rs199473692	Y495H	DAMAGING	-3.32
rs746582737	E242G	DAMAGING	-1.52

Supplementary Table 1F: Results of analyses of non-syn-SNVs using iMutant 3.0 analyses (iMutant 3.0 results were not used to prioritize the *DNMT1* SNVs but to look into the changes brought about by respective substitutions upon DNMT1 protein stability; Note: Results could not be obtained for 34 non-syn-SNVs out of 731)

rs numbers	aa substitution 1	iMutant predictions

		SVM2 prediction (DDG<0: Decrease Stability; DDG>0: Increase Stability)		
		**DDG Value Prediction (Kcal/mol)**	**Protein Stability**	**RI**
rs760959433	I1608V	-0.8	Decrease	5
rs764265673	L1590F	-0.87	Decrease	6
rs1203183469	G1589C	-0.86	Decrease	5
rs750979984	A1587T	-0.58	Decrease	5
rs1248265917	P1582L	-0.43	Decrease	5
rs930531589	H1573Q	-0.57	Decrease	6
rs1301210022	K1572N	-0.55	Decrease	1
rs750769453	I1569T	-2.63	Decrease	9
rs758849260	I1569V	-1.15	Decrease	8
rs1479455681	N1568S	-0.7	Decrease	7
rs747467776	R1564W	-1.07	Decrease	8
rs1395078736	T1562S	-0.98	Decrease	8
rs1170573036	G1558S	-1.32	Decrease	8
rs1461695373	R1555C	-1.2	Decrease	5
rs1393111051	R1551Q	-1.46	Decrease	7
rs1388371493	R1551W	-0.35	Decrease	4
rs1299212888	V1548M	0.75	Decrease	6
rs1305204736	V1547M	-0.87	Decrease	7
rs919486255	V1539M	-0.92	Decrease	7
rs1292341520	V1529I	-0.28	Decrease	2
rs1441106688	G1537S	-0.94	Decrease	5
rs1316848811	E1518K	-0.86	Decrease	9
rs1194509546	A1489G	-0.73	Decrease	8
rs1418458019	A1488V	-0.25	Decrease	2
rs749413587	A1488T	-0.77	Decrease	8
rs1236455214	D1486H	-0.17	Decrease	1
rs746932608	G1482V	-0.64	Decrease	7
rs768764350	G1482S	-0.97	Decrease	8
rs761440391	A1481V	-0.21	Decrease	1
rs768672451	E1480Q	-1.07	Decrease	8
rs781301028	V1479M	-0.68	Decrease	7
rs745789285	R1473H	-1.28	Decrease	8
rs772660711	L1472R	-1.66	Decrease	9
rs866729887	L1472F	-1.25	Decrease	8
rs536916632	A1471T	-0.65	Decrease	5
rs1363710824	S1469C	-0.92	Decrease	7
rs1405528301	S1468N	-0.62	Decrease	3
rs770535972	R1466H	-1.45	Decrease	9
rs147984942	R1466C	-1.21	Decrease	4
rs1368186167	G1465S	-1.3	Decrease	8
rs764531629	N1464S	-0.6	Decrease	5
rs755902999	R1462S	-1.42	Decrease	8
rs142647321	H1460Q	-0.74	Decrease	4
rs765347113	H1460R	-0.45	Decrease	2
rs1274835966	H1459Q	-0.75	Decrease	5
rs1265513327	H1459R	-0.48	Decrease	4

rs1361579493	T1458I	-0.77	Decrease	8
rs750520436	T1458A	-1.55	Decrease	9
rs780170131	R1456Q	-1.06	Decrease	8
rs1189691665	M1451I	-0.74	Decrease	7
rs1415174324	T1450I	-0.72	Decrease	8
rs754833277	G1465S	-1.3	Decrease	8
rs1387794444	S1447L	-0.12	Decrease	1
rs748089711	R1445Q	-1.22	Decrease	9
rs769675693	R1445W	-0.53	Decrease	7
rs1341093379	V1444A	-1.73	Decrease	7
rs1488470434	R1453C	-1.09	Decrease	5
rs770571074	G1449R	-0.63	Decrease	6
rs774010132	P1432S	-1.59	Decrease	9
rs1283448599	A1431T	-0.65	Decrease	6
rs1442538027	P1429L	-0.47	Decrease	4
rs1255037718	H1427N	-0.6	Decrease	4
rs1187348790	R1426Q	-0.85	Decrease	7
rs139918621	R1426G	-0.34	Decrease	4
rs1183358759	R1424H	-1.18	Decrease	8
rs1365871222	R1424C	-0.79	Decrease	4
rs555878888	A1423D	-0.74	Decrease	6
rs1158543755	A1422T	-0.67	Decrease	6
rs1031456207	M1417V	-1.19	Decrease	8
rs1375081003	D1416N	-0.61	Decrease	2
rs1386402643	C1414S	-0.82	Decrease	6
rs1252893841	I1413V	-1.12	Decrease	9
rs1450243310	D1411N	-0.47	Decrease	2
rs374047326	A1419T	-0.72	Decrease	6
rs1241729369	G1402D	-0.5	Decrease	1
rs1442803580	R1401Q	-0.39	Decrease	4
rs766558026	F1396L	-0.53	Decrease	3
rs1324326054	F1396S	-1.36	Decrease	7
rs1226189469	W1395C	-1.19	Decrease	7
rs759807018	G1393H	-0.76	Decrease	6
rs367897930	E1391D	-0.26	Decrease	1
rs886054127	I1386F	-1.17	Decrease	9
rs757243017	R1378Q	-1.16	Decrease	9
rs1454184730	E1376G	-1.65	Decrease	9
rs1382078791	P1375R	-1.22	Decrease	8
rs1356963122	D1373N	-1.05	Decrease	5
rs748486662	R1368Q	-1.17	Decrease	8
rs1259943763	S1359L	-0.35	Decrease	1
rs771611874	S1359T	-0.84	Decrease	5
rs774538556	S1358I	-0.06	Decrease	2
rs776250179	R1356K	-1.27	Decrease	9
rs201167482	I1354L	-1.26	Decrease	7
rs1265788604	N1353S	-0.69	Decrease	7
rs1164503433	N1353H	-1.08	Decrease	9
rs1369475967	K1348R	-0.34	Decrease	1
rs1233616787	V1344L	-1.07	Decrease	7

rs765098980	V1343M	-1.05	Decrease	8
rs141791913	A1338V	-0.19	Decrease	1
rs1254786385	P1336L	-0.5	Decrease	5
rs765793648	A1335D	-0.43	Decrease	1
rs867399027	P1328L	-0.29	Decrease	5
rs1411224615	L1326V	-1.44	Decrease	5
rs780926114	K1323N	-0.42	Decrease	4
rs754203974	K1323R	-0.21	Decrease	4
rs1328566140	E1322K	-0.51	Decrease	7
rs757703243	G1321E	-0.45	Decrease	0
rs1307854066	P1336R	-0.95	Decrease	6
rs1435919291	P1336S	-1.44	Decrease	8
rs779490963	A1335G	-1.2	Decrease	7
rs779967013	A1318T	-0.71	Decrease	7
rs1044312254	I1315M	-1.21	Decrease	8
rs1197552228	G1321S	-0.98	Decrease	7
rs767041788	G1397S	-1.03	Decrease	5
rs1442277728	C1294S	-0.69	Decrease	6
rs1182970957	C1294G	-1.22	Decrease	8
rs564106859	R1289H	-1.18	Decrease	9
rs200312526	R1289C	-0.9	Decrease	4
rs1010743136	R1285H	-1.28	Decrease	9
rs755492225	R1285C	-0.96	Decrease	5
rs748262646	R1276H	-1.73	Decrease	9
rs1235846614	V1266I	-0.96	Decrease	8
rs1343782138	E1266D	-0.6	Decrease	4
rs1178617228	L1280I	-1.34	Decrease	8
rs535946725	F1262L	-1.65	Decrease	8
rs774073234	R1261Q	-1.39	Decrease	9
rs1052868434	R1261W	-0.74	Decrease	6
rs1296808828	R1259W	-0.65	Decrease	6
rs1168306248	D1256N	-1.18	Decrease	8
rs1488049319	V1249A	-2.18	Decrease	9
rs1408962462	V1248M	-1.27	Decrease	7
rs1085307800	Y1240C	-1.46	Decrease	3
rs780156714	R1238H	-1.55	Decrease	8
rs746456488	R1238C	-1.23	Decrease	2
rs1290055474	S1253L	-0.51	Decrease	5
rs768280180	N1236S	-0.77	Decrease	5
rs759915904	G1231R	-0.34	Decrease	3
rs767953016	P1212A	-1.44	Decrease	8
rs753205659	R1210Q	-0.8	Decrease	8
rs1269778997	R1210W	-0.18	Decrease	3
rs1255396898	G1208D	-0.88	Decrease	4
rs764137405	G1208S	-1.26	Decrease	7
rs757460628	R1207H	-1.11	Decrease	8
rs779029407	R1207C	-0.81	Decrease	1
rs142648642	T1204I	-0.27	Decrease	5
rs201497993	T1203N	-0.9	Decrease	5
rs780063940	T1203A	-1.04	Decrease	8

rs1223800609	A1200G	-1.42	Decrease	9
rs746926863	L1197M	-1.12	Decrease	8
rs1367702161	T1185I	-0.85	Decrease	8
rs780717834	T1183S	-1.37	Decrease	9
rs1255153711	R1178W	-0.28	Decrease	9
rs1434985283	A1176V	-0.22	Decrease	4
rs772419584	A1173V	-0.27	Decrease	4
rs1193652618	P1172R	-0.65	Decrease	4
rs1412363660	A1166T	-0.5	Decrease	8
rs151305495	Q1573H	-0.56	Decrease	5
rs754742754	V1144L	-1.47	Decrease	9
rs767421908	R1140Q	-0.93	Decrease	9
rs752571105	K1135R	-0.27	Decrease	6
rs756132614	I1132M	-1.35	Decrease	8
rs545615552	I1132T	-1.8	Decrease	8
rs1412256768	P1130R	-0.63	Decrease	5
rs762819722	E1129K	-0.29	Decrease	5
rs201308454	S1122C	-0.31	Decrease	1
rs770370709	G1118D	-0.56	Decrease	2
rs1049058944	G1118S	-0.82	Decrease	3
rs1284939504	G1116R	-0.31	Decrease	0
rs933543639	N1108S	-0.05	Decrease	5
rs1202584286	S1105N	-0.53	Decrease	0
rs200950656	R1104C	0.99	Decrease	4
rs150331990	H1102R	-0.22	Decrease	3
rs534263445	N1101K	-0.35	Decrease	3
rs759653796	P1099L	-0.55	Decrease	0
rs755865204	P1099S	-1.64	Decrease	9
rs1479104782	F1096L	-0.96	Decrease	1
rs377078524	R1082H	-1.45	Decrease	9
rs1316623288	R1082C	-1.06	Decrease	3
rs747265409	G1079S	-1.26	Decrease	8
rs950686369	G1078C	-1	Decrease	5
rs370075258	M1077K	-1.49	Decrease	6
rs768431034	M1077V	-0.81	Decrease	8
rs1194144103	V1074L	-1.03	Decrease	6
rs776461147	V1072I	-0.51	Decrease	6
rs1276680499	L1068V	-1.65	Decrease	7
rs560179619	V1062M	-0.93	Decrease	8
rs757791181	T1061A	-0.97	Decrease	7
rs779489678	R1059H	-1.39	Decrease	9
rs140852137	R1059C	-0.94	Decrease	6
rs747215182	A1055P	-0.29	Decrease	0
rs1426702144	K1054R	-0.18	Decrease	2
rs776374384	F1053L	-1.04	Decrease	6
rs1478047497	V1051G	-2.34	Decrease	10
rs187394074	V1050M	-1.11	Decrease	8
rs762862182	A1049V	-0.24	Decrease	5
rs548883904	D1046N	-0.74	Decrease	3
rs1160158444	Y1043C	-1.04	Decrease	3

rs767308840	N1040S	-0.15	Decrease	6
rs754199607	I1039V	-1.13	Decrease	8
rs1350013741	A1037G	-1.28	Decrease	8
rs370786558	A1037T	-0.63	Decrease	5
rs758882865	A1049V	-0.24	Decrease	5
rs780153466	T1031I	-0.41	Decrease	2
rs751674187	T1031A	-1.48	Decrease	7
rs774833250	N1019K	-0.33	Decrease	3
rs199827346	R1017Q	-1.51	Decrease	9
rs144533539	R1017W	-0.8	Decrease	7
rs764244867	I1014V	-1.41	Decrease	8
rs1282355438	T1012I	-1.08	Decrease	9
rs1233155491	E1011D	-0.43	Decrease	4
rs373940840	N1026S	-0.6	Decrease	6
rs866159764	P1009L	-0.86	Decrease	4
rs756998907	R1008K	-1.03	Decrease	9
rs1256073808	G1007S	-1.44	Decrease	9
rs1221584713	S1005T	-0.33	Decrease	0
rs1248096623	K1020T	-0.62	Decrease	5
rs1487276325	P1002L	-0.58	Decrease	2
rs1196329826	P1002S	-1.56	Decrease	8
rs1199169323	F1000S	-1.75	Decrease	7
rs745780816	E998D	-0.27	Decrease	2
rs1330228268	K997R	-0.42	Decrease	8
rs758453625	K997E	-0.8	Decrease	8
rs376854079	I996M	-1.4	Decrease	9
rs1167927296	I996T	-1.87	Decrease	8
rs876661227	R995W	-0.4	Decrease	6
rs746527645	R1008Q	-1.35	Decrease	9
rs776149246	Y991C	-1.2	Decrease	4
rs747897637	E989K	-0.62	Decrease	7
rs760003805	Y976H	-1.51	Decrease	7
rs775590756	P970A	-1.46	Decrease	6
rs1056948381	Y969H	-1.39	Decrease	6
rs760683699	L968M	-1.45	Decrease	7
rs764301881	D967E	-0.27	Decrease	1
rs753992945	D965A	-0.64	Decrease	3
rs1489509959	D965N	-0.81	Decrease	4
rs757550838	V964M	-1.08	Decrease	8
rs895501426	E962G	-1.32	Decrease	7
rs750276897	R960Q	-1.2	Decrease	9
rs148038464	V956M	-1.2	Decrease	8
rs1328974927	P955T	-1.37	Decrease	8
rs1362626891	S953C	-0.71	Decrease	4
rs754990148	T947M	-0.47	Decrease	3
rs978444139	F946L	-1.37	Decrease	8
rs780533354	F946I	-1.37	Decrease	8
rs755712065	V939L	-1.44	Decrease	9
rs1181438211	D937N	-1.39	Decrease	8
rs1378414629	I931M	-2.02	Decrease	8

rs1478243877	I931N	-2.27	Decrease	6
rs1174048074	I931L	-1.15	Decrease	8
rs977832207	G930V	-0.58	Decrease	7
rs777416084	G930S	-1.66	Decrease	9
rs1302114385	N929D	-0.41	Decrease	2
rs1349231023	T927A	-1.86	Decrease	9
rs747445212	S925A	-1.26	Decrease	8
rs1389160994	Y923S	-1.64	Decrease	9
rs762002107	L922P	-1.9	Decrease	5
rs770079133	L922V	-1.84	Decrease	8
rs773559669	V921L	-1.22	Decrease	5
rs763321599	R920Q	-0.99	Decrease	8
rs550380640	R920G	-1.41	Decrease	8
rs910412179	D916N	-1.06	Decrease	6
rs767511749	E915K	-0.95	Decrease	8
rs1453506357	Q913L	-0.12	Decrease	3
rs755625901	E912K	-0.88	Decrease	8
rs777323413	L911P	-1.69	Decrease	4
rs973184667	R909T	-0.66	Decrease	8
rs1360494258	E906K	-0.55	Decrease	8
rs753467933	E901K	-0.85	Decrease	9
rs756944531	R898H	-1.34	Decrease	9
rs745753378	R898C	-0.89	Decrease	5
rs768990499	F892C	-1.75	Decrease	7
rs1057518774	F890L	-1.34	Decrease	6
rs1204551824	Q883E	-0.8	Decrease	7
rs201213597	T882I	-0.3	Decrease	1
rs746143694	K881E	-0.48	Decrease	6
rs1379893711	P880S	-1.65	Decrease	8
rs775305891	P879S	-1.62	Decrease	8
rs1303994790	A874V	-0.11	Decrease	5
rs569867388	Y873C	-1.11	Decrease	1
rs1336107599	D870E	0.05	Decrease	0
rs367681882	D870H	-0.18	Decrease	5
rs1314966532	F864L	-1	Decrease	7
rs62621087	G860R	-0.29	Decrease	0
rs749258509	D859N	-0.63	Decrease	1
rs571299133	D858G	-1.01	Decrease	5
rs779777304	E856G	-1.28	Decrease	7
rs768517249	L854P	-1.46	Decrease	5
rs538290672	S853C	-0.21	Decrease	0
rs761717775	E852K	-0.52	Decrease	7
rs1176297175	M849V	-0.66	Decrease	7
rs758465656	M845I	-0.52	Decrease	4
rs751754286	E841K	-0.61	Decrease	8
rs868584117	S840C	-0.4	Decrease	1
rs781003939	P839T	-1.07	Decrease	5
rs1177086967	A838V	-0.3	Decrease	3
rs1363883264	K837R	-0.33	Decrease	5
rs1422521201	I835V	-1.28	Decrease	8

rs747921118	S830G	-1.04	Decrease	8
rs1345541911	M823T	-1.07	Decrease	8
rs1228333897	P811L	-0.66	Decrease	7
rs963570572	P811S	-1.46	Decrease	8
rs140993011	T808M	-0.14	Decrease	0
rs183555527	G806R	-0.39	Decrease	2
rs373366822	A799T	-0.56	Decrease	5
rs988229673	A794G	-1.27	Decrease	8
rs1244845928	D785H	-0.38	Decrease	2
rs757300501	A781G	-1.39	Decrease	9
rs148987580	T780M	-0.43	Decrease	3
rs899735320	T780A	-1.41	Decrease	8
rs1223065154	A777S	-0.8	Decrease	9
rs1181332562	Y775C	-1.06	Decrease	2
rs113497353	L774P	-1.7	Decrease	5
rs1201583942	P773L	-0.96	Decrease	7
rs1313976937	S786C	-0.29	Decrease	0
rs1219579994	V765L	-1.36	Decrease	8
rs1330899035	C762G	-1.37	Decrease	7
rs778066229	V759M	-0.93	Decrease	8
rs1346601061	E758G	-1.19	Decrease	9
rs1407084701	E758K	-0.74	Decrease	9
rs142562681	T756N	-0.86	Decrease	2
rs573248196	A754V	-0.11	Decrease	1
rs1251285527	I752T	-1.97	Decrease	5
rs1272046307	I752V	-1.09	Decrease	7
rs760149381	C751G	-1.2	Decrease	8
rs760768778	K743Q	-0.48	Decrease	1
rs764389789	D741G	-0.92	Decrease	4
rs762172122	V738I	-0.35	Decrease	6
rs1381758934	G735R	-0.73	Decrease	6
rs1263023164	P710S	-1.32	Decrease	6
rs767075259	I709F	-1.18	Decrease	5
rs375620967	V705I	-0.22	Decrease	1
rs1037000664	E703D	-0.61	Decrease	7
rs1336324296	N693S	-0.25	Decrease	7
rs766157757	R689W	-0.2	Decrease	3
rs774333932	Q687R	-0.19	Decrease	2
rs1029809799	S682C	-0.53	Decrease	4
rs1307537239	S679T	-0.12	Decrease	0
rs1486893166	V655I	-0.06	Decrease	4
rs760173233	G654S	-1.01	Decrease	8
rs1359313210	R651Q	-1.03	Decrease	9
rs763570334	R651W	-0.48	Decrease	6
rs753503985	R650H	-1.48	Decrease	9
rs761410935	R650C	-1.12	Decrease	6
rs766722875	F648L	-1.47	Decrease	5
rs1292456314	A647G	-1.53	Decrease	7
rs146467216	A647T	-0.83	Decrease	5
rs1016379814	N646S	-0.56	Decrease	7

rs1445415598	N646D	-0.58	Decrease	1
rs781764761	E645D	-0.69	Decrease	6
rs756254318	E642Q	-0.53	Decrease	5
rs1228041705	A633T	-0.7	Decrease	6
rs1162379853	R613K	-0.62	Decrease	5
rs776032838	R609T	-0.89	Decrease	6
rs761476734	T608S	-0.9	Decrease	7
rs978864962	R605S	-1.09	Decrease	7
rs751902349	R601Q	-0.56	Decrease	4
rs753235229	A599V	-0.09	Decrease	4
rs1375945352	A599T	-0.6	Decrease	7
rs777870040	A597D	-0.45	Decrease	3
rs397509391	V590F	-1.19	Decrease	8
rs397509393	G589E	-0.41	Decrease	0
rs775941265	M581T	-0.83	Decrease	7
rs1420508762	M581L	-0.88	Decrease	8
rs760952579	P579S	-1.56	Decrease	8
rs762421501	T578I	-0.25	Decrease	1
rs1330611538	I575V	-0.88	Decrease	8
rs758553732	E572D	-0.63	Decrease	6
rs1201469324	S570T	-0.33	Decrease	2
rs959058396	D569H	-0.19	Decrease	0
rs1227919940	G568R	-0.32	Decrease	2
rs990456303	E566K	-0.8	Decrease	8
rs397509392	A554G	-1.24	Decrease	7
rs1003447106	A554T	-0.68	Decrease	7
rs748821501	R552Q	-1	Decrease	9
rs768869946	D548N	-1.05	Decrease	6
rs375474222	R544C	-1.17	Decrease	5
rs1315979941	G539V	-0.53	Decrease	7
rs763014417	T534M	-0.49	Decrease	5
rs368761660	T534A	-1.41	Decrease	8
rs556213169	T533I	-0.44	Decrease	6
rs865891586	E532K	-0.66	Decrease	8
rs1269876059	I531F	-1.77	Decrease	7
rs774523299	T523I	-0.38	Decrease	2
rs767797868	D521N	-1.32	Decrease	7
rs760542365	F515V	-1.61	Decrease	8
rs1405357614	P497L	-0.13	Decrease	0
rs1064796687	A496E	-0.5	Decrease	4
rs753794138	A496S	-0.79	Decrease	8
rs199473690	Y495C	-0.93	Decrease	2
rs199473692	Y495H	-1.22	Decrease	6
rs561762200	E494D	-0.37	Decrease	4
rs750390469	I477L	-0.35	Decrease	2
rs1487562713	A475S	-0.58	Decrease	5
rs1190635928	E473K	-0.57	Decrease	7
rs374027926	E463A	-0.46	Decrease	4
rs979325432	L449V	-1.67	Decrease	7
rs1308265840	P447S	-1.77	Decrease	8

rs1403539644	D444H	-0.95	Decrease	6
rs762835601	F434L	-1.09	Decrease	5
rs1345721072	L449R	-1.72	Decrease	6
rs751093624	E432K	-0.72	Decrease	8
rs1181209213	D423N	-0.49	Decrease	3
rs758850158	G417S	-1.02	Decrease	6
rs777569395	S411N	-0.19	Decrease	0
rs867003508	E400K	-0.61	Decrease	7
rs367727676	N392S	-0.29	Decrease	6
rs966116269	K385N	-0.65	Decrease	5
rs1288550596	K386R	-0.32	Decrease	6
rs142673915	N383D	-0.37	Decrease	6
rs1161974493	P372Q	-1.16	Decrease	7
rs776120784	P372A	-0.99	Decrease	4
rs774875858	D362N	-0.33	Decrease	0
rs1294787183	Q358H	-0.62	Decrease	7
rs1326232301	I354S	-2.18	Decrease	9
rs1316802867	I354L	-0.93	Decrease	9
rs756138932	L351P	-0.46	Decrease	4
rs1333537251	L350P	-0.41	Decrease	2
rs761967857	N345K	0.03	Decrease	0
rs1064796441	M344I	-0.47	Decrease	4
rs1464709973	V343I	-0.17	Decrease	6
rs765440005	T342K	-0.75	Decrease	5
rs750700551	K341R	-0.12	Decrease	0
rs758106190	K341Q	-0.36	Decrease	0
rs529074384	A340T	-0.65	Decrease	4
rs201945078	R339H	-0.95	Decrease	6
rs1390264889	M337I	-0.42	Decrease	3
rs544428951	M337T	-0.67	Decrease	2
rs775954754	T333K	-0.74	Decrease	7
rs370592431	P332L	-0.11	Decrease	4
rs762344471	K330R	-0.14	Decrease	3
rs1459359320	K330E	-0.37	Decrease	3
rs779486758	P329H	-0.82	Decrease	5
rs773301158	T327M	-0.19	Decrease	3
rs763026348	K326R	-0.3	Decrease	4
rs781477930	E317A	-0.42	Decrease	4
rs748515801	E317K	-0.62	Decrease	6
rs1279218298	E314D	-0.6	Decrease	7
rs61758431	I311M	-1.23	Decrease	7
rs2228612	I311V	-1.14	Decrease	6
rs143287044	Q310P	-0.5	Decrease	4
rs1483769228	E321K	-0.52	Decrease	6
rs1179101598	E319D	-0.57	Decrease	6
rs1231795266	K302R	-0.22	Decrease	4
rs200601847	E300K	-0.68	Decrease	8
rs1434425247	R298G	-1.17	Decrease	8
rs775360871	R297Q	-0.77	Decrease	7
rs1372902099	R297W	-0.3	Decrease	4

rs544747966	K296R	-0.14	Decrease	2
rs1406374567	A296D	-0.73	Decrease	5
rs559861049	A294V	-0.37	Decrease	2
rs61758430	L293V	-1.63	Decrease	8
rs148831705	K291E	-0.26	Decrease	3
rs761747950	P290T	-1.25	Decrease	8
rs368346471	Q289H	-0.64	Decrease	7
rs1301997168	R287G	-0.99	Decrease	7
rs762434544	H286R	-0.15	Decrease	0
rs751183319	K285Q	-0.5	Decrease	3
rs759143980	K284Q	-0.55	Decrease	3
rs1257025095	K276R	-0.71	Decrease	7
rs200024502	E274K	-0.67	Decrease	7
rs766504703	V270A	-0.66	Decrease	5
rs368960099	V270M	-0.55	Decrease	3
rs1199842664	R267G	-1.06	Decrease	7
rs1395459443	A266V	-0.43	Decrease	4
rs1436304759	A366S	-0.91	Decrease	9
rs1419316960	R264G	-1.05	Decrease	8
rs1390469147	E261K	-0.66	Decrease	8
rs755835618	E276Q	-0.49	Decrease	4
rs1232498004	T253A	-0.79	Decrease	7
rs779634956	P252R	-0.51	Decrease	3
rs1419372864	R246Q	-0.84	Decrease	7
rs1459602995	L245R	-1.35	Decrease	6
rs746582737	E242G	-0.81	Decrease	3
rs1163672521	E241G	-0.81	Decrease	3
rs768339415	E251D	-0.24	Decrease	3
rs528992391	K233E	-0.4	Decrease	4
rs370064676	T231N	-0.83	Decrease	2
rs771381056	R246H	-1.11	Decrease	8
rs201749864	R246C	-0.78	Decrease	2
rs143598088	T229M	-0.13	Decrease	1
rs1211520360	T229A	-0.65	Decrease	5
rs992035736	A225G	-1.25	Decrease	6
rs764212584	E239D	-0.51	Decrease	5
rs926180987	P222L	-2.01	Decrease	9
rs1319952912	E221V	-1.51	Decrease	9
rs758190156	P216L	-0.65	Decrease	5
rs374856119	P216T	-1.03	Decrease	8
rs568861011	R215K	-0.77	Decrease	7
rs777469378	R212Q	-0.95	Decrease	8
rs780431940	V207I	-0.26	Decrease	2
rs747469397	R205H	-1.37	Decrease	9
rs1194542262	R205C	-0.99	Decrease	5
rs1244292821	R204K	-0.95	Decrease	8
rs1474694064	E202G	-1.11	Decrease	3
rs756886439	K198N	-0.61	Decrease	1
rs751913403	I193L	-0.47	Decrease	1
rs748641297	A287G	-1.14	Decrease	5

rs769876242	R186K	-1.24	Decrease	6
rs1208887534	R186G	-0.7	Decrease	6
rs1476219241	P180R	-0.67	Decrease	3
rs900075322	R178W	-0.24	Decrease	3
rs62621089	A176V	-0.19	Decrease	0
rs1375806732	G174S	-1.1	Decrease	8
rs374440818	K160R	-0.33	Decrease	5
rs1020363356	K160Q	-0.66	Decrease	4
rs201319352	T158P	-0.49	Decrease	0
rs1334578298	R156K	-0.71	Decrease	5
rs1462015831	R156G	-1.39	Decrease	6
rs978575291	P155R	-0.79	Decrease	6
rs886054129	P151R	-0.89	Decrease	6
rs1327078717	P151T	-1.13	Decrease	7
rs756038357	E150G	-1.3	Decrease	7
rs764561233	R149S	-1.54	Decrease	8
rs1140470	A147G	-1.07	Decrease	5
rs754359114	A147S	-0.54	Decrease	7
rs1292830551	G145A	-0.78	Decrease	4
rs765353997	D144N	-0.97	Decrease	4
rs1193001884	S143A	-0.7	Decrease	4
rs377146699	T137R	-0.38	Decrease	0
rs377146700	T137M	-0.4	Decrease	3
rs775139340	R136H	-1.11	Decrease	8
rs775139341	R136L	-0.56	Decrease	7
rs138841970	R136C	-0.9	Decrease	3
rs1361938846	P135H	-1.28	Decrease	7
rs769726219	P135S	-1.43	Decrease	8
rs748783652	L132I	-1.1	Decrease	6
rs1057518769	P131S	-1.5	Decrease	9
rs370207020	P129H	-1.45	Decrease	8
rs370207021	P129R	-0.91	Decrease	5
rs1085307715	P129A	-1.3	Decrease	7
rs1425787169	P128R	-1.01	Decrease	6
rs1425787170	P128L	-0.54	Decrease	3
rs146601335	P128T	-1.29	Decrease	8
rs146601336	P128S	-1.74	Decrease	9
rs751681312	N126T	-0.03	Decrease	2
rs1417767085	A125T	-0.75	Decrease	7
rs1085307725	A123G	-1.5	Decrease	8
rs780782457	M122V	-1	Decrease	5
rs1397430053	G121E	-0.52	Decrease	1
rs1331733943	G121R	-0.42	Decrease	1
rs373923585	R119S	-1.14	Decrease	9
rs777666240	R119T	-0.77	Decrease	7
rs146516082	R119G	-1.34	Decrease	6
rs149362098	R118H	-1.14	Decrease	7
rs149362099	R118L	-0.24	Decrease	4
rs745455817	R118C	-0.79	Decrease	2
rs554894511	R114K	-0.95	Decrease	9

rs762241565	A113G	-1.46	Decrease	8
rs906790753	N111S	-0.54	Decrease	7
rs376894659	G110R	-0.43	Decrease	3
rs1157913728	N109D	-0.39	Decrease	3
rs751662619	R106H	-1.32	Decrease	8
rs759600542	R106C	-0.99	Decrease	4
rs1170262960	N104D	-0.33	Decrease	3
rs1039555172	E102Q	-0.52	Decrease	6
rs1401130665	R101Q	-0.92	Decrease	8
rs369196079	R101W	-0.47	Decrease	6
rs752966527	N100K	-0.49	Decrease	6
rs1375073063	N100S	-0.5	Decrease	6
rs755873606	A98S	-0.95	Decrease	9
rs16999593	H97R	-0.3	Decrease	4
rs1338494594	A96P	-0.52	Decrease	3
rs778222019	N94D	-0.39	Decrease	5
rs779818604	L90V	-1.5	Decrease	7
rs953187775	N87S	-0.25	Decrease	4
rs984728792	G77V	-0.28	Decrease	0
rs746687493	G77S	-1.04	Decrease	6
rs746687494	G77R	-0.41	Decrease	1
rs1206145138	E75K	-0.57	Decrease	7
rs1477841278	E71G	-1	Decrease	7
rs1279416770	K70T	-0.3	Decrease	4
rs61750053	R69H	-0.94	Decrease	7
rs61750054	R69L	-0.19	Decrease	7
rs753673660	R69C	-0.57	Decrease	0
rs1351910032	N59K	0.17	Decrease	0
rs375585911	T55A	-0.82	Decrease	7
rs1401965330	E51D	-0.23	Decrease	3
rs755995375	E51K	-0.44	Decrease	7
rs1234060339	L46F	-0.62	Decrease	2
rs757938210	K45R	0	Decrease	1
rs1242559756	E44K	-0.43	Decrease	6
rs1264838175	K43R	-0.12	Decrease	3
rs779730617	C41W	-0.07	Decrease	1
rs1379799242	E38K	-0.64	Decrease	8
rs374817622	R33G	-1.46	Decrease	8
rs753721329	R27Q	-1.1	Decrease	9
rs780503694	R26S	-1.36	Decrease	9
rs1340824034	R26K	-1.09	Decrease	9
rs1206120198	R25C	-1.09	Decrease	7
rs1271779036	V24I	-0.64	Decrease	7
rs1257821053	D23N	-1.05	Decrease	5
rs1224343919	D22N	-1.04	Decrease	6
rs747559452	S19L	-0.19	Decrease	5
rs1476955893	P16Q	-1.48	Decrease	9
rs1324769959	P16S	-1.62	Decrease	9
rs1395421881	V15A	-1.4	Decrease	9
rs769897113	V15I	-0.57	Decrease	7

rs1348019056	L13Q	-1.87	Decrease	8
rs762812098	L13M	-1.28	Decrease	8
rs1170521928	T12A	-0.73	Decrease	4
rs1216341078	P11S	-1.41	Decrease	9
rs1269997956	V10M	-0.86	Decrease	7
rs759577079	R9Q	-0.54	Decrease	5
rs994411260	A8G	-1.07	Decrease	7
rs1217509888	A8S	-0.62	Decrease	8
rs766984573	P7S	-1.34	Decrease	9
rs1487433053	A6T	-0.62	Decrease	7
rs1248298976	P2R	-0.61	Decrease	4
rs763647098	P2A	-0.99	Decrease	7
rs753525461	M1V	-0.64	Decrease	6
rs1388362405	S1556F	0.14	Increase	3
rs1316990553	N1529I	1	Increase	5
rs769347864	S1477F	-0.03	Increase	0
rs1167554550	S1467I	-0.01	Increase	0
rs770284928	Q1404E	-0.12	Increase	2
rs1323856557	R1401W	0.11	Increase	1
rs375225009	S1382L	0.07	Increase	1
rs1292991293	S1372F	0.21	Increase	3
rs766051225	A1318V	-0.02	Increase	2
rs1312709316	G1222S	-0.74	Increase	1
rs1353293786	D1216N	-0.75	Increase	2
rs764541314	T1163M	0.07	Increase	1
rs1345421531	D1162E	0.44	Increase	7
rs776723837	D1162H	0.23	Increase	5
rs375976847	P1127L	-0.15	Increase	0
rs920360352	C1125Y	-0.2	Increase	1
rs1011300495	Q1123K	-0.14	Increase	2
rs768902636	S1122P	-0.15	Increase	1
rs374676749	H1102Y	-0.04	Increase	6
rs1217143527	Q1073E	-0.32	Increase	3
rs1460029697	S895R	-0.08	Increase	4
rs150863675	V910L	-0.61	Increase	0
rs1287452669	N888I	1.05	Increase	6
rs1262319814	T885R	-0.28	Increase	2
rs530293931	Q883H	-0.35	Increase	3
rs771879227	P879L	-0.27	Increase	0
rs1160065459	S878F	0.48	Increase	7
rs750081202	Q866R	-0.04	Increase	3
rs1305123223	Q866K	-0.3	Increase	2
rs758032585	K861N	-0.34	Increase	2
rs753140952	G860E	-0.44	Increase	1
rs772216000	G857E	-0.38	Increase	2
rs1402220036	H795Y	0.42	Increase	6
rs1379915323	S786R	0.16	Increase	7
rs1468958840	S786R	0.16	Increase	7
rs865989923	S786F	0.56	Increase	7
rs370056750	E761D	-0.12	Increase	6

rs1402672876	C751Y	-0.11	Increase	0
rs1195515645	K748Q	-0.44	Increase	0
rs1224353267	K743R	-0.19	Increase	0
rs910109905	N729D	0.11	Increase	4
rs548322208	K722Q	-0.32	Increase	1
rs754509457	P713L	-0.2	Increase	3
rs891162524	M712R	-0.61	Increase	3
rs752326785	D706G	-0.69	Increase	6
rs1254979538	D706N	-0.61	Increase	3
rs368319266	D702N	-0.4	Increase	4
rs767317190	F676Y	-0.53	Increase	0
rs752235220	K668T	-0.04	Increase	1
rs1316701284	D640N	-0.54	Increase	2
rs1181179755	Y625C	-0.67	Increase	7
rs1359908894	S538Y	-0.21	Increase	3
rs1037636262	S538L	-0.04	Increase	0
rs181300723	H416Y	0.36	Increase	6
rs141562679	K366I	0.49	Increase	4
rs747668607	R339C	-0.59	Increase	2
rs749001465	K335Q	-0.17	Increase	3
rs1361695976	R325C	-0.56	Increase	1
rs1440048632	D316G	-0.91	Increase	3
rs996476593	Y313D	-0.1	Increase	5
rs1210097019	P309L	-0.19	Increase	3
rs1478185698	E317V	-0.25	Increase	0
rs765923835	K285R	-0.19	Increase	0
rs1169597458	D275A	0	Increase	2
rs750916721	D273E	0.14	Increase	6
rs781257220	D279G	-0.69	Increase	4
rs1430311136	P262L	-0.3	Increase	2
rs1393687942	E261V	0.02	Increase	1
rs372129479	E276V	-0.18	Increase	0
rs1391845033	E251V	0.19	Increase	3
rs1435887591	D238E	-0.1	Increase	5
rs150999369	G228E	-0.39	Increase	1
rs775928642	S227P	-0.05	Increase	6
rs762056108	P222S	-0.17	Increase	0
rs769029526	A219T	0.33	Increase	1
rs758761731	S209T	-0.33	Increase	1
rs1281646304	D190N	-0.58	Increase	4
rs781646588	S189L	0.28	Increase	6
rs1414937646	S141R	-0.12	Increase	5
rs773200699	S133F	0.02	Increase	6
rs75616428	V120L	-0.26	Increase	4
rs753670606	H97Y	-0.07	Increase	5
rs1268674999	S91F	0.23	Increase	6
rs1456240920	K88E	-0.01	Increase	1
rs1218241317	C41R	-0.18	Increase	1
rs751109006	S35N	-0.45	Increase	1
rs768745074	A17V	-0.15	Increase	0

rs760288360	A3V	0.05	Increase	1

Analysis of DNMT1 Gene Variants in Progression of Neural Tube Defects- an *insilico* to *invitro* approach

Susanta Sadhukhan[1†], Nirvika Paul[1†], Sudakshina Ghosh[2], Dinesh Munian[3], Kausik Ganguly[4], Krishnendu Ghosh[1], Mainak Sengupta[4], Madhusudan Das[1,*]

[1]Department of Zoology, University of Calcutta, 35 Ballygunge Circular Road, Kolkata-700019, India
[2]Department of Zoology, Vidyasagar College for Women, 39 Sankar Ghosh Lane, Kolkata-700006, India
[3]Department of Neonatology, Institute of Postgraduate Medical Education & Research, 244 Acharya Jagadish Chandra Bose Road, Kolkata, 700020, India
[4]Department of Genetics, University of Calcutta, 35 Ballygunge Circular Road, Kolkata-700019, India

[*]Corresponding author at the Dept. of Zoology, University of Calcutta: madhuzoo@yahoo.com/ mdzoo@caluniv.ac.in (mail id)
†Authors contributed equally

Supplementary Table 2: Results of *in silico* analyses of synonymous variants (syn SNVs) of *DNMT1*. Result table include all those 82 syn SNVs for which codon usage were found to alter 2 folds or more upon concerned allelic changes. Interesting for all these 82 syn SNVs significant alterations in secondary mRNA structures were also found after mFold analyses and concerned delta G values were noted down in this table. It should be noted here that this table contains those syn SNVs too for which we did not find significant changes in exon skipping after ex-skip analyses along with those syn SNVs for which significant changes were observed after ex-skip analyses.

Supplementary Table 2A: Synonymous single nucleotide variants (Syn SNVs) of *DNMT1* for which 2 folds or more changes in Codon usages were found

SNP Id	Coded amino acid	Codon 1	Frequency of usage	Codon 2	Frequency of usage	Change in usage (difference only)	Change in usage (fold change)	ALLELE 1 (A1)	DELTA G VALUE (kcal/mol)	ALLELE 2 (A2)	DELTA G VALUE (kcal/mol)	Energy change (A2 - A1)	Ex-skip predictions
rs147713850	Leu	TTG	0.13	CTG	0.4	0.27	3.0769231	T	-186.91	C	-181.7	5.21	Both alleles have a comparable chance of exon skipping.
rs368325985	Thr	ACG	0.11	ACA	0.28	0.17	2.5454545	G	-80.18	A	-71.7	8.48	Allele seq-mut has a higher chance of exon skipping than allele seq-wt.
rs184125970	Thr	ACG	0.11	ACA	0.28	0.17	2.5454545	G	-157.96	A	-158.97	-1.01	Both alleles have a comparable chance of exon skipping.
rs372988540	Ala	GCG	0.11	GCA	0.23	0.12	2.0909091	G	-78.46	A	-76.16	2.3	Allele seq-wt has a higher
													chance of exon skipping than allele seq-mut.
rs201007703	Ser	TCG	0.05	TCA	0.15	0.1	3	G	-154.76	A	-153.6	1.16	Allele seq-wt has a higher chance of exon skipping than allele seq-mut.
rs772752543	Ala	GCG	0.11	GCA	0.23	0.12	2.0909091	G	-22.1	A	17.3	39.4	Allele seq-wt has a higher chance of exon skipping than allele seq-mut.
rs761140414	Thr	ACG	0.11	ACA	0.28	0.17	2.5454545	G	-18.2	A	-13.6	4.6	Allele seq-wt has a higher chance of exon skipping than allele seq-mut.
rs199739823	Ser	TCG	0.05	TCA	0.15	0.1	3	G	-136.65	A	-135.74	0.91	Allele seq-wt has a higher chance of exon skipping than allele seq-mut.
rs750931983	Ala	GCG	0.11	GCA	0.23	0.12	2.0909091	G	-12.5	A	-11.4	1.1	Allele seq-wt has a higher chance of exon skipping than allele seq-mut.
rs372694243	Ala	GCG	0.11	GCA	0.23	0.12	2.0909091	G	-72.45	A	-69.75	2.7	Allele seq-mut has a higher chance of exon skipping than allele seq-wt.

rs749216491	Ala	GCG	0.11	GCA	0.23	0.12	2.0909091	G	-13	A	-9.5	3.5	Allele seq-wt has a higher chance of exon skipping than allele seq-mut.
rs778997572	Pro	CCG	0.11	CCA	0.28	0.17	2.5454545	G	-7.7	A	-8.2	-0.5	Allele seq-wt has a higher chance of exon skipping than allele seq-mut.
rs771365059	Ala	GCG	0.11	GCA	0.23	0.12	2.0909091	G	-5.9	A	-5.2	0.7	Allele seq-wt has a higher chance of exon skipping than allele seq-mut.
rs761852984	Ala	GCG	0.11	GCA	0.23	0.12	2.0909091	G	-14.9	A	-14.1	0.8	Both alleles have a comparable chance of exon skipping.
rs748007341	Ala	GCG	0.11	GCA	0.23	0.12	2.0909091	G	-17.4	A	-16.6	0.8	Allele seq-wt has a higher chance of exon skipping than allele seq-mut.
rs1459010920	Ser	TCG	0.05	TCA	0.15	0.1	3	G	-11.4	A	-11.4	0	Allele seq-mut has a higher chance of exon skipping than allele seq-wt.
rs764039807	Ala	GCG	0.11	GCA	0.23	0.12	2.0909091	G	-12.6	A	-11.6	1	Allele seq-wt has a higher chance of exon skipping than allele seq-mut.
rs749045750	Ala	GCG	0.11	GCA	0.23	0.12	2.0909091	G	-12.2	A	-13	-0.8	Allele seq-wt has a higher
													chance of exon skipping than allele seq-mut.
rs991716655	Thr	ACG	0.11	ACA	0.28	0.17	2.5454545	G	-7.6	A	-6.8	0.8	Allele seq-mut has a higher chance of exon skipping than allele seq-wt.
rs748449856	Pro	CCG	0.11	CCA	0.28	0.17	2.5454545	G	-11.4	A	-9.9	1.5	Allele seq-mut has a higher chance of exon skipping than allele seq-wt.
rs750234435	Pro	CCG	0.11	CCA	0.28	0.17	2.5454545	G	-9	A	-8.1	0.9	Allele seq-wt has a higher chance of exon skipping than allele seq-mut.
rs369311643	Thr	ACG	0.11	ACA	0.28	0.17	2.5454545	G	-54.25	A	-52.55	1.7	Allele seq-wt has a higher chance of exon skipping than allele seq-mut.
rs893895641	Leu	CTA	0.07	CTG	0.4	0.33	5.7142857	A	-4.5	G	-4.5	0	Allele seq-mut has a higher chance of exon skipping than allele seq-wt.
rs754986621	Leu	TTG	0.13	CTG	0.4	0.27	3.0769231	T	-5.7	C	-6.3	-0.6	Allele seq-mut has a higher chance of exon skipping than allele seq-wt.

rs1456849796	Ile	ATA	0.17	ATC	0.47	0.3	2.7647059	A	-8	C	-4.88	3.12	Allele seq-wt has a higher chance of exon skipping than allele seq-mut.
rs780874970	Leu	CTC	0.2	CTG	0.4	0.2	2	C	-9.7	G	-8.7	1	Allele seq-mut has a higher chance of exon skipping than allele seq-wt.
rs564679424	Pro	CCG	0.11	CCA	0.28	0.17	2.5454545	G	-16.4	A	-14.8	1.6	Allele seq-wt has a higher chance of exon skipping than allele seq-mut.
rs773637284	Ala	GCG	0.11	GCT	0.27	0.16	2.4545455	C	-23.3	T	-21.5	1.8	Allele seq-mut has a higher chance of exon skipping than allele seq-wt.
rs529120158	Leu	CTC	0.2	CTG	0.4	0.2	2	C	-15.6	G	-19.2	-3.6	Allele seq-wt has a higher chance of exon skipping than allele seq-mut.

Supplementary Table 2B: Synonymous single nucleotide variants (SynSNVs) of *DNMT1* for which 2 folds or less changes in Codon usages were found

SNP Id	Coded amino acid	Codon 1	Frequency of usage	Codon 2	Frequency of usage	Change in usage (difference only)	Change in usage (fold change)	ALLELE 1 (A1)	DELTAG VALUE (kcal/mol)	ALLELE 2 (A2)	DELTAG VALUE (kcal/mol)	Energy change (A2 - A1)	Ex-skip predictions
rs1452414039	Leu	CTG	0.4	CTA	0.07	-0.33	0.175	G	-22.5	A	-20.2	2.3	Allele seq-wt has a higher chance of exon skipping than allele seq-mut.
rs769962566	Arg	CGC	0.18	CGT	0.08	-0.1	0.4444444	C	-18.5	T	-17.5	1	Allele seq-mut has a higher chance of exon skipping than allele seq-wt.
rs760037814	Thr	ACC	0.36	ACG	0.11	-0.25	0.3055556	C	-13.9	G	-18.8	-4.9	Allele seq-mut has a higher chance of exon skipping than allele seq-wt.
rs902335532	Arg	CGG	0.2	CGT	0.08	-0.12	0.4	G	-11.3	T	-15.7	-4.4	Allele seq-mut has a higher chance of exon skipping than allele seq-wt.
rs372868127	Pro	CCC	0.32	CCG	0.11	-0.21	0.34375	C	-13.9	G	-15.9	-2	Allele seq-wt has a higher chance of exon skipping than allele seq-mut.
rs1472939030	Leu	CTG	0.4	TTG	0.13	-0.27	0.325	C	-9.65	T	-11.5	-1.85	Allele seq-wt has a higher chance of exon skipping than allele seq-mut.
rs1452755314	Leu	CTG	0.4	CTA	0.07	-0.33	0.175	G	-9.8	A	-9.8	0	Allele seq-wt has a higher chance of exon skipping than allele seq-mut.
rs1371570796	Ile	ATC	0.47	ATA	0.17	-0.3	0.3617021	C	-5.95	A	-9.2	-3.25	Allele seq-wt has a higher chance of exon skipping than allele seq-mut.
rs1243858519	Arg	CGG	0.2	CGT	0.08	-0.12	0.4	G	-16.4	T	-13.6	2.8	Allele seq-mut has a higher chance of exon skipping than allele seq-wt.
rs1476199849	Gln	CAG	0.73	CAA	0.27	-0.46	0.369863	G	-19.8	A	-15.9	3.9	Allele seq-mut has a higher chance of exon skipping than allele seq-wt.
rs1436631668	Leu	CTG	0.4	TTG	0.13	-0.27	0.325	C	-13.7	T	-12.4	1.3	Allele seq-wt has a higher chance of exon skipping than allele seq-mut.
rs748976355	Leu	CTG	0.4	TTG	0.13	-0.27	0.325	C	-8.8	T	-9.3	-0.5	Allele seq-mut has a higher chance of exon skipping than allele seq-wt.
rs1405376714	Arg	CGG	0.2	CGT	0.08	-0.12	0.4	G	-10.9	T	-10.1	0.8	Allele seq-mut has a higher chance of exon skipping than allele seq-wt.
rs772090333	Arg	CGC	0.18	CGT	0.08	-0.1	0.4444444	C	-11	T	-14.5	-3.5	Allele seq-mut has a higher chance of exon skipping than allele seq-wt.
rs768760115	Leu	CTG	0.4	CTA	0.07	-0.33	0.175	G	-12.1	A	-8.9	3.2	Allele seq-wt has a higher chance of exon skipping than allele seq-mut.
rs1489027030	Val	GTG	0.46	GTT	0.18	-0.28	0.3913043	G	-7.7	T	-5.7	2	Allele seq-mut has a higher chance of exon skipping than allele seq-wt.
rs1263434012	Leu	CTG	0.4	TTG	0.13	-0.27	0.325	C	-9.9	T	-8.5	1.4	Allele seq-mut has a higher chance of exon skipping than allele seq-wt.
rs144675407	Pro	CCC	0.32	CCG	0.11	-0.21	0.34375	C	-136.29	G	-137.23	-0.94	Allele seq-mut has a higher chance of exon skipping than allele seq-wt.
rs1265124271	Gln	CAG	0.73	CAA	0.27	-0.46	0.369863	G	-20.9	A	-19.2	1.7	Both alleles have a comparable chance of exon skipping.
rs1459002984	Leu	CTG	0.4	CTA	0.07	-0.33	0.175	G	-18.5	A	-11.8	6.7	Allele seq-wt has a higher chance of exon skipping than allele seq-mut.

rs780059022	Val	GTG	0.46	GTT	0.18	-0.28	0.391304	G	-12.7	T	-11.4	1.3	Allele seq-mut has a higher chance of exonskipping than allele seq-wt.
rs150359172	Pro	CCC	0.32	CCG	0.11	-0.21	0.34375	C	-128.01	G	-133.84	-5.83	Allele seq-mut has a higher chance of exon skipping than allele seq-wt.
rs1183694434	Pro	CCA	0.28	CCG	0.11	-0.17	0.392857 1	A	-13.1	G	-17.3	-4.2	Allele seq-mut has a higher chance of exon skipping than allele seq-wt.
rs746458713	Leu	CTG	0.4	CTC	0.2	-0.2	0.5	G	-13.7	C	-16.4	-2.7	Allele seq-wt has a higher chance of exonskipping than allele seq-mut.
rs1175415967	Leu	CTG	0.4	TTG	0.13	-0.27	0.325	C	-11	T	-14	-3	Allele seq-mut has a higher chance of exon skipping than allele seq-wt.
rs1427221135	Pro	CCT	0.29	CCG	0.11	-0.18	0.379310 3	T	-9.2	G	-13.4	-4.2	Allele seq-mut has a higher chance of exon skipping than allele seq-wt.
rs1419289846	Ile	ATC	0.47	ATA	0.17	-0.3	0.361702 1	C	-9	A	-8.1	0.9	Both alleles have a comparable chance of exonskipping.
rs749306478	Leu	CTG	0.4	CTA	0.07	-0.33	0.175	G	-11.2	A	-5.04	6.16	Both alleles have a comparable chance of exon skipping.
rs763497211	Thr	ACA	0.28	ACG	0.11	-0.17	0.392857 1	A	-14.4	G	-15.4	-1	Allele seq-mut has a higher chance of exon skipping than allele seq-wt.
rs112405538	Thr	ACA	0.28	ACG	0.11	-0.17	0.392857 1	A	-131.55	G	-134.66	-3.11	Allele seq-wt has a higher chance of exon skipping than allele seq-mut.
rs915110490	Leu	CTG	0.4	TTG	0.13	-0.27	0.325	C	-7.4	T	-7.1	0.3	Allele seq-wt has a higher chance of exon skipping than allele seq-mut.
rs757830475	Pro	CCA	0.28	CCG	0.11	-0.17	0.392857 1	A	-1.04	G	-5.6	-4.56	Allele seq-mut has a higher chance of exon skipping than allele seq-wt.
rs532599778	Thr	ACC	0.36	ACG	0.11	-0.25	0.305555 6	C	-11.4	G	-13.8	-2.4	Allele seq-mut has a higher chance of exon skipping than allele seq-wt.
rs756623224	Gln	CAG	0.73	CAA	0.27	-0.46	0.369863	G	-21	A	-13.5	7.5	Allele seq-wt has a higher chance of exon skipping than allele seq-mut.
rs779773075	Leu	CTG	0.4	CTA	0.07	-0.33	0.175	G	-6.5	A	-6.3	0.2	Allele seq-wt has a higher chance of exon skipping than allele seq-mut.
rs721186	Thr	ACA	0.28	ACG	0.11	-0.17	0.392857 1	A	-356.63	G	-361.23	-4.6	Allele seq-mut has a higher chance of exon skipping than allele seq-wt.
rs765260449	Leu	CTG	0.4	TTG	0.13	-0.27	0.325	C	-7.14	T	-11.3	-4.16	Both alleles have a comparable chance of exon skipping.
rs2228613	Ile	ATC	0.47	ATA	0.17	-0.3	0.361702 1	C	-258.4	A	-260.6	-2.2	Both alleles have a comparable chance of exon skipping.
rs372722081	Leu	CTG	0.4	CTA	0.07	-0.33	0.175	G	-66.2	A	-61.18	5.02	Allele seq-mut has a higher chance of exon skipping than allele seq-wt.
rs1348242844	Leu	CTG	0.4	CTA	0.07	-0.33	0.175	G	-9.8	A	-9.5	0.3	Allele seq-wt has a higher chance of exon skipping than allele seq-mut.
rs2228611	Pro	CCA	0.28	CCG	0.11	-0.17	0.392857 1	A	-303.33	G	-307.15	-3.82	Both alleles have a comparable chance of exon skipping.
rs1331327607	Ala	GCA	0.23	GCG	0.11	-0.12	0.478260 9	A	-6.7	G	-6.9	-0.2	Allele seq-mut has a higher chance of exon skipping than allele seq-wt.
rs201487376	Ile	ATC	0.47	ATA	0.17	-0.3	0.361702 1	C	-13.1	A	-10.7	2.4	Allele seq-mut has a higher chance of exon skipping than allele seq-wt.

rs1269449523	Thr	ACC	0.36	ACG	0.11	-0.25	0.3055556	C	-15.5	G	-12.5	3	Allele seq-mut has a higher chance of exon skipping than allele seq-wt.
rs1483440897	Val	GTG	0.46	GTT	0.18	-0.28	0.3913043	G	-8.5	T	-5.1	3.4	Allele seq-mut has a higher chance of exon skipping than allele seq-wt.
rs777578400	Thr	ACA	0.28	ACG	0.11	-0.17	0.3928571	A	-11	G	-11.7	-0.7	Allele seq-mut has a higher chance of exon skipping than allele seq-wt.
rs1393946866	Pro	CCT	0.29	CCG	0.11	-0.18	0.3793103	T	-7	G	-9.7	-2.7	Allele seq-mut has a higher chance of exon skipping than allele seq-wt.
rs771543838	Ser	TCC	0.22	TCG	0.05	-0.17	0.2272727	C	-4.4	G	-6.1	-1.7	Allele seq-wt has a higher chance of exon skipping than allele seq-mut.
rs1233113560	Leu	CTG	0.4	TTG	0.13	-0.27	0.325	C	-5.2	T	-6.2	-1	Allele seq-mut has a higher chance of exon skipping than allele seq-wt.
rs770898949	Thr	ACA	0.28	ACG	0.11	-0.17	0.3928571	A	-15.08	G	-22.1	-7.02	Allele seq-mut has a higher chance of exon skipping than allele seq-wt.
rs577069147	Val	GTG	0.46	GTA	0.12	-0.34	0.2608696	G	-9	A	-6.6	2.4	Allele seq-wt has a higher chance of exonskipping than allele seq-mut.
rs760372702	Arg	CGC	0.18	CGT	0.08	-0.1	0.4444444	C	-12.8	T	-12.4	0.4	Both alleles have a comparable chance of exon skipping.
rs775786809	Arg	CGC	0.18	CGT	0.08	-0.1	0.4444444	C	-14.4	T	-13.7	0.7	Allele seq-wt has a higher chance of exon skipping than allele seq-mut.

Analysis of DNMT1 Gene Variants in Progression of Neural Tube Defects- an *insilico* to *invitro* approach

Susanta Sadhukhan[1†], Nirvika Paul[1†], Sudakshina Ghosh[2], Dinesh Munian[3], KausikGanguly[4], Krishnendu Ghosh[1], Mainak Sengupta[4], Madhusudan Das[1,*]

[1]Department of Zoology, University of Calcutta, 35 Ballygunge Circular Road, Kolkata-700019, India
[2]Department of Zoology, Vidyasagar College for Women, 39 Sankar Ghosh Lane, Kolkata-700006, India
[3]Department of Neonatology, Institute of Postgraduate Medical Education & Research, 244 Acharya Jagadish Chandra Bose Road, Kolkata, 700020, India
[4]Department of Genetics, University of Calcutta, 35 Ballygunge Circular Road, Kolkata-700019, India
[*]Corresponding author at the Dept. of Zoology, University of Calcutta: madhuzoo@yahoo.com/ mdzoo@caluniv.ac.in (mail id)
†Authors contributed equally

Supplementary Table 3: Results of *in silico* analyses of intronic single nucleotide variants (intronic SNVs) of *DNMT1*.Result table contains those intronic SNVs only for which maximal evidences were found in support of them being regulatory SNV (rSNV) i.e. being regulatory to expression of concerned loci as mentioned in the "F" and "G" columns.

SNV IDs (A)	Prioritized SNVs in LD with SNP in column A (B)	LD (r^2) value (C)	Co-ordinate of SNV [GRCh37] (D)	Regulome DB score (E)	Target loci found from rSNPBase (F)	Mode of regulation type(G)
rs201924117	None	_	chr19:10250535	2c	DNMT1	Proximal transcriptional regulation
					SNORD105	Distal transcriptional regulation
					PPAN	Distal transcriptional regulation
					SNORD105B	Distal transcriptional regulation
					EIF3G	Distal transcriptional regulation
					DNMT1	Distal transcriptional regulation
rs188026806	None	_	chr19:10246784	2b	DNMT1	Proximal transcriptional regulation
					P2RY11	Distal transcriptional regulation
					DNMT1	Distal transcriptional regulation
rs201753242	None	_	chr19:10246994	2b	DNMT1	Proximal transcriptional regulation
					EIF3G	Distal transcriptional regulation
					DNMT1	Distal transcriptional regulation
rs140884982	None	_	chr19:10248157	2b	DNMT1	Proximal transcriptional regulation
					EIF3G	Distal transcriptional regulation

rs144703096	None	_	chr19:10246575	2b	DNMT1	Proximal transcriptional regulation
					P2RY11	Distal transcriptional regulation
					DNMT1	Distal transcriptional regulation
rs79772680	None	_	chr19:10249962	2b	DNMT1	Proximal transcriptional regulation
					SNORD105	Distal transcriptional regulation
					PPAN	Distal transcriptional regulation
					SNORD105B	Distal transcriptional regulation
					EIF3G	Distal transcriptional regulation
					DNMT1	Distal transcriptional regulation
rs181852258	None	_	chr19:10244723	2b	PPAN	Distal transcriptional regulation
					SNORD105	Distal transcriptional regulation
					SNORD105B	Distal transcriptional regulation
					DNMT1	Distal transcriptional regulation
rs190841054	None	_	chr19:10250623	2b	DNMT1	Proximal transcriptional regulation
					SNORD105	Distal transcriptional regulation
					PPAN	Distal transcriptional regulation
					SNORD105B	Distal transcriptional regulation
					EIF3G	Distal transcriptional regulation
					PPAN-P2RY11	Distal transcriptional regulation
rs151121498	None	_	chr19:10250648	2b	DNMT1	Proximal transcriptional regulation
					SNORD105	Distal transcriptional regulation
					PPAN	Distal transcriptional regulation
					SNORD105B	Distal transcriptional regulation
					EIF3G	Distal transcriptional regulation
					PPAN-P2RY11	Distal transcriptional regulation
rs138664441	None	_	chr19:10248282	2b	DNMT1	Proximal transcriptional regulation
					EIF3G	Distal transcriptional regulation
					DNMT1	Distal transcriptional regulation
rs141232635	None	_	chr19:10248362	2b	DNMT1	Proximal transcriptional regulation
					EIF3G	Distal transcriptional regulation
					DNMT1	Distal transcriptional regulation

rs139988162	None	_	chr19:10246355	2c	DNMT1	Proximal transcriptional regulation
					PPAN	Distal transcriptional regulation
					SNORD105	Distal transcriptional regulation
					P2RY11	Distal transcriptional regulation
					DNMT1	Distal transcriptional regulation
rs115310241	None	_	chr19:10250132	2b	DNMT1	Proximal transcriptional regulation
					SNORD105	Distal transcriptional regulation
					PPAN	Distal transcriptional regulation
					SNORD105B	Distal transcriptional regulation
					EIF3G	Distal transcriptional regulation
					DNMT1	Distal transcriptional regulation
rs149568166	None	_	chr19:10248894	2b	DNMT1	Proximal transcriptional regulation
					SNORD105	Distal transcriptional regulation
					PPAN	Distal transcriptional regulation
					SNORD105B	Distal transcriptional regulation
					EIF3G	Distal transcriptional regulation
					DNMT1	Distal transcriptional regulation
rs114043198	None	_	chr19:10246207	2b	DNMT1	Proximal transcriptional regulation
					PPAN	Distal transcriptional regulation
					SNORD105	Distal transcriptional regulation
					P2RY11	Distal transcriptional regulation
					DNMT1	Distal transcriptional regulation
rs184657709	None	_	chr19:10248154	2b	DNMT1	Proximal transcriptional regulation
					EIF3G	Distal transcriptional regulation
rs148508558	None	_	chr19:10246597	2b	DNMT1	Proximal transcriptional regulation
					P2RY11	Distal transcriptional regulation
					DNMT1	Distal transcriptional regulation
rs148561427	None	_	chr19:10250134	2b	DNMT1	Proximal transcriptional regulation
					SNORD105	Distal transcriptional regulation
					PPAN	Distal transcriptional regulation
					SNORD105B	Distal transcriptional regulation
					EIF3G	Distal transcriptional regulation

					DNMT1	Distal transcriptional regulation
rs115805097	None	_	chr19:10248272	2b	DNMT1	Proximal transcriptional regulation
					EIF3G	Distal transcriptional regulation
					DNMT1	Distal transcriptional regulation
rs186169527	None	_	chr19:10250561	2b	DNMT1	Proximal transcriptional regulation
					SNORD105	Distal transcriptional regulation
					PPAN	Distal transcriptional regulation
					SNORD105B	Distal transcriptional regulation
					EIF3G	Distal transcriptional regulation
rs73922328	None	_	chr19:10246585	2b	DNMT1	Proximal transcriptional regulation
					P2RY11	Distal transcriptional regulation
					DNMT1	Distal transcriptional regulation
rs186675841	None	_	chr19:10249894	2b	DNMT1	Proximal transcriptional regulation
					SNORD105	Distal transcriptional regulation
					PPAN	Distal transcriptional regulation
					SNORD105B	Distal transcriptional regulation
					EIF3G	Distal transcriptional regulation
					DNMT1	Distal transcriptional regulation
rs75357496	None	_	chr19:10248889	2b	DNMT1	Proximal transcriptional
						regulation
					SNORD105	Distal transcriptional regulation
					PPAN	Distal transcriptional regulation
					SNORD105B	Distal transcriptional regulation
					EIF3G	Distal transcriptional regulation
					DNMT1	Distal transcriptional regulation
rs137949464	None	_	chr19:10247986	2b	DNMT1	Proximal transcriptional regulation
					EIF3G	Distal transcriptional regulation
rs184409608	None		chr19:10253433	2b	DNMT1	Proximal transcriptional regulation
					MIR4322	Distal transcriptional regulation
					EIF3G	Distal transcriptional regulation
					DNMT1	Distal transcriptional regulation
					MRPL4	Distal transcriptional regulation

rs2290683	rs190816746 rs79585069 rs78051786 rs3745269	1	chr19:10247975	2b	DNMT1	Proximal transcriptional regulation
					EIF3G	Distal transcriptional regulation
rs192339783	None	_	chr19:10252932	2b	DNMT1	Proximal transcriptional regulation
					MIR4322	Distal transcriptional regulation
					EIF3G	Distal transcriptional regulation
					DNMT1	Distal transcriptional regulation
					MRPL4	Distal transcriptional regulation
rs149321035	None	_	chr19:10246378	2b	DNMT1	Proximal transcriptional regulation
					PPAN	Distal transcriptional regulation
					SNORD105	Distal transcriptional regulation
					P2RY11	Distal transcriptional regulation
					DNMT1	Distal transcriptional regulation
rs190816746	rs2290683 rs79585069 rs78051786 rs3745269	1	chr19:10246315	2b	DNMT1	Proximal transcriptional regulation
					PPAN	Distal transcriptional regulation
					SNORD105	Distal transcriptional regulation
					P2RY11	Distal transcriptional regulation
					DNMT1	Distal transcriptional regulation
rs200887793	None	_	chr19:10252942	2b	DNMT1	Proximal transcriptional regulation
					MIR4322	Distal transcriptional regulation
					EIF3G	Distal transcriptional regulation
					DNMT1	Distal transcriptional regulation
					MRPL4	Distal transcriptional regulation
rs79585069	rs190816746 rs2290683 rs78051786 rs3745269	1	chr19:10247973	2b	DNMT1	Proximal transcriptional regulation
					EIF3G	Distal transcriptional regulation
rs115024008	None	_	chr19:10255369	2b	DNMT1	Proximal transcriptional regulation
					DNMT1	Distal transcriptional regulation
					DNMT1	Proximal transcriptional regulation

rs189195828	None	_	chr19:10248365	2b	EIF3G	Distal transcriptional regulation
					DNMT1	Distal transcriptional regulation
rs10854076	rs66653469	1	chr19:10248270	2b	DNMT1	Proximal transcriptional regulation
					EIF3G	Distal transcriptional regulation
					DNMT1	Distal transcriptional regulation
rs28679320	None	_	chr19:10247701	2b	DNMT1	Proximal transcriptional regulation
					EIF3G	Distal transcriptional regulation
					DNMT1	Distal transcriptional regulation
rs144996150	None	_	chr19:10254770	2b	DNMT1	Proximal transcriptional regulation
					MIR4322	Distal transcriptional regulation
					DNMT1	Distal transcriptional regulation
rs117579193	None	_	chr19:10247718	2b	DNMT1	Proximal transcriptional regulation
					EIF3G	Distal transcriptional regulation
					DNMT1	Distal transcriptional regulation
rs186777667	None	_	chr19:10261416	2b	DNMT1	Distal transcriptional regulation
rs182176004	None	_	chr19:10269427	2b	DNMT1	Proximal transcriptional regulation
					DNMT1	Distal transcriptional regulation
					OLFM2	Distal transcriptional regulation
rs74671219	None	_	chr19:10265222	2b	DNMT1	Proximal transcriptional regulation
					DNMT1	Distal transcriptional
						regulation
rs4804491	None	_	chr19:10270291	2b	DNMT1	Proximal transcriptional regulation
					DNMT1	Distal transcriptional regulation
					OLFM2	Distal transcriptional regulation
rs116892102	None	_	chr19:10257563	2c	DNMT1	Proximal transcriptional regulation
					DNMT1	Distal transcriptional regulation
rs189214882	None	_	chr19:10270505	2b	DNMT1	Proximal transcriptional regulation
					DNMT1	Distal transcriptional regulation
					OLFM2	Distal transcriptional regulation
rs200288095	None	_	chr19:10265543	2b	DNMT1	Proximal transcriptional regulation
					DNMT1	Distal transcriptional regulation
					DNMT1	Proximal transcriptional regulation

rs6511677	None	_	chr19:10277799	1f	DNMT1	Distal transcriptional regulation
rs8101866	rs7258766	1	chr19:10275660	1f	DNMT1	Proximal transcriptional regulation
					SNORD105B	Distal transcriptional regulation
					PPAN	Distal transcriptional regulation
rs190171531	None	_	chr19:10284457	2b	DNMT1	Distal transcriptional regulation
rs201204934	None	_	chr19:10277411	2b	DNMT1	Distal transcriptional regulation
rs56162188	None	_	chr19:10284452	2b	DNMT1	Distal transcriptional regulation
rs192464543	None	_	chr19:10277106	2b	DNMT1	Distal transcriptional regulation
rs7258766	rs8101866	1	chr19:10283281	2b	DNMT1	Distal transcriptional regulation
rs190972098	None	_	chr19:10274112	2b	DNMT1	Distal transcriptional regulation
rs185732622	None	_	chr19:10276744	2b	DNMT1	Proximal transcriptional regulation
					SNORD105B	Distal transcriptional regulation
					PPAN	Distal transcriptional regulation
rs151298589	None	_	chr19:10273538	2c	DNMT1	Distal transcriptional regulation
rs190441512	None	_	chr19:10302076	2a	DNMT1	Distal transcriptional regulation
					ICAM4	Distal transcriptional regulation
					P2RY11	Distal transcriptional regulation
					EIF3G	Distal transcriptional regulation
					MIR4322	Distal transcriptional regulation
					ENSG00000267534	Distal transcriptional regulation
rs192458451	None	_	chr19:10295759	2b	DNMT1	Proximal transcriptional regulation
					DNMT1	Distal transcriptional regulation
rs78051786	rs190816746 rs2290683 rs79585069 rs3745269	1	chr19:10298311	2b	DNMT1	Proximal transcriptional regulation
					DNMT1	Distal transcriptional regulation
					MIR4322	Distal transcriptional regulation
					ENSG00000267534	Distal transcriptional regulation
rs190175638	None	_	chr19:10303183	2b	MRPL4	Distal transcriptional regulation
					P2RY11	Distal transcriptional regulation
					EIF3G	Distal transcriptional regulation
					MIR4322	Distal transcriptional regulation
					ENSG00000267534	Distal transcriptional regulation
					DNMT1	Distal transcriptional regulation

rs149702090	None	_	chr19:10295312	2c	DNMT1	Proximal transcriptional regulation
					DNMT1	Distal transcriptional regulation
rs150488951	None	_	chr19:10302244	2c	DNMT1	Distal transcriptional regulation
					ICAM4	Distal transcriptional regulation
					P2RY11	Distal transcriptional regulation
					EIF3G	Distal transcriptional regulation
					MIR4322	Distal transcriptional regulation
					ENSG00000267534	Distal transcriptional regulation
rs66653469	rs10854076	1	chr19:10293627	2c	DNMT1	Proximal transcriptional regulation
					DNMT1	Distal transcriptional regulation
rs74433001	None	_	chr19:10304723	2b	WDR74	Distal transcriptional regulation
					MRPL4	Distal transcriptional regulation
					MIR4322	Distal transcriptional regulation
					ENSG00000267534	Distal transcriptional regulation
					DNMT1	Distal transcriptional regulation
					TYK2	Distal transcriptional regulation
					P2RY11	Distal transcriptional regulation
					EIF3G	Distal transcriptional regulation
					S1PR2	Distal transcriptional
						regulation
					RAVER1	Distal transcriptional regulation
					ICAM3	Distal transcriptional regulation
					PPAN-P2RY11	Distal transcriptional regulation
					PPAN	Distal transcriptional regulation
					SNORD105	Distal transcriptional regulation
					MESDC2	Distal transcriptional regulation
					DNMT1	Proximal transcriptional regulation
					TBC1D9B	Distal transcriptional regulation
					WDR74	Distal transcriptional regulation
					IQGAP1	Distal transcriptional regulation
					ALDOA	Distal transcriptional regulation
					POLR2A	Distal transcriptional regulation

rs3745269	rs190816746 rs2290683 rs79585069 rs78051786	1	chr19:10305409	2b	SEH1L	Distal transcriptional regulation
					VPRBP	Distal transcriptional regulation
					SETD5-AS1	Distal transcriptional regulation
					UBTD2	Distal transcriptional regulation
					FNIP1	Distal transcriptional regulation
					CENPK	Distal transcriptional regulation
					PPWD1	Distal transcriptional regulation
					ARRDC2	Distal transcriptional regulation
					GRAMD1A	Distal transcriptional regulation
					MIR4322	Distal transcriptional regulation
					ENSG00000267534	Distal transcriptional regulation
					DNMT1	Distal transcriptional regulation
					TYK2	Distal transcriptional regulation
					P2RY11	Distal transcriptional regulation
					EIF3G	Distal transcriptional regulation
					MRPL4	Distal transcriptional regulation
					S1PR2	Distal transcriptional regulation
					RAVER1	Distal transcriptional regulation
					ICAM3	Distal transcriptional regulation
					CYP2R1	Distal transcriptional regulation
					ARID2	Distal transcriptional regulation
					DLEU2	Distal transcriptional regulation
					ENSG00000263053	Distal transcriptional regulation
					DDX17	Distal transcriptional regulation
					YEATS2	Distal transcriptional regulation
					SETD9	Distal transcriptional regulation
					ENSG00000236753	Distal transcriptional regulation
					GINS4	Distal transcriptional regulation
					DNMT1	Proximal transcriptional regulation
					TBC1D9B	Distal transcriptional regulation
					WDR74	Distal transcriptional regulation
					IQGAP1	Distal transcriptional regulation

rs74930654	None	_	chr19:10305282	2b	ALDOA	Distal transcriptional regulation
					POLR2A	Distal transcriptional regulation
					SEH1L	Distal transcriptional regulation
					VPRBP	Distal transcriptional regulation
					UBTD2	Distal transcriptional regulation
					FNIP1	Distal transcriptional regulation
					CENPK	Distal transcriptional regulation
					PPWD1	Distal transcriptional regulation
					ARRDC2	Distal transcriptional regulation
					MIR4322	Distal transcriptional regulation
					ENSG00000267534	Distal transcriptional regulation
					DNMT1	Distal transcriptional regulation
					TYK2	Distal transcriptional regulation
					P2RY11	Distal transcriptional regulation
					EIF3G	Distal transcriptional regulation
					MRPL4	Distal transcriptional regulation
					S1PR2	Distal transcriptional regulation
					RAVER1	Distal transcriptional
						regulation
					ICAM3	Distal transcriptional regulation
					DLEU2	Distal transcriptional regulation
					ENSG00000263053	Distal transcriptional regulation
					DDX17	Distal transcriptional regulation
					YEATS2	Distal transcriptional regulation
					SETD9	Distal transcriptional regulation
					ENSG00000236753	Distal transcriptional regulation
					MRPL4	Distal transcriptional regulation
					MIR4322	Distal transcriptional regulation
					ENSG00000267534	Distal transcriptional regulation
					DNMT1	Distal transcriptional regulation
					TYK2	Distal transcriptional regulation
rs117789533	None	_	chr19:10304364	2b	P2RY11	Distal transcriptional regulation

					EIF3G	Distal transcriptional regulation
					S1PR2	Distal transcriptional regulation
					PPAN-P2RY11	Distal transcriptional regulation
					PPAN	Distal transcriptional regulation
					SNORD105	Distal transcriptional regulation

Analysis of DNMT1 Gene Variants in Progression of Neural Tube Defects- an *insilico* to *invitro* approach

Susanta Sadhukhan[1†], Nirvika Paul[1†], Sudakshina Ghosh[2], Dinesh Munian[3], Kausik Ganguly[4], Krishnendu Ghosh[1], Mainak Sengupta[4],Madhusudan Das[1,*]

[1]Department of Zoology, University of Calcutta, 35 Ballygunge Circular Road, Kolkata-700019, India
[2]Department of Zoology, Vidyasagar College for Women, 39 Sankar Ghosh Lane, Kolkata-700006, India
[3]Department of Neonatology, Institute of Postgraduate Medical Education & Research, 244 Acharya Jagadish Chandra Bose Road, Kolkata, 700020, India
[4]Department of Genetics, University of Calcutta, 35 Ballygunge Circular Road, Kolkata-700019, India

[*]Corresponding author at the Dept. of Zoology, University of Calcutta: madhuzoo@yahoo.com/mdzoo@caluniv.ac.in (mail id)
†Authors contributed equally

Supplementary Table 4: Results of *in silico* analyses of intergenic single nucleotide variants (intergenic SNVs) of *DNMT1*.Results of prioritization of intergenic SNVs of *DNMT1* that belong to positively and negatively correlated DNase Hypersensitive Sites (DHS). The table contains those intergenic SNVs only for which maximal evidences were found in support of them being regulatory SNV (rSNV) i.e. being regulatory to expression of concerned loci as mentioned in the "H" and "I" columns.

Supplementary Table 4A: Results of prioritization of SNVs of *DNMT1* that belong to positively correlated DNase Hypersensitive Sites (DHS)

SNV IDs (A)	Prioritized SNVs in LD with SNP in column A (B)	LD (r^2) value (C)	Co-ordinate of SNV [GRCh37] (D)	Co-ordinate of DHS (E)	Correlation of DHS with expression of the gene in column A (F)	Regulome DB score (G)	Target loci found from rSNPBase (H)	Mode of regulation type (I)
rs3745269	None	_	chr19:10305408	chr19: 10305300-10305450	Positive	2b	DNMT1	Proximal transcriptional regulation
							TBC1D9B	Distal transcriptional regulation
							WDR74	Distal transcriptional regulation
							IQGAP1	Distal transcriptional regulation
							ALDOA	Distal transcriptional regulation
							POLR2A	Distal transcriptional regulation

							SEH1L	Distal transcriptional regulation
							VPRBP	Distal transcriptional regulation
							SETD5-AS1	Distal transcriptional regulation
							UBTD2	Distal transcriptional regulation
							FNIP1	Distal transcriptional regulation
							CENPK	Distal transcriptional regulation
							PPWD1	Distal transcriptional regulation
							ARRDC2	Distal transcriptional regulation
							GRAMD1A	Distal transcriptional regulation
							MIR4322	Distal transcriptional regulation
							ENSG00000267534	Distal transcriptional regulation
							DNMT1	Distal transcriptional regulation
							TYK2	Distal transcriptional regulation
							P2RY11	Distal transcriptional regulation
							EIF3G	Distal transcriptional regulation
							MRPL4	Distal transcriptional regulation
							S1PR2	Distal transcriptional regulation
							RAVER1	Distal transcriptional regulation
							ICAM3	Distal transcriptional regulation
							CYP2R1	Distal transcriptional regulation
							ARID2	Distal transcriptional regulation
							DLEU2	Distal transcriptional regulation
							ENSG00000263053	Distal transcriptional regulation
							DDX17	Distal transcriptional regulation
							YEATS2	Distal transcriptional regulation
							SETD9	Distal transcriptional regulation
							ENSG00000236753	Distal transcriptional regulation
							GINS4	Distal transcriptional regulation
rs144353420	None	_	chr19:10223997	chr19: 10223900-10224050	Positive	2a	OLFM2	Distal transcriptional regulation
							C19orf66	Distal transcriptional regulation
							ENSG00000267387	Distal transcriptional regulation

							DNMT1	Distal transcriptional regulation
							MRPL4	Distal transcriptional regulation
							ICAM1	Distal transcriptional regulation
							ANGPTL6	Distal transcriptional regulation
							DCPS	Distal transcriptional regulation
rs186812573	None	_	chr19:10224002	chr19: 10223900-10224050	Positive	2a	OLFM2	Distal transcriptional regulation
							C19orf66	Distal transcriptional regulation
							ENSG00000267387	Distal transcriptional regulation
							DNMT1	Distal transcriptional regulation
							MRPL4	Distal transcriptional regulation
							ICAM1	Distal transcriptional regulation
							ANGPTL6	Distal transcriptional regulation
							DCPS	Distal transcriptional regulation
rs190521607	None	_	chr19:10224042	chr19: 10223900-10224050	Positive	2b	OLFM2	Distal transcriptional regulation
							C19orf66	Distal transcriptional regulation
							ENSG00000267387	Distal transcriptional regulation
							DNMT1	Distal transcriptional regulation
							MRPL4	Distal transcriptional regulation
							ICAM1	Distal transcriptional regulation
							ANGPTL6	Distal transcriptional regulation
							DCPS	Distal transcriptional regulation
rs200573354	None	_	chr19:10363116	chr19: 10363060-10363210	Positive	2b	MRPL4	Proximal transcriptional regulation
							SNORD60	Distal transcriptional regulation
							ENSG00000260260	Distal transcriptional regulation
							ANGPTL6	Distal transcriptional regulation
							EIF3G	Distal transcriptional regulation
							DNMT1	Distal transcriptional regulation
							MIR4322	Distal transcriptional regulation
							ENSG00000267534	Distal transcriptional regulation
							ICAM5	Distal transcriptional regulation
							ICAM1	Distal transcriptional regulation

							TYK2	Distal transcriptional regulation
							CDKN2D	Distal transcriptional regulation
							P2RY11	Distal transcriptional regulation
							CDC37	Distal transcriptional regulation
							PDE4A	Distal transcriptional regulation
							ICAM4	Distal transcriptional regulation
							MIR1181	Distal transcriptional regulation
							PPAN	Distal transcriptional regulation
							SNORD105	Distal transcriptional regulation
							SNORD105B	Distal transcriptional regulation
							YES1	Distal transcriptional regulation
							ZNF516	Distal transcriptional regulation
							ENSG00000220032	Distal transcriptional regulation
							SERP1	Distal transcriptional regulation
							ENSG00000198843	Distal transcriptional regulation
							CKS2	Distal transcriptional regulation
							ECH1	Distal transcriptional regulation
							ENSG00000266978	RNA binding protein mediated regulation
							MRPL4	RNA binding protein mediated regulation
rs200851671	None	_	chr19:10363118	chr19: 10363060-10363210	Positive	2b	MRPL4	Proximal transcriptional regulation
							SNORD60	Distal transcriptional regulation
							ENSG00000260260	Distal transcriptional regulation
							ANGPTL6	Distal transcriptional regulation
							EIF3G	Distal transcriptional regulation
							DNMT1	Distal transcriptional regulation
							MIR4322	Distal transcriptional regulation
							ENSG00000267534	Distal transcriptional regulation
							ICAM5	Distal transcriptional regulation
							ICAM1	Distal transcriptional regulation
							TYK2	Distal transcriptional regulation
							CDKN2D	Distal transcriptional regulation

							P2RY11	Distal transcriptional regulation
							CDC37	Distal transcriptional regulation
							PDE4A	Distal transcriptional regulation
							ICAM4	Distal transcriptional regulation
							MIR1181	Distal transcriptional regulation
							PPAN	Distal transcriptional regulation
							SNORD105	Distal transcriptional regulation
							SNORD105B	Distal transcriptional regulation
							YES1	Distal transcriptional regulation
							ZNF516	Distal transcriptional regulation
							ENSG00000220032	Distal transcriptional regulation
							SERP1	Distal transcriptional regulation
							ENSG00000198843	Distal transcriptional regulation
							CKS2	Distal transcriptional regulation
							ECH1	Distal transcriptional regulation
							ENSG00000266978	RNA binding protein mediated regulation
							MRPL4	RNA binding protein mediated regulation
rs199583987	None	_	chr19:10363122	chr19: 10363060-10363210	Positive	2b	MRPL4	Proximal transcriptional regulation
							SNORD60	Distal transcriptional regulation
							ENSG00000260260	Distal transcriptional regulation
							ANGPTL6	Distal transcriptional regulation
							EIF3G	Distal transcriptional regulation
							DNMT1	Distal transcriptional regulation
							MIR4322	Distal transcriptional regulation
							ENSG00000267534	Distal transcriptional regulation
							ICAM5	Distal transcriptional regulation
							ICAM1	Distal transcriptional regulation
							TYK2	Distal transcriptional regulation
							CDKN2D	Distal transcriptional regulation
							P2RY11	Distal transcriptional regulation
							CDC37	Distal transcriptional regulation

							PDE4A	Distal transcriptional regulation
							ICAM4	Distal transcriptional regulation
							MIR1181	Distal transcriptional regulation
							PPAN	Distal transcriptional regulation
							SNORD105	Distal transcriptional regulation
							SNORD105B	Distal transcriptional regulation
							YES1	Distal transcriptional regulation
							ZNF516	Distal transcriptional regulation
							ENSG00000220032	Distal transcriptional regulation
							SERP1	Distal transcriptional regulation
							ENSG00000198843	Distal transcriptional regulation
							CKS2	Distal transcriptional regulation
							ECH1	Distal transcriptional regulation
							ENSG00000266978	RNA binding protein mediated regulation
							MRPL4	RNA binding protein mediated regulation
rs143729133	None	_	chr19:10363133	chr19: 10363060-10363210	Positive	2b	MRPL4	Proximal transcriptional regulation
							SNORD60	Distal transcriptional regulation
							ENSG00000260260	Distal transcriptional regulation
							ANGPTL6	Distal transcriptional regulation
							EIF3G	Distal transcriptional regulation
							DNMT1	Distal transcriptional regulation
							MIR4322	Distal transcriptional regulation
							ENSG00000267534	Distal transcriptional regulation
							ICAM5	Distal transcriptional regulation
							ICAM1	Distal transcriptional regulation
							TYK2	Distal transcriptional regulation
							CDKN2D	Distal transcriptional regulation
							P2RY11	Distal transcriptional regulation
							CDC37	Distal transcriptional regulation
							PDE4A	Distal transcriptional regulation
							ICAM4	Distal transcriptional regulation

							MIR1181	Distal transcriptional regulation
							PPAN	Distal transcriptional regulation
							SNORD105	Distal transcriptional regulation
							SNORD105B	Distal transcriptional regulation
							YES1	Distal transcriptional regulation
							ZNF516	Distal transcriptional regulation
							ENSG00000220032	Distal transcriptional regulation
							SERP1	Distal transcriptional regulation
							ENSG00000198843	Distal transcriptional regulation
							CKS2	Distal transcriptional regulation
							ECH1	Distal transcriptional regulation
							ENSG00000266978	RNA binding protein mediated regulation
							MRPL4	RNA binding protein mediated regulation
rs12460842	None	_	chr19:10222194	chr19: 10222100-10222250	Positive	2b	P2RY11	Proximal transcriptional regulation
							MRPL4	Distal transcriptional regulation
							ANGPTL6	Distal transcriptional regulation
rs73011214	None	_	chr19:10222204	chr19: 10222100-10222250	Positive	2b	P2RY11	Proximal transcriptional regulation
							MRPL4	Distal transcriptional regulation
							ANGPTL6	Distal transcriptional regulation
rs118169733	None	_	chr19:10223638	chr19: 10223580-10223730	Positive	2b	OLFM2	Distal transcriptional regulation
							DNMT1	Distal transcriptional regulation
							MRPL4	Distal transcriptional regulation
							ANGPTL6	Distal transcriptional regulation
							CSRNP2	Distal transcriptional regulation
rs77442942	None	_	chr19:10215450	chr19: 10215440-10215590	Positive	2b	PPAN	Proximal transcriptional regulation
							ANGPTL6	Proximal transcriptional regulation
							SNORD105	Proximal transcriptional regulation
							SNORD105B	Proximal transcriptional regulation
							PPAN-P2RY11	Proximal transcriptional regulation
							RDH8	Distal transcriptional regulation
							ENSG00000267387	Distal transcriptional regulation

							C19orf66	Distal transcriptional regulation
rs184068397	None	_	chr19:10215583	chr19: 10215440-10215590	Positive	2b	ANGPTL6	Distal transcriptional regulation
							PPAN	Proximal transcriptional regulation
							ANGPTL6	Proximal transcriptional regulation
							SNORD105	Proximal transcriptional regulation
							SNORD105B	Proximal transcriptional regulation
							PPAN-P2RY11	Proximal transcriptional regulation
							RDH8	Distal transcriptional regulation
							ENSG00000267387	Distal transcriptional regulation
							C19orf66	Distal transcriptional regulation
							ANGPTL6	Distal transcriptional regulation
rs755258	None	_	chr19:10215585	chr19: 10215440-10215590	Positive	2b	PPAN	Proximal transcriptional regulation
							ANGPTL6	Proximal transcriptional regulation
							SNORD105	Proximal transcriptional regulation
							SNORD105B	Proximal transcriptional regulation
							PPAN-P2RY11	Proximal transcriptional regulation
							RDH8	Distal transcriptional regulation
							ENSG00000267387	Distal transcriptional regulation
							C19orf66	Distal transcriptional regulation
							ANGPTL6	Distal transcriptional regulation
rs138165069	None	_	chr19:10223181	chr19: 10223100-10223250	Positive	2b	OLFM2	Distal transcriptional regulation
							DNMT1	Distal transcriptional regulation
							MRPL4	Distal transcriptional regulation
							ANGPTL6	Distal transcriptional regulation
							CSRNP2	Distal transcriptional regulation
							HIST1H3H	Distal transcriptional regulation
rs150374591	None	_	chr19:10156249	chr19: 10156160-10156310	Positive	2a	C3P1	RNA binding protein mediated regulation
rs111375517	None	_	chr19:10230328	chr19: 10230240-10230390	Positive	2c	EIF3G	Proximal transcriptional regulation
							C19orf66	Distal transcriptional regulation
							ENSG00000267387	Distal transcriptional regulation

							MRPL4	Distal transcriptional regulation
							ICAM5	Distal transcriptional regulation
							CDC37	Distal transcriptional regulation
							PDE4A	Distal transcriptional regulation
							OLFM2	Distal transcriptional regulation
							DNMT1	Distal transcriptional regulation
							ANGPTL6	Distal transcriptional regulation
							MIR4322	Distal transcriptional regulation
							ENSG00000267534	Distal transcriptional regulation
							SLC1A5	Distal transcriptional regulation
							MIR1181	Distal transcriptional regulation
							EIF3G	RNA binding protein mediated regulation
rs200842732	None	_	chr19:10229874	chr19: 10229865-10230015	Positive	2b	EIF3G	Proximal transcriptional regulation
							C19orf66	Distal transcriptional regulation
							ENSG00000267387	Distal transcriptional regulation
							MRPL4	Distal transcriptional regulation
							ICAM5	Distal transcriptional regulation
							CDC37	Distal transcriptional regulation
							PDE4A	Distal transcriptional regulation
							DNMT1	Distal transcriptional regulation
							ANGPTL6	Distal transcriptional regulation
							MIR4322	Distal transcriptional regulation
							ENSG00000267534	Distal transcriptional regulation
							EIF3G	RNA binding protein mediated regulation
rs115986278	None	_	chr19:10224936	chr19: 10224900-10225050	Positive	2a	PPAN-P2RY11	Proximal transcriptional regulation
							OLFM2	Distal transcriptional regulation
							C19orf66	Distal transcriptional regulation
							ENSG00000267387	Distal transcriptional regulation
							DNMT1	Distal transcriptional regulation
							MRPL4	Distal transcriptional regulation
							S1PR2	Distal transcriptional regulation

							ENSG00000267607	Distal transcriptional regulation
							ICAM5	Distal transcriptional regulation
							L2HGDH	Distal transcriptional regulation
							ATP5S	Distal transcriptional regulation
							ICAM1	Distal transcriptional regulation
							RNU5E-1	Distal transcriptional regulation
							RNU5E-4P	Distal transcriptional regulation
							TMED3	Distal transcriptional regulation
							ANGPTL6	Distal transcriptional regulation
rs114550611	None	_	chr19:10330428	chr19: 10330340-10330490	Positive	2c	EIF3G	Distal transcriptional regulation
							MIR4322	Distal transcriptional regulation
							ENSG00000267534	Distal transcriptional regulation
							DNMT1	Distal transcriptional regulation
							S1PR2	Distal transcriptional regulation
rs200231187	None	_	chr19:10225252	chr19: 10225220-10225370	Positive	2a	PPAN-P2RY11	Proximal transcriptional regulation
							OLFM2	Distal transcriptional regulation
							C19orf66	Distal transcriptional regulation
							ENSG00000267387	Distal transcriptional regulation
							DNMT1	Distal transcriptional regulation
							MRPL4	Distal transcriptional regulation
							S1PR2	Distal transcriptional regulation
							ENSG00000267607	Distal transcriptional regulation
							ICAM5	Distal transcriptional regulation
							L2HGDH	Distal transcriptional regulation
							ATP5S	Distal transcriptional regulation
							ICAM1	Distal transcriptional regulation
							RNU5E-1	Distal transcriptional regulation
							RNU5E-4P	Distal transcriptional regulation
							TMED3	Distal transcriptional regulation
							ANGPTL6	Distal transcriptional regulation
rs138241238	None	_	chr19:10225259	chr19:	Positive	2a	PPAN-P2RY11	Proximal transcriptional regulation

				10225220-10225370			OLFM2	Distal transcriptional regulation
							C19orf66	Distal transcriptional regulation
							ENSG00000267387	Distal transcriptional regulation
							DNMT1	Distal transcriptional regulation
							MRPL4	Distal transcriptional regulation
							S1PR2	Distal transcriptional regulation
							ENSG00000267607	Distal transcriptional regulation
							ICAM5	Distal transcriptional regulation
							L2HGDH	Distal transcriptional regulation
							ATP5S	Distal transcriptional regulation
							ICAM1	Distal transcriptional regulation
							RNU5E-1	Distal transcriptional regulation
							RNU5E-4P	Distal transcriptional regulation
							TMED3	Distal transcriptional regulation
							ANGPTL6	Distal transcriptional regulation
rs141546180	None	_	chr19:10225302	chr19: 10225220-10225370	Positive	2b	PPAN-P2RY11	Proximal transcriptional regulation
							OLFM2	Distal transcriptional regulation
							C19orf66	Distal transcriptional regulation
							ENSG00000267387	Distal transcriptional regulation
							DNMT1	Distal transcriptional regulation
							MRPL4	Distal transcriptional regulation
							S1PR2	Distal transcriptional regulation
							ENSG00000267607	Distal transcriptional regulation
							ICAM5	Distal transcriptional regulation
							L2HGDH	Distal transcriptional regulation
							ATP5S	Distal transcriptional regulation
							ICAM1	Distal transcriptional regulation
							RNU5E-1	Distal transcriptional regulation
							RNU5E-4P	Distal transcriptional regulation
							TMED3	Distal transcriptional regulation
							ANGPTL6	Distal transcriptional regulation

rs62638736	None	_	chr19:10225307	chr19: 10225220-10225370	Positive	2b	PPAN-P2RY11	Proximal transcriptional regulation
							OLFM2	Distal transcriptional regulation
							C19orf66	Distal transcriptional regulation
							ENSG00000267387	Distal transcriptional regulation
							DNMT1	Distal transcriptional regulation
							MRPL4	Distal transcriptional regulation
							S1PR2	Distal transcriptional regulation
							ENSG00000267607	Distal transcriptional regulation
							ICAM5	Distal transcriptional regulation
							L2HGDH	Distal transcriptional regulation
							ATP5S	Distal transcriptional regulation
							ICAM1	Distal transcriptional regulation
							RNU5E-1	Distal transcriptional regulation
							RNU5E-4P	Distal transcriptional regulation
							TMED3	Distal transcriptional regulation
							ANGPTL6	Distal transcriptional regulation
rs111544544	None	_	chr19:10197371	chr19: 10197305-10197455	Positive	2a	C19orf66	Proximal transcriptional regulation
							C19orf66	Distal transcriptional regulation
							ENSG00000267607	Distal transcriptional regulation
							ICAM5	Distal transcriptional regulation
							ANGPTL6	Distal transcriptional regulation
							ENSG00000267387	Distal transcriptional regulation
							C19orf66	RNA binding protein mediated regulation
rs77076061	None	_	chr19:10197430	chr19: 10197305-10197455	Positive	2b	C19orf66	Proximal transcriptional regulation
							C19orf66	Distal transcriptional regulation
							ENSG00000267607	Distal transcriptional regulation
							ICAM5	Distal transcriptional regulation
							ANGPTL6	Distal transcriptional regulation
							ENSG00000267387	Distal transcriptional regulation
							C19orf66	RNA binding protein mediated regulation
rs36213469	None	_	chr19:10381082	chr19:	Positive	2a	ICAM1	Proximal transcriptional regulation

				10381080-10381230			MRPL4	Distal transcriptional regulation
							CDC37	Distal transcriptional regulation
							TYK2	Distal transcriptional regulation
							ENSG00000167807	Distal transcriptional regulation
							FDX1L	Distal transcriptional regulation
							CDKN2D	Distal transcriptional regulation
							RAVER1	Distal transcriptional regulation
							ICAM3	Distal transcriptional regulation
							MIR1181	Distal transcriptional regulation
							ENSG00000267105	Distal transcriptional regulation
							ICAM1	Distal transcriptional regulation
							ENSG00000266978	Distal transcriptional regulation
							ENSG00000267607	Distal transcriptional regulation
							ICAM5	Distal transcriptional regulation
							PDCL3	Distal transcriptional regulation
rs3760755	None	_	chr19:10216186	chr19: 10216140-10216290	Positive	2b	PPAN	Proximal transcriptional regulation
							ANGPTL6	Proximal transcriptional regulation
							SNORD105	Proximal transcriptional regulation
							SNORD105B	Proximal transcriptional regulation
							PPAN-P2RY11	Proximal transcriptional regulation
							RDH8	Distal transcriptional regulation
							ENSG00000267387	Distal transcriptional regulation
							C19orf66	Distal transcriptional regulation
							ANGPTL6	Distal transcriptional regulation
							ICAM5	Distal transcriptional regulation
							ENSG00000212195	Distal transcriptional regulation
							EEF2	Distal transcriptional regulation
rs5030391	None	_	chr19:10382636	chr19: 10382560-10382710	Positive	2b	MRPL4	Distal transcriptional regulation
							CDC37	Distal transcriptional regulation
							TYK2	Distal transcriptional regulation
							RAVER1	Distal transcriptional regulation

							ICAM3	Distal transcriptional regulation
							MIR1181	Distal transcriptional regulation
							ENSG00000267105	Distal transcriptional regulation
							ENSG00000167807	Distal transcriptional regulation
							FDX1L	Distal transcriptional regulation
							ENSG00000267607	Distal transcriptional regulation
							ICAM5	Distal transcriptional regulation
							ENSG00000266978	Distal transcriptional regulation
							ENSG00000266978	RNA binding protein mediated regulation
							ICAM1	RNA binding protein mediated regulation

Supplementary Table 4B: Results of prioritization of SNVs of *DNMT1* that belong to negatively correlated DNase Hypersensitive Sites (DHS)

SNV IDs (A)	Prioritized SNVs in LD with SNP in column A (B)	LD (r^2) value (C)	Co-ordinate of SNV [GRCh37] (E)	Co-ordinate of DHS (F)	Correlation of DHS with expression of the gene in column A (G)	Regulome DB score (I)	Target loci found from rSNPBase (J)	Mode of regulation type (K)
rs144570250	None	_	chr19:10242475	chr19: 10242360-10242510	Negative	2a	DNMT1	Distal transcriptional regulation
							PPAN-P2RY11	Distal transcriptional regulation
							PPAN	Distal transcriptional regulation
							SNORD105	Distal transcriptional regulation
rs139592077	None	_	chr19:10231671	chr19:	Negative	2b	EIF3G	Proximal transcriptional regulation

				10231620-10231770			DNMT1	Distal transcriptional regulation
							OLFM2	Distal transcriptional regulation
							ANGPTL6	Distal transcriptional regulation
							MIR4322	Distal transcriptional regulation
							ENSG00000267534	Distal transcriptional regulation
